# Synthesis of 4-Alkyl-4*H*-1,2,4-triazole Derivatives by Suzuki Cross-Coupling Reactions and Their Luminescence Properties

**DOI:** 10.3390/molecules24030652

**Published:** 2019-02-12

**Authors:** Monika Olesiejuk, Agnieszka Kudelko, Marcin Swiatkowski, Rafal Kruszynski

**Affiliations:** 1 Department of Chemical Organic Technology and Petrochemistry, The Silesian University of Technology, Krzywoustego 4, PL-44100 Gliwice, Poland; monika.olesiejuk@polsl.pl; 2 Department of X-ray Crystallography and Crystal Chemistry, Institute of General and Ecological Chemistry, Lodz University of Technology, Żeromskiego 116, PL-90924 Łódź, Poland; marcin.swiatkowski@p.lodz.pl (M.S.); rafal.kruszynski@p.lodz.pl (R.K.)

**Keywords:** Suzuki cross-coupling, heterocycles, 1,2,4-triazole derivatives, ionic liquids, luminescence properties

## Abstract

New derivatives of 4-alkyl-3,5-diaryl-4*H*-1,2,4-triazole were synthesized utilizing the Suzuki cross-coupling reaction. The presented methodology comprises of the preparation of bromine-containing 4-alkyl-4*H*-1,2,4-triazoles and their coupling with different commercially available boronic acids in the presence of ionic liquids or in conventional solvents. The obtained compounds were tested for their luminescence properties.

## 1. Introduction

Heterocyclic compounds containing 4*H*-1,2,4-triazole moiety are very attractive due to their wide range of biological activities [1], such as antibacterial [2], anticancer [3], anticonvulsant [4], anti-diabetic [5] or anti-neuropathic [6] properties. They are also applied in agriculture as fungicides and herbicides [7], and in industry as corrosion inhibitors [8,9] or in material science due to their valuable electronic and optical properties [10].

The most popular method for the synthesis of 3,4,5-trisubstituted 4*H*-1,2,4-triazole core [11,12] involves the reaction of diacylhydrazines with aromatic amines, in the presence of the dehydrating agent, e.g., phosphorus pentoxide [13], zinc chloride [14] or *N*,*N*′-diphenylphosphenimidous amide [15]. Other methods include the conversion of other heterocyclic systems, such as 1,3,4-oxadiazoles [16,17], 1,3,4-thiadiazoles [18] or dihydro-1,2,4,5-tetrazines [19].

Ionic liquids (IL) are considered modern green solvents, mainly due to their low vapor pressure, good thermal stability, and wide liquid regions [20,21,22]. Recent reports have also shown that IL can act as effective catalysts [23,24,25].

Our previous reports examined diacylhydrazines as potent reagents in the synthesis of heterocycles such as 1,3,4-oxadiazoles and 1,3,4-thiadiazoles [26,27], which were found to be valuable units in the synthesis of five-membered rings, allowing the formation of compounds bearing extended π-conjugated systems with excellent optical properties. Herein, we report the synthesis of 4*H*-1,2,4-triazole core as a structural analogue of our previously investigated heterocyclic arrangements, expecting that it will exhibit equally good features.

## 2. Results and Discussion

**Scheme 1:**
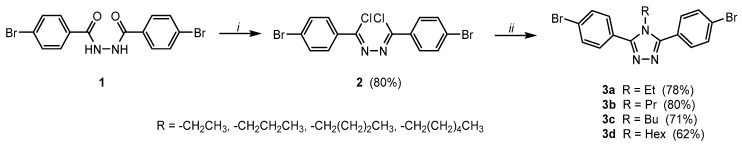
Synthesis of 4-alkyl-4*H*-1,2,4-triazole core. Reagents and conditions: (*i*) PCl_5_, toluene, reflux, 5 min; (*ii*) R-NH_2_, toluene, reflux, 10 h.

Our study began by synthesizing four basic 4-alkyl-3,5-bis(4-bromophenyl)-4*H*-1,2,4-triazole (**3a–d**) units (Scheme 1). The starting material in the reaction sequence was *N*,*N*′-bis(4-bromobenzoyl)hydrazine (**1**), which was prepared using previously described methods [26,28]. Compound **1** was transformed in 80% yield (entry 3, Table 1) into the more reactive chloro-derivative **2** in the presence of phosphorus pentachloride and in the non-polar solvent toluene. Heating compound **2** with excess butylamine generated the corresponding 4-butyl-4*H*-1,2,4-triazole derivative **3c** (71%, entry 3, Table 1). An attempt to extend the reaction time only slightly improved the yield (73%, entry 4, Table 1). Using our designed conditions, three other 4-alkyl-4*H*-1,2,4-triazole derivatives with phenyl groups with terminally substituted bromine were synthesized in good yields (**3a**, **3b**, **3d**, Scheme 1) and subsequently used as precursors for Suzuki cross-coupling reactions.

In order to select the most favorable conditions for the next stage—Suzuki cross-coupling—the selected dibromo 1,2,4-triazole derivative **3c** was treated with the phenylboronic acid (**4a**). Due to the use of a two-phase solvent system, it was necessary to employ a phase transfer catalyst. A better result was obtained when tetrabutylammonium bromide acted as the catalyst instead of its chloro counterpart (Table 2, entries 1 and 2). The use of a slight excess of a boronic acid with respect to the triazole derivative **3c** resulted in a significant improvement in yield (Table 2, entries 2 and 3). Then the influence of the type of base on the reaction yield was investigated (Table 2, entries 3–10). The study revealed that the best results in the two-phase solvent system were achieved with the use of different carbonates (Table 2, entries 3–6). Interestingly, it turned out that product **7a** can also be obtained in a single-phase system employing an organic base such as sodium alkoxide. In this case, the best result was obtained using sodium *t*-butoxide (Table 2, entry 10).

**Scheme 2:**
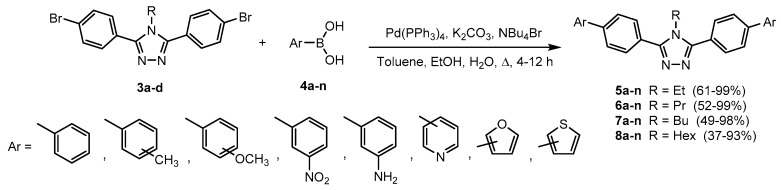
The conventional Suzuki cross-coupling reaction for 4-alkyl-3,5-bis(4-bromophenyl)-4*H*-1,2,4-triazole moiety. Reagents and conditions: aryl dibromide **3a–d** (1.00 mmol), arylboronic acid **4a–n** (2.50 mmol), Pd(PPh_3_)_4_ (0.05 mmol), NBu_4_Br (0.10 mmol), K_2_CO_3_ (10 mmol), toluene/H_2_O/EtOH (10:6:3 mL), oil bath 130 °C, 4–12 h.

Using these reaction conditions, new organic hybrids containing a 4-alkyl-4*H*-1,2,4-triazole moiety were synthesized (Scheme 2). 4-Alkyl-3,5-bis(4-bromophenyl)-4*H*-1,2,4-triazoles (**3a**–**d**) were first heated in an oil bath with excess boronic acids **4a**–**n** in the presence of 5 mol% palladium catalyst Pd(PPh_3_)_4_ and 10 mol% phase transfer catalyst NBu_4_Br. Reactions were conducted in a two-phase toluene/H_2_O/EtOH solvent system and monitored by TLC until the initial 4-alkyl-3,5-bis(4-bromophenyl)-4*H*-1,2,4-triazole (**3a**–**d**) was entirely consumed.

An alternative method was sought for 3,5-bis(biphenyl-4-yl)-4-butyl-4*H*-1,2,4-triazole (**7a**) synthesis. A commercially available IL was selected based its ability to act both as a solvent and base necessary in the catalytic cycle.

**Figure 1:**
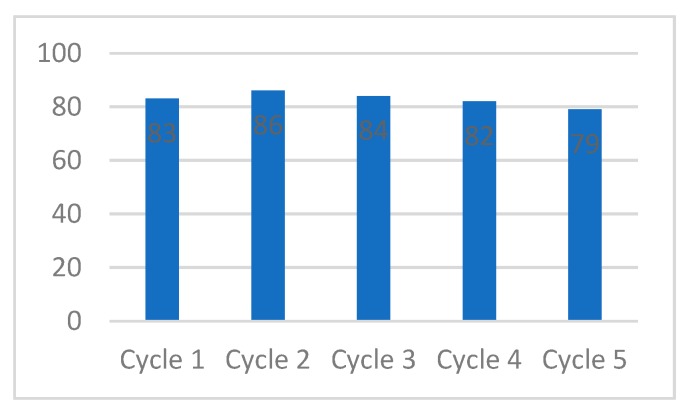
Yield of the selected product **7a** for the synthesis in choline ionic liquids.

Aqueous solutions of four different hydroxide IL containing different cations, i.e., benzyltrimethylammonium (BTMA-OH), choline (Choline-OH), hexadecyltrimethylammonium (HDTMA-OH) and tetrabutylammonium (TBA-OH), were tested. Due to the limited solubility of substrates in these solvents, the addition of 10% non-polar conventional solvent (toluene) was necessary. Interestingly, among the tested systems, only one IL, Choline-OH, could be used without the addition of a base, resulting in the formation of the product **7a** in 83% yield (entry 4, Table 3). We found that the IL can be regenerated and recycled into the reaction with only a slight decrease in product yield after five cycles (Figure 1).

Using our standard conditions, novel sets of compounds containing a 4-alkyl-4*H*-1,2,4-triazole moiety were synthesized (Table 4). Generally, compounds containing a 4*H*-1,2,4-triazole core substituted with an ethyl or propyl group at the position 4 were synthesized at slightly higher yields than their counterparts with butyl or hexyl substituents (61–99% for **5a**–**n**, 52–99% for **6a**–**n**, 49–98% for **7a–n**, 37–93% for **8a–n**, Table 4). Interestingly, the lowest yields were obtained for compounds **5m**, **6m**, **7m**, **8m**, containing a terminal thiophen-2-yl group (61% for **5m**, 52% for **6m**, 49% for **7m**, 37% for **8m**, Table 4). Other products possesing terminal electron-deficient and electron-rich substituted phenyl group or other heterocyclic arrangements were produced in relatively higher yields (85–99% for **5a**–**l,n**, 69–99% for **6a**–**l,n**, 74–98% for **7a**–**l,n**, 62–93% for **8a**–**l,n**, Table 4).

**Figure 2:**
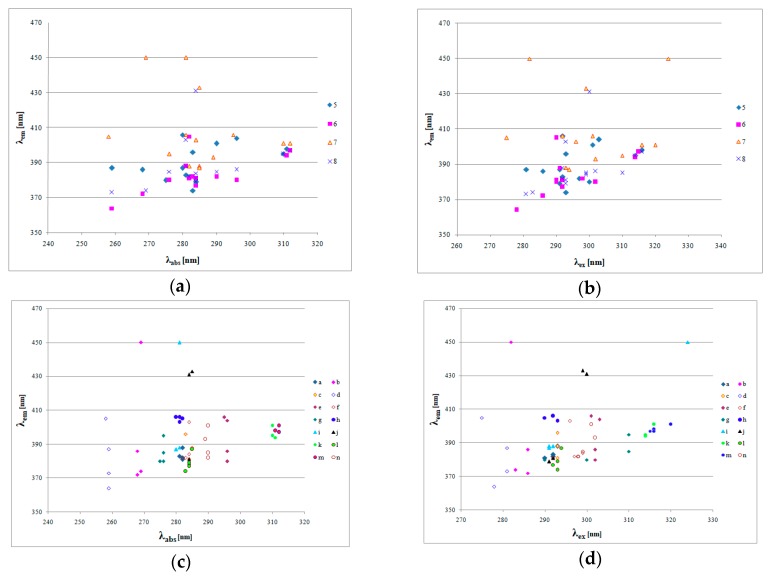
Absorption-emission or excitation-emission maxima of studied compounds: (**a**) Absorption-emission maxima of studied compounds containing ethyl (**5a–n**), propyl (**6a–n**), butyl (**7a–n**) and hexyl (**8a–n**) substituents; (**b**) excitation-emission maxima of studied compounds containing ethyl (**5a–n**), propyl (**6a–n**), butyl (**7a–n**) and hexyl (**8a–n**) substituents; (**c**) absorption-emission maxima of studied compounds containing different aryl substituents; (**d**) excitation-emission maxima of studied compounds containing different aryl substituents.

All the final products were screened for their luminescence properties, displaying strong fluorescent properties. The only exception occurred for compounds **5**–**8g** containing a nitrophenyl moiety (Table 4), due the presence of a strong electron withdrawing -NO_2_ group, which diminished the electron density within an aromatic system and subsequently changed the energy of delocalized orbitals accompanied by a decrease in population of fluorescent transitions. Generally, compounds containing aliphatic chains with an even number of carbon atoms exhibit longer emission wavelengths, than those of odd numbers (Table 4). This effect was independent from the absorption or excitation wavelengths (Figure 2a,b). The wavelengths of absorption and excitation maxima depended mainly on the Ar substituent type, i.e., for the same Ar substituent, these wavelengths are similar (Figure 2c,d).

**Figure 3:**
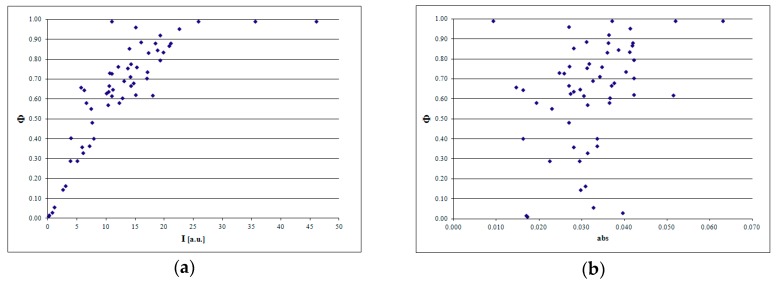
Quantum yield of studied compounds: (**a**) Quantum yield and intensity of fluorescence of studied compounds; (**b**) Quantum yield of fluorescence and absorbance at excitation wavelength ($\lambda_{max}^{ex}$) of studied compounds.

The calculated quantum yields (Φ) correlated with registered values of fluorescence (larger fluorescence leads to larger Φ, Figure 3a), whereas no relationship between the Φ and absorption was observed (Figure 3b). This meant that the emission mechanism was similar in all compounds and the differences in values of absorption originated from the variation in the amount of electromagnetic energy (photons) transformed into internal energy. The 3D fluorescence spectra in most cases displayed one well-shaped maximum, originating from the absorption-emission effects occurring within the central moiety. The exceptions are compounds **6i** and **8i**, containing 4-pyridyl substituent (spectrum of compound **6i** contains two maxima, and of **8i** exhibits large broadening at longer emission wavelengths). The presence of a fluorescent substituent (pyridyl) caused the formation of the second re-emission effect, partially overlapped with the fluorescence of the central moiety, influencing the shape of the emission spectrum. These changes were also observed in other synthesized compounds containing the pyridyl moiety (**5i**, **7i**, **5j**–**8j**); however, the broadening was less visible due to a smaller shift and larger overlap of the emission maxima. In all cases, the n→π* absorption transitions are the main origin of excited states leading to subsequent fluorescence.

**Figure 4:**
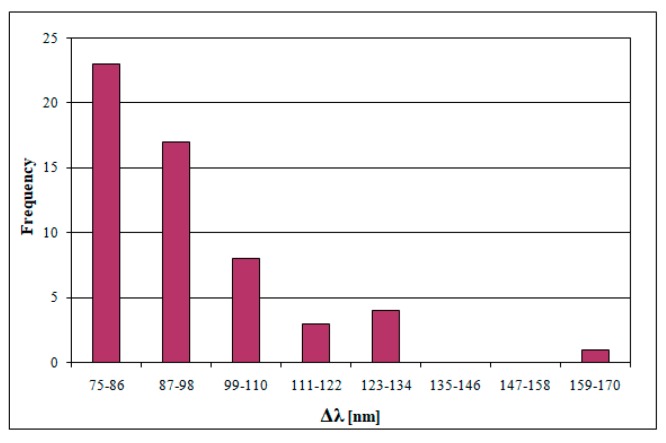
The distribution of the Δλ = $\lambda_{max}^{em}\;$ − $\lambda_{max}^{ex}$ for the studied compounds.

The determined optimal excitation wavelengths are similar to those registered for 1,2,4-triazole and certain bromo derivatives; however, in our case, the calculated quantum yields were much greater than the ones for 1,2,4-triazole and the above-mentioned derivatives (up to four orders of magnitudes, e.g., Φ_1,2,4-triazole_ = 5 × 10^4^) [32]. The differences between the excitation and emission wavelengths at global maxima of 3D fluorescence spectra vary from 75 nm to 168 nm, with the most populated value at ca. 80 nm (Figure 4). The largest shift in wavelengths occurred for compound **7b**, which upon absorption of ultraviolet light emitted a strong blue fluorescence. Such shift is rare, as typically fluorescent compounds possess emission wavelengths separated by less than 100 nm from excitation wavelengths. Upon irradiation by UV light, **7i** emitted a blue light and compounds **7j** and **8j** emitted a violet light visible by the naked eye.

## 3. Experimental

### 3.1. General Information

Melting points were measured on a Stuart SMP3 melting point apparatus. The ^1^H-NMR and ^13^C-NMR spectra were recorded on an Agilent 400-NMR spectrometer in CDCl_3_ solution using TMS as the internal standard. FT-IR spectra were performed between 4000 and 650 cm^−1^ using a FT-IR Nicolet 6700 apparatus with a Smart iTR accessory. UV-Vis spectra were registered at room temperature in CH_2_Cl_2_ on a Jasco V-660 spectrophotometer. Fluorescence spectra were registered at room temperature in CH_2_Cl_2_ using a Jasco F-6300 fluorescence spectrophotometer. High-resolution mass spectra were recorded on a Waters ACQUITY UPLC/Xevo G2QT instrument. Thin-layer chromatography was performed on silica gel 60 F_254_ (Merck, Darmstadt, Germany) TLC plates using CHCl_3_/EtOAc (5:1 *v*/*v*) as the mobile phase. All reagents were purchased from commercial sources and used without further purification. Aqueous solutions of four different hydroxide IL (Merck) containing different cations including: benzyltrimethylammonium hydroxide solution 40 wt. % in H_2_O (BTMA-OH), choline hydroxide solution 46 wt. % in H_2_O (Choline-OH), hexadecyltrimethylammonium hydroxide solution 10 wt. % in H_2_O (HDTMA-OH) and tetrabutylammonium hydroxide solution 40 wt. % in H_2_O (TBA-OH) were tested. Copies of the ^1^H-NMR and ^13^C-NMR spectra and 3D emission spectra of the compounds are available in the online Supplementary Materials.

### 3.2. Synthesis and Characterization

#### 3.2.1. Procedure for the Synthesis of Precursor 2

*N,N′-Bis[(4-bromophenyl)chloromethylene]hydrazine* (**2**). A mixture of *N,N′*-bis(4-bromobenzoyl)hydrazine (**1**, 3.981 g, 0.01 mol) with phosphorus pentachloride (4.164 g, 0.02 mol) in toluene (70 mL) was heated under reflux in an oil bath (130 °C) for 5 min. The clear yellow solution was evaporated under vacuum. The residue was then dissolved in chloroform (50 mL) and transferred to a separating funnel. The organic layer was washed with distilled water (5 × 50 mL), dried over MgSO_4_ and concentrated using a rotary evaporator. The crude yellow residue was purified by column chromatography (silica gel, CHCl_3_ as the mobile phase) to give *N*,*N*′-bis[(4-bromophenyl)chloromethylene]hydrazine (**2**, 3.490 g, 80% yield) as a light cream solid mp 143–145 °C (lit. [33]: 144–145 °C).

#### 3.2.2. General Procedure for the Synthesis of Suzuki Cross-Coupling Precursors: 4-alkyl-3,5-bis(4-bromophenyl)-4*H*-1,2,4-triazoles (**3a–d**)

To a cooled, vigorously stirred solution of *N*,*N*′-bis[(4-bromophenyl)chloromethylene]hydrazine (2, 4.349 g, 0.01 mol) in toluene (50 mL), the appropriate amine (0.04 mol) was added. The solution was stirred in an ice bath for 3 h and then at room temperature overnight, followed by heating for an additional 10 h before being concentrated using a rotary evaporator. The crude residue was purified by column chromatography (silica gel, CHCl_3_/EtOAc, 5:1 *v*/*v* as the mobile phase) to give 4-alkyl-3,5-bis(4-bromophenyl)-4*H*-1,2,4-triazole (**3a–d**).

*3,5-Bis(4-bromophenyl)-4-ethyl-4H-1,2,4-triazole* (**3a**). White solid in 78% yield, 3.175 g, m.p. 214–215 °C; UV (CH_2_Cl_2_) λ_max_ 257.5 nm (ε⋅10^−3^ 33.3 cm^−1^M^−1^); IR (ATR) ν: 2966, 1597, 1473, 1457, 1431, 1379, 1080, 1067, 1009, 968, 824, 794, 753, 737, 730, 665 cm^−1^; ^1^H-NMR (400 MHz, CDCl_3_): δ 1.07 (t, *J* = 7.2 Hz, 3H, CH_3_), 4.08 (q, *J* = 7.2 Hz, 2H, CH_2_), 7.52 (d, *J* = 8.4 Hz, 4H, ArH), 7.65 (d, *J* = 8.4 Hz, 4H, ArH); ^13^C-NMR (100 MHz, CDCl_3_): δ 15.8, 40.0, 124.9, 126.4, 130.4, 132.3, 154.5; HRMS *m/z* calcd for (C_16_H_13_N_3_Br_2_ + H^+^): 405.9549; found: 405.9544.

*3,5-Bis(4-bromophenyl)-4-propyl-4H-1,2,4-triazole* (**3b**). White solid in 80% yield, 3.369 g, m.p. 208–210 °C; UV (CH_2_Cl_2_) λ_max_ 258.5 nm (ε⋅10^−3^ 27.7 cm^−1^M^−1^); IR (ATR) ν: 2955, 2930, 2869, 1468, 1455, 1429, 1401, 1379, 1349, 1269, 1105, 1091, 1069, 1010, 967, 849, 827, 800, 746 cm^−1^; ^1^H-NMR (400 MHz, CDCl_3_): δ 0.63 (t, *J* = 7.2 Hz, 3H, CH_3_), 1.41 (sext, *J* = 7.2 Hz, 2H, CH_2_), 4.03 (t, *J* = 7.2 Hz, 2H, CH_2_), 7.54 (d, *J* = 8.4 Hz, 4H, ArH), 7.68 (d, *J* = 8.4 Hz, 4H, ArH); ^13^C-NMR (100 MHz, CDCl_3_): δ 10.6, 23.4, 46.6, 124.8, 126.6, 130.4, 132.3, 154.8; HRMS *m/z* calcd for (C_17_H_15_N_3_Br_2_ + H^+^): 419.9705; found: 419.9701.

*3,5-Bis(4-bromophenyl)-4-butyl-4H-1,2,4-triazole* (**3c**). White solid in 71% yield, 3.085 g, m.p. 220–223 °C; UV (CH_2_Cl_2_) λ_max_ 258.0 nm (ε⋅10^−3^ 28.0 cm^−1^M^−1^); IR (ATR) ν: 2963, 2927, 2856, 1468, 1430, 1400, 1382, 1268, 1096, 1069, 1011, 973, 846, 826, 756, 744, 720 cm^−1^; ^1^H-NMR (400 MHz, CDCl_3_): δ 0.67 (t, *J* = 7.6 Hz, 3H, CH_3_), 1.01 (sext, *J* = 7.6 Hz, 2H, CH_2_), 1.35 (quin, *J* = 7.6 Hz, 2H, CH_2_), 4.06 (t, *J* = 7.6 Hz, 2H, CH_2_), 7.54 (d, *J* = 8.4 Hz, 4H, ArH), 7.67 (d, *J* = 8.4 Hz, 4H, ArH); ^13^C-NMR (100 MHz, CDCl_3_): δ 13.1, 19.2, 31.9, 44.8, 124.7, 126.5, 130.4, 132.3, 154.8; HRMS *m/z* calcd for (C_18_H_17_N_3_Br_2_ + H^+^): 433.9862; found: 433.9863.

*3,5-Bis(4-bromophenyl)-4-hexyl-4H-1,2,4-triazole* (**3d**). White solid in 62% yield, 2.872 g, m.p. 168–169 °C; UV (CH_2_Cl_2_) λ_max_ 258.0 nm (ε⋅10^−3^ 31.3 cm^−1^M^−1^); IR (ATR) ν: 2951, 2931, 2860, 1599, 1464, 1417, 1398, 1381, 1353, 1331, 1147, 1101, 1071, 1014, 977, 853, 842, 825, 772, 753, 734, 720 cm^−1^; ^1^H-NMR (400 MHz, CDCl_3_): δ 0.76 (t, *J* = 7.2 Hz, 3H, CH_3_), 0.98 (m, 4H, 2 × CH_2_), 1.00 (quin, *J* = 7.2 Hz, 2H, CH_2_), 1.36 (quin, *J* = 7.2 Hz, 2H, CH_2_), 4.05 (t, *J* = 7.2 Hz, 2H, CH_2_), 7.54 (d, *J* = 8.4 Hz, 4H, ArH), 7.68 (d, *J* = 8.4 Hz, 4H, ArH); ^13^C-NMR (100 MHz, CDCl_3_): δ 13.7, 22.2, 25.6, 29.8, 30.6, 45.0, 124.7, 126.6, 130.4, 132.3, 154.8; HRMS *m/z* calcd for (C_20_H_21_N_3_Br_2_ + H^+^): 462.0175; found: 462.0177.

#### 3.2.3. General Procedure for Conventional Suzuki Cross-Coupling Reactions. Synthesis of 4-Alkyl-3,5-bis(4-arylphenyl)-4*H*-1,2,4-triazoles **5a–n**, **6a–n**, **7a–n**, **8a–n**

To a mixture of 4-alkyl-3,5-bis(4-bromophenyl)-4*H*-1,2,4-triazole (3a–d, 1.00 mmol), the corresponding boronic acid (4a–n, 2.50 mmol), palladium catalyst Pd(PPh_3_)_4_ (0.058 g, 0.05 mmol), phase transfer catalyst NBu_4_Br (0.032 g, 0.10 mmol) and base K_2_CO_3_ (1.382 g, 10.00 mmol), toluene (9 mL), H_2_O (6 mL) and EtOH (3 mL) were added. The mixture was heated under reflux in an oil bath (130 °C) for 4–12 h (reaction was monitored by TLC). After cooling, chloroform (50 mL) was added and the solution transferred to a separating funnel. The aqueous layer was extracted with chloroform (2 × 10 mL). The combined organic layers were filtered through a silica gel plug (10 mL), which was then flushed with CHCl_3_/EtOAc (5:1 *v*/*v*). The filtrate was dried over MgSO_4_ and then concentrated using a rotary evaporator. The product was precipitated using EtOAc (5 mL), filtered, washed with fresh EtOAc and air-dried to give the corresponding pure 4-alkyl-3,5-bis(4-arylphenyl)-4*H*-1,2,4-triazole (**5a–n**, **6a–n**, **7a–n**, **8a–n**).

*3,5-Bis(biphenyl-4-yl)-4-ethyl-4H-1,2,4-triazole* (**5a**). Beige solid in 87% yield, 0.348 g, m.p. 229–231 °C; UV (CH_2_Cl_2_) λ_max_ 281.0 nm (ε⋅10^−3^ 36.0 cm^−1^M^−1^); IR (ATR) ν: 3029, 2965, 1471, 1456, 1445, 1420, 1340, 1083, 1076, 1007, 968, 847, 765, 750, 730, 695, 598 cm^−1^; ^1^H-NMR (400 MHz, CDCl_3_): δ 1.16 (t, *J* = 7.2 Hz, 3H, CH_3_), 4.23 (q, *J* = 7.2 Hz, 2H, CH_2_), 7.39 (t, *J* = 7.2 Hz, 2H, ArH), 7.47 (t, *J* = 7.2 Hz, 4H, ArH), 7.64 (d, *J* = 7.2 Hz, 4H, ArH), 7.74–7.78 (m, 8H, ArH); ^13^C-NMR (100 MHz, CDCl_3_): δ 15.9, 40.0, 127.2, 127.6, 127.9, 128.3, 128.9, 129.3, 140.0, 142.9, 155.1; HRMS *m/z* calcd for (C_28_H_23_N_3_ + H^+^): 402.1965; found: 402.1963.

*4-Ethyl-3,5-bis(2′-methylbiphenyl-4-yl)-4H-1,2,4-triazole* (**5b**). White solid in 85% yield, 0.366 g, m.p. 247–250 °C; UV (CH_2_Cl_2_) λ_max_ 268.0 nm (ε⋅10^−3^ 32.6 cm^−1^M^−1^); IR (ATR) ν: 3060, 3016, 1473, 1454, 1425, 1379, 1109, 1051, 1007, 968, 849, 842, 835, 807, 765, 743, 729 cm^−1^; ^1^H-NMR (400 MHz, CDCl_3_): δ 1.22 (t, *J* = 7.2 Hz, 3H, CH_3_), 2.32 (s, 6H, 2 × CH_3_), 4.27 (q, *J* = 7.2 Hz, 2H, CH_2_), 7.28–7.31 (m, 8H, ArH), 7.50 (d, *J* = 8.4 Hz, 4H, ArH), 7.75 (d, *J* = 8.4 Hz, 4H, ArH); ^13^C-NMR (100 MHz, CDCl_3_): δ 16.0, 20.4, 40.0, 125.9, 126.2, 127.7, 128.7, 129.6, 129.8, 130.5, 135.3, 140.9, 143.8, 155.2; HRMS *m/z* calcd for (C_30_H_27_N_3_ + H^+^): 430.2278; found: 430.2274.

*4-Ethyl-3,5-bis(3′-methylbiphenyl-4-yl)-4H-1,2,4-triazole* (**5c**). White solid in 97% yield, 0.417 g, m.p. 194–195 °C; UV (CH_2_Cl_2_) λ_max_ 283.0 nm (ε⋅10^−3^ 41.3 cm^−1^M^−1^); IR (ATR) ν: 3015, 2983, 1740, 1474, 1418, 1371, 1337, 1236, 1113, 1083, 1042, 1018, 968, 853, 842, 780, 755, 730, 609 cm^−1^; ^1^H-NMR (400 MHz, CDCl_3_): δ 1.17 (t, *J* = 7.2 Hz, 3H, CH_3_), 2.45 (s, 6H, 2 × CH_3_), 4.23 (q, *J* = 7.2 Hz, 2H, CH_2_), 7.22 (d, *J* = 7.6 Hz, 2H, ArH), 7.37 (t, *J* = 7.6 Hz, 2H, ArH), 7.45–7.47 (m, 4H, ArH), 7.61–7.63 (m, 8H, ArH); ^13^C-NMR (100 MHz, CDCl_3_): δ 15.9, 21.5, 40.0, 124.3, 126.5, 127.6, 127.9, 128.7, 128.8, 129.3, 138.6, 140.1, 143.0, 155.2; HRMS *m/z* calcd for (C_30_H_27_N_3_ + H^+^): 430.2278; found: 430.2279.

*4-Ethyl-3,5-bis(2′,6′-dimethylbiphenyl-4-yl)-4H-1,2,4-triazole* (**5d**). White solid in 90% yield, 0.412 g, m.p. 326–329 °C; UV (CH_2_Cl_2_) λ_max_ 259.0 nm (ε⋅10^−3^ 28.5 cm^−1^M^−1^); IR (ATR) ν: 2984, 2917, 1482, 1466, 1442, 1426, 1384, 1163, 1112, 1004, 963, 846, 773, 728, 719 cm^−1^; ^1^H-NMR (400 MHz, CDCl_3_): δ 1.22 (t, *J* = 7.2 Hz, 3H, CH_3_), 2.08 (s, 12H, 4 × CH_3_), 4.29 (q, *J* = 7.2 Hz, 2H, CH_2_), 7.14 (d, *J* = 7.2 Hz, 4H, ArH), 7.19–7.23 (m, 2H, ArH), 7.34 (d, *J* = 8.4 Hz, 4H, ArH), 7.77 (d, *J* = 8.4 Hz, 4H, ArH); ^13^C-NMR (100 MHz, CDCl_3_): δ 15.8, 20.8, 40.0, 126.2, 127.4, 129.1, 129.8, 135.8, 140.8, 143.2, 155.3; HRMS *m/z* calcd for (C_32_H_31_N_3_ + H^+^): 458.2591; found: 458.2593.

*4-Ethyl-3,5-bis(2′-methoxybiphenyl-4-yl)-4H-1,2,4-triazole* (**5e**). Creamy solid in 91% yield, 0.420 g, m.p. 189–190 °C; UV (CH_2_Cl_2_) λ_max_ 272.0 nm (ε⋅10^−3^ 33.0 cm^−1^M^−1^) and 296.0 (33.2); IR (ATR) ν: 3016, 2941, 2838, 1595, 1493, 1470, 1432, 1400, 1258, 1244, 1123, 1053, 1021, 1004, 964, 855, 835, 803, 749, 734, 727 cm^−1^; ^1^H-NMR (400 MHz, CDCl_3_): δ 1.20 (t, *J* = 7.2 Hz, 3H, CH_3_), 3.85 (s, 6H, 2 × OCH_3_), 4.26 (q, *J* = 7.2 Hz, 2H, CH_2_), 7.02 (d, *J* = 8.4 Hz, 2H, ArH), 7.07 (t, *J* = 7.6 Hz, 2H, ArH), 7.35–7.40 (m, 4H, ArH), 7.71 (d, *J* = 8.4 Hz, 4H, ArH), 7.75 (d, *J* = 8.4 Hz, 4H, ArH); ^13^C-NMR (100 MHz, CDCl_3_): δ 16.0, 40.0, 55.6, 111.4, 121.0, 126.2, 128.5, 129.2, 129.6, 130.5, 130.8, 140.3, 155.3, 156.5; HRMS *m/z* calcd for (C_30_H_27_N_3_O_2_ + H^+^): 462.2176; found: 462.2175.

*4-Ethyl-3,5-bis(3′-methoxybiphenyl-4-yl)-4H-1,2,4-triazole* (**5f**). White solid in 99% yield, 0.457 g, m.p. 191–192 °C; UV (CH_2_Cl_2_) λ_max_ 282.0 nm (ε⋅10^−3^ 31.6 cm^−1^M^−1^); IR (ATR) ν: 3007, 2953, 2836, 1737, 1609, 1583, 1474, 1437, 1417, 1294, 1268, 1212, 1170, 1114, 1095, 1082, 1053, 1030, 1014, 853, 840, 768, 753, 730 cm^−1^; ^1^H-NMR (400 MHz, CDCl_3_): δ1.18 (t, *J* = 7.2 Hz, 3H, CH_3_), 3.90 (s, 6H, 2 × OCH_3_), 4.24 (q, *J* = 7.2 Hz, 2H, CH_2_), 6.95 (dd, *J* = 8.0 and 2.4 Hz, 2H, ArH), 7.19 (t, *J* = 2.4 Hz, 2H, ArH), 7.23–7.26 (m, 2H, ArH), 7.41 (t, *J* = 8.0 Hz, 2H, ArH), 7.74–7.78 (m, 8H, ArH); ^13^C-NMR (100 MHz, CDCl_3_): δ 15.9, 40.0, 55.4, 113.0, 113.3, 119.7, 126.8, 127.7, 129.3, 130.0, 141.6, 142.7, 155.1, 160.1; HRMS *m/z* calcd for (C_30_H_27_N_3_O_2_ + H^+^): 462.2176; found: 462.2170.

*4-Ethyl-3,5-bis(3′-nitrobiphenyl-4-yl)-4H-1,2,4-triazole* (**5g**). Yellow solid in 94% yield, 0.462 g, m.p. 246–248 °C; UV (CH_2_Cl_2_) λ_max_ 275.0 nm (ε⋅10^−3^ 57.4 cm^−1^M^−1^); IR (ATR) ν: 3076, 1517, 1485, 1469, 1346, 1287, 1103, 1086, 1010, 963, 877, 854, 837, 804, 776, 729, 798, 692 cm^−1^; ^1^H-NMR (400 MHz, CDCl_3_): δ 1.20 (t, *J* = 7.2 Hz, 3H, CH_3_), 4.26 (q, *J* = 7.2 Hz, 2H, CH_2_), 7.69 (t, *J* = 8.0 Hz, 2H, ArH), 7.83 (d, *J* = 8.4 Hz, 4H, ArH), 7.87 (d, *J* = 8.4 Hz, 4H, ArH), 8.00 (d, *J* = 8.0 Hz, 2H, ArH), 8.28 (d, *J* = 8.0 Hz, 2H, ArH), 8.54 (s, 2H, ArH); ^13^C-NMR (100 MHz, CDCl_3_): δ 15.9, 40.1, 122.0, 122.7, 127.8, 127.9, 129.7, 130.0, 133.0, 140.4, 141.7, 148.9, 154.9; HRMS *m/z* calcd for (C_28_H_21_N_5_O_4_ + H^+^): 492.1666; found: 492.1678.

*3,5-Bis(3′-aminobiphenyl-4-yl)-4-ethyl-4H-1,2,4-triazole* (**5h**). Beige solid in 99% yield, 0.428 g, m.p. 232–234 °C; UV (CH_2_Cl_2_) λ_max_ 258.0 nm (ε⋅10^−3^ 28.5 cm^−1^M^−1^) and 280.0 (32.2); IR (ATR) ν: 3455, 3426, 3348, 3305, 3188, 3046, 1621, 1598, 1566, 1473, 1448, 1426, 1313, 971, 888, 867, 840, 777, 748 cm^−1^; ^1^H-NMR (400 MHz, CDCl_3_): δ 1.16 (t, *J* = 7.2 Hz, 3H, CH_3_), 3.80 (br.s, 4H, 2 × NH_2_), 4.22 (q, *J* = 7.2 Hz, 2H, CH_2_), 6.72 (d, *J* = 7.6 Hz, 2H, ArH), 6.97 (s, 2H, ArH), 7.05 (d, *J* = 7.6 Hz, 2H, ArH), 7.27 (t, *J* = 7.6 Hz, 2H, ArH), 7.72-7.75 (m, 8H, ArH); ^13^C-NMR (100 MHz, CDCl_3_): δ 15.9, 40.0, 113.8, 114.7, 117.6, 126.5, 127.5, 129.2, 129.9, 141.3, 143.0, 146.9, 155.2; HRMS *m/z* calcd for (C_28_H_25_N_5_ + H^+^): 432.2183; found: 432.2169.

*4-Ethyl-3,5-bis[4-(pyridin-4-yl)phenyl]-4H-1,2,4-triazole* (**5i**). Grey solid in 89% yield, 0.359 g, m.p. 248–251 °C; UV (CH_2_Cl_2_) λ_max_ 280.0 nm (ε⋅10^−3^ 31.0 cm^−1^M^−1^); IR (ATR) ν: 3067, 1594, 1541, 1474, 1411, 1221, 993, 969, 858, 808, 771, 751, 733, 664 cm^−1^; ^1^H-NMR (400 MHz, CDCl_3_): δ 1.19 (t, *J* = 7.2 Hz, 3H, CH_3_), 4.25 (q, *J* = 7.2 Hz, 2H, CH_2_), 7.57 (d, *J* = 6.0 Hz, 4H, ArH), 7.82 (d, *J* = 8.4 Hz, 4H, ArH), 7.85 (d, *J* = 8.4 Hz, 4H, ArH), 8.72 (d, *J* = 6.0 Hz, 4H, ArH); ^13^C-NMR (100 MHz, CDCl_3_): δ 15.9, 40.1, 121.6, 127.6, 128.3, 129.6, 139.9, 147.1, 150.5, 154.8; HRMS *m/z* calcd for (C_26_H_21_N_5_ + H^+^): 404.1870; found: 404.1868.

*4-Ethyl-3,5-bis[4-(pyridin-3-yl)phenyl]-4H-1,2,4-triazole* (**5j**). Yellow solid in 93% yield, 0.374 g, m.p. 199–202 °C; UV (CH_2_Cl_2_) λ_max_ 284.0 nm (ε⋅10^−3^ 54.8 cm^−1^M^−1^); IR (ATR) ν: 3025, 1464, 1433, 1416, 1383, 1025, 999, 971, 855, 848, 806, 772, 749, 712 cm^−1^; ^1^H-NMR (400 MHz, CDCl_3_): δ 1.19 (t, *J* = 7.2 Hz, 3H, CH_3_), 4.26 (q, *J* = 7.2 Hz, 2H, CH_2_), 7.42 (dd, *J* = 7.6 and 5.2 Hz, 2H, ArH), 7.77 (d, *J* = 8.4 Hz, 4H, ArH), 7.84 (d, *J* = 8.4 Hz, 4H, ArH), 7.95 (d, *J* = 7.6 Hz, 2H, ArH), 8.66 (dd, *J* = 5.2 and 1.6 Hz, 2H, ArH), 8.93 (d, *J* = 1.6 Hz, 2H, ArH); ^13^C-NMR (100 MHz, CDCl_3_): δ 15.9, 40.1, 123.7, 127.4, 127.7, 129.6, 134.4, 135.5, 139.6, 148.3, 149.1, 154.9; HRMS *m/z* calcd for (C_26_H_21_N_5_ + H^+^): 404.1870; found: 404.1870.

*4-Ethyl-3,5-bis[4-(furan-2-yl)phenyl]-4H-1,2,4-triazole* (**5k**). Creamy solid in 92% yield, 0.351 g, m.p. 236–237 °C; UV (CH_2_Cl_2_) λ_max_ 310.0 nm (ε⋅10^−3^ 53.0 cm^−1^M^−1^); IR (ATR) ν: 3114, 1615, 1493, 1471, 1189, 1158, 1083, 1007, 967, 905, 884, 848, 833, 809, 792, 744, 719 cm^−1^; ^1^H-NMR (400 MHz, CDCl_3_): δ 1.13 (t, *J* = 7.2 Hz, 3H, CH_3_), 4.19 (q, *J* = 7.2 Hz, 2H, CH_2_), 6.52 (dd, *J* = 3.6 and 2.0 Hz, 2H, ArH), 6.78 (d, *J* = 3.6 Hz, 2H, ArH), 7.53 (d, *J* = 2.0 Hz, 2H, ArH), 7.71 (d, *J* = 8.8 Hz, 4H, ArH), 7.83 (d, *J* = 8.8 Hz, 4H, ArH); ^13^C-NMR (100 MHz, CDCl_3_): δ 15.7, 40.0, 106.4, 111.9, 124.1, 126.3, 129.2, 132.3, 142.8, 153.0, 155.1; HRMS *m/z* calcd for (C_24_H_19_N_3_O_2_ + H^+^): 382.1550; found: 382.1554.

*4-Ethyl-3,5-bis[4-(furan-3-yl)phenyl]-4H-1,2,4-triazole* (**5l**). Creamy solid in 91% yield, 0.347 g, m.p. 246–248 °C; UV (CH_2_Cl_2_) λ_max_ 283.0 nm (ε⋅10^−3^ 27.7 cm^−1^M^−1^); IR (ATR) ν: 3143, 2969, 1507, 1472, 1403, 1350, 1195, 1162, 1115, 1084, 1054, 1016, 923, 872, 843, 785, 760, 739 cm^−1^; ^1^H-NMR (400 MHz, CDCl_3_): δ 1.13 (t, *J* = 7.2 Hz, 3H, CH_3_), 4.18 (q, *J* = 7.2 Hz, 2H, CH_2_), 6.76 (dd, *J* = 1.6 and 0.8 Hz, 2H, ArH), 7.53 (t, *J* = 1.6 Hz, 2H, ArH), 7.65 (d, *J* = 8.8 Hz, 4H, ArH), 7.71 (d, *J* = 8.8 Hz, 4H, ArH), 7.83 (dd, *J* = 1.6 and 0.8 Hz, 2H, ArH); ^13^C-NMR (100 MHz, CDCl_3_): δ 15.8, 39.9, 108.7, 125.7, 126.2, 126.3, 129.4, 134.2, 139.2, 144.1, 155.1; HRMS *m/z* calcd for (C_24_H_19_N_3_O_2_ + H^+^): 382.1550; found: 382.1548.

*4-Ethyl-3,5-bis[4-(thiophen-2-yl)phenyl]-4H-1,2,4-triazole* (**5m**). Creamy solid in 61% yield, 0.254 g, m.p. 239–242 °C; UV (CH_2_Cl_2_) λ_max_ 311.0 nm (ε⋅10^−3^ 43.2 cm^−1^M^−1^); IR (ATR) ν: 3089, 3004, 2961, 1470, 1428, 1353, 1260, 1216, 1194, 1116, 1081, 1012, 967, 959, 853, 843, 819, 785, 773, 740, 704, 693 cm^−1^; ^1^H-NMR (400 MHz, CDCl_3_): δ 1.14 (t, *J* = 7.2 Hz, 3H, CH_3_), 4.20 (q, *J* = 7.2 Hz, 2H, CH_2_), 7.12 (dd, *J* = 5.2 and 3.6 Hz, 2H, ArH), 7.35 (d, *J* = 5.2 Hz, 2H, ArH), 7.42 (d, *J* = 3.6 Hz, 2H, ArH), 7.70 (d, *J* = 8.4 Hz, 4H, ArH), 7.77 (d, *J* = 8.4 Hz, 4H, ArH); ^13^C-NMR (100 MHz, CDCl_3_): δ 15.8, 40.0, 124.0, 125.8, 126.2, 126.5, 128.3, 129.4, 136.0, 143.1, 155.0; HRMS *m/z* calcd for (C_24_H_19_N_3_S_2_ + H^+^): 414.1093; found: 414.1077.

*4-Ethyl-3,5-bis[4-(thiophen-3-yl)phenyl]-4H-1,2,4-triazole* (**5n**). Beige solid in 90% yield, 0.372 g, m.p. 265–266 °C; UV (CH_2_Cl_2_) λ_max_ 290.0 nm (ε⋅10^−3^ 42.5 cm^−1^M^−1^); IR (ATR) ν: 3094, 2962, 1470, 1352, 1278, 1205, 1119, 1083, 1017, 968, 870, 843, 780, 760, 733, 695, 667, 631 cm^−1^; ^1^H-NMR (400 MHz, CDCl_3_): δ 1.14 (t, *J* = 7.2 Hz, 3H, CH_3_), 4.20 (q, *J* = 7.2 Hz, 2H, CH_2_), 7.43-7.47 (m, 4H, ArH), 7.57 (dd, *J* = 2.8 and 1.6 Hz, 2H, ArH), 7.72 (d, *J* = 8.4 Hz, 4H, ArH), 7.77 (d, *J* = 8.4 Hz, 4H, ArH); ^13^C-NMR (100 MHz, CDCl_3_): δ 15.8, 40.0, 121.3, 126.1, 126.3, 126.7, 126.8, 129.4, 137.4, 141.2, 155.1; HRMS *m/z* calcd for (C_24_H_19_N_3_S_2_ + H^+^): 414.1093; found: 414.1102.

*3,5-Bis(biphenyl-4-yl)-4-propyl-4H-1,2,4-triazole* (**6a**). Creamy solid in 94% yield, 0.391 g, m.p. 285–288 °C; UV (CH_2_Cl_2_) λ_max_ 282.0 nm (ε⋅10^−3^ 41.8 cm^−1^M^−1^); IR (ATR) ν: 3058, 2958, 2934, 2874, 1467, 1446, 1420, 1397, 1385, 1349, 1005, 969, 843, 802, 767, 745, 734, 724, 688 cm^−1^; ^1^H-NMR (400 MHz, CDCl_3_): δ 0.68 (t, *J* = 7.6 Hz, 3H, CH_3_), 1.50 (sext, *J* = 7.6 Hz, 2H, CH_2_), 4.16 (t, *J* = 7.6 Hz, 2H, CH_2_), 7.40 (t, *J* = 7.2 Hz, 2H, ArH), 7.49 (t, *J* = 7.2 Hz, 4H, ArH), 7.67 (d, *J* = 7.2 Hz, 4H, ArH), 7.75–7.79 (m, 8H, ArH); ^13^C-NMR (100 MHz, CDCl_3_): δ 10.7, 23.5, 46.6, 126.7, 127.1, 127.6, 127.9, 128.9, 129.3, 140.0, 142.8, 155.4; HRMS *m/z* calcd for (C_29_H_25_N_3_ + H^+^): 416.2121; found: 416.2120.

*3,5-Bis(2′-methylbiphenyl-4-yl)-4-propyl-4H-1,2,4-triazole* (**6b**). White solid in 81% yield, 0.360 g, m.p. 230–232 °C; UV (CH_2_Cl_2_) λ_max_ 268.0 nm (ε⋅10^−3^ 37.9 cm^−1^M^−1^); IR (ATR) ν: 3052, 2961, 2873, 1474, 1425, 1382, 1112, 1093, 1007, 972, 864, 847, 768, 747, 722 cm^−1^; ^1^H-NMR (400 MHz, CDCl_3_): δ 0.71 (t, *J* = 7.2 Hz, 3H, CH_3_), 1.54 (sext, *J* = 7.2 Hz, 2H, CH_2_), 2.32 (s, 6H, 2 × CH_3_), 4.19 (t, *J* = 7.2 Hz, 2H, CH_2_), 7.28–7.31 (m, 8H, ArH), 7.50 (d, *J* = 8.0 Hz, 4H, ArH), 7.75 (d, *J* = 8.0 Hz, 4H, ArH); ^13^C-NMR (100 MHz, CDCl_3_): δ 10.7, 20.4, 23.5, 46.6, 125.9, 126.3, 127.7, 128.7, 129.6, 129.8, 130.5, 135.3, 140.9, 143.8, 155.5; HRMS *m/z* calcd for (C_31_H_29_N_3_ + H^+^): 444.2434; found: 444.2432.

*3,5-Bis(3′-methylbiphenyl-4-yl)-4-propyl-4H-1,2,4-triazole* (**6c**). White solid in 91% yield, 0.404 g, m.p. 223–225 °C; UV (CH_2_Cl_2_) λ_max_ 284.0 nm (ε⋅10^−3^ 45.2 cm^−1^M^−1^); IR (ATR) ν: 3050, 2967, 2877, 1607, 1470, 1414, 1382, 1336, 1093, 1020, 969, 863, 852, 838, 775, 752, 725 cm^−1^; ^1^H-NMR (400 MHz, CDCl_3_): δ 0.68 (t, *J* = 7.6 Hz, 3H, CH_3_), 1.50 (sext, *J* = 7.6 Hz, 2H, CH_2_), 2.45 (s, 6H, 2 × CH_3_), 4.15 (t, *J* = 7.6 Hz, 2H, CH_2_), 7.22 (d, *J* = 7.6 Hz, 2H, ArH), 7.38 (t, *J* = 7.6 Hz, 2H, ArH), 7.45–7.48 (m, 4H, ArH), 7.74–7.77 (m, 8H, ArH); ^13^C-NMR (100 MHz, CDCl_3_): δ 10.7, 21.5, 23.5, 46.6, 124.3, 126.6, 127.6, 127.9, 128.7, 128.9, 129.3, 138.6, 140.0, 142.9, 155.5; HRMS *m/z* calcd for (C_31_H_29_N_3_ + H^+^): 444.2434; found: 444.2430.

*3,5-Bis(2′,6′-dimethylbiphenyl-4-yl)-4-propyl-4H-1,2,4-triazole* (**6d**). White solid in 94% yield, 0.444 g, m.p. 285–286 °C; UV (CH_2_Cl_2_) λ_max_ 259.0 nm (ε⋅10^−3^ 29.5 cm^−1^M^−1^); IR (ATR) ν: 3035, 2969, 1466, 1424, 1379, 1092, 1004, 973, 863, 841, 765, 751, 729 cm^−1^; ^1^H-NMR (400 MHz, CDCl_3_): δ 0.69 (t, *J* = 7.2 Hz, 3H, CH_3_), 1.53 (sext, *J* = 7.2 Hz, 2H, CH_2_), 2.07 (s, 12H, 4 × CH_3_), 4.20 (t, *J* = 7.2 Hz, 2H, CH_2_), 7.14 (d, *J* = 7.2 Hz, 4H, ArH), 7.19–7.23 (m, 2H, ArH), 7.33 (d, *J* = 8.4 Hz, 4H, ArH), 7.77 (d, *J* = 8.4 Hz, 4H, ArH); ^13^C-NMR (100 MHz, CDCl_3_): δ 10.7, 20.8, 23.4, 46.6, 99.1, 126.3, 127.4, 129.1, 129.8, 135.8, 140.8, 143.1, 155.6; HRMS *m/z* calcd for (C_33_H_33_N_3_ + H^+^): 472.2747; found: 472.2744.

*3,5-Bis(2′-methoxybiphenyl-4-yl)-4-propyl-4H-1,2,4-triazole* (**6e**). White solid in 91% yield, 0.433 g, m.p. 198–199 °C; UV (CH_2_Cl_2_) λ_max_ 273.0 nm (ε⋅10^−3^ 27.7 cm^−1^M^−1^) and 296.0 (28.6); IR (ATR) ν: 3037, 2933, 2830, 1598, 1495, 1465, 1421, 1257, 1239, 1122, 1112, 1055, 1024, 1005, 860, 839, 803, 735, 721 cm^−1^; ^1^H-NMR (400 MHz, CDCl_3_): δ 0.71 (t, *J* = 7.6 Hz, 3H, CH_3_), 1.55 (sext, *J* = 7.6 Hz, 2H, CH_2_), 3.85 (s, 6H, 2 × OCH_3_), 4.18 (t, *J* = 7.6 Hz, 2H, CH_2_), 7.02 (d, *J* = 8.4 Hz, 2H, ArH), 7.07 (t, *J* = 7.6 Hz, 2H, ArH), 7.35-7.40 (m, 4H, ArH), 7.70 (d, *J* = 8.4 Hz, 4H, ArH), 7.74 (d, *J* = 8.4 Hz, 4H, ArH); ^13^C-NMR (100 MHz, CDCl_3_): δ 10.7, 23.5, 46.6, 55.6, 111.4, 121.0, 126.3, 128.5, 129.2, 129.6, 130.0, 130.8, 140.3, 155.6, 156.5; HRMS *m/z* calcd for (C_31_H_29_N_3_O_2_ + H^+^): 476.2333; found: 476.2332.

*3,5-Bis(3′-methoxybiphenyl-4-yl)-4-propyl-4H-1,2,4-triazole* (**6f**). White solid in 95% yield, 0.452 g, m.p. 193–195 °C; UV (CH_2_Cl_2_) λ_max_ 283.0 nm (ε⋅10^−3^ 35.5 cm^−1^M^−1^); IR (ATR) ν: 2969, 2836, 1600, 1592, 1560, 1463, 1418, 1301, 1219, 1166, 1050, 1029, 1016, 858, 869, 849, 840, 795, 778, 753, 744 cm^−1^; ^1^H-NMR (400 MHz, CDCl_3_): δ 0.68 (t, *J* = 7.6 Hz, 3H, CH_3_), 1.50 (sext, *J* = 7.6 Hz, 2H, CH_2_), 3.90 (s, 6H, 2 × OCH_3_), 4.15 (t, *J* = 7.6 Hz, 2H, CH_2_), 6.95 (dd, *J* = 8.0 and 2.4 Hz, 2H, ArH), 7.19 (t, *J* = 2.4 Hz, 2H, ArH), 7.23–7.26 (m, 2H, ArH), 7.40 (t, *J* = 8.0 Hz, 2H, ArH), 7.74–7.78 (m, 8H, ArH); ^13^C-NMR (100 MHz, CDCl_3_): δ 10.7, 23.5, 46.6, 55.4, 112.9, 113.3, 119.6, 126.9, 127.6, 129.3, 130.0, 141.5, 142.6, 155.4, 160.1; HRMS *m/z* calcd for (C_31_H_29_N_3_O_2_ + H^+^): 476.2333; found: 476.2334.

*3,5-Bis(3′-nitrobiphenyl-4-yl)-4-propyl-4H-1,2,4-triazole* (**6g**). Creamy solid in 91% yield, 0.460 g, m.p. 291–293 °C; UV (CH_2_Cl_2_) λ_max_ 276.0 nm (ε⋅10^−3^ 47.3 cm^−1^M^−1^); IR (ATR) ν: 3034, 2969, 2872, 1521, 1488, 1468, 1344, 1294, 1104, 876, 852, 840, 802, 777, 759, 742, 723 cm^−1^; ^1^H-NMR (400 MHz, CDCl_3_): δ 0.70 (t, *J* = 7.2 Hz, 3H, CH_3_), 1.52 (sext, *J* = 7.2 Hz, 2H, CH_2_), 4.19 (t, *J* = 7.2 Hz, 2H, CH_2_), 7.68 (t, *J* = 7.6 Hz, 2H, ArH), 7.82 (d, *J* = 8.4 Hz, 4H, ArH), 7.86 (d, *J* = 8.4 Hz, 4H, ArH), 8.00 (dd, *J* = 7.6 and 2.0 Hz, 2H, ArH), 8.27 (dd, *J* = 7.6 and 2.0 Hz, 2H, ArH), 8.54 (t, *J* = 2.0 Hz, 2H, ArH); ^13^C-NMR (100 MHz, CDCl_3_): δ 10.7, 23.6, 46.8, 122.0, 122.7, 127.8, 128.0, 129.7, 130.0, 133.0, 137.2, 140.3, 141.7, 155.2; HRMS *m/z* calcd for (C_29_H_23_N_5_O_4_ + H^+^): 506.1823; found: 506.1811.

*3,5-Bis(3′-aminobiphenyl-4-yl)-4-propyl-4H-1,2,4-triazole* (**6h**). Beige solid in 99% yield, 0.428 g, m.p. 216–218 °C; UV (CH_2_Cl_2_) λ_max_ 235.0 nm (ε⋅10^−3^ 27.0 cm^−1^M^−1^), 258.0 (29.2) and 282.0 (33.9); IR (ATR) ν: 3441, 3407, 3336, 3143, 2961, 1601, 1586, 1564, 1479, 1431, 1324, 1233, 993, 893, 842, 775, 743, 714 cm^−1^; ^1^H-NMR (400 MHz, CDCl_3_): δ 0.67 (t, *J* = 7.2 Hz, 3H, CH_3_), 1.49 (sext, *J* = 7.2 Hz, 2H, CH_2_), 3.80 (br.s, 4H, 2x NH_2_), 4.13 (t, *J* = 7.2 Hz, 2H, CH_2_), 6.72 (dd, *J* = 8.0 and 2.0 Hz, 2H, ArH), 6.98 (t, *J* = 2.0 Hz, 2H, ArH), 7.05 (dd, *J* = 8.0 and 2.0 Hz, 2H, ArH), 7.27 (t, *J* = 8.0 Hz, 2H, ArH), 7.72–7.74 (m, 8H, ArH); ^13^C-NMR (100 MHz, CDCl_3_): δ 10.7, 23.5, 46.6, 113.8, 114.7, 117.5, 126.6, 127.5, 129.2, 129.9, 141.2, 143.0, 146.9, 155.5; HRMS *m/z* calcd for (C_29_H_27_N_5_ + H^+^): 446.2339; found: 446.2348.

*4-Propyl-3,5-bis[4-(pyridin-4-yl)phenyl]-4H-1,2,4-triazole* (**6i**). Creamy solid in 90% yield, 0.376 g, m.p. 237–240 °C; UV (CH_2_Cl_2_) λ_max_ 281.0 nm (ε⋅10^−3^ 36.5 cm^−1^M^−1^); IR (ATR) ν: 2957, 1596, 1541, 1471, 1408, 1347, 1217, 992, 969, 859, 812, 772, 752, 737 cm^−1^; ^1^H-NMR (400 MHz, CDCl_3_): δ 0.69 (t, *J* = 7.6 Hz, 3H, CH_3_), 1.50 (sext, *J* = 7.6 Hz, 2H, CH_2_), 4.17 (t, *J* = 7.6 Hz, 2H, CH_2_), 7.58 (d, *J* = 6.0 Hz, 4H, ArH), 7.81–7.84 (m, 8H, ArH), 8.73 (d, *J* = 6.0 Hz, 4H, ArH); ^13^C-NMR (100 MHz, CDCl_3_): δ 10.7, 23.5, 46.7, 121.6, 127.6, 128.4, 129.6, 139.8, 147.1, 150.5, 155.2; HRMS *m/z* calcd for (C_27_H_23_N_5_ + H^+^): 418.2026; found: 418.2022.

*4-Propyl-3,5-bis[4-(pyridin-3-yl)phenyl]-4H-1,2,4-triazole* (**6j**). Creamy solid in 69% yield, 0.288 g, m.p. 212–215 °C; UV (CH_2_Cl_2_) λ_max_ 284.0 nm (ε⋅10^−3^ 40.6 cm^−1^M^−1^); IR (ATR) ν: 3035, 2965, 2875, 1470, 1426, 1414, 1383, 1339, 1025, 1001, 970, 851, 799, 749, 707 cm^−1^; ^1^H-NMR (400 MHz, CDCl_3_): δ 0.69 (t, *J* = 7.6 Hz, 3H, CH_3_), 1.51 (sext, *J* = 7.6 Hz, 2H, CH_2_), 4.18 (t, *J* = 7.6 Hz, 2H, CH_2_), 7.42 (dd, *J* = 8.0 and 4.8 Hz, 2H, ArH), 7.77 (d, *J* = 8.4 Hz, 4H, ArH), 7.83 (d, *J* = 8.4 Hz, 4H, ArH), 7.95 (dt, *J* = 8.0 and 1.6 Hz, 2H, ArH), 8.66 (dd, *J* = 4.8 and 1.6 Hz, 2H, ArH), 8.93 (d, *J* = 1.6 Hz, 2H, ArH); ^13^C-NMR (100 MHz, CDCl_3_): δ 10.7, 23.5, 46.7, 123.7, 127.5, 127.7, 129.6, 134.4, 135.5, 139.5, 148.3, 149.1, 155.2; HRMS *m/z* calcd for (C_27_H_23_N_5_ + H^+^): 418.2026; found: 418.2028.

*3,5-Bis[4-(furan-2-yl)phenyl]-4-propyl-4H-1,2,4-triazole* (**6k**). Beige solid in 96% yield, 0.379 g, m.p. 279–281 °C; UV (CH_2_Cl_2_) λ_max_ 311.0 nm (ε⋅10^−3^ 63.6 cm^−1^M^−1^); IR (ATR) ν: 2960, 1615, 1491, 1465, 1351, 1282, 1220, 1116, 1076, 1020, 1010, 903, 862, 839, 815, 805, 757, 735, 663 cm^−1^; ^1^H-NMR (400 MHz, CDCl_3_): δ 0.64 (t, *J* = 7.6 Hz, 3H, CH_3_), 1.45 (sext, *J* = 7.6 Hz, 2H, CH_2_), 4.10 (t, *J* = 7.6 Hz, 2H, CH_2_), 6.53 (dd, *J* = 3.6 and 2.0 Hz, 2H, ArH), 6.78 (d, *J* = 3.6 Hz, 2H, ArH), 7.53 (d, *J* = 2.0 Hz, 2H, ArH), 7.71 (d, *J* = 8.8 Hz, 4H, ArH), 7.83 (d, *J* = 8.8 Hz, 4H, ArH); ^13^C-NMR (100 MHz, CDCl_3_): δ 10.7, 23.4, 46.7, 106.4, 111.9, 124.1, 126.4, 129.2, 132.3, 142.8, 153.0, 155.4; HRMS *m/z* calcd for (C_25_H_21_N_3_O_2_ + H^+^): 396.1707; found: 396.1709.

*3,5-Bis[4-(furan-3-yl)phenyl]-4-propyl-4H-1,2,4-triazole* (**6l**). Creamy solid in 92% yield, 0.363 g, m.p. 299–303 °C; UV (CH_2_Cl_2_) λ_max_ 284.0 nm (ε⋅10^−3^ 35.3 cm^−1^M^−1^); IR (ATR) ν: 3141, 1507, 1471, 1351, 1162, 1115, 1055, 1015, 923, 872, 843, 785, 754, 739 cm^−1^; ^1^H-NMR (400 MHz, CDCl_3_): δ 0.65 (t, *J* = 7.6 Hz, 3H, CH_3_), 1.46 (sext, *J* = 7.6 Hz, 2H, CH_2_), 4.10 (t, *J* = 7.6 Hz, 2H, CH_2_), 6.77 (dd, *J* = 2.0 and 1.2 Hz, 2H, ArH), 7.53 (d, *J* = 2.0 Hz, 2H, ArH), 7.65 (d, *J* = 8.8 Hz, 4H, ArH), 7.70 (d, *J* = 8.8 Hz, 4H, ArH), 7.84 (d, *J* = 1.2 Hz, 2H, ArH); ^13^C-NMR (100 MHz, CDCl_3_): δ 10.7, 23.4, 46.6, 108.6, 125.7, 126.2, 126.3, 129.4, 134.2, 139.2, 144.1, 155.4; HRMS *m/z* calcd for (C_25_H_21_N_3_O_2_ + H^+^): 396.1707; found: 396.1710.

*4-Propyl-3,5-bis[4-(thiophen-2-yl)phenyl]-4H-1,2,4-triazole* (**6m**). Grey solid in 52% yield, 0.223 g, m.p. 283–285 °C; UV (CH_2_Cl_2_) λ_max_ 312.0 nm (ε⋅10^−3^ 36.4 cm^−1^M^−1^); IR (ATR) ν: 3091, 2959, 2873, 1469, 1427, 1398, 1352, 1260, 1216, 1193, 1116, 1093, 1013, 970, 959, 844, 819, 757, 741, 707, 694 cm^−1^; ^1^H-NMR (400 MHz, CDCl_3_): δ 0.66 (t, *J* = 7.6 Hz, 3H, CH_3_), 1.48 (sext, *J* = 7.6 Hz, 2H, CH_2_), 4.12 (t, *J* = 7.6 Hz, 2H, CH_2_), 7.13 (dd, *J* = 4.8 and 3.6 Hz, 2H, ArH), 7.36 (d, *J* = 4.8 Hz, 2H, ArH), 7.43 (d, *J* = 3.6 Hz, 2H, ArH), 7.71 (d, *J* = 8.4 Hz, 4H, ArH), 7.78 (d, *J* = 8.4 Hz, 4H, ArH); ^13^C-NMR (100 MHz, CDCl_3_): δ 10.7, 23.5, 46.7, 124.0, 125.8, 126.2, 126.6, 128.3, 129.4, 136.0, 143.2, 155.3; HRMS *m/z* calcd for (C_25_H_21_N_3_S_2_ + H^+^): 428.1250; found: 428.1241.

*4-Propyl-3,5-bis[4-(thiophen-3-yl)phenyl]-4H-1,2,4-triazole* (**6n**). Beige solid in 90% yield, 0.385 g, m.p. 302–305 °C; UV (CH_2_Cl_2_) λ_max_ 290.0 nm (ε⋅10^−3^ 44.8 cm^−1^M^−1^); IR (ATR) ν: 3094, 2966, 1467, 1423, 1402, 1352, 1206, 1197, 1117, 1089, 1017, 970, 871, 843, 781, 753, 743, 732, 720 cm^−1^; ^1^H -NMR (400 MHz, CDCl_3_): δ 0.66 (t, *J* = 7.6 Hz, 3H, CH_3_), 1.48 (sext, *J* = 7.6 Hz, 2H, CH_2_), 4.13 (t, *J* = 7.6 Hz, 2H, CH_2_), 7.44 (dd, *J* = 5.2 and 3.2 Hz, 2H, ArH), 7.47 (dd, *J* = 5.2 and 1.6 Hz, 2H, ArH), 7.58 (dd, *J* = 3.2 and 1.6 Hz, 2H, ArH), 7.73 (d, *J* = 8.4 Hz, 4H, ArH), 7.78 (d, *J* = 8.4 Hz, 4H, ArH); ^13^C-NMR (100 MHz, CDCl_3_): δ 10.7, 23.5, 46.6, 121.3, 126.1, 126.4, 126.7, 126.8, 129.4, 137.4, 141.2, 155.4; HRMS *m/z* calcd for (C_25_H_21_N_3_S_2_ + H^+^): 428.1250; found: 428.1259.

*3,5-Bis(biphenyl-4-yl)-4-butyl-4H-1,2,4-triazole* (**7a**). Creamy solid in 92% yield, 0.396 g, m.p. 297–300 °C; UV (CH_2_Cl_2_) λ_max_ 282.0 nm (ε⋅10^−3^ 41.9 cm^−1^M^−1^); IR (ATR) ν: 3034, 2953, 2926, 2859, 1472, 1447, 1423, 1384, 1211, 1121, 1098, 1005, 973, 842, 768, 753, 741, 723, 688 cm^−1^; ^1^H-NMR (400 MHz, CDCl_3_): δ 0.69 (t, *J* = 7.6 Hz, 3H, CH_3_), 1.07 (sext, *J* = 7.6 Hz, 2H, CH_2_), 1.45 (quin, *J* = 7.6 Hz, 2H, CH_2_), 4.19 (t, *J* = 7.6 Hz, 2H, CH_2_), 7.40 (t, *J* = 7.2 Hz, 2H, ArH), 7.49 (t, *J* = 7.2 Hz, 4H, ArH), 7.67 (d, *J* = 7.2 Hz, 4H, ArH), 7.75-7.79 (m, 8H, ArH); ^13^C-NMR (100 MHz, CDCl_3_): δ 13.2, 19.3, 32.0, 44.8, 126.6, 127.1, 127.6, 127.9, 128.9, 129.3, 140.0, 142.8, 155.4; HRMS *m/z* calcd for (C_30_H_27_N_3_ + H^+^): 430.2278; found: 430.2277.

*4-Butyl-3,5-bis(2′-methylbiphenyl-4-yl)-4H-1,2,4-triazole* (**7b**). Grey solid in 83% yield, 0.380 g, m.p. 182–183 °C; UV (CH_2_Cl_2_) λ_max_ 269.0 nm (ε⋅10^−3^ 32.6 cm^−1^M^−1^); IR (ATR) ν: 3051, 3015, 2963, 2926, 2859, 1470, 1460, 1421, 1383, 1355, 1326, 1109, 1008, 970, 863, 854, 840, 807, 766, 745, 734, 724 cm^−1^; ^1^H-NMR (400 MHz, CDCl_3_): δ 0.70 (t, *J* = 7.2 Hz, 3H, CH_3_), 1.10 (sext, *J* = 7.2 Hz, 2H, CH_2_), 1.48 (quin, *J* = 7.2 Hz, 2H, CH_2_), 2.32 (s, 6H, 2 × CH_3_), 4.21 (t, *J* = 7.2 Hz, 2H, CH_2_), 7.28–7.31 (m, 8H, ArH), 7.50 (d, *J* = 8.4 Hz, 4H, ArH), 7.74 (d, *J* = 8.4 Hz, 4H, ArH); ^13^C-NMR (100 MHz, CDCl_3_): δ 13.2, 19.3, 20.4, 32.1, 44.7, 125.9, 126.3, 127.7, 128.7, 129.6, 129.8, 130.5, 135.3, 140.9, 143.8, 155.5; HRMS *m/z* calcd for (C_32_H_31_N_3_ + H^+^): 458.2591; found: 458.2588.

*4-Butyl-3,5-bis(3′-methylbiphenyl-4-yl)-4H-1,2,4-triazole* (**7c**). White solid in 96% yield, 0.440 g, m.p. 211–213 °C; UV (CH_2_Cl_2_) λ_max_ 285.0 nm (ε⋅10^−3^ 44.9 cm^−1^M^−1^); IR (ATR) ν: 3020, 2960, 2872, 1607, 1466, 1416, 1340, 1262, 1111, 1094, 1039, 1019, 972, 862, 855, 845, 780, 761, 749, 608 cm^−1^; ^1^H-NMR (400 MHz, CDCl_3_): δ 0.69 (t, *J* = 7.2 Hz, 3H, CH_3_), 1.06 (sext, *J* = 7.2 Hz, 2H, CH_2_), 1.45 (quin, *J* = 7.2 Hz, 2H, CH_2_), 2.45 (s, 6H, 2 × CH_3_), 4.18 (t, *J* = 7.2 Hz, 2H, CH_2_), 7.22 (d, *J* = 7.6 Hz, 2H, ArH), 7.38 (t, *J* = 7.6 Hz, 2H, ArH), 7.45–7.49 (m, 4H, ArH), 7.75–7.79 (m, 8H, ArH); ^13^C-NMR (100 MHz, CDCl_3_): δ 13.2, 19.3, 21.5, 32.1, 44.8, 124.3, 126.6, 127.6, 127.9, 128.7, 128.9, 129.3, 138.6, 140.0, 142.9, 155.4; HRMS *m/z* calcd for (C_32_H_31_N_3_ + H^+^): 458.2591; found: 458.2592.

*4-Butyl-3,5-bis(2′,6′-dimethylbiphenyl-4-yl)-4H-1,2,4-triazole* (**7d**). White solid in 79% yield, 0.384 g, m.p. 269–271 °C; UV (CH_2_Cl_2_) λ_max_ 258.0 nm (ε⋅10^-3^ 31.1 cm^−1^M^−1^); IR (ATR) ν: 3035, 2969, 1464, 851, 839, 778, 751 cm^−1^; ^1^H-NMR (400 MHz, CDCl_3_): δ 0.66 (t, *J* = 7.2 Hz, 3H, CH_3_), 1.07 (sext, *J* = 7.2 Hz, 2H, CH_2_), 1.47 (quin, *J* = 7.2 Hz, 2H, CH_2_), 2.07 (s, 12H, 4 × CH_3_), 4.23 (t, *J* = 7.2 Hz, 2H, CH_2_), 7.15 (d, *J* = 7.2 Hz, 4H, ArH), 7.19–7.23 (m, 2H, ArH), 7.34 (d, *J* = 8.4 Hz, 4H, ArH), 7.76 (d, *J* = 8.4 Hz, 4H, ArH); ^13^C-NMR (100 MHz, CDCl_3_): δ 13.0, 19.1, 20.8, 31.9, 44.5, 126.3, 127.4, 129.1, 129.8, 135.8, 140.8, 143.1, 155.5; HRMS *m/z* calcd for (C_34_H_35_N_3_ + H^+^): 486.2904; found: 486.2901.

*4-Butyl-3,5-bis(2′-methoxybiphenyl-4-yl)-4H-1,2,4-triazole* (**7e**). White solid in 85% yield, 0.417 g, m.p. 190–193 °C; UV (CH_2_Cl_2_) λ_max_ 272.0 nm (ε⋅10^−3^ 29.5 cm^−1^M^−1^) and 295.0 (28.3); IR (ATR) ν: 2956, 2932, 2832, 1597, 1582, 1493, 1462, 1423, 1258, 1239, 1122, 1056, 1025, 1005, 974, 866, 837, 801, 756, 736 cm^−1^; ^1^H-NMR (400 MHz, CDCl_3_): δ 0.71 (t, *J* = 7.6 Hz, 3H, CH_3_), 1.10 (sext, *J* = 7.6 Hz, 2H, CH_2_), 1.49 (quin, *J* = 7.6 Hz, 2H, CH_2_), 3.85 (s, 6H, 2 × OCH_3_), 4.20 (t, *J* = 7.6 Hz, 2H, CH_2_), 7.02 (d, *J* = 7.6 Hz, 2H, ArH), 7.07 (t, *J* = 7.6 Hz, 2H, ArH), 7.35–7.40 (m, 4H, ArH), 7.70 (d, *J* = 8.4 Hz, 4H, ArH), 7.73 (d, *J* = 8.4 Hz, 4H, ArH); ^13^C-NMR (100 MHz, CDCl_3_): δ 13.2, 19.4, 32.0, 44.8, 55.6, 111.5, 121.0, 126.3, 128.5, 129.2, 129.7, 130.0, 130.8, 140.3, 155.5, 156.5; HRMS *m/z* calcd for (C_32_H_31_N_3_O_2_ + H^+^): 490.2489; found: 490.2491.

*4-Butyl-3,5-bis(3′-methoxybiphenyl-4-yl)-4H-1,2,4-triazole* (**7f**). Grey solid in 82% yield, 0.402 g, m.p. 182–184 °C; UV (CH_2_Cl_2_) λ_max_ 284.0 nm (ε⋅10^-3^ 33.5 cm^−1^M^−1^); IR (ATR) ν: 2954, 2930, 2830, 1608, 1583, 1560, 1474, 1421, 1297, 1212, 1172, 1055, 1033, 1015, 856, 839, 779, 752, 693 cm^−1^; ^1^H-NMR (400 MHz, CDCl_3_): δ 0.69 (t, *J* = 7.6 Hz, 3H, CH_3_), 1.07 (sext, *J* = 7.6 Hz, 2H, CH_2_), 1.45 (quin, *J* = 7.6 Hz, 2H, CH_2_), 3.90 (s, 6H, 2 × OCH_3_), 4.19 (t, *J* = 7.6 Hz, 2H, CH_2_), 6.95 (dd, *J* = 8.0 and 2.4 Hz, 2H, ArH), 7.19 (t, *J* = 2.4 Hz, 2H, ArH), 7.24–7.26 (m, 2H, ArH), 7.40 (t, *J* = 8.0 Hz, 2H, ArH), 7.74–7.78 (m, 8H, ArH); ^13^C-NMR (100 MHz, CDCl_3_): δ 13.2, 19.3, 32.1, 44.8, 55.4, 112.9, 113.3, 119.6, 126.8, 127.6, 129.3, 130.0, 141.5, 142.6, 155.4, 160.1; HRMS *m/z* calcd for (C_32_H_31_N_3_O_2_ + H^+^): 490.2489; found: 490.2492.

*4-Butyl-3,5-bis(3′-nitrobiphenyl-4-yl)-4H-1,2,4-triazole* (**7g**). Creamy solid in 91% yield, 0.473 g, m.p. 252–253 °C; UV (CH_2_Cl_2_) λ_max_ 276.0 nm (ε⋅10^−3^ 46.9 cm^−1^M^−1^); IR (ATR) ν: 3099, 2926, 2861, 1522, 1490, 1467, 1347, 1292, 1102, 1018, 973, 876, 850, 832, 799, 780, 740, 724, 704 cm^−1^; ^1^H-NMR (400 MHz, CDCl_3_): δ 0.71 (t, *J* = 7.6 Hz, 3H, CH_3_), 1.09 (sext, *J* = 7.6 Hz, 2H, CH_2_), 1.47 (quin, *J* = 7.6 Hz, 2H, CH_2_), 4.22 (t, *J* = 7.6 Hz, 2H, CH_2_), 7.68 (t, *J* = 8.0 Hz, 2H, ArH), 7.82 (d, *J* = 8.4 Hz, 4H, ArH), 7.86 (d, *J* = 8.4 Hz, 4H, ArH), 8.00 (dd, *J* = 8.0 and 2.0 Hz, 2H, ArH), 8.27 (dd, *J* = 8.0 and 2.0 Hz, 2H, ArH), 8.54 (t, *J* = 2.0 Hz, 2H, ArH); ^13^C-NMR (100 MHz, CDCl_3_): δ 13.2, 19.3, 32.1, 45.0, 122.0, 122.7, 127.7, 128.0, 129.7, 130.0, 133.0, 140.3, 141.7, 148.9, 155.1; HRMS *m/z* calcd for (C_30_H_25_N_5_O_4_ + H^+^): 520.1979; found: 520.1967.

*3,5-Bis(3′-aminobiphenyl-4-yl)-4-butyl-4H-1,2,4-triazole* (**7h**). Beige solid in 98% yield, 0.451 g, m.p. 244–246 °C; UV (CH_2_Cl_2_) λ_max_ 232.0 nm (ε⋅10^−3^ 27.7 cm^−1^M^−1^), 258.0 (29.6) and 281.0 (34.1); IR (ATR) ν: 3428, 3348, 2955, 2926, 2871, 1619, 1601, 1588, 1472, 1449, 1422, 1318, 1229, 1172, 973, 836, 777, 755, 744, 665 cm^−1^; ^1^H-NMR (400 MHz, CDCl_3_): δ 0.68 (t, *J* = 7.2 Hz, 3H, CH_3_), 1.05 (sext, *J* = 7.2 Hz, 2H, CH_2_), 1.43 (quin, *J* = 7.2 Hz, 2H, CH_2_), 3.81 (br.s, 4H, 2 × NH_2_), 4.17 (t, *J* = 7.2 Hz, 2H, CH_2_), 6.72 (dd, *J* = 8.0 and 2.0 Hz, 2H, ArH), 6.98 (t, *J* = 2.0 Hz, 2H, ArH), 7.05 (dd, *J* = 8.0 and 2.0 Hz, 2H, ArH), 7.26 (t, *J* = 8.0 Hz, 2H, ArH), 7.71–7.75 (m, 8H, ArH); ^13^C-NMR (100 MHz, CDCl_3_): δ 13.2, 19.3, 32.0, 44.8, 113.7, 114.7, 117.5, 126.6, 127.5, 129.2, 129.9, 141.2, 143.0, 147.0, 155.4; HRMS *m/z* calcd for (C_30_H_29_N_5_ + H^+^): 460.2496; found: 460.2479.

*4-Butyl-3,5-bis[4-(pyridin-4-yl)phenyl]-4H-1,2,4-triazole* (**7i**). Creamy solid in 92% yield, 0.397 g, m.p. 258–259 °C; UV (CH_2_Cl_2_) λ_max_ 281.0 nm (ε⋅10^−3^ 33.7 cm^−1^M^−1^); IR (ATR) ν: 2963, 1595, 1540, 1471, 1407, 1217, 971, 857, 811, 771, 757, 736 cm^−1^; ^1^H-NMR (400 MHz, CDCl_3_): δ 0.70 (t, *J* = 7.6 Hz, 3H, CH_3_), 1.07 (sext, *J* = 7.6 Hz, 2H, CH_2_), 1.45 (quin, *J* = 7.6 Hz, 2H, CH_2_), 4.21 (t, *J* = 7.6 Hz, 2H, CH_2_), 7.58 (d, *J* = 6.0 Hz, 4H, ArH), 7.81–7.85 (m, 8H, ArH), 8.73 (d, *J* = 6.0 Hz, 4H, ArH); ^13^C-NMR (100 MHz, CDCl_3_): δ 13.2, 19.3, 32.1, 44.9, 121.6, 127.6, 128.4, 129.6, 139.8, 147.1, 150.5, 155.1; HRMS *m/z* calcd for (C_28_H_25_N_5_ + H^+^): 432.2183; found: 432.2180.

*4-Butyl-3,5-bis[4-(pyridin-3-yl)phenyl]-4H-1,2,4-triazole* (**7j**). White solid in 74% yield, 0.320 g, m.p. 207–209 °C; UV (CH_2_Cl_2_) λ_max_ 285.0 nm (ε⋅10^−3^ 40.4 cm^−1^M^−1^); IR (ATR) ν: 3033, 2929, 2870, 1575, 1468, 1428, 1413, 1386, 1024, 1000, 972, 853, 803, 774, 746, 710 cm^−1^; ^1^H-NMR (400 MHz, CDCl_3_): δ 0.70 (t, *J* = 7.2 Hz, 3H, CH_3_), 1.08 (sext, *J* = 7.2 Hz, 2H, CH_2_), 1.46 (quin, *J* = 7.2 Hz, 2H, CH_2_), 4.21 (t, *J* = 7.2 Hz, 2H, CH_2_), 7.42 (dd, *J* = 7.6 and 4.8 Hz, 2H, ArH), 7.78 (d, *J* = 8.4 Hz, 4H, ArH), 7.84 (d, *J* = 8.4 Hz, 4H, ArH), 7.95 (dt, *J* = 7.6 and 1.6 Hz, 2H, ArH), 8.66 (dd, *J* = 4.8 and 1.6 Hz, 2H, ArH), 8.93 (d, *J* = 1.6 Hz, 2H, ArH); ^13^C-NMR (100 MHz, CDCl_3_): δ 13.2, 19.3, 32.1, 44.9, 123.7, 127.5, 127.6, 129.6, 134.4, 135.5, 139.5, 148.3, 149.1, 155.2; HRMS *m/z* calcd for (C_28_H_25_N_5_ + H^+^): 432.2183; found: 432.2181.

*4-Butyl-3,5-bis[4-(furan-2-yl)phenyl]-4H-1,2,4-triazole* (**7k**). Creamy solid in 75% yield, 0.307 g, m.p. 264–267 °C; UV (CH_2_Cl_2_) λ_max_ 310.0 nm (ε⋅10^-3^ 43.6 cm^−1^M^−1^); IR (ATR) ν: 2956, 2926, 2871, 1615, 1491, 1471, 1220, 1189, 1157, 1115, 1008, 972, 904, 884, 848, 805, 773, 735, 702 cm^−1^; ^1^H-NMR (400 MHz, CDCl_3_): δ 0.66 (t, *J* = 7.6 Hz, 3H, CH_3_), 1.03 (sext, *J* = 7.6 Hz, 2H, CH_2_), 1.40 (quin, *J* = 7.6 Hz, 2H, CH_2_), 4.14 (t, *J* = 7.6 Hz, 2H, CH_2_), 6.53 (dd, *J* = 3.2 and 1.6 Hz, 2H, ArH), 6.78 (d, *J* = 3.2 Hz, 2H, ArH), 7.53 (d, *J* = 1.6 Hz, 2H, ArH), 7.71 (d, *J* = 8.4 Hz, 4H, ArH), 7.83 (d, *J* = 8.4 Hz, 4H, ArH); ^13^C-NMR (100 MHz, CDCl_3_): δ 13.2, 19.3, 32.0, 44.8, 106.4, 111.9, 124.1, 126.4, 129.2, 132.3, 142.8, 153.0, 155.4; HRMS *m/z* calcd for (C_26_H_23_N_3_O_2_ + H^+^): 410.1863; found: 410.1866.

*4-Butyl-3,5-bis[4-(furan-3-yl)phenyl]-4H-1,2,4-triazole* (**7l**). Yellow solid in 94% yield, 0.384 g, m.p. 274–277 °C; UV (CH_2_Cl_2_) λ_max_ 285.0 nm (ε⋅10^−3^ 37.3 cm^−1^M^−1^); IR (ATR) ν: 3134, 2960, 1507, 1469, 1194, 1162, 1115, 1094, 1054, 1015, 975, 923, 873, 842, 785, 749 cm^−1^; ^1^H-NMR (400 MHz, CDCl_3_): δ 0.67 (t, *J* = 7.6 Hz, 3H, CH_3_), 1.04 (sext, *J* = 7.6 Hz, 2H, CH_2_), 1.41 (quin, *J* = 7.6 Hz, 2H, CH_2_), 4.14 (t, *J* = 7.6 Hz, 2H, CH_2_), 6.77 (d, *J* = 1.6 Hz, 2H, ArH), 7.53 (d, *J* = 1.6 Hz, 2H, ArH), 7.65 (d, *J* = 8.8 Hz, 4H, ArH), 7.71 (d, *J* = 8.8 Hz, 4H, ArH), 7.83 (s, 2H, ArH); ^13^C-NMR (100 MHz, CDCl_3_): δ 13.2, 19.3, 32.0, 44.8, 108.6, 125.7, 126.2, 126.3, 129.4, 134.2, 139.2, 144.0, 155.4; HRMS *m/z* calcd for (C_26_H_23_N_3_O_2_ + H^+^): 410.1863; found: 410.1867.

*4-Butyl-3,5-bis[4-(thiophen-2-yl)phenyl]-4H-1,2,4-triazole* (**7m**). Grey solid in 49% yield, 0.216 g, m.p. 289–292 °C; UV (CH_2_Cl_2_) λ_max_ 312.0 nm (ε⋅10^−3^ 37.4 cm^−1^M^−1^); IR (ATR) ν: 2955, 1472, 1426, 1400, 1215, 1193, 1115, 1099, 974, 844, 819, 742, 695 cm^−1^; ^1^H-NMR (400 MHz, CDCl_3_): δ 0.68 (t, *J* = 7.6 Hz, 3H, CH_3_), 1.05 (sext, *J* = 7.6 Hz, 2H, CH_2_), 1.43 (quin, *J* = 7.6 Hz, 2H, CH_2_), 4.15 (t, *J* = 7.6 Hz, 2H, CH_2_), 7.13 (dd, *J* = 4.8 and 3.6 Hz, 2H, ArH), 7.36 (d, *J* = 4.8 Hz, 2H, ArH), 7.43 (d, *J* = 3.6 Hz, 2H, ArH), 7.70 (d, *J* = 8.4 Hz, 4H, ArH), 7.78 (d, *J* = 8.4 Hz, 4H, ArH); ^13^C-NMR (100 MHz, CDCl_3_): δ 13.2, 19.3, 32.0, 44.9, 124.0, 125.8, 126.2, 126.6, 128.3, 129.4, 136.0, 143.2, 155.3; HRMS *m/z* calcd for (C_26_H_23_N_3_S_2_ + H^+^): 442.1406; found: 442.1412.

*4-Butyl-3,5-bis[4-(thiophen-3-yl)phenyl]-4H-1,2,4-triazole* (**7n**). Beige solid in 77% yield, 0.342 g, m.p. 288–290 °C; UV (CH_2_Cl_2_) λ_max_ 289.0 nm (ε⋅10^−3^ 37.4 cm^−1^M^−1^); IR (ATR) ν: 3067, 2955, 2872, 1470, 1428, 1363, 1196, 1094, 1014, 973, 867, 841, 782, 746, 688 cm^−1^; ^1^H-NMR (400 MHz, CDCl_3_): δ 0.68 (t, *J* = 7.6 Hz, 3H, CH_3_), 1.04 (sext, *J* = 7.6 Hz, 2H, CH_2_), 1.41 (quin, *J* = 7.6 Hz, 2H, CH_2_), 4.13 (t, *J* = 7.6 Hz, 2H, CH_2_), 7.43–7.47 (m, 2H, ArH), 7.56–7,59 (m, 2H, ArH), 7.68–7.78 (m, 10H, ArH); ^13^C-NMR (100 MHz, CDCl_3_): δ 13.2, 19.3, 32.0, 44.8, 121.4, 126.1, 126.2, 126.7, 126.8, 129.4, 132.3, 141.2, 154.6; HRMS *m/z* calcd for (C_26_H_23_N_3_S_2_ + H^+^): 442.1406; found: 442.1397.

*3,5-Bis(biphenyl-4-yl)-4-hexyl-4H-1,2,4-triazole* (**8a**). White solid in 92% yield, 0.421 g, m.p. 240–242 °C; UV (CH_2_Cl_2_) λ_max_ 282.0 nm (ε⋅10^-3^ 45.4 cm^−1^M^−1^); IR (ATR) ν: 3028, 2926, 2855, 1474, 1444, 1429, 1379, 1072, 1007, 967, 858, 847, 835, 767, 738, 695 cm^−1^; ^1^H-NMR (400 MHz, CDCl_3_): δ 0.73 (t, *J* = 7.6 Hz, 3H, CH_3_), 1.00–1.11 (m, 6H, 3 × CH_2_), 1.46 (quin, *J* = 7.6 Hz, 2H, CH_2_), 4.18 (t, *J* = 7.6 Hz, 2H, CH_2_), 7.40 (t, *J* = 7.2 Hz, 2H, ArH), 7.49 (t, *J* = 7.2 Hz, 4H, ArH), 7.67 (d, *J* = 7.2 Hz, 4H, ArH), 7.75–7.79 (m, 8H, ArH); ^13^C-NMR (100 MHz, CDCl_3_): δ 13.8, 22.2, 25.7, 29.9, 30.7, 45.0, 126.7, 127.1, 127.6, 127.9, 128.9, 129.3, 140.1, 142.8, 155.4; HRMS *m/z* calcd for (C_32_H_31_N_3_ + H^+^): 458.2591; found: 458.2588.

*4-Hexyl-3,5-bis(2′-methylbiphenyl-4-yl)-4H-1,2,4-triazole* (**8b**). White solid in 72% yield, 0.350 g, m.p. 185–186 °C; UV (CH_2_Cl_2_) λ_max_ 269.0 nm (ε⋅10^−3^ 31.5 cm^−1^M^−1^); IR (ATR) ν: 3022, 2955, 2929, 2857, 1470, 1425, 1378, 1261, 1099, 1007, 966, 840, 766, 743, 723 cm^−1^; ^1^H-NMR (400 MHz, CDCl_3_): δ 0.75 (t, *J* = 7.2 Hz, 3H, CH_3_), 1.01–1.13 (m, 6H, 3 × CH_2_), 1.49 (quin, *J* = 7.2 Hz, 2H, CH_2_), 2.32 (s, 6H, 2 × CH_3_), 4.20 (t, *J* = 7.6 Hz, 2H, CH_2_), 7.27–7.32 (m, 8H, ArH), 7.50 (d, *J* = 8.4 Hz, 4H, ArH), 7.75 (d, *J* = 8.4 Hz, 4H, ArH); ^13^C-NMR (100 MHz, CDCl_3_): δ 13.8, 20.4, 22.1, 25.6, 29.9, 30.7, 44.9, 125.9, 126.3, 127.7, 128.7, 129.6, 129.8, 130.5, 135.3, 140.9, 143.8, 155.5; HRMS *m/z* calcd for (C_34_H_35_N_3_ + H^+^): 486.2904; found: 486.2905.

*4-Hexyl-3,5-bis(3′-methylbiphenyl-4-yl)-4H-1,2,4-triazole* (**8c**). White solid in 91% yield, 0.442 g, m.p. 217–218 °C; UV (CH_2_Cl_2_) λ_max_ 284.0 nm (ε⋅10^−3^ 40.3 cm^−1^M^−1^); IR (ATR) ν: 3017, 2929, 2863, 1607, 1468, 1416, 1380, 1338, 1112, 1017, 968, 855, 843, 754, 691 cm^−1^; ^1^H-NMR (400 MHz, CDCl_3_): δ 0.73 (t, *J* = 7.2 Hz, 3H, CH_3_), 1.01–1.11 (m, 6H, 3 × CH_2_), 1.45 (quin, *J* = 7.2 Hz, 2H, CH_2_), 2.45 (s, 6H, 2 × CH_3_), 4.17 (t, *J* = 7.6 Hz, 2H, CH_2_), 7.22 (d, *J* = 7.6 Hz, 2H, ArH), 7.37 (t, *J* = 7.6 Hz, 2H, ArH), 7.45–7.48 (m, 4H, ArH), 7.74–7.77 (m, 8H, ArH); ^13^C-NMR (100 MHz, CDCl_3_): δ 13.8, 21.5, 22.2, 25.7, 29.9, 30.7, 45.0, 124.2, 126.6, 127.6, 127.9, 128.7, 128.8, 129.2, 138.6, 140.1, 142.9, 155.4; HRMS *m/z* calcd for (C_34_H_35_N_3_ + H^+^): 486.2904; found: 486.2902.

*4-Hexyl-3,5-bis(2′,6′-dimethylbiphenyl-4-yl)-4H-1,2,4-triazole* (**8d**). White solid in 93% yield, 0.478 g, m.p. 200–202 °C; UV (CH_2_Cl_2_) λ_max_ 259.0 nm (ε⋅10^−3^ 26.8 cm^−1^M^−1^); IR (ATR) ν: 3014, 2954, 2930, 2855, 1464, 1418, 1380, 1260, 1096, 1005, 969, 852, 839, 770, 737 cm^−1^; ^1^H-NMR (400 MHz, CDCl_3_): δ 0.75 (t, *J* = 7.2 Hz, 3H, CH_3_), 1.00–1.13 (m, 6H, 3 × CH_2_), 1.48 (quin, *J* = 7.2 Hz, 2H, CH_2_), 2.07 (s, 12H, 4 × CH_3_), 4.22 (t, *J* = 7.6 Hz, 2H, CH_2_), 7.14 (d, *J* = 7.2 Hz, 4H, ArH), 7.19–7.23 (m, 2H, ArH), 7.34 (d, *J* = 8.4 Hz, 4H, ArH), 7.77 (d, *J* = 8.4 Hz, 4H, ArH); ^13^C-NMR (100 MHz, CDCl_3_): δ 13.7, 20.8, 22.1, 25.6, 29.7, 30.6, 44.9, 115.6, 126.3, 127.4, 129.1, 129.8, 135.8, 140.8, 143.1, 155.6; HRMS *m/z* calcd for (C_36_H_39_N_3_ + H^+^): 514.3217; found: 514.3217.

*4-Hexyl-3,5-bis(2′-methoxybiphenyl-4-yl)-4H-1,2,4-triazole* (**8e**). Creamy solid in 88% yield, 0.456 g, m.p. 171–172 °C; UV (CH_2_Cl_2_) λ_max_ 273.0 nm (ε⋅10^−3^ 28.1 cm^−1^M^−1^) and 296.0 (28.8); IR (ATR) ν: 3049, 2953, 2860, 1599, 1494, 1467, 1433, 1421, 1257, 1235, 1179, 1125, 1112, 1029, 1016, 1005, 861, 841, 801, 748, 737, 721 cm^−1^; ^1^H-NMR (400 MHz, CDCl_3_): δ 0.76 (t, *J* = 7.2 Hz, 3H, CH_3_), 1.02–1.13 (m, 6H, 3 × CH_2_), 1.50 (quin, *J* = 7.2 Hz, 2H, CH_2_), 3.85 (s, 6H, 2 × OCH_3_), 4.20 (t, *J* = 7.6 Hz, 2H, CH_2_), 7.03 (d, *J* = 8.4 Hz, 2H, ArH), 7.07 (t, *J* = 7.6 Hz, 2H, ArH), 7.34–7.40 (m, 4H, ArH), 7.70 (d, *J* = 8.4 Hz, 4H, ArH), 7.73 (d, *J* = 8.4 Hz, 4H, ArH); ^13^C-NMR (100 MHz, CDCl_3_): δ 13.8, 22.2, 25.7, 30.0, 30.7, 44.9, 55.6, 111.5, 121.0, 126.3, 128.5, 129.2, 129.7, 130.0, 130.8, 140.3, 155.5, 156.5; HRMS *m/z* calcd for (C_34_H_35_N_3_O_2_ + H^+^): 518.2802; found: 518.2800.

*4-Hexyl-3,5-bis(3′-methoxybiphenyl-4-yl)-4H-1,2,4-triazole* (**8f**). White solid in 83% yield, 0.430 g, m.p. 182–184 °C; UV (CH_2_Cl_2_) λ_max_ 284.0 nm (ε⋅10^−3^ 33.6 cm^−1^M^−1^); IR (ATR) ν: 3036, 2954, 2931, 2853, 1599, 1590, 1476, 1461, 1428, 1414, 1323, 1300, 1221, 1177, 1049, 1035, 1022, 861, 834, 788, 747 cm^−1^; ^1^H-NMR (400 MHz, CDCl_3_): δ 0.73 (t, *J* = 7.2 Hz, 3H, CH_3_), 1.01–1.10 (m, 6H, 3 × CH_2_), 1.46 (quin, *J* = 7.2 Hz, 2H, CH_2_), 3.90 (s, 6H, 2 × OCH_3_), 4.18 (t, *J* = 7.2 Hz, 2H, CH_2_), 6.95 (dd, *J* = 8.0 and 2.4 Hz, 2H, ArH), 7.19 (d, *J* = 2.4 Hz, 2H, ArH), 7.23–7.27 (m, 2H, ArH), 7.40 (t, *J* = 8.0 Hz, 2H, ArH), 7.74–7.78 (m, 8H, ArH); ^13^C-NMR (100 MHz, CDCl_3_): δ 13.8, 22.2, 25.7, 29.9, 30.7, 45.0, 55.4, 113.0, 113.3, 119.6, 126.9, 127.6, 129.3, 130.0, 141.6, 142.6, 155.4, 160.1; HRMS *m/z* calcd for (C_34_H_35_N_3_O_2_ + H^+^): 518.2802; found: 518.2801.

*4-Hexyl-3,5-bis(3′-nitrobiphenyl-4-yl)-4H-1,2,4-triazole* (**8g**). Creamy solid in 85% yield, 0.466 g, m.p. 223–225 °C; UV (CH_2_Cl_2_) λ_max_ 276.0 nm (ε⋅10^-3^ 48.3 cm^−1^M^−1^); IR (ATR) ν: 3086, 2927, 2857, 1522, 1472, 1344, 1294, 1105, 1085, 968, 877, 843, 813, 778, 730, 702, 684 cm^−1^; ^1^H-NMR (400 MHz, CDCl_3_): δ 0.74 (t, *J* = 7.2 Hz, 3H, CH_3_), 1.01–1.13 (m, 6H, 3 × CH_2_), 1.47 (quin, *J* = 7.6 Hz, 2H, CH_2_), 4.21 (t, *J* = 7.6 Hz, 2H, CH_2_), 7.68 (t, *J* = 7.6 Hz, 2H, ArH), 7.82 (d, *J* = 8.4 Hz, 4H, ArH), 7.86 (d, *J* = 8.4 Hz, 4H, ArH), 8.00 (dd, *J* = 7.6 and 2.0 Hz, 2H, ArH), 8.27 (dd, *J* = 7.6 and 2.0 Hz, 2H, ArH), 8.53 (t, *J* = 2.0 Hz, 2H, ArH); ^13^C-NMR (100 MHz, CDCl_3_): δ 13.8, 22.2, 25.7, 30.0, 30.7, 45.1, 122.0, 122.7, 127.7, 128.0, 129.7, 130.0, 133.0, 140.3, 141.7, 148.8, 155.1; HRMS *m/z* calcd for (C_32_H_29_N_5_O_4_ + H^+^): 548.2292; found: 548.2299.

*3,5-Bis(3′-aminobiphenyl-4-yl)-4-hexyl-4H-1,2,4-triazole* (**8h**). Beige solid in 78% yield, 0.381 g, m.p. 181–183 °C; UV (CH_2_Cl_2_) λ_max_ 233.0 nm (ε⋅10^−3^ 30.3 cm^−1^M^−1^), 258.0 (32.8) and 281.0 (37.7); IR (ATR) ν: 3442, 3369, 3200, 3034, 2956, 2926, 2856, 1617, 1601, 1586, 1562, 1474, 1427, 1321, 1227, 1166, 993, 888, 835, 780, 746, 722 cm^−1^; ^1^H-NMR (400 MHz, CDCl_3_): δ 0.73 (t, *J* = 7.2 Hz, 3H, CH_3_), 0.98–1.10 (m, 6H, 3 × CH_2_), 1.44 (quin, *J* = 7.2 Hz, 2H, CH_2_), 3.78 (br.s, 4H, 2 × NH_2_), 4.16 (t, *J* = 7.6 Hz, 2H, CH_2_), 6.73 (dd, *J* = 7.6 and 2.0 Hz, 2H, ArH), 6.98 (t, *J* = 2.0 Hz, 2H, ArH), 7.05 (dd, *J* = 7.6 and 2.0 Hz, 2H, ArH), 7.26 (t, *J* = 7.6 Hz, 2H, ArH), 7.72–7.74 (m, 8H, ArH); ^13^C-NMR (100 MHz, CDCl_3_): δ 13.8, 22.2, 25.6, 29.9, 30.7, 45.0, 113.7, 114.7, 117.5, 126.6, 127.5, 129.2, 129.9, 141.2, 143.0, 146.9, 155.4; HRMS *m/z* calcd for (C_32_H_33_N_5_ + H^+^): 488.2809; found: 488.2817.

*4-Hexyl-3,5-bis[4-(pyridin-4-yl)phenyl]-4H-1,2,4-triazole* (**8i**). White solid in 86% yield, 0.396 g, m.p. 205–207 °C; UV (CH_2_Cl_2_) λ_max_ 281.0 nm (ε⋅10^−3^ 46.6 cm^−1^M^−1^); IR (ATR) ν: 2924, 2855, 1595, 1540, 1471, 1408, 1216, 993, 967, 857, 812, 771, 753, 737, 667 cm^−1^; ^1^H-NMR (400 MHz, CDCl_3_): δ 0.73 (t, *J* = 7.2 Hz, 3H, CH_3_), 0.99-1.11 (m, 6H, 3 × CH_2_), 1.46 (quin, *J* = 7.2 Hz, 2H, CH_2_), 4.20 (t, *J* = 7.2 Hz, 2H, CH_2_), 7.58 (d, *J* = 6.0 Hz, 4H, ArH), 7.82–7.85 (m, 8H, ArH), 8.73 (d, *J* = 6.0 Hz, 4H, ArH); ^13^C-NMR (100 MHz, CDCl_3_): δ 13.8, 22.1, 25.6, 29.9, 30.7, 45.1, 121.6, 127.6, 128.4, 129.6, 139.8, 147.1, 150.5, 155.1; HRMS *m/z* calcd for (C_30_H_29_N_5_ + H^+^): 460.2496; found: 460.2499.

*4-Hexyl-3,5-bis[4-(pyridin-3-yl)phenyl]-4H-1,2,4-triazole* (**8j**). Creamy solid in 83% yield, 0.382 g, m.p. 198–199 °C; UV (CH_2_Cl_2_) λ_max_ 284.0 nm (ε⋅10^−3^ 41.6 cm^−1^M^−1^); IR (ATR) ν: 2927, 2855, 1574, 1469, 1415, 1380, 1102, 1026, 1001, 967, 849, 839, 804, 751, 709 cm^−1^; ^1^H-NMR (400 MHz, CDCl_3_): δ 0.73 (t, *J* = 7.2 Hz, 3H, CH_3_), 1.00–1.11 (m, 6H, 3 × CH_2_), 1.47 (quin, *J* = 7.2 Hz, 2H, CH_2_), 4.20 (t, *J* = 7.2 Hz, 2H, CH_2_), 7.42 (dd, *J* = 8.0 and 4.8 Hz, 2H, ArH), 7.78 (d, *J* = 8.4 Hz, 4H, ArH), 7.83 (d, *J* = 8.4 Hz, 4H, ArH), 7.96 (dt, *J* = 8.0 and 1.6 Hz, 2H, ArH), 8.66 (dd, *J* = 4.8 and 1.6 Hz, 2H, ArH), 8.94 (d, *J* = 1.6 Hz, 2H, ArH); ^13^C-NMR (100 MHz, CDCl_3_): δ 13.8, 22.2, 25.7, 29.9, 30.7, 45.1, 123.7, 127.5, 127.7, 129.6, 134.4, 135.5, 139.5, 148.3, 149.1, 155.2; HRMS *m/z* calcd for (C_30_H_29_N_5_ + H^+^): 460.2496; found: 460.2497.

*3,5-Bis[4-(furan-2-yl)phenyl]-4-hexyl-4H-1,2,4-triazole* (**8k**). Creamy solid in 89% yield, 0.390 g, m.p. 199–202 °C; UV (CH_2_Cl_2_) λ_max_ 310.0 nm (ε⋅10^−3^ 37.8 cm^−1^M^−1^); IR (ATR) ν: 2933, 1687, 1611, 1473, 1374, 1259, 1180, 1092, 1009, 903, 844, 800, 731, 664 cm^−1^; ^1^H-NMR (400 MHz, CDCl_3_): δ 0.72 (t, *J* = 7.2 Hz, 3H, CH_3_), 0.97–1.10 (m, 6H, 3 × CH_2_), 1.41 (quin, *J* = 7.2 Hz, 2H, CH_2_), 4.13 (t, *J* = 7.6 Hz, 2H, CH_2_), 6.53 (dd, *J* = 3.6 and 2.0 Hz, 2H, ArH), 6.78 (d, *J* = 3.6 Hz, 2H, ArH), 7.53 (d, *J* = 2.0 Hz, 2H, ArH), 7.70 (d, *J* = 8.8 Hz, 4H, ArH), 7.83 (d, *J* = 8.8 Hz, 4H, ArH); ^13^C-NMR (100 MHz, CDCl_3_): δ 13.7, 22.2, 25.7, 29.8, 30.7, 45.0, 106.4, 111.9, 124.1, 126.3, 129.2, 132.3, 142.8, 153.0, 155.3; HRMS *m/z* calcd for (C_28_H_27_N_3_O_2_ + H^+^): 438.2176; found: 438.2175.

*3,5-Bis[4-(furan-3-yl)phenyl]-4-hexyl-4H-1,2,4-triazole* (**8l**). Yellow solid in 69% yield, 0.302 g, m.p. 175–178 °C; UV (CH_2_Cl_2_) λ_max_ 284.0 nm (ε⋅10^−3^ 17.8 cm^−1^M^−1^); IR (ATR) ν: 3093, 2933, 2864, 1740, 1615, 1475, 1429, 1261, 1164, 1116, 1055, 1015, 957, 922, 873, 839, 785, 750, 723, 695 cm^−1^; ^1^H-NMR (400 MHz, CDCl_3_): δ 0.73 (t, *J* = 7.2 Hz, 3H, CH_3_), 0.97–1.10 (m, 6H, 3 × CH_2_), 1.41 (quin, *J* = 7.2 Hz, 2H, CH_2_), 4.13 (t, *J* = 7.6 Hz, 2H, CH_2_), 6.77 (dd, *J* = 2.0 and 1.2 Hz, 2H, ArH), 7.53 (d, *J* = 2.0 Hz, 2H, ArH), 7.65 (d, *J* = 8.8 Hz, 4H, ArH), 7.69 (d, *J* = 8.8 Hz, 4H, ArH), 7.83 (d, *J* = 1.2 Hz, 2H, ArH); ^13^C-NMR (100 MHz, CDCl_3_): δ 13.8, 22.2, 25.7, 29.8, 30.7, 45.0, 108.6, 125.7, 126.1, 126.2, 129.3, 134.2, 139.2, 144.0, 155.3; HRMS *m/z* calcd for (C_28_H_27_N_3_O_2_ + H^+^): 438.2176; found: 438.2173.

*4-Hexyl-3,5-bis[4-(thiophen-2-yl)phenyl]-4H-1,2,4-triazole* (**8m**). Beige solid in 37% yield, 0.174 g, m.p. 219–220 °C; UV (CH_2_Cl_2_) λ_max_ 312.0 nm (ε⋅10^−3^ 42.0 cm^−1^M^−1^); IR (ATR) ν: 2954, 2923, 2853, 1611, 1471, 1427, 1400, 1377, 1351, 1259, 1213, 1115, 959, 848, 818, 776, 752, 662 cm^−1^; ^1^H-NMR (400 MHz, CDCl_3_): δ 0.73 (t, *J* = 7.2 Hz, 3H, CH_3_), 0.99–1.09 (m, 6H, 3 × CH_2_), 1.43 (quin, *J* = 7.2 Hz, 2H, CH_2_), 4.14 (t, *J* = 7.2 Hz, 2H, CH_2_), 7.13 (dd, *J* = 4.8 and 3.6 Hz, 2H, ArH), 7.36 (d, *J* = 4.8 Hz, 2H, ArH), 7.43 (d, *J* = 3.6 Hz, 2H, ArH), 7.71 (d, *J* = 8.4 Hz, 4H, ArH), 7.78 (d, *J* = 8.4 Hz, 4H, ArH); ^13^C-NMR (100 MHz, CDCl_3_): δ 13.8, 22.2, 25.7, 29.9, 30.7, 45.0, 124.0, 125.8, 126.2, 126.6, 128.3, 129.4, 136.0, 143.2, 155.3; HRMS *m/z* calcd for (C_28_H_27_N_3_S_2_ + H^+^): 470.1719; found: 470.1707.

*4-Hexyl-3,5-bis[4-(thiophen-3-yl)phenyl]-4H-1,2,4-triazole* (**8n**). Creamy solid in 62% yield, 0.291 g, m.p. 237–239 °C; UV (CH_2_Cl_2_) λ_max_ 290.0 nm (ε⋅10^−3^ 39.6 cm^−1^M^−1^); IR (ATR) ν: 3065, 2925, 2856, 1614, 1467, 1426, 1355, 1260, 1194, 1100, 1014, 864, 846, 781, 749 cm^−1^; ^1^H-NMR (400 MHz, CDCl_3_): δ 0.73 (t, *J* = 7.2 Hz, 3H, CH_3_), 0.98–1.09 (m, 6H, 3 × CH_2_), 1.43 (quin, *J* = 7.2 Hz, 2H, CH_2_), 4.15 (t, *J* = 7.2 Hz, 2H, CH_2_), 7.44 (dd, *J* = 5.2 and 2.8 Hz, 2H, ArH), 7.47 (dd, *J* = 5.2 and 1.6 Hz, 2H, ArH), 7.57 (dd, *J* = 2.8 and 1.6 Hz, 2H, ArH), 7.72 (d, *J* = 8.4 Hz, 4H, ArH), 7.77 (d, *J* = 8.4 Hz, 4H, ArH); ^13^C-NMR (100 MHz, CDCl_3_): δ 13.8, 22.2, 25.7, 29.9, 30.7, 45.0, 121.3, 126.1, 126.4, 126.7, 126.8, 129.4, 137.3, 141.2, 155.4; HRMS *m/z* calcd for (C_28_H_27_N_3_S_2_ + H^+^): 470.1719; found: 470.1728.

#### 3.2.4. An IL Alternative Approach for Suzuki Cross-Coupling Reaction. Synthesis of 3,5-Bis(biphenyl-4-yl)-4-butyl-4H-1,2,4-triazole (**7a**)

To a mixture of 3,5-bis(4-bromophenyl)-4-butyl-4*H*-1,2,4-triazole (3c, 0.435 g, 1.00 mmol), phenylboronic acid (4a, 0.305 g, 2.50 mmol) and Pd(PPh_3_)_4_ (0.058 g, 0.05 mmol), aq. choline hydroxide solution 46 wt.% (Choline-OH, 10 mL) and toluene (1 mL) were added. The mixture was heated under reflux in an oil bath (130 °C) for 24 h (reaction was monitored by TLC). After cooling, chloroform (50 mL) was added and then transferred to a separating funnel. The IL was extracted with chloroform (2 × 10 mL). The remaining IL after extraction can be used for the next cycle. The combined chloroform layers were filtered through a silica gel plug (10 mL), which was then flushed with CHCl_3_/EtOAc (5:1 v/v). The filtrate was dried over MgSO_4_ and concentrated using a rotary evaporator. The product was precipitated using EtOAc (5 mL), filtered, washed with fresh EtOAc and air-dried to give 3,5-bis(biphenyl-4-yl)-4-butyl-4*H*-1,2,4-triazole (**7a**) in 83% yield.

## 4. Conclusions

We developed an efficient methodology for the synthesis of four series of 4*H*-1,2,4-triazole derivatives conjugated to different aromatic and heteroaromatic arrangements via 1,4-phenylene linker. The final Suzuki cross-coupling reactions of the intermediate 4-alkyl-3,5-bis(4-bromophenyl)-4*H*-1,2,4-triazoles and boronic acids were conducted by both conventional and alternative ionic liquid (IL) approach. The application of IL in the transformation resulted in the formation of the selected product in high yield with the possibility of regeneration of this green solvent and its subsequent recycling with only a slight decrease in product yield. Generally, products were obtained in excellent yields at each stage of a few-step methodology. The presence of alkyl substituent on the ring nitrogen atom enhances the solubility of the final products, which is particularly important for their potential application in the production of optoelectronic devices. Strong fluorescence has been observed for almost all compounds, except products containing a terminal 3-nitrophenyl substituent. High fluorescence quantum yields were observed, reaching up to 98%, dependent on the nature of terminal substituents, suggesting conjugation extending over all five rings of the investigated systems.

## Figures and Tables

**Table 1:** Products of the reaction of *N*,*N*′-bis(4-bromobenzoyl)hydrazine (**1**) with PCl_5_ and *N*,*N*′-bis[(4-bromophenyl)chloromethylene]hydrazine (**2**) with butylamine.

Entry	Product 2				Product 3c				
	1: PCl_5_ Ratio (equiv.)	Temp. (°C)	Time (min.)	Yield (%)	2: Bu-NH_2_ Ratio (equiv.)	Solvent	Temp. (°C)	Time (h)	Yield (%)
1	1:2	20	60	12	1:2	chloroform	61	10	33
2	1:2	110	60	3	1:2	toluene	110	10	50
3	1:2	110	5	80	1:4	toluene	110	10	71
4	1:4	110	5	56	1:4	toluene	110	20	73

**Table 2:** The conventional Suzuki cross-coupling reaction for 3,5-bis(biphenyl-4-yl)-4-butyl-4*H*-1,2,4-triazole (**7a**).

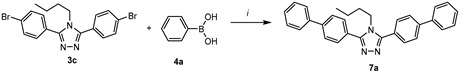					
Entry	3c/4a Ratio (equiv.)	Solvents	PTC Catalyst (equiv.)	Base (equiv.)	Yield (%) ^a^
1	1:2	Toluene/H_2_O/EtOH	NBu_4_Cl (0.1)	Li_2_CO_3_ (10)	36
2	1:2	Toluene/H_2_O/EtOH	NBu_4_Br (0.1)	Li_2_CO_3_ (10)	58
3	1:2.5	Toluene/H_2_O/EtOH	NBu_4_Br (0.1)	Li_2_CO_3_ (10)	89
4	1:2.5	Toluene/H_2_O/EtOH	NBu_4_Br (0.1)	Na_2_CO_3_ (10)	91
5	1:2.5	Toluene/H_2_O/EtOH	NBu_4_Br (0.1)	K_2_CO_3_ (10)	92
6	1:2.5	Toluene/H_2_O/EtOH	NBu_4_Br (0.1)	Cs_2_CO_3_ (10)	87
7	1:2.5	Toluene/H_2_O/EtOH	NBu_4_Br (0.1)	K_3_PO_4_ (10)	8
8	1:2.5	Toluene	-	EtONa (10)	9
9	1:2.5	Toluene	-	*i*PrONa (10)	77
10	1:2.5	Toluene	-	*t*BuONa (10)	88

^a^ Yield with respect to the 4-butyl-3,5-bis(4-bromophenyl)-4*H*-1,2,4-triazole (**3c**). Conditions: oil bath 130 °C, 5 h.

**Table 3:** The synthesis of 3,5-bis(biphenyl-4-yl)-4-butyl-4*H*-1,2,4-triazole (**7a**) in IL.

Entry	IL (10 mL)	Traditional Solvents (1 mL)	Time (h)	Yield (%)
1	BTMA-OH	Chloroform	24	-
2	BTMA-OH	Toluene	24	-
3	Choline-OH	Chloroform	24	-
4	Choline-OH	Toluene	24	83
5	HDTMA-OH	Chloroform	24	-
6	HDTMA-OH	Toluene	24	-
7	TBA-OH	Chloroform	24	-
8	TBA-OH	Toluene	24	-

**Table 4:** 4-Alkyl-3,5-bis(4-arylphenyl)-4*H*-1,2,4-triazoles **5a–n, 6a–n, 7a–n, 8a–n** prepared in Suzuki cross-coupling reaction.

	Central Core	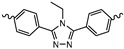	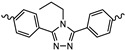	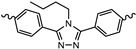	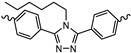
Ar Substituent					
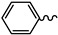		**5a** Y = 87 ^a^ Φ = 0.797/*0.886* ^b^ Δ = 102 ^c^ $\lambda_{\max}^{\mathrm{abs}}$ = 281 $\lambda_{\max}^{\mathrm{ex}}$ = 292 $\lambda_{\max}^{\mathrm{em}}$ = 383	**6a** Y = 94 ^a^ Φ = 0.763/*0.848* ^b^ Δ = 99 ^c^ $\lambda_{\max}^{\mathrm{abs}}$ = 282 $\lambda_{\max}^{\mathrm{ex}}$ = 290 $\lambda_{\max}^{\mathrm{em}}$ = 381	**7a** Y = 92 ^a^ Φ = 0.793/*0.882* ^b^ Δ = 99 ^c^ $\lambda_{max}^{abs}$ = 282 $\lambda_{max}^{ex}$ = 293 $\lambda_{max}^{em}$ = 381	**8a** Y = 92 ^a^ Φ = 0.663/*0.737* ^b^ Δ = 100 ^c^ $\lambda_{max}^{abs}$ = 282 $\lambda_{max}^{ex}$ = 292 $\lambda_{max}^{em}$ = 382
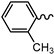		**5b** Y = 85 ^a^ Φ = 0.496/*0.552* ^b^ Δ = 118 ^c^ $\lambda_{max}^{abs}$ = 268 $\lambda_{max}^{ex}$ = 286 $\lambda_{max}^{em}$ = 386	**6b** Y = 81 ^a^ Φ = 0.687/*0.764* ^b^Δ = 104 ^c^ $\lambda_{max}^{abs}$ = 268 $\lambda_{max}^{ex}$ = 286 $\lambda_{max}^{em}$ = 372	**7b** Y = 83 ^a^ Φ = 0.433/*0.482* ^b^ Δ = 110 ^c^ $\lambda_{max}^{abs}$ = 269 $\lambda_{max}^{ex}$ = 282 $\lambda_{max}^{em}$ = 379	**8b** Y = 7 ^a^ Φ = 0.655/*0.728* ^b^ Δ = 105 ^c^ $\lambda_{max}^{abs}$ = 269 $\lambda_{max}^{ex}$ = 283 $\lambda_{max}^{em}$ = 374
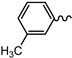		**5c** Y = 97 ^a^ Φ = 0.601/*0.668* ^b^ Δ = 113 ^c^ $\lambda_{max}^{abs}$ = 283 $\lambda_{max}^{ex}$ = 293 $\lambda_{max}^{em}$ = 396	**6c** Y = 91 ^a^ Φ = 0.781/*0.869* ^b^ Δ = 97 ^c^ $\lambda_{max}^{abs}$ = 284 $\lambda_{max}^{ex}$ = 292 $\lambda_{max}^{em}$ = 381	**7c** Y = 96 ^a^ Φ = 0.634/*0.705* ^b^ Δ = 97 ^c^ $\lambda_{max}^{abs}$ = 285 $\lambda_{max}^{ex}$ = 293 $\lambda_{max}^{em}$ = 382	**8c** Y = 91^a^ Φ = 0.830/*0.923* ^b^ Δ = 97 ^c^ $\lambda_{max}^{abs}$ = 284 $\lambda_{max}^{ex}$ = 293 $\lambda_{max}^{em}$= 381
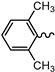		**5d** Y = 90 ^a^ Φ = 0.363/*0.404* ^b^ Δ = 128 ^c^ $\lambda_{max}^{abs}$ = 259 $\lambda_{max}^{ex}$ = 281 $\lambda_{max}^{em}$ = 387	**6d** Y = 94 ^a^ Φ = 0.523/*0.582* ^b^ Δ = 105 ^c^ $\lambda_{max}^{abs}$ = 259 $\lambda_{max}^{ex}$ = 278 $\lambda_{max}^{em}$ = 364	**7d** Y = 79 ^a^ Φ = 0.262/*0.291* ^b^ Δ = 105 ^c^ $\lambda_{max}^{abs}$ = 258 $\lambda_{max}^{ex}$ = 275 $\lambda_{max}^{em}$ = 363	**8d** Y = 93 ^a^ Φ = 0.593/*0.659* ^b^ Δ = 114 ^c^ $\lambda_{max}^{abs}$ = 259 $\lambda_{max}^{ex}$ = 281 $\lambda_{max}^{em}$ = 373
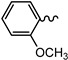		**5e** Y = 91 ^a^ Φ = 0.556/*0.618* ^b^ Δ = 132 ^c^ $\lambda_{max}^{abs}$ = 296 $\lambda_{max}^{ex}$ = 303 $\lambda_{max}^{em}$ = 404	**6e** Y = 91 ^a^ Φ = 0.864/*0.961* ^b^ Δ = 84 ^c^ $\lambda_{max}^{abs}$ = 296 $\lambda_{max}^{ex}$ = 302 $\lambda_{max}^{em}$ = 380	**7e** Y = 85 ^a^ Φ = 0.601/*0.668* ^b^ Δ = 85 ^c^ $\lambda_{max}^{abs}$ = 295 $\lambda_{max}^{ex}$ = 301 $\lambda_{max}^{em}$ = 380	**8e** Y = 88 ^a^ Φ = 0.565/*0.629* ^b^ Δ = 90 ^c^ $\lambda_{max}^{abs}$ = 296 $\lambda_{max}^{ex}$ = 302 $\lambda_{max}^{em}$ = 386
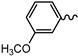		**5f** Y = 99 ^a^ Φ = 0.770/*0.856* ^b^ Δ = 100 ^c^ $\lambda_{max}^{abs}$ = 282 $\lambda_{max}^{ex}$ = 297 $\lambda_{max}^{em}$ = 382	**6f** Y = 95 ^a^ Φ = 0.699/*0.777* ^b^ Δ = 99 ^c^ $\lambda_{max}^{abs}$ = 283 $\lambda_{max}^{ex}$ = 298 $\lambda_{max}^{em}$ = 382	**7f** Y = 8 ^a^ Φ = 0.680/*0.756* ^b^ Δ = 97 ^c^ $\lambda_{max}^{abs}$ = 284 $\lambda_{max}^{ex}$ = 296 $\lambda_{max}^{em}$ = 381	**8f** Y = 83 ^a^ Φ = 0.583/*0.648* ^b^ Δ = 100 ^c^ $\lambda_{max}^{abs}$ = 284 $\lambda_{max}^{ex}$ = 299 $\lambda_{max}^{em}$ = 384
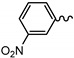		**5g** Y = 94 ^a^ Φ = 0.052/*0.057* ^b^ Δ = 105 ^c^ $\lambda_{max}^{abs}$ = 275 $\lambda_{max}^{ex}$ = 300 $\lambda_{max}^{em}$ = 380	**6g** Y = 91 ^a^ Φ = 0.027/*0.031* ^b^ Δ = 104 ^c^ $\lambda_{max}^{abs}$ = 276 $\lambda_{max}^{ex}$ = 290 $\lambda_{max}^{em}$ = 380	**7g** Y = 91 ^a^ Φ = 0.016/*0.018* ^b^ Δ = 119 ^c^ $\lambda_{max}^{abs}$ = 276 $\lambda_{max}^{ex}$ = 310 $\lambda_{max}^{em}$ = 295	**8g** Y = 85 ^a^ Φ = 0.010/*0.011* ^b^ Δ = 109 ^c^ $\lambda_{max}^{abs}$ = 276 $\lambda_{max}^{ex}$ = 310 $\lambda_{max}^{em}$ = 285
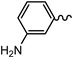		**5h** Y = 99^a^ Φ = 0.323/*0.360* ^b^ Δ = 126 ^c^ $\lambda_{max}^{abs}$ = 280 $\lambda_{max}^{ex}$ = 292 $\lambda_{max}^{em}$ = 406	**6h** Y = 99 ^a^ Φ = 0.298/*0.331* ^b^ Δ = 123 ^c^ $\lambda_{max}^{abs}$ = 282 $\lambda_{max}^{ex}$ = 290 $\lambda_{max}^{em}$ = 405	**7h** Y = 98 ^a^ Φ = 0.328/*0.364* ^b^ Δ = 125 ^c^ $\lambda_{max}^{abs}$ = 281 $\lambda_{max}^{ex}$ = 292 $\lambda_{max}^{em}$ = 406	**8h** Y = 78 ^a^ Φ = 0.131/*0.146* ^b^ Δ = 122 ^c^ $\lambda_{max}^{abs}$ = 281 $\lambda_{max}^{ex}$ = 293 $\lambda_{max}^{em}$ = 403
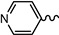		**5i** Y = 89^a^ Φ = 0.574/*0.638* ^b^ Δ = 107 ^c^ $\lambda_{max}^{abs}$ = 280 $\lambda_{max}^{ex}$ = 291 $\lambda_{max}^{em}$ = 387	**6i** Y = 90 ^a^ Φ = 0.363/*0.403* ^b^ Δ = 107 ^c^ $\lambda_{max}^{abs}$ = 281 $\lambda_{max}^{ex}$ = 291 $\lambda_{max}^{em}$ = 388	**7i** Y = 87 ^a^ Φ > 0.98 ^b,d^ Δ = 169 ^c^ $\lambda_{max}^{abs}$ = 281 $\lambda_{max}^{ex}$ = 324 $\lambda_{max}^{em}$ = 450	**8i** Y = 86 ^a^ Φ = 0.560/*0.623* ^b^ Δ = 107 ^c^ $\lambda_{max}^{abs}$ = 281 $\lambda_{max}^{ex}$ = 292 $\lambda_{max}^{em}$ = 388
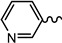		**5j** Y = 93^a^ Φ = 0.557/*0.619* ^b^ Δ = 95 ^c^ $\lambda_{max}^{abs}$ = 284 $\lambda_{max}^{ex}$ = 291 $\lambda_{max\;}^{em}$ = 379	**6j** Y = 69 ^a^ Φ = 0.612/*0.680* ^b^ Δ = 97 ^c^ $\lambda_{max}^{abs}$ = 284 $\lambda_{max}^{ex}$ = 292 $\lambda_{max}^{em}$ = 381	**7j** Y = 74 ^a^ Φ = 0.149/*0.165* ^b^ Δ = 148 ^c^ $\lambda_{max}^{abs}$ = 285 $\lambda_{max}^{ex}$ = 299 $\lambda_{max}^{em}$ = 433	**8j** Y = 83 ^a^ Φ = 0.262/*0.291* ^b^ Δ = 147 ^c^ $\lambda_{max}^{abs}$ $=284\;\lambda_{max}^{ex}$ $=300\;\lambda_{max}^{em}$ = 431
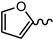		**5k** Y = 92 ^a^ Φ > 0.98 ^b,d^ Δ = 85 ^c^ $\lambda_{max}^{abs}$ = 310 $\lambda_{max}^{ex}$ = 314 $\lambda_{max}^{em}$ = 395	**6k** Y = 96 ^a^ Φ > 0.98 ^b,d^ Δ = 83 ^b^ $\lambda_{max}^{abs}$ = 311 $\lambda_{max}^{ex}$ = 314 $\lambda_{max}^{em}$ = 394	**7k** Y = 75 ^a^ Φ = 0.857/*0.953* ^b^ Δ = 91 ^c^ $\lambda_{max}^{abs}$ = 310 $\lambda_{max}^{ex}$ = 316 $\lambda_{max}^{em}$ = 401	**8k** Y = 89 ^a^ Φ > 0.98 ^b,d^ Δ = 85 ^c^ $\lambda_{max}^{abs}$ = 310 $\lambda_{max}^{ex}$ = 314 $\lambda_{max}^{em}$ = 395
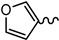		**5l** Y = 91^a^ Φ = 0.658/*0.732* ^b^ Δ = 91 ^c^ $\lambda_{max}^{abs}$ = 283 $\lambda_{max}^{ex}$ = 293 $\lambda_{max}^{em}$ = 374	**6l** Y = 92 ^a^ Φ = 0.622/*0.691* ^b^ Δ = 93 ^c^ $\lambda_{max}^{abs}$ = 284 $\lambda_{max}^{ex}$ = 292 $\lambda_{max}^{em}$ = 377	**7l** Y = 94^a^ Φ = 0.642/*0.714* ^b^ Δ = 102 ^c^ $\lambda_{max}^{abs}$ = 285 $\lambda_{max}^{ex}$ = 294 $\lambda_{max}^{em}$ = 387	**8l** Y = 69 ^a^ Φ = 0.580/*0.645* ^b^ Δ = 95 ^c^ $\lambda_{max}^{abs}$ = 284 $\lambda_{max}^{ex}$ = 293 $\lambda_{max}^{em}$ = 379
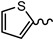		**5m** Y = 61 ^a^ Φ = 0.716/*0.796* ^b^ Δ = 87 ^c^ $\lambda_{max}^{abs}$ = 311 $\lambda_{max}^{ex}$ = 316 $\lambda_{max}^{em}$ = 398	**6m** Y = 52 ^a^ Φ = 0.750/*0.834* ^b^ Δ = 85 ^c^ $\lambda_{max}^{abs}$ = 312 $\lambda_{max}^{ex}$ = 315 $\lambda_{max}^{em}$ = 397	**7m** Y = 49 ^a^ Φ = 0.685/*0.762* ^b^ Δ = 89 ^c^ $\lambda_{max}^{abs}$ = 312 $\lambda_{max}^{ex}$ = 320 $\lambda_{max}^{em}$ = 401	**8m** Y = 37 ^a^ Φ = 0.753/*0.837* ^b^ Δ = 85 ^c^ $\lambda_{max}^{abs}$ = 312 $\lambda_{max}^{ex}$ = 316 $\lambda_{max}^{em}$ = 397
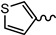		**5n** Y = 90^a^ Φ = 0.545/*0.605* ^b^ Δ = 111 ^c^ $\lambda_{max}^{abs}$ = 290 $\lambda_{max}^{ex}$ = 301 $\lambda_{max}^{em}$ = 401	**6n** Y = 90^a^ Φ = 0.793/*0.881* ^b^ Δ = 85 ^c^ $\lambda_{max}^{abs}$ = 290 $\lambda_{max}^{ex}$ = 298 $\lambda_{max}^{em}$ = 382	**7n** Y = 77 ^a^ Φ = 0.513/*0.570* ^b^ Δ = 104 ^c^ $\lambda_{max}^{abs}$ = 289 $\lambda_{max}^{ex}$ = 302 $\lambda_{max}^{em}$ = 393	**8n** Y = 62 ^a^ Φ = 0.524/*0.583* ^b^ Δ = 95 ^c^ $\lambda_{max}^{abs}$ = 290 $\lambda_{max}^{ex}$ = 299 $\lambda_{max}^{em}$ = 385

^a^ The reaction yield (Y) [%]. ^b^ Quantum yield (Φ) [29]. Quinine sulfate [30] and *trans,trans-1,4-diphenyl-1,3-butadiene* [31] were used as standards. ^c^ Stokes shift (Δ) from the equation $\Delta =\lambda_{max}^{em}-\lambda_{max}^{abs}$. Wavelength determined from the 3D emission spectrum [nm]. ^d^ Exact value cannot be determined due to nonlinearity of standard/sample dependence [29] in the Φ region 0.97–1.00.

## Supplementary Materials

Copies of the ^1^H-NMR and ^13^C-NMR spectra and 3D emission spectra of the compounds are available in the online Supplementary Materials.
