# Supplementary material for: Synthesis of 4-Alkyl-4H-1,2,4-triazole Derivatives by Suzuki Cross-Coupling Reactions and Their Luminescence Properties
Source: Molecules. 2019 Feb 12;24(3):652. doi: 10.3390/molecules24030652 (PMC6384732; doi:10.3390/molecules24030652)

Supplementary Material

Synthesis of 4-Alkyl-4*H*-1,2,4-triazole Derivatives by Suzuki Cross-coupling Reactions and Their Luminescence Properties

Monika Olesiejuk [1], Agnieszka Kudelko [1,*], Marcin Swiatkowski [2] and Rafal Kruszynski [2]

[1] Department of Chemical Organic Technology and Petrochemistry, The Silesian University of Technology, Krzywoustego 4, PL-44100 Gliwice, Poland; monika.olesiejuk@polsl.pl (M.O.); agnieszka.kudelko@polsl.pl (A.K)

[2] Department of X-ray Crystallography and Crystal Chemistry, Institute of General and Ecological Chemistry, Lodz University of Technology, Żeromskiego 116, PL-90924 Łódź, Poland; marcin.swiatkowski@p.lodz.pl (M.S.); rafal.kruszynski@p.lodz.pl (R.K.)

* Correspondence: agnieszka.kudelko@polsl.pl; Tel.: +48-32-237-17-29

Table of contents

1. ^{1}H NMR and ^{13}C NMR spectra of compounds 2
2. 3D emission spectra of compounds 62

1. ^{1}H NMR and ^{13}C NMR spectra of compounds

^{1}H and ^{13}C NMR of 3,5-bis(4-bromophenyl)-4-ethyl-4*H*-1,2,4-triazole **(3a)**

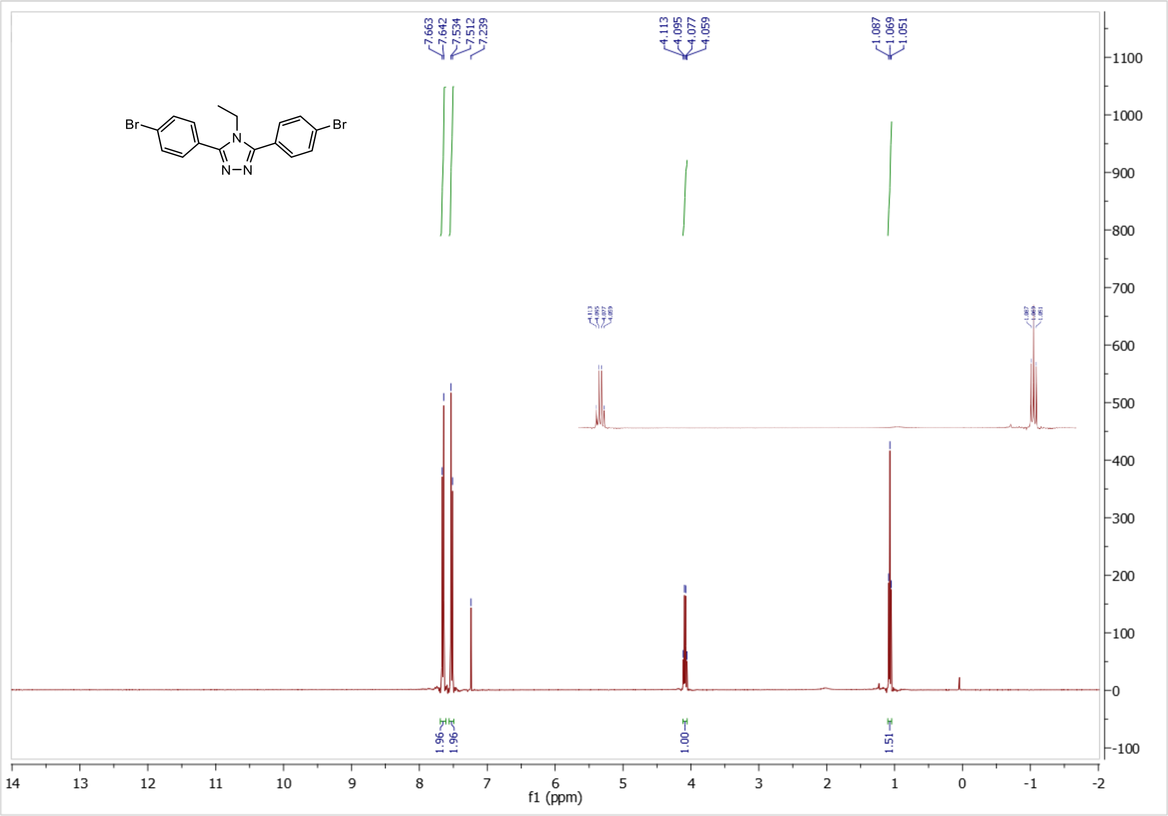

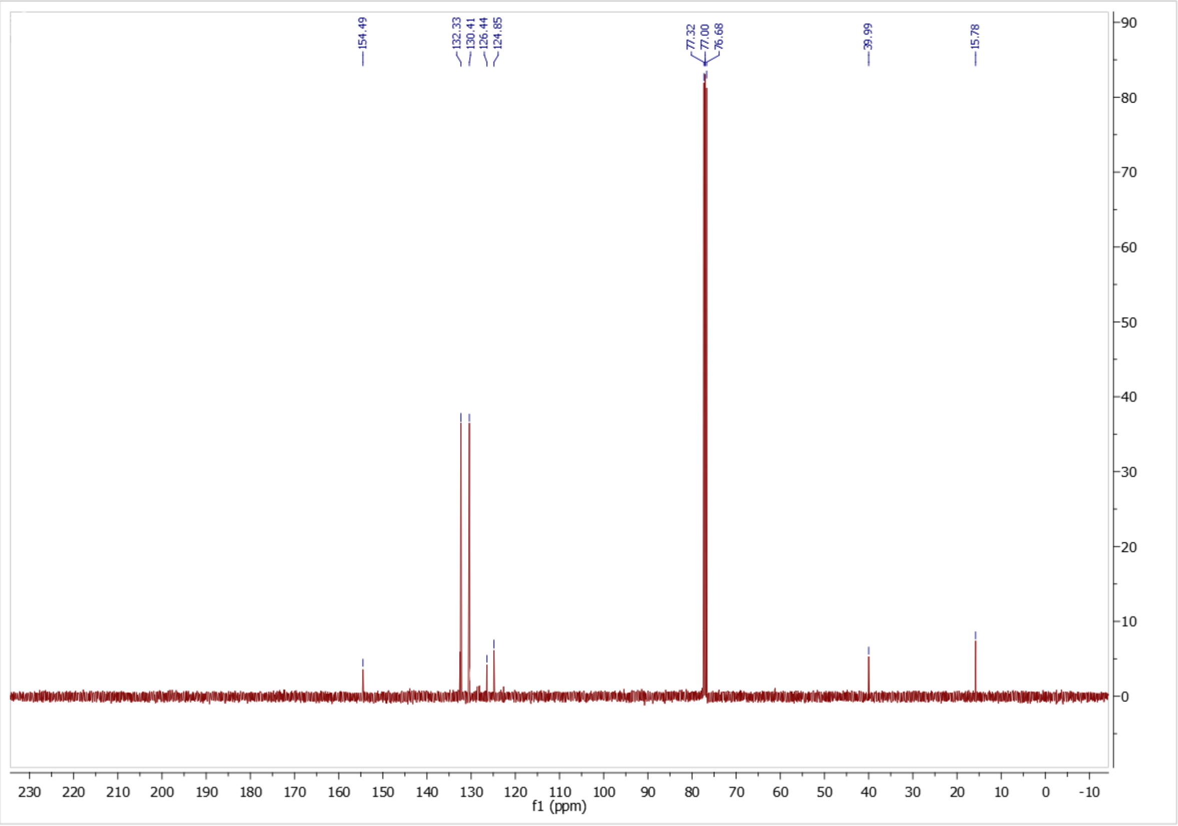

^{1}H and ^{13}C NMR of 3,5-bis(4-bromophenyl)-4-propyl-4*H*-1,2,4-triazole **(3b)**

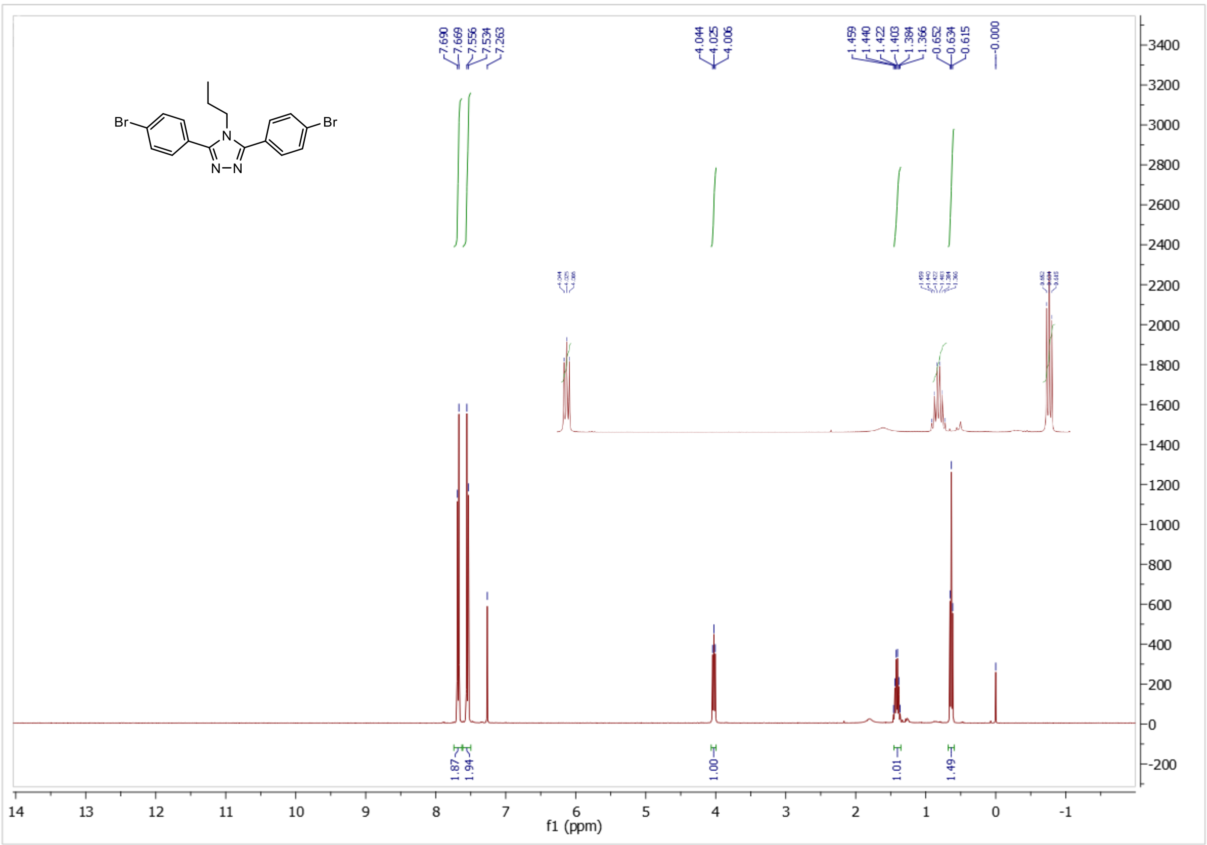

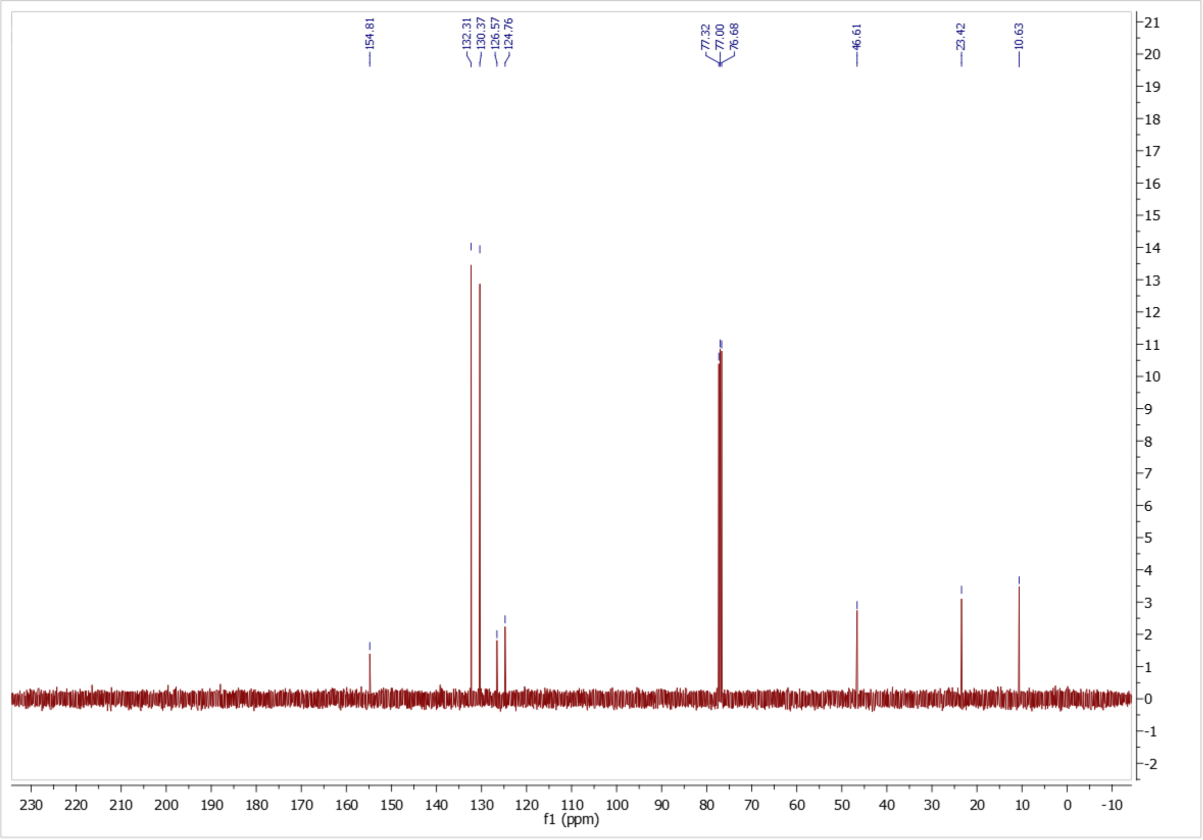

^{1}H and ^{13}C NMR of 3,5-bis(4-bromophenyl)-4-butyl-4*H*-1,2,4-triazole **(3c)**

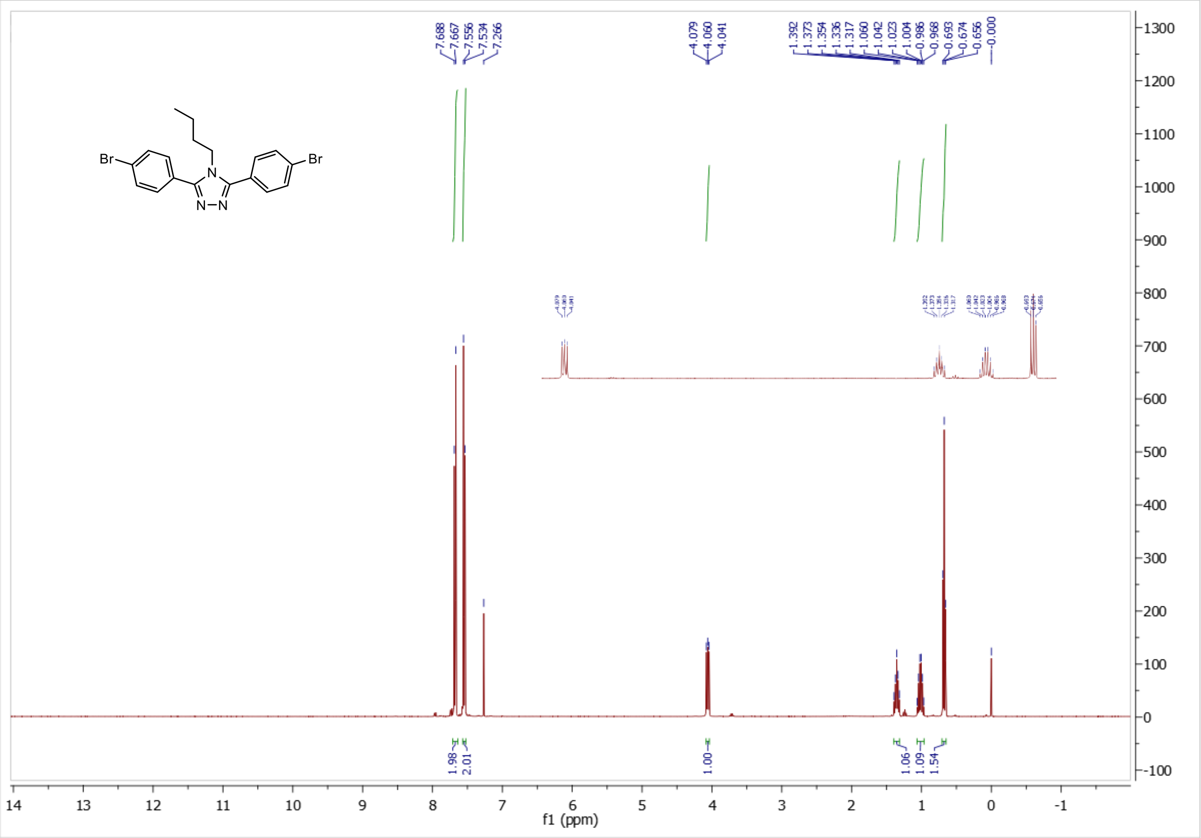

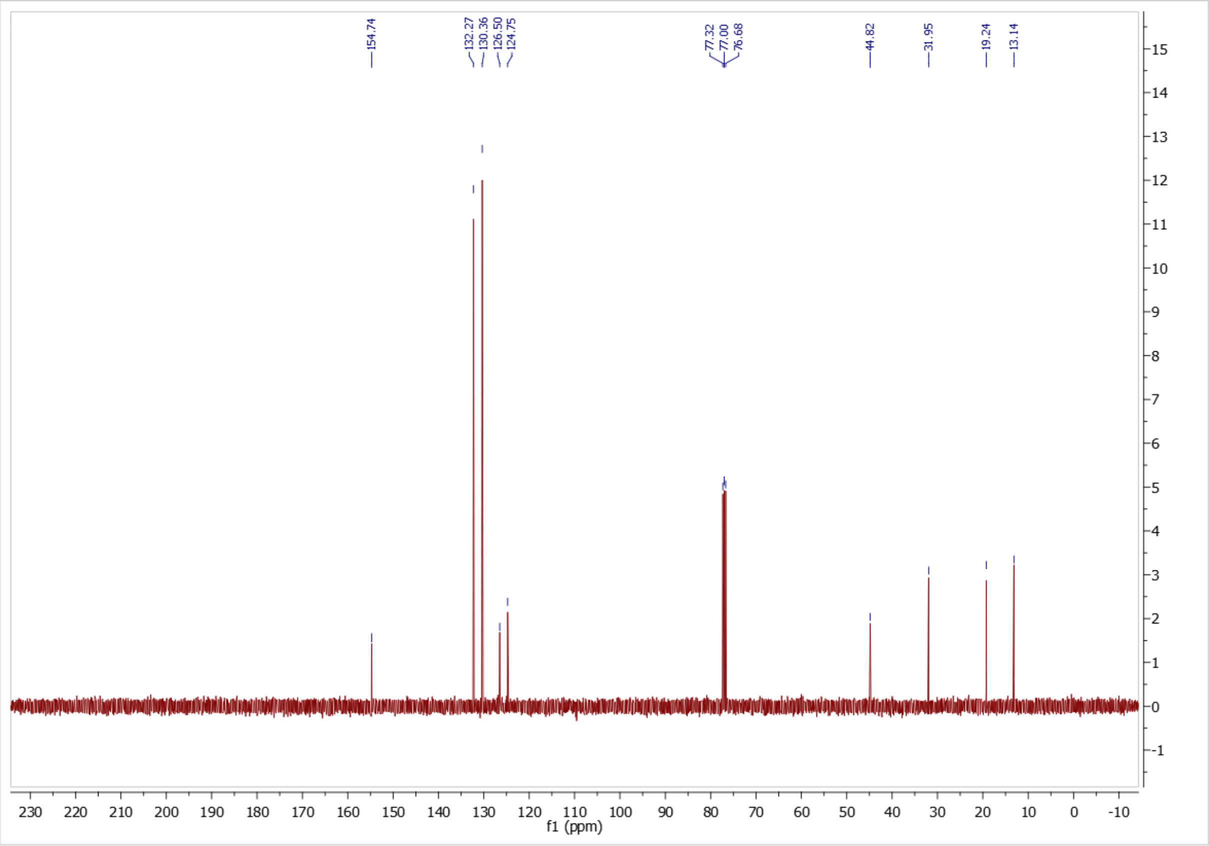

^{1}H and ^{13}C NMR of 3,5-bis(4-bromophenyl)-4-hexyl-4*H*-1,2,4-triazole **(3d)**

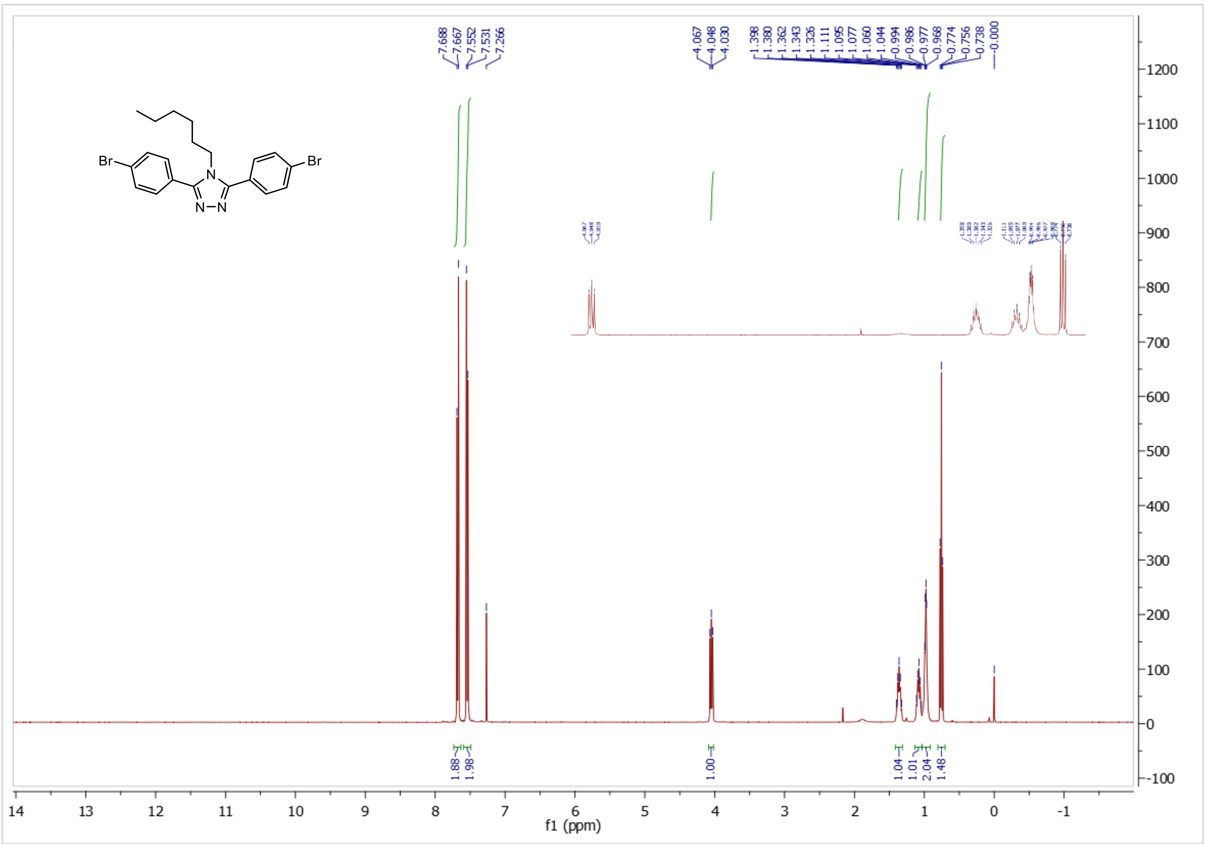

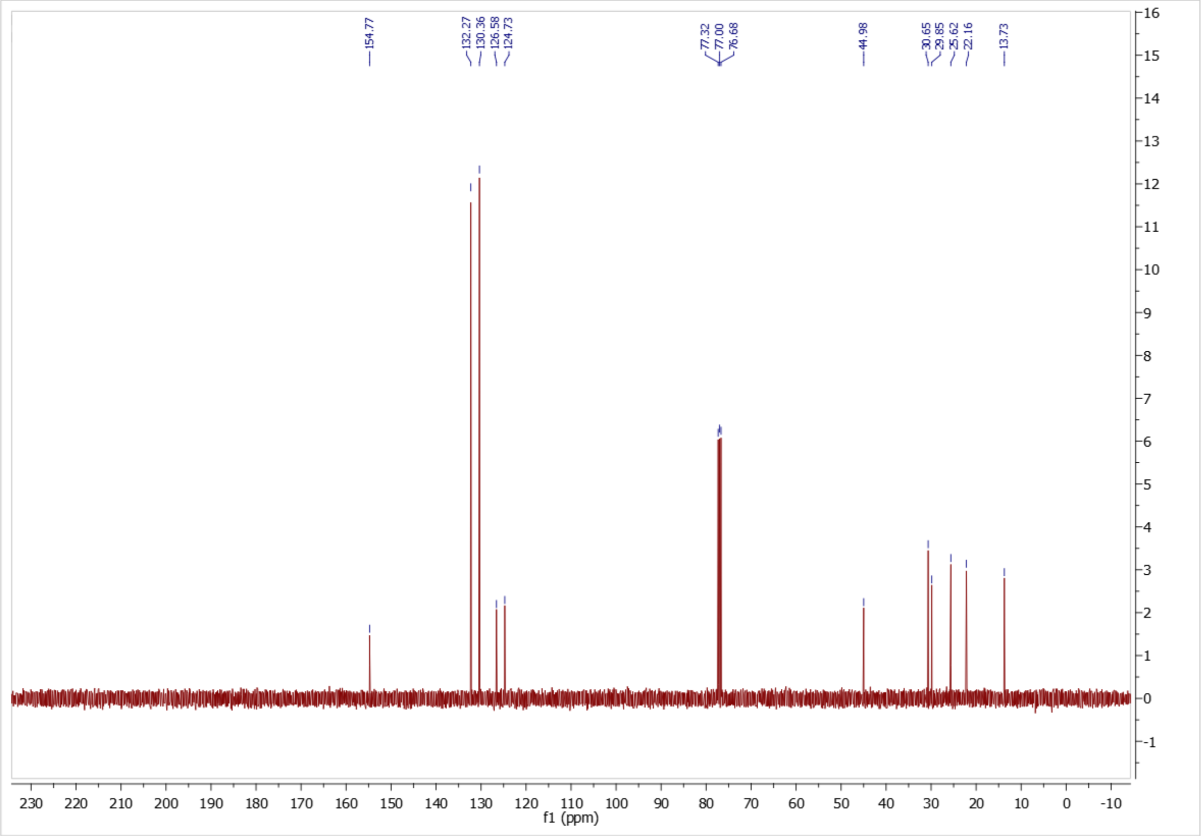

^{1}H and ^{13}C NMR of 3,5-bis(biphenyl-4-yl)-4-ethyl-4*H*-1,2,4-triazole **(5a)**

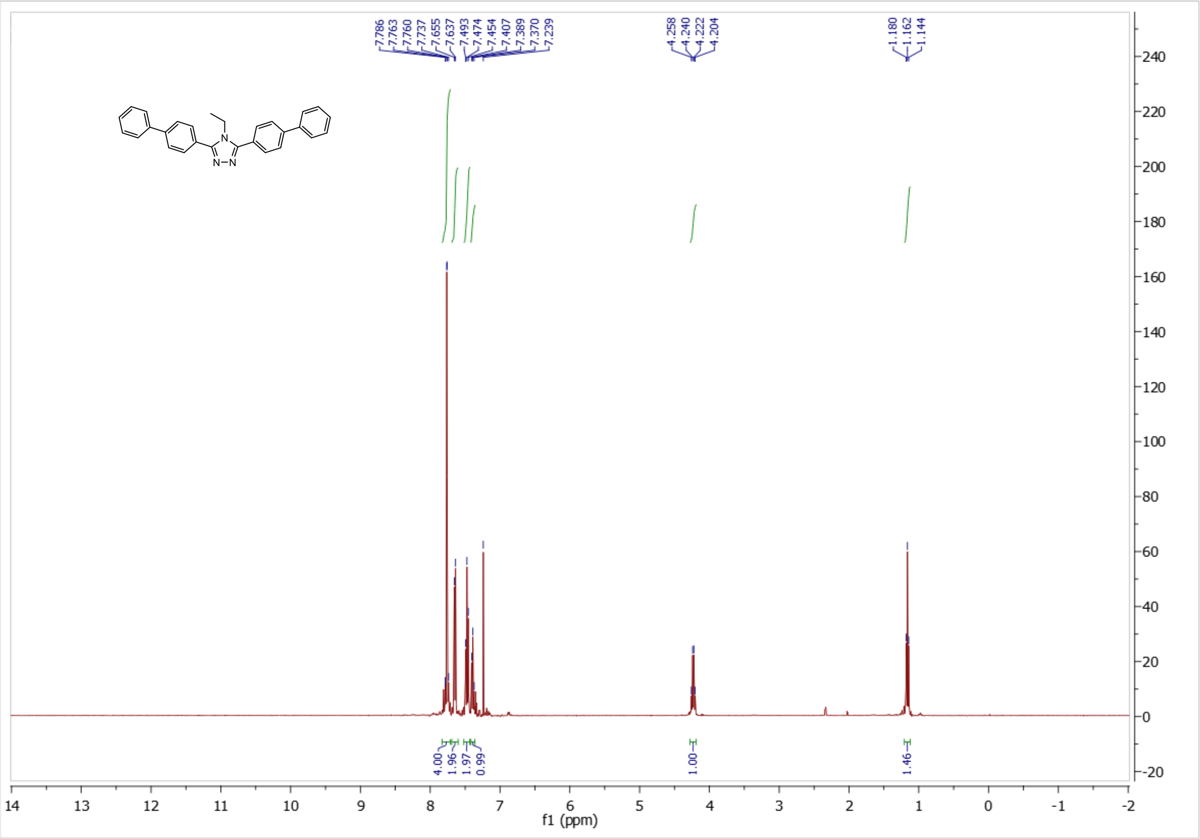

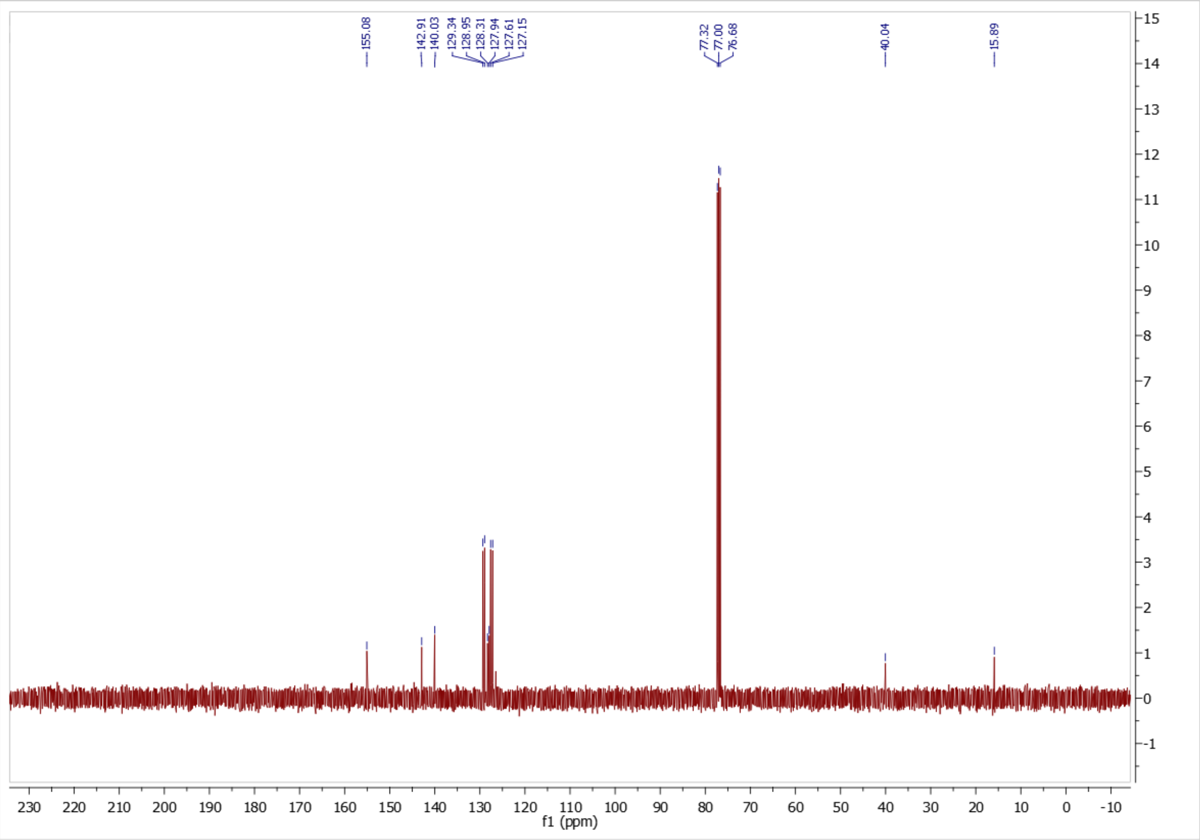

^{1}H and ^{13}C NMR of 4-ethyl-3,5-bis(2'-methylbiphenyl-4-yl)-4*H*-1,2,4-triazole **(5b)**

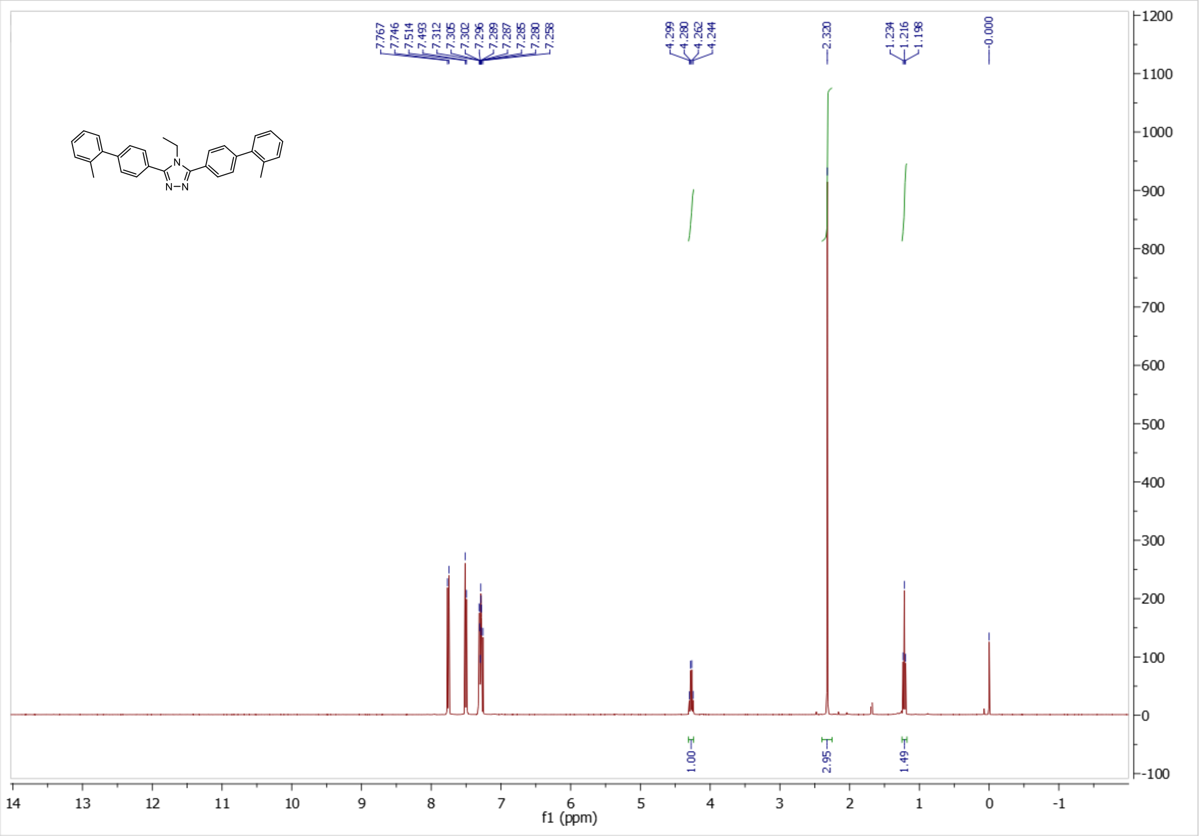

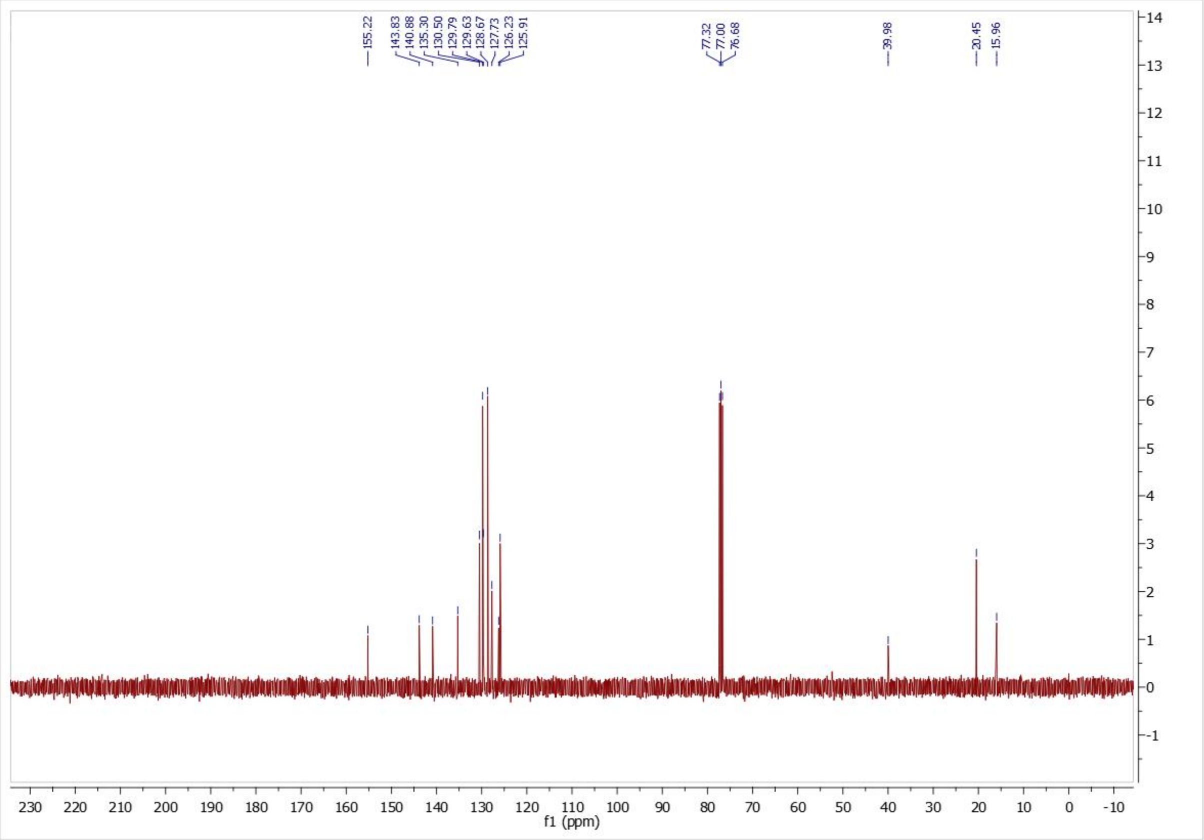

^{1}H and ^{13}C NMR of 4-ethyl-3,5-bis(3’-methylbiphenyl-4-yl)-4*H*-1,2,4-triazole **(5c)**

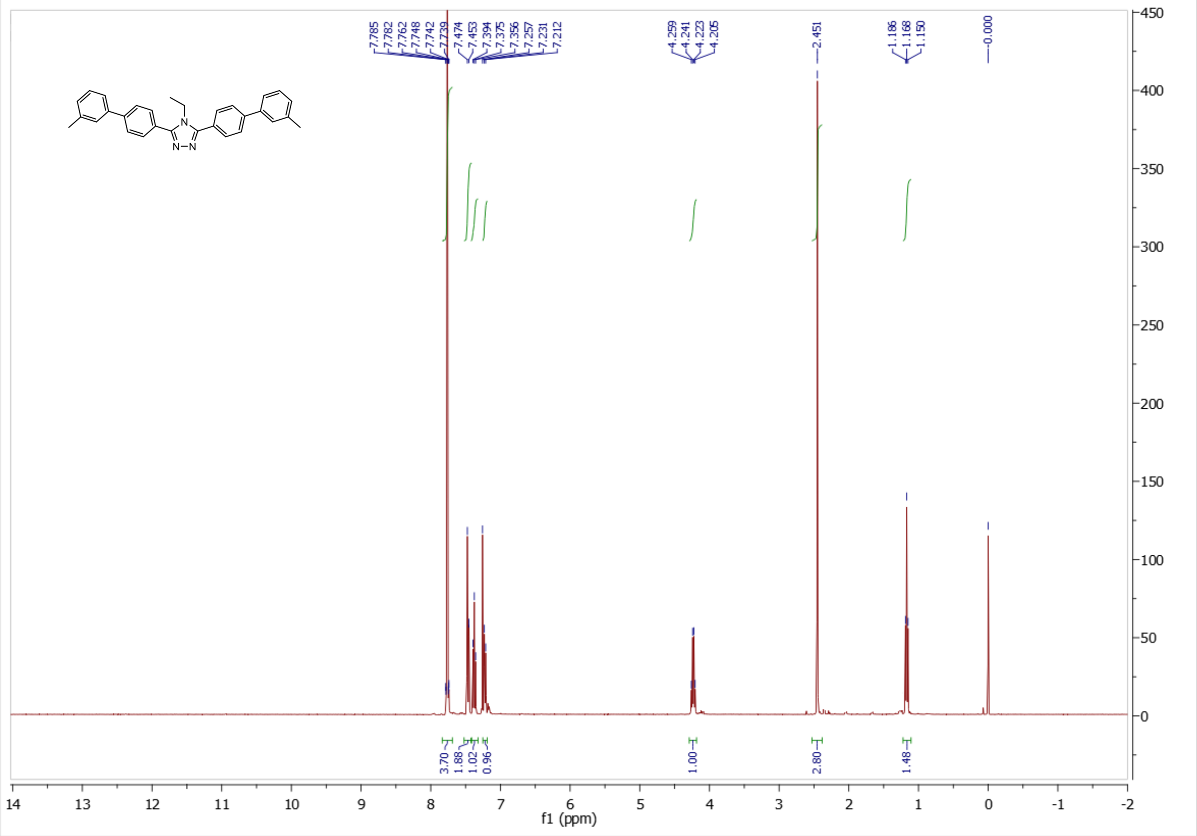

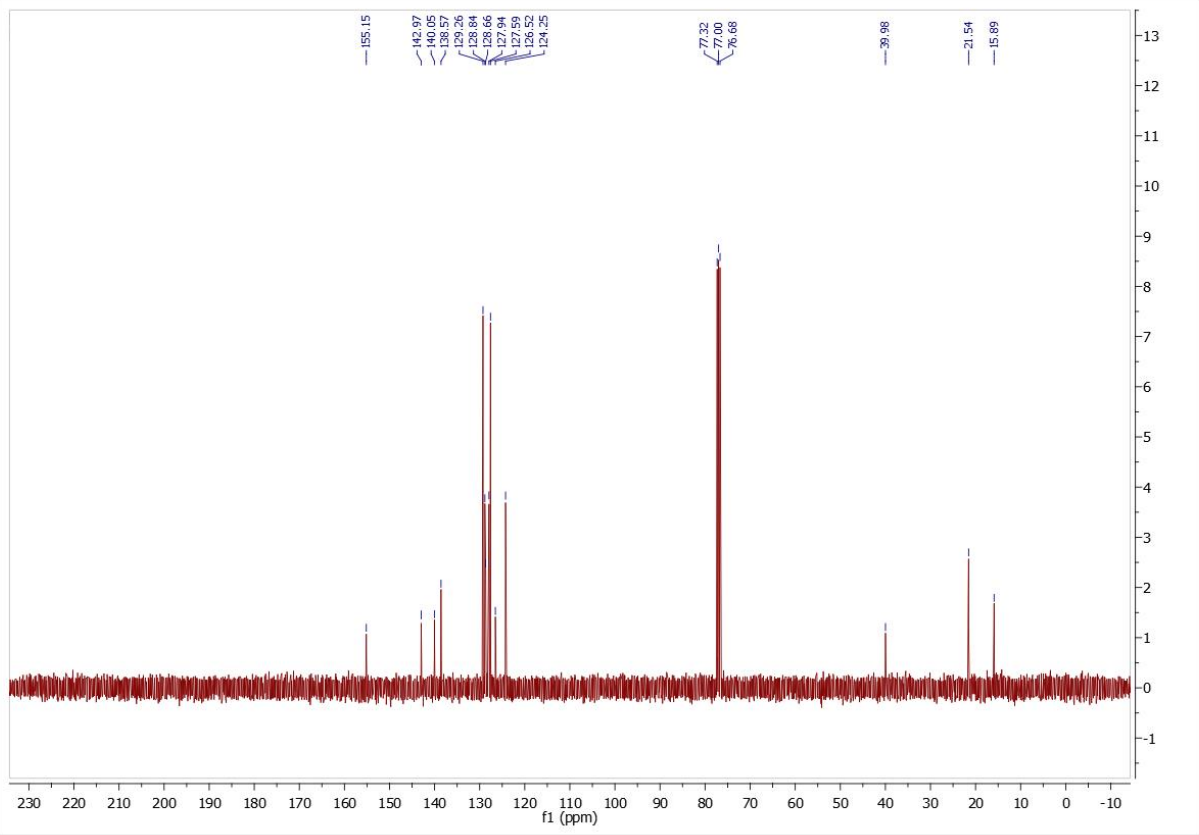

^{1}H and ^{13}C NMR of 4-ethyl-3,5-bis(2',6'-dimethylbiphenyl-4-yl)-4*H*-1,2,4-triazole **(5d)**

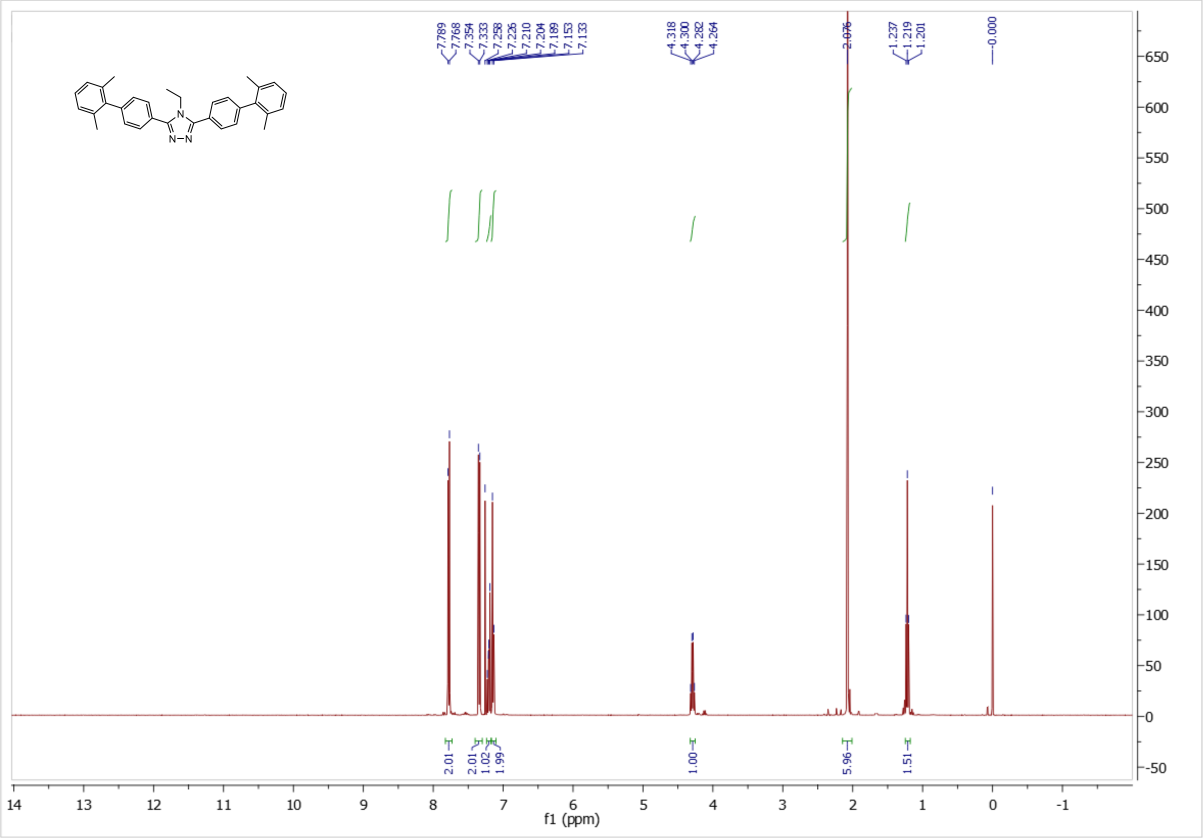

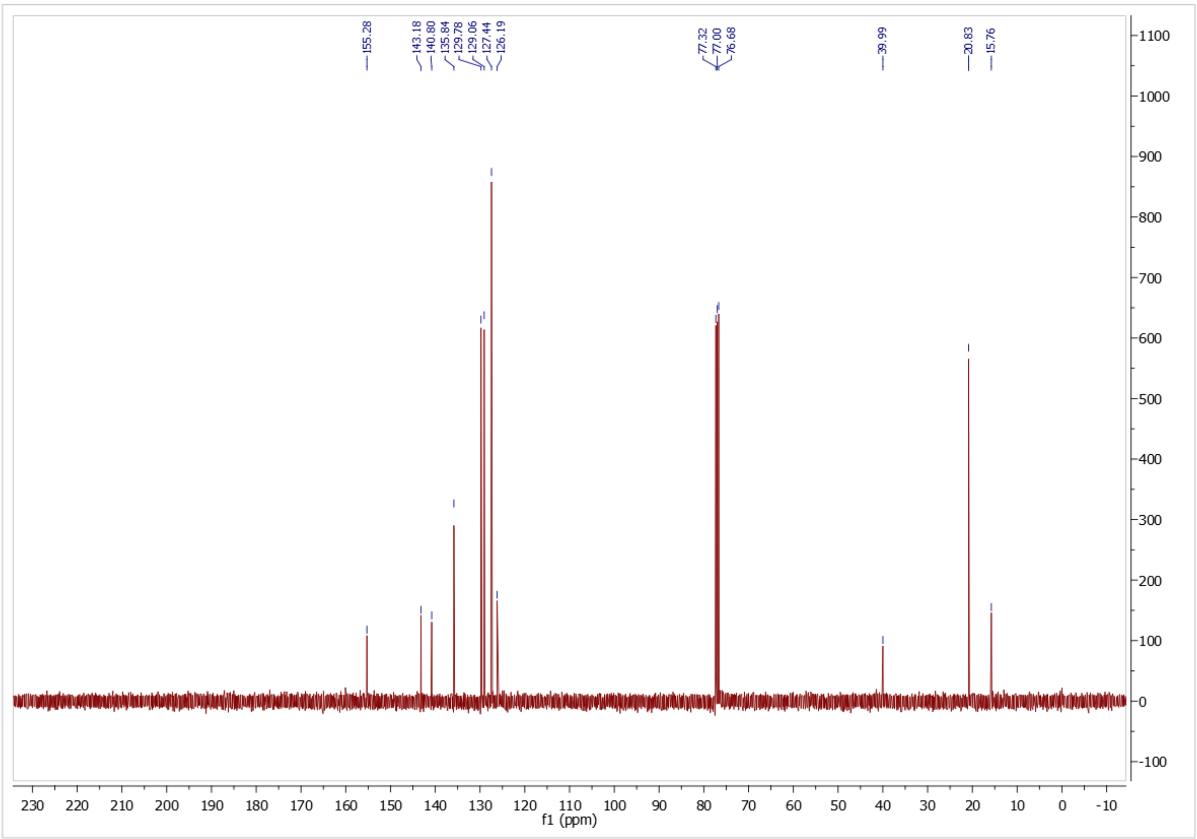

^{1}H and ^{13}C NMR of 4-ethyl-3,5-bis(2'-methoxybiphenyl-4-yl)-4*H*-1,2,4-triazole **(5e)**

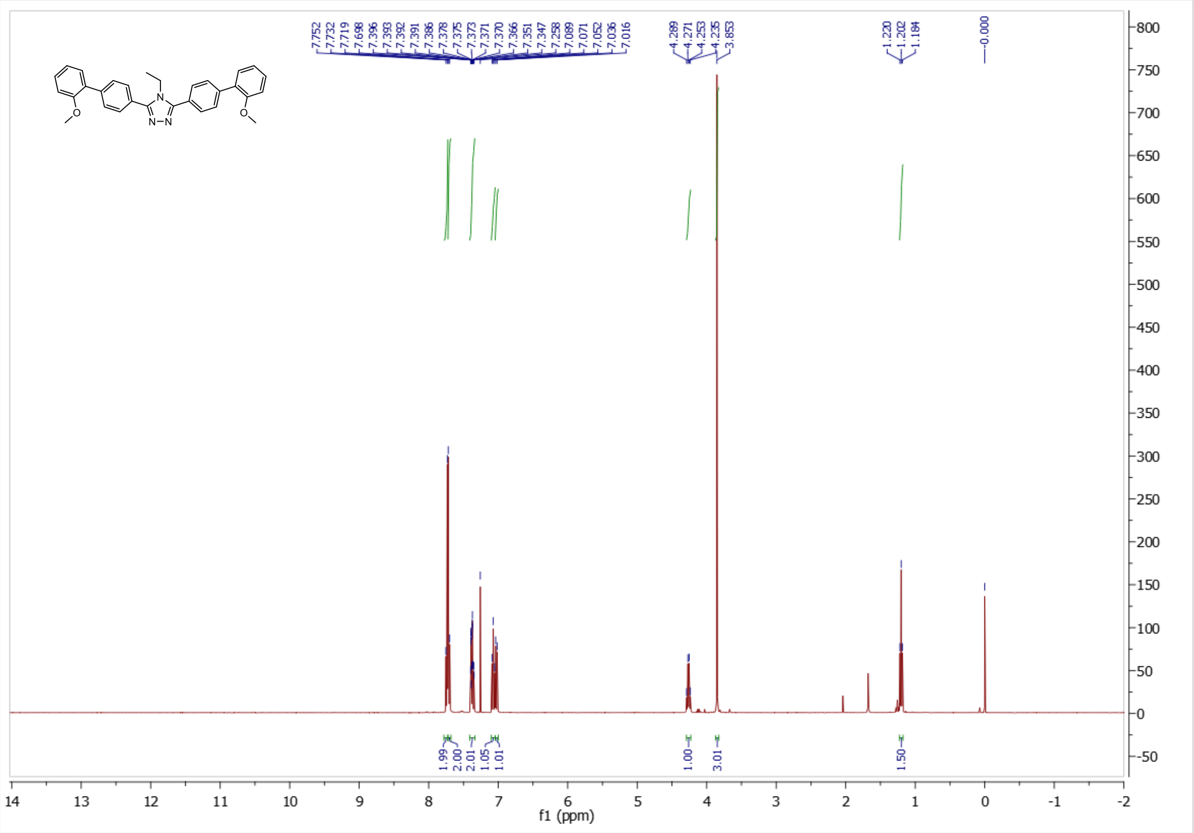

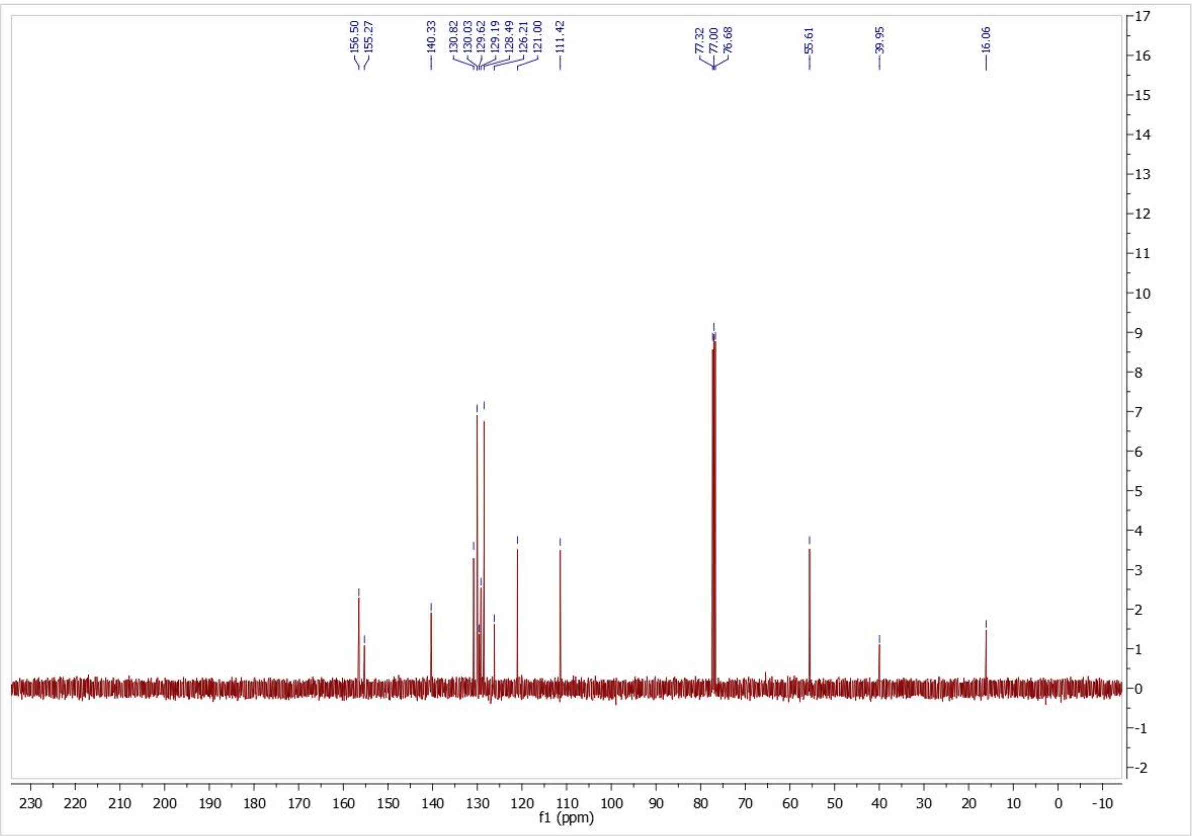

^{1}H and ^{13}C NMR of 4-ethyl-3,5-bis(3'-methoxybiphenyl-4-yl)-4*H*-1,2,4-triazole **(5f)**

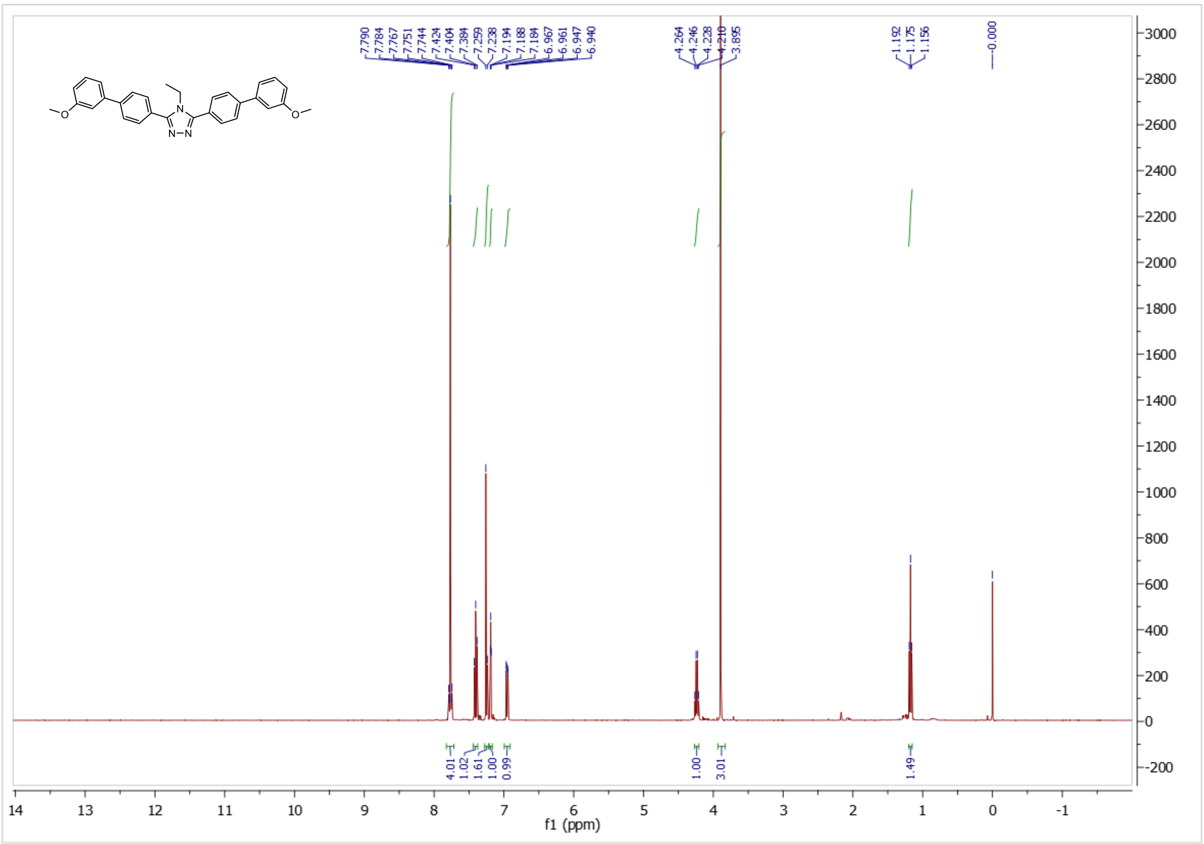

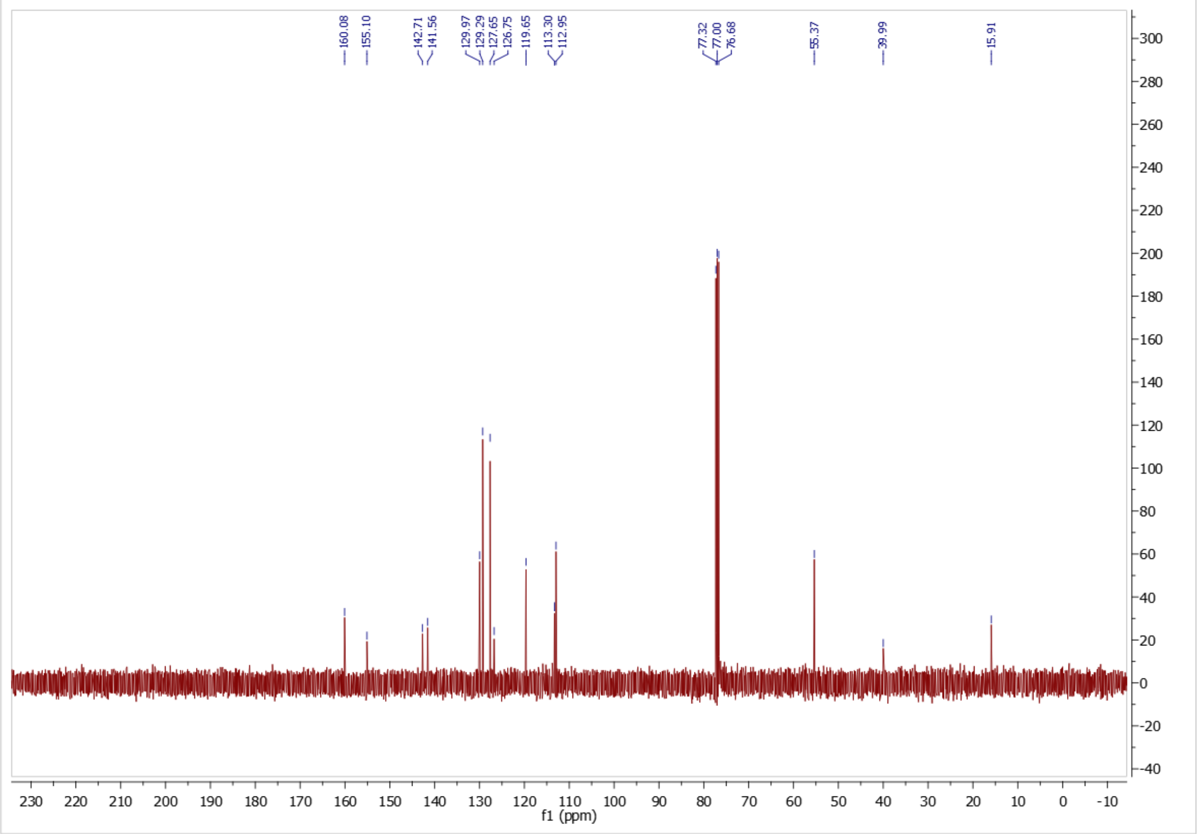

^{1}H and ^{13}C NMR of 4-ethyl-3,5-bis(3’-nitrobiphenyl-4-yl)-4*H*-1,2,4-triazole **(5g)**

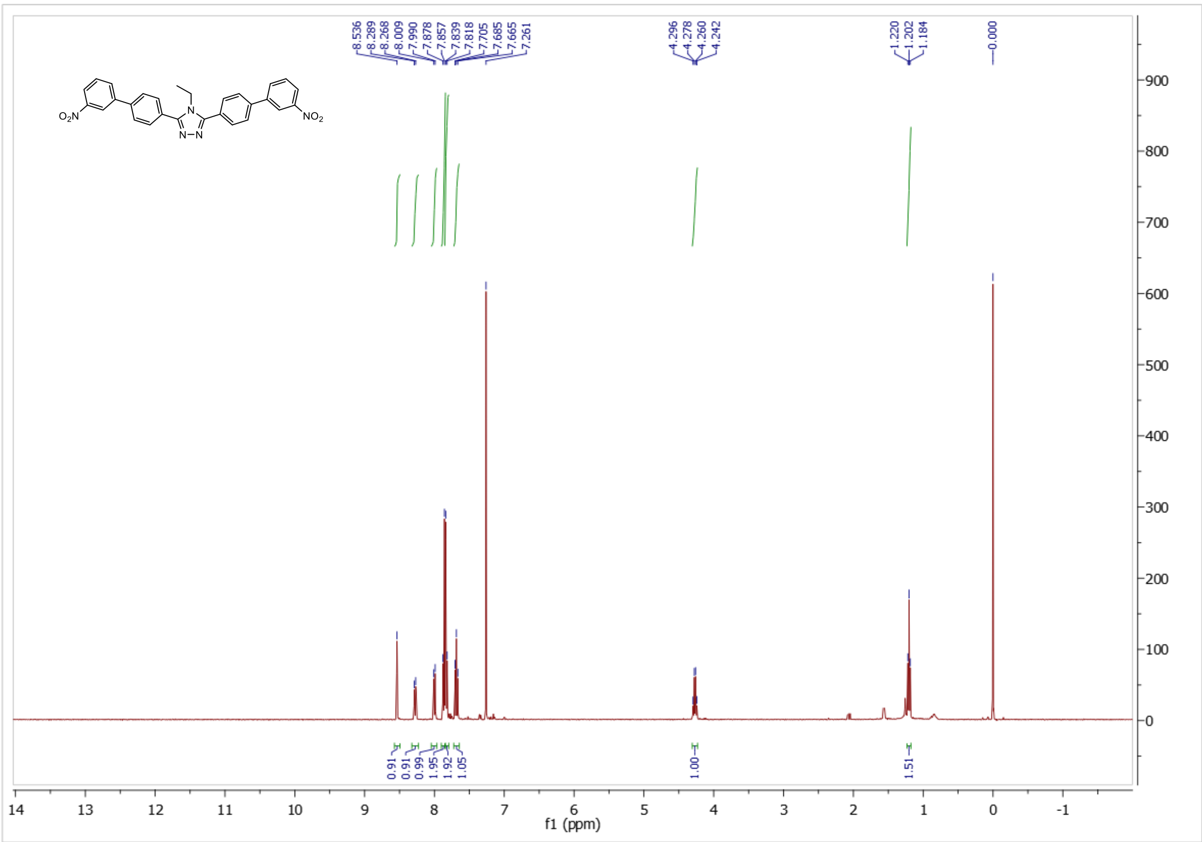

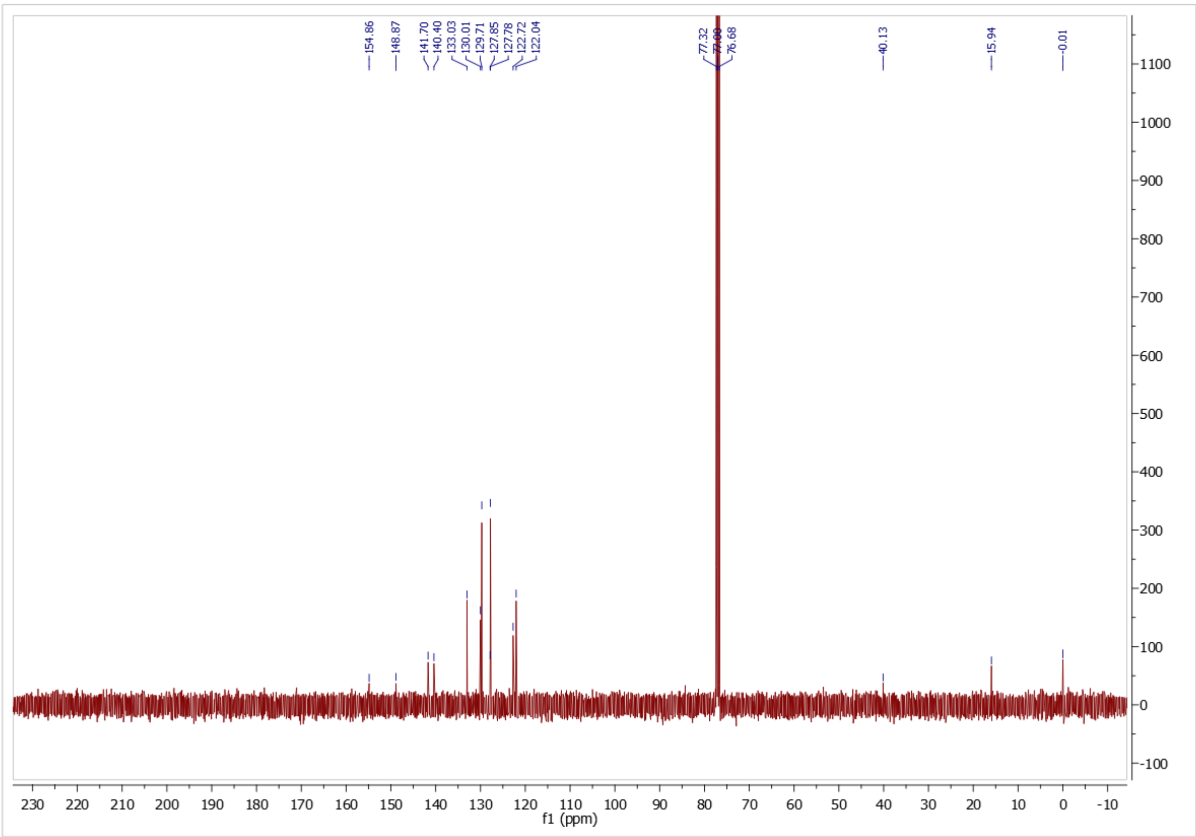

^{1}H and ^{13}C NMR of 3,5-bis(3'-aminobiphenyl-4-yl)-4-ethyl-4*H*-1,2,4-triazole **(5h)**

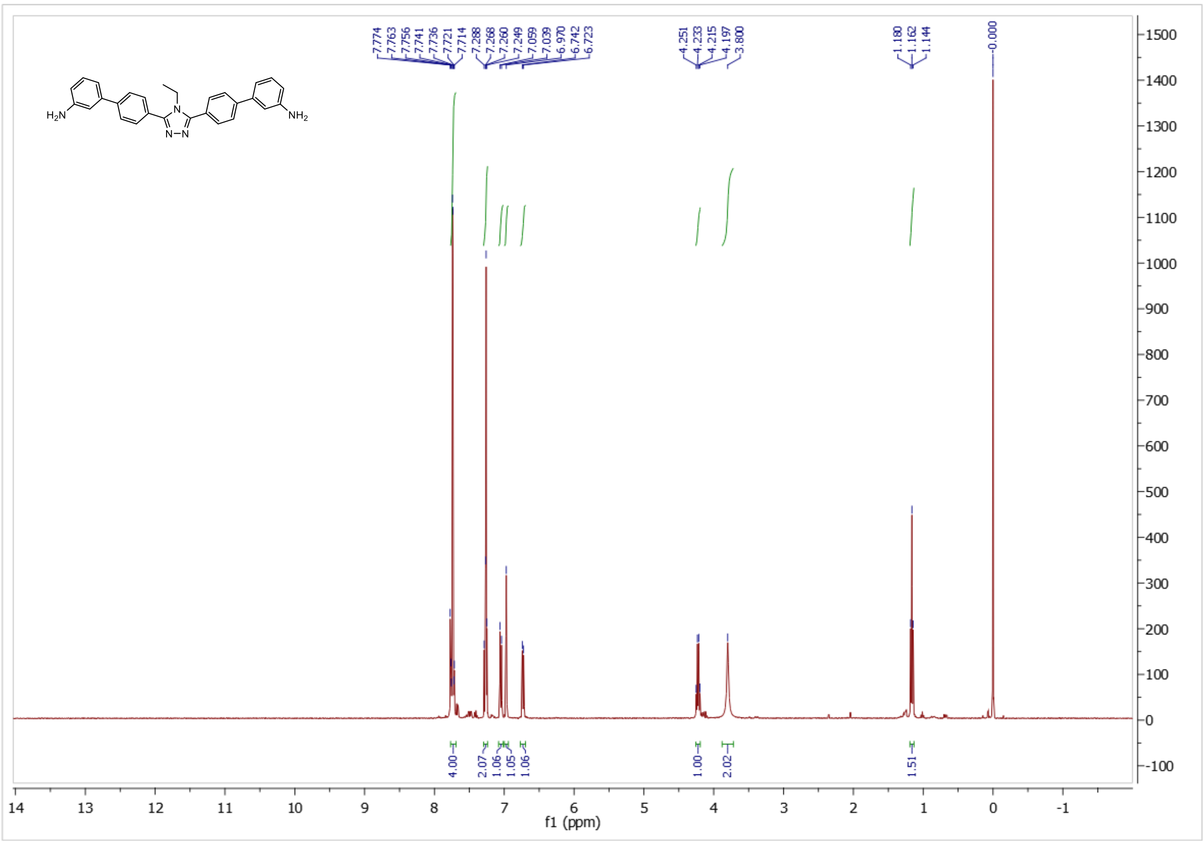

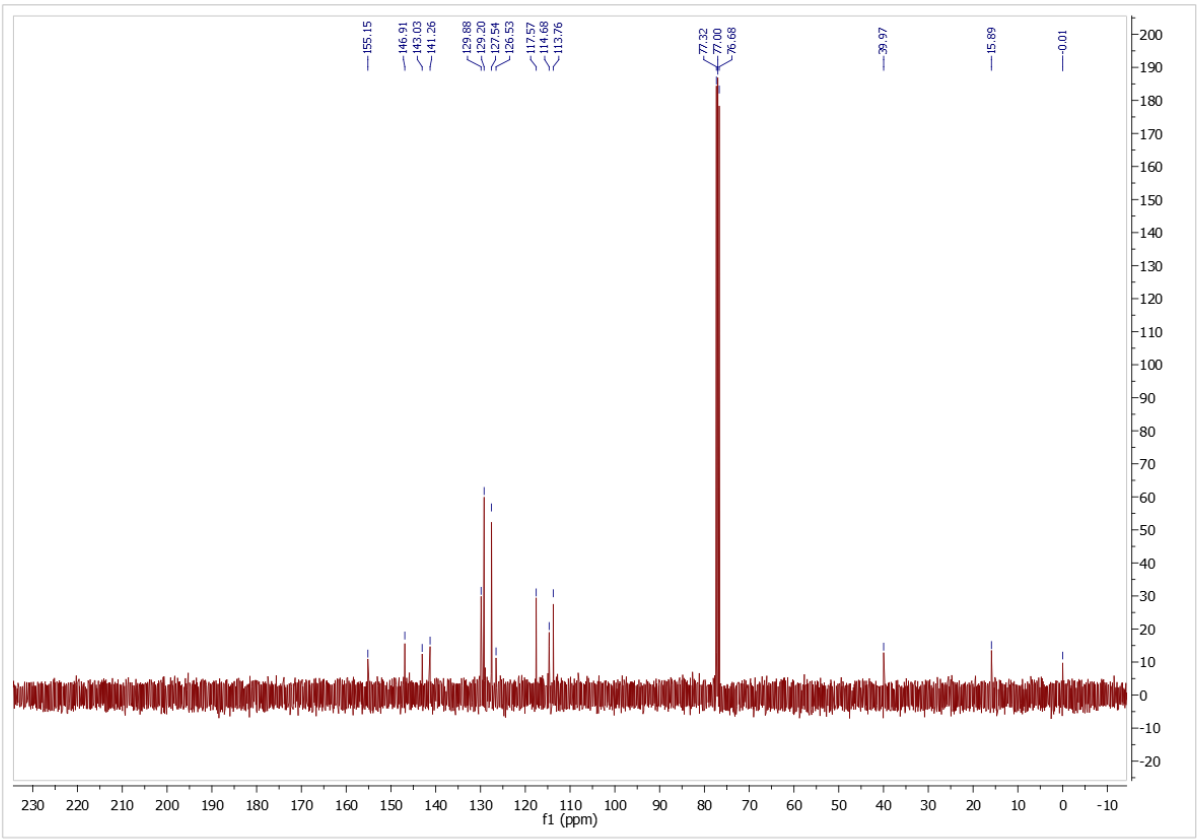

^{1}H and ^{13}C NMR of 4-ethyl-3,5-bis[4-(pyridin-4-yl)phenyl]-4*H*-1,2,4-triazole **(5i)**

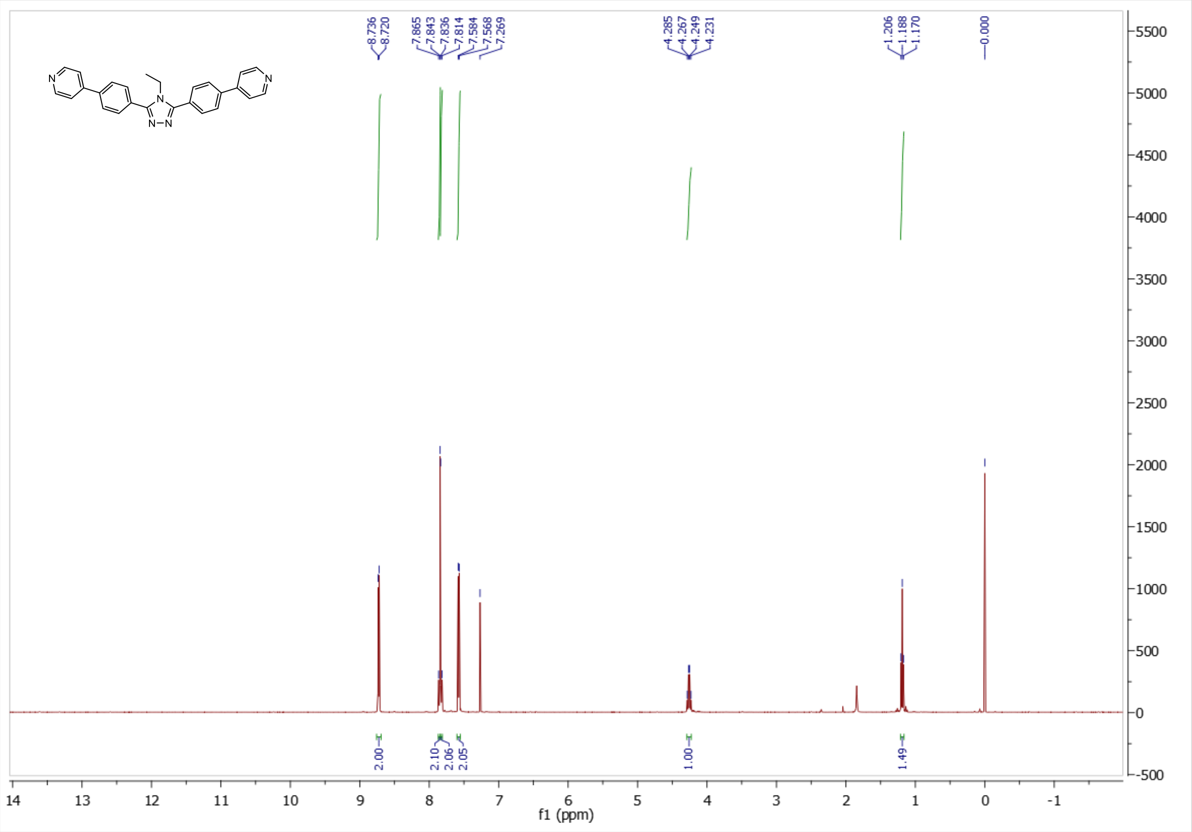

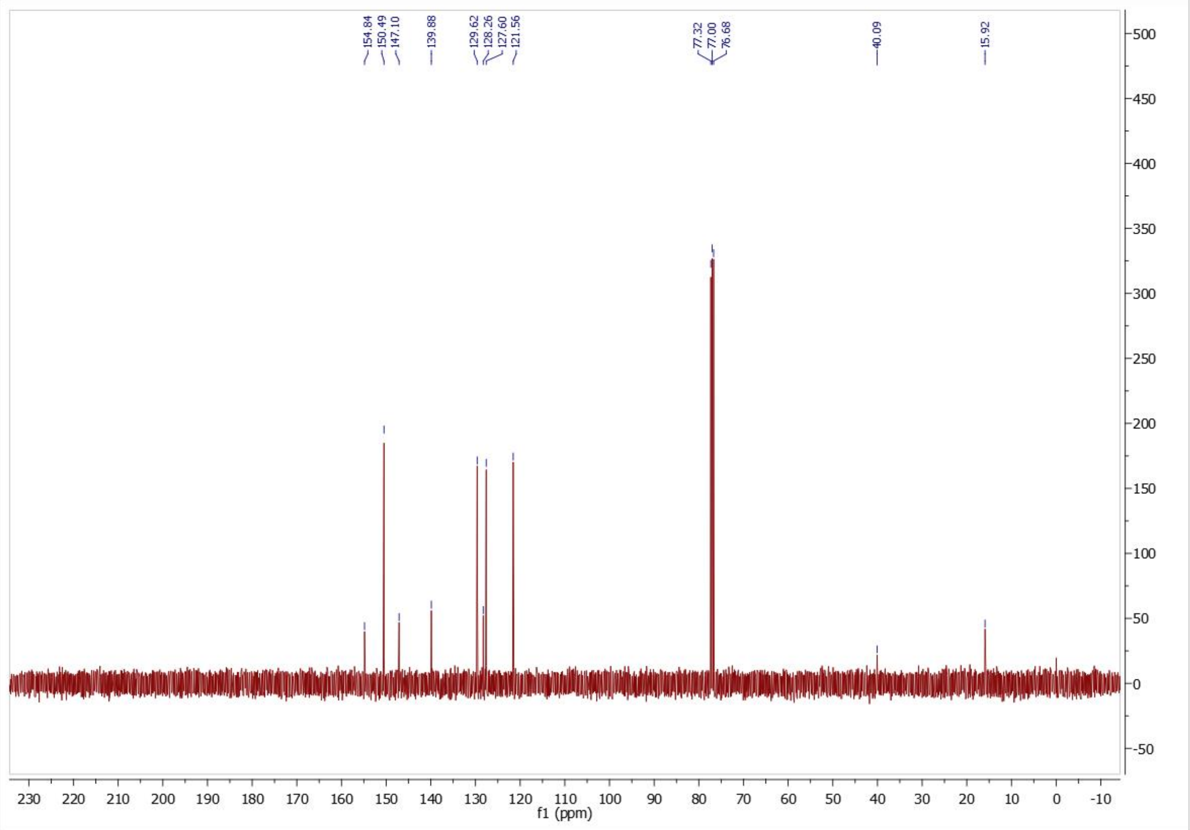

^{1}H and ^{13}C NMR of 4-ethyl-3,5-bis[4-(pyridin-3-yl)phenyl]-4*H*-1,2,4-triazole **(5j)**

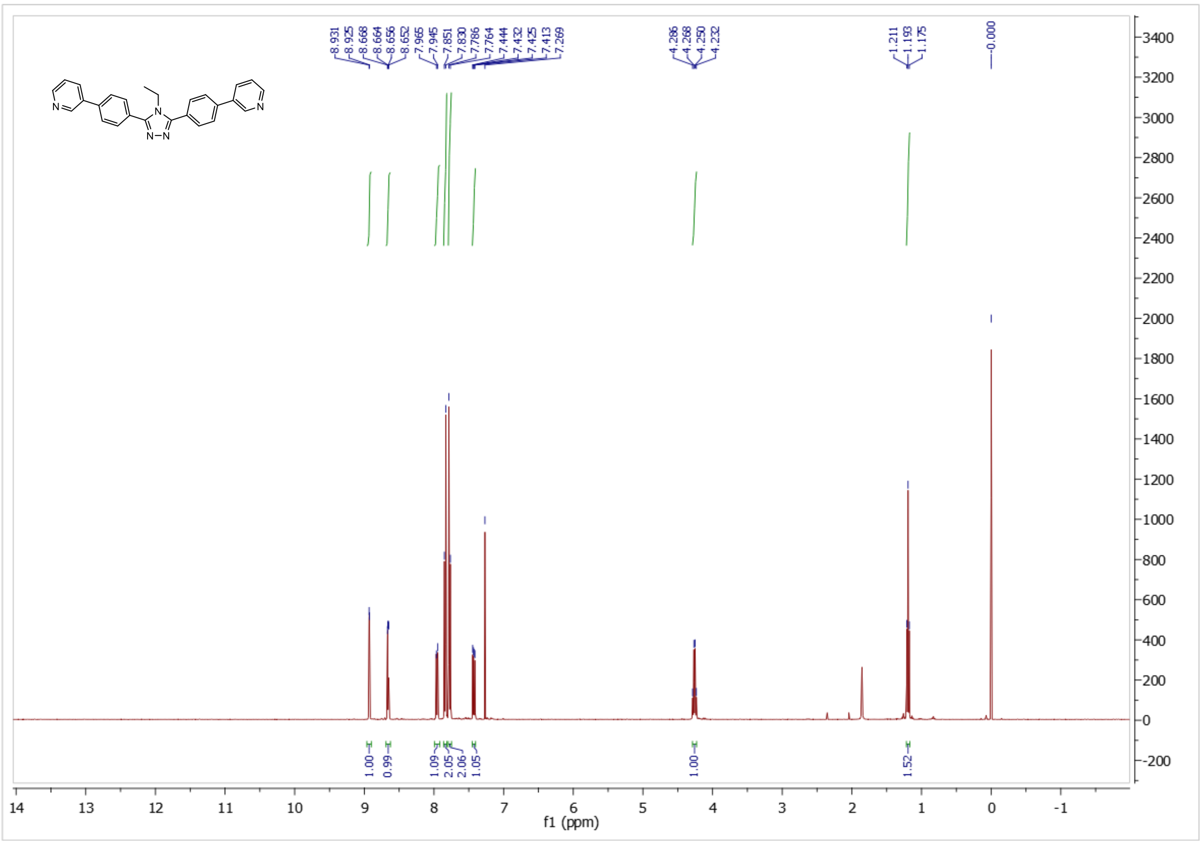

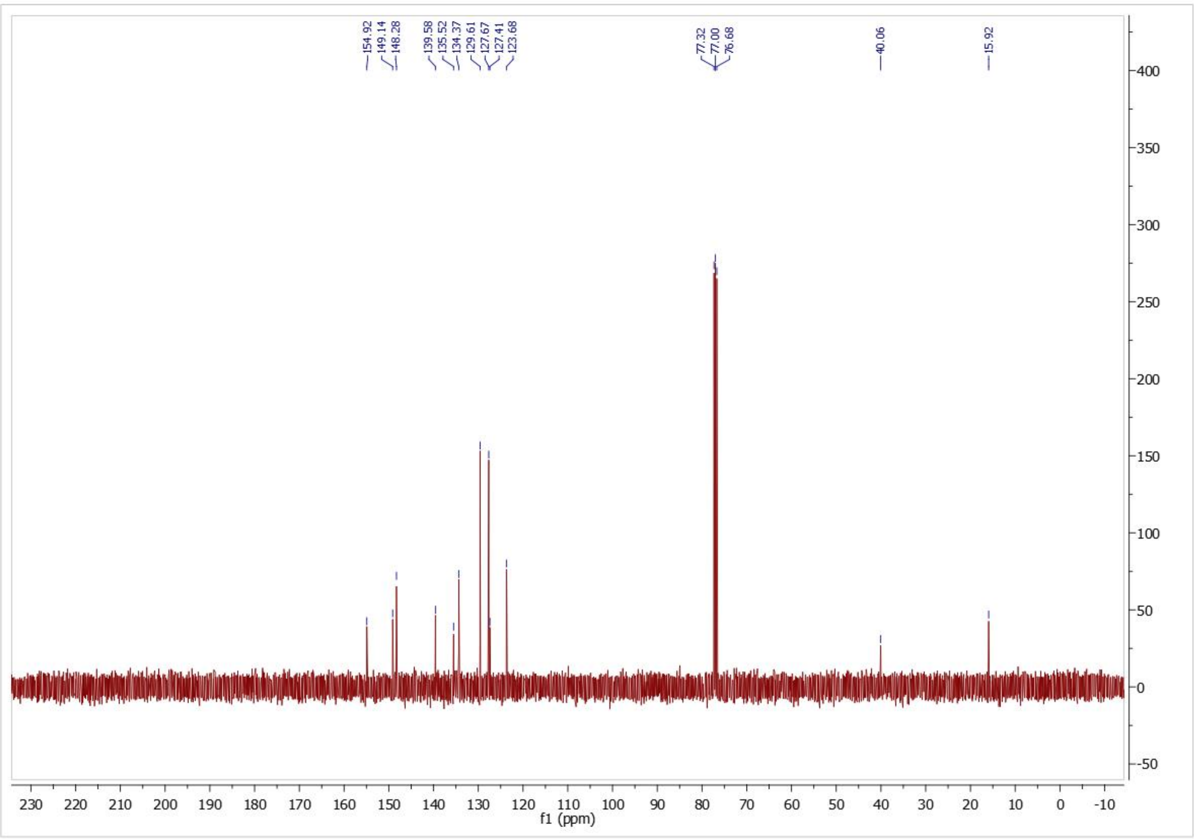

^{1}H and ^{13}C NMR of 4-ethyl-3,5-bis[4-(furan-2-yl)phenyl]-4*H*-1,2,4-triazole **(5k)**

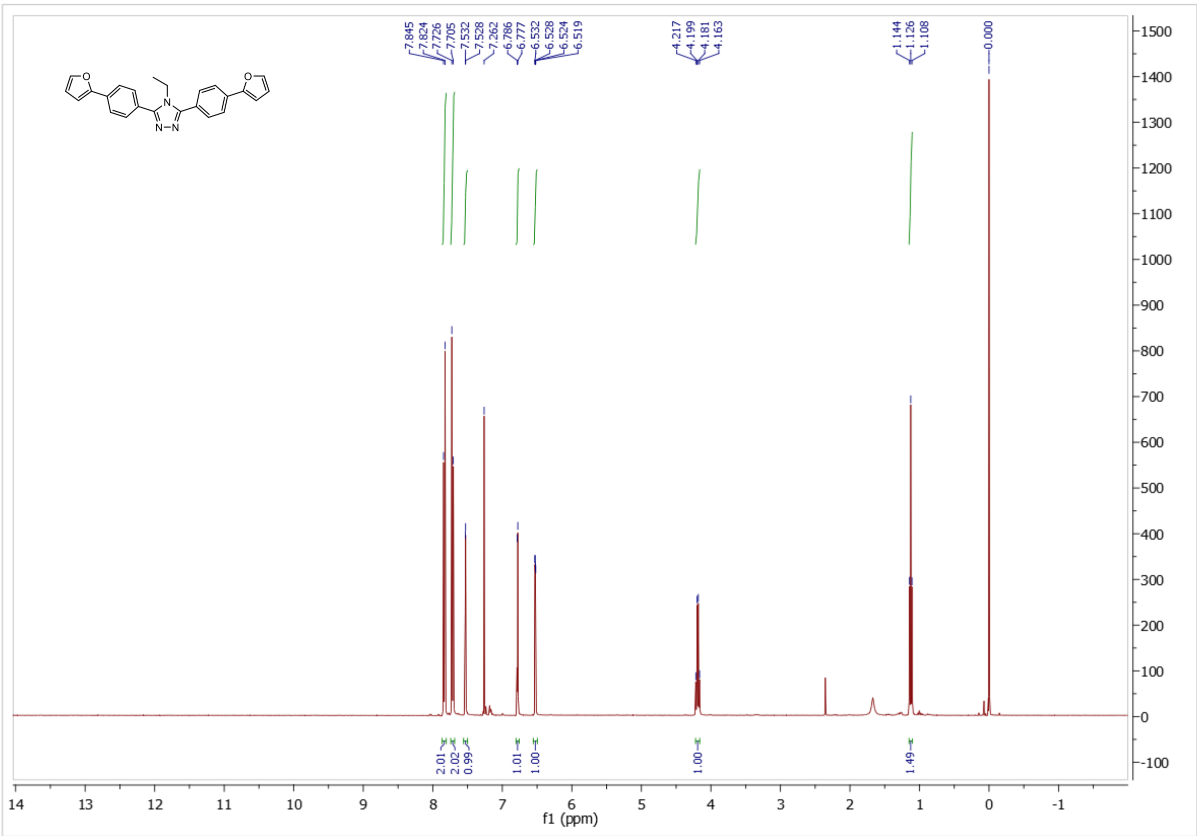

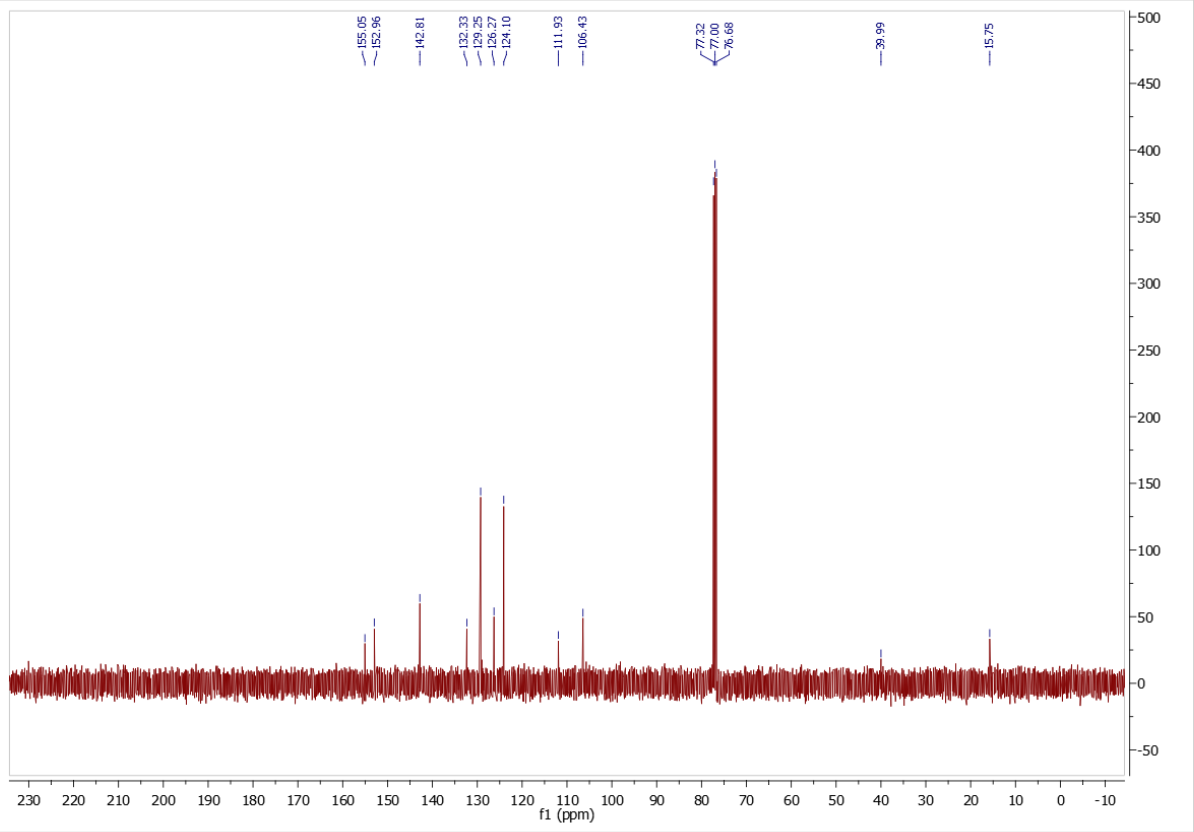

^{1}H and ^{13}C NMR of 4-ethyl-3,5-bis[4-(furan-3-yl)phenyl]-4*H*-1,2,4-triazole **(5l)**

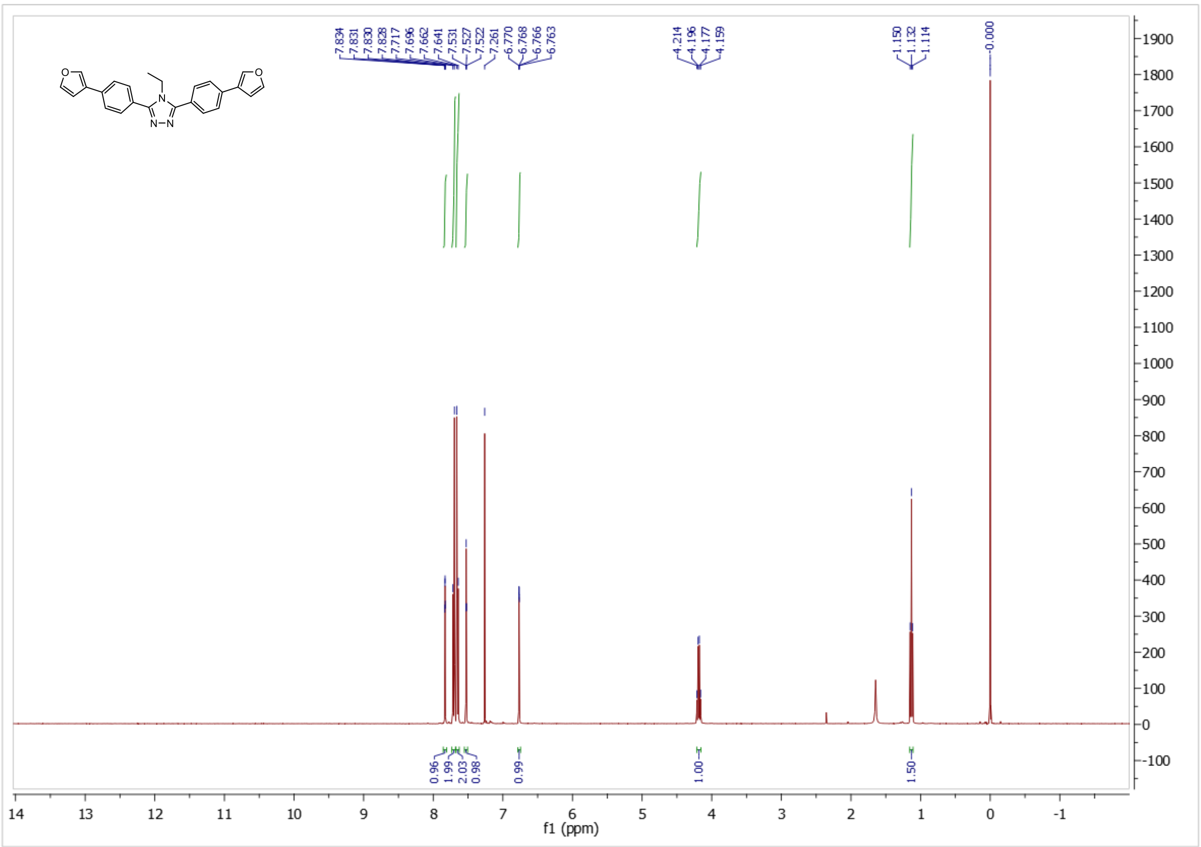

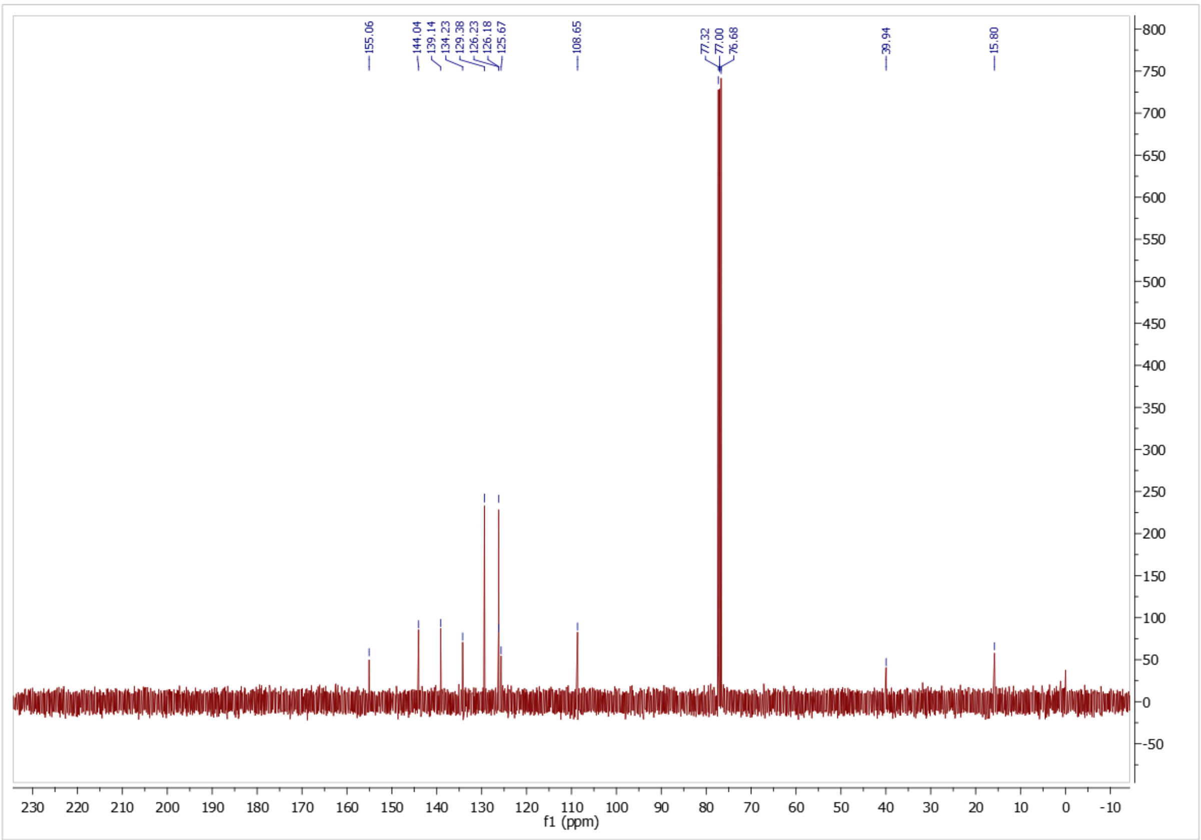

^{1}H and ^{13}C NMR of 4-ethyl-3,5-bis[4-(thiophen-2-yl)phenyl]-4*H*-1,2,4-triazole **(5m)**

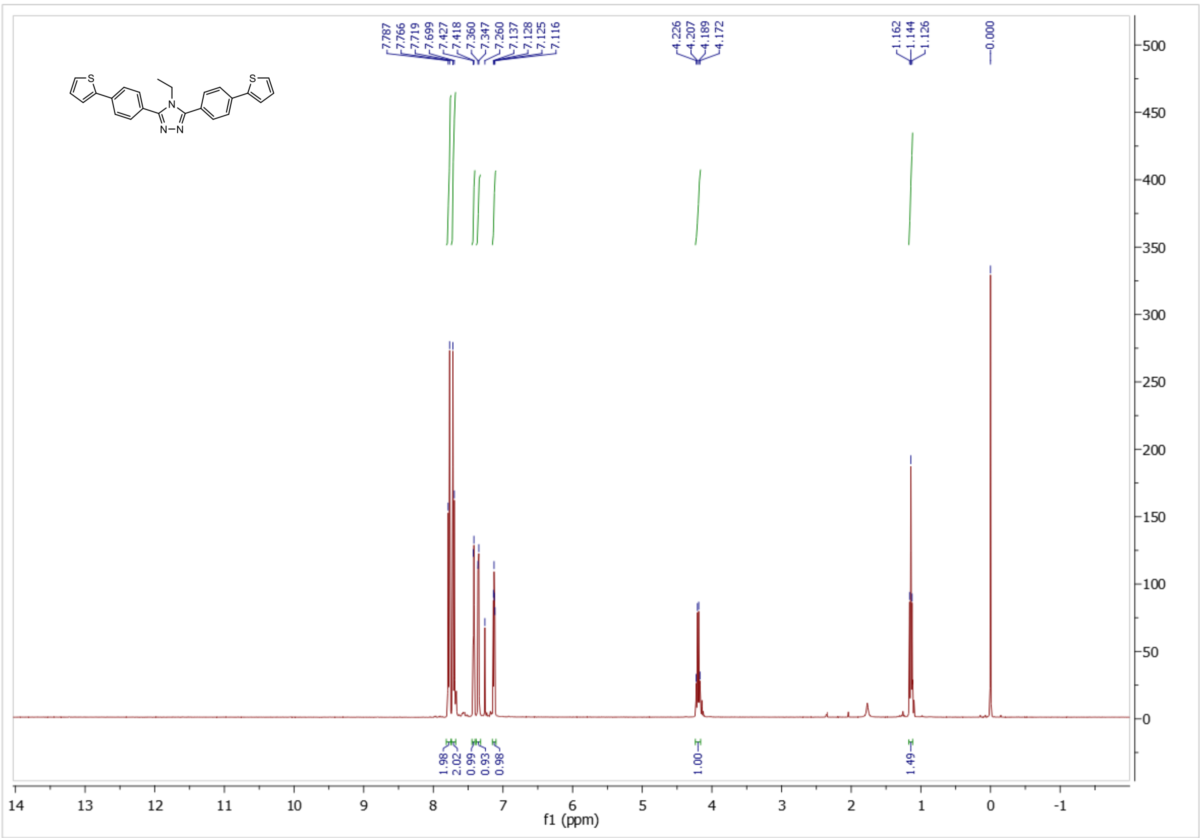

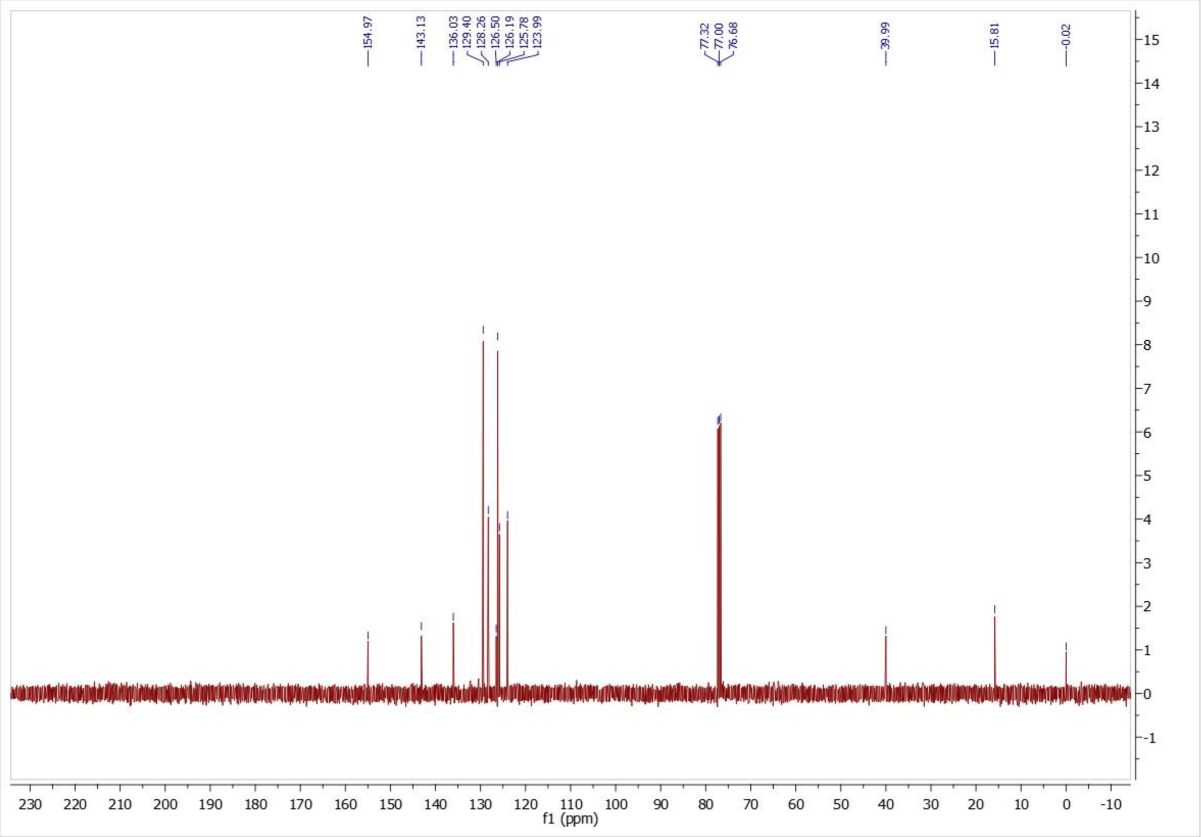

^{1}H and ^{13}C NMR of 4-ethyl-3,5-bis[4-(thiophen-3-yl)phenyl]-4*H*-1,2,4-triazole **(5n)**

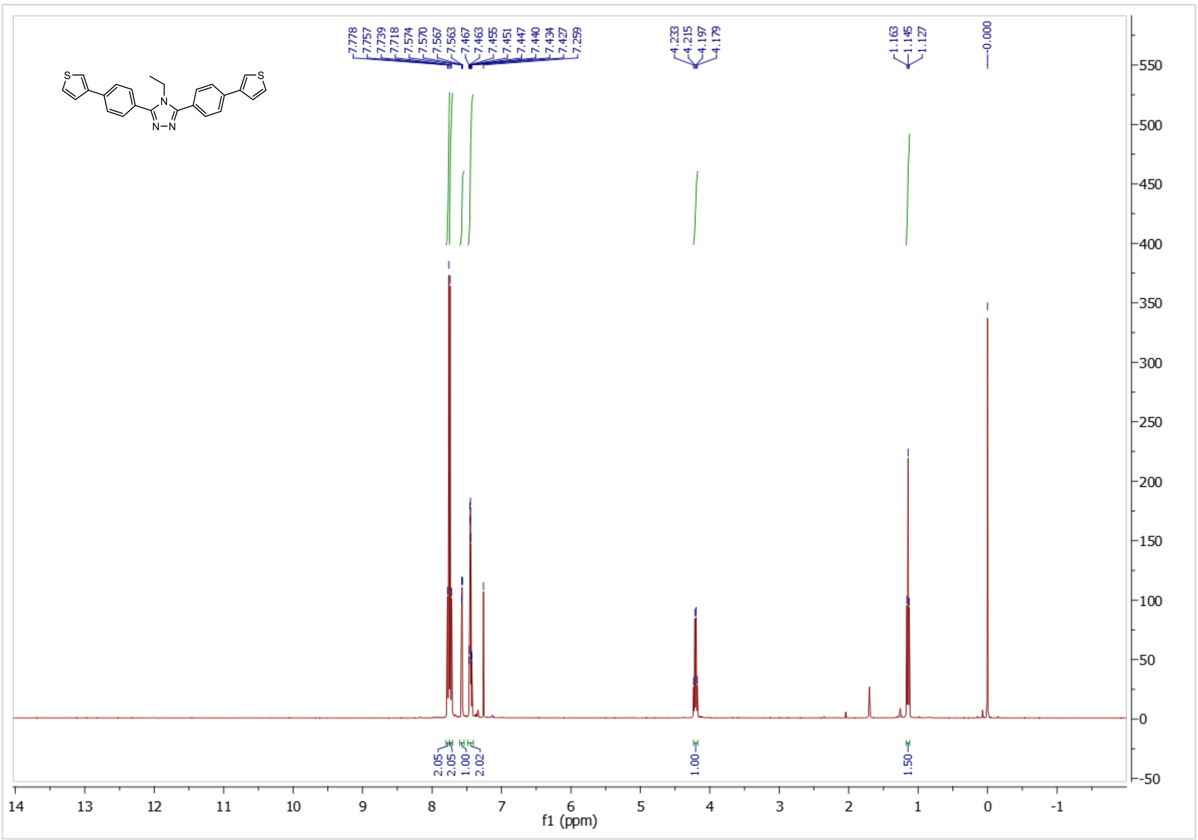

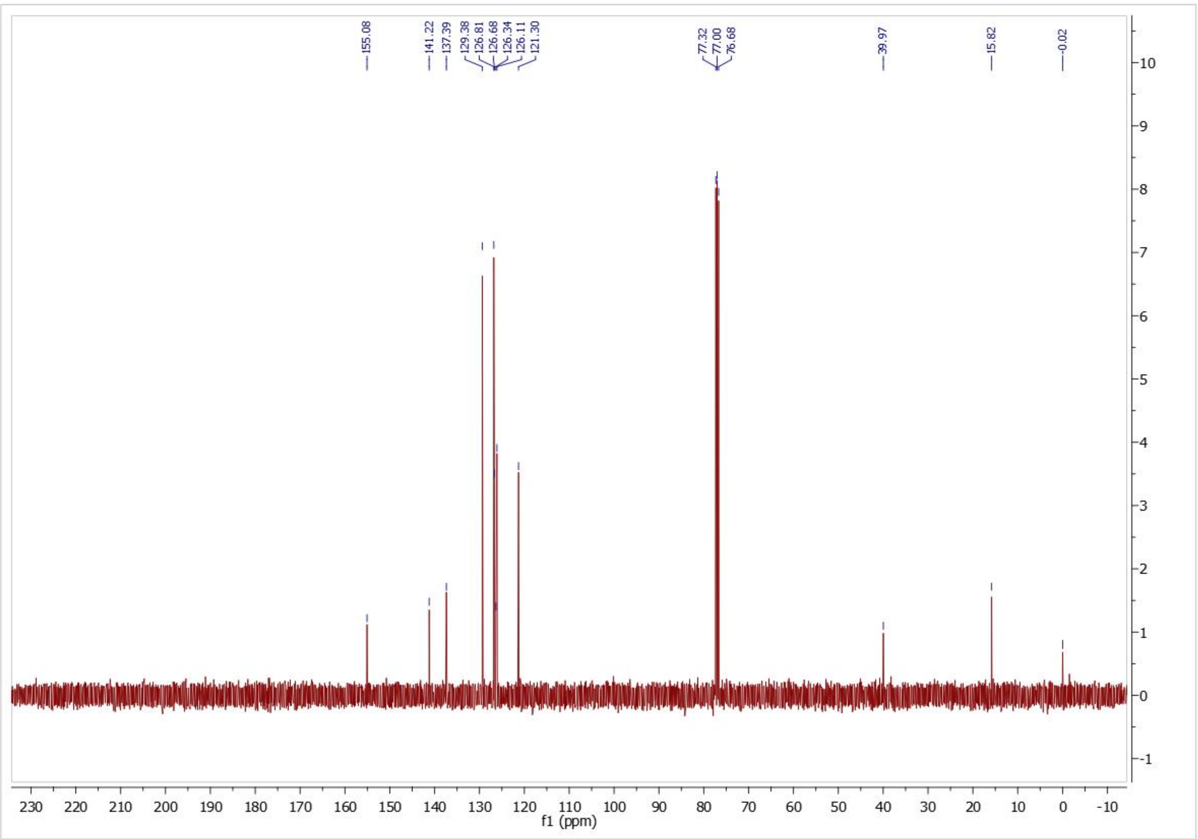

^{1}H and ^{13}C NMR of 3,5-bis(biphenyl-4-yl)-4-propyl-4*H*-1,2,4-triazole **(6a)**

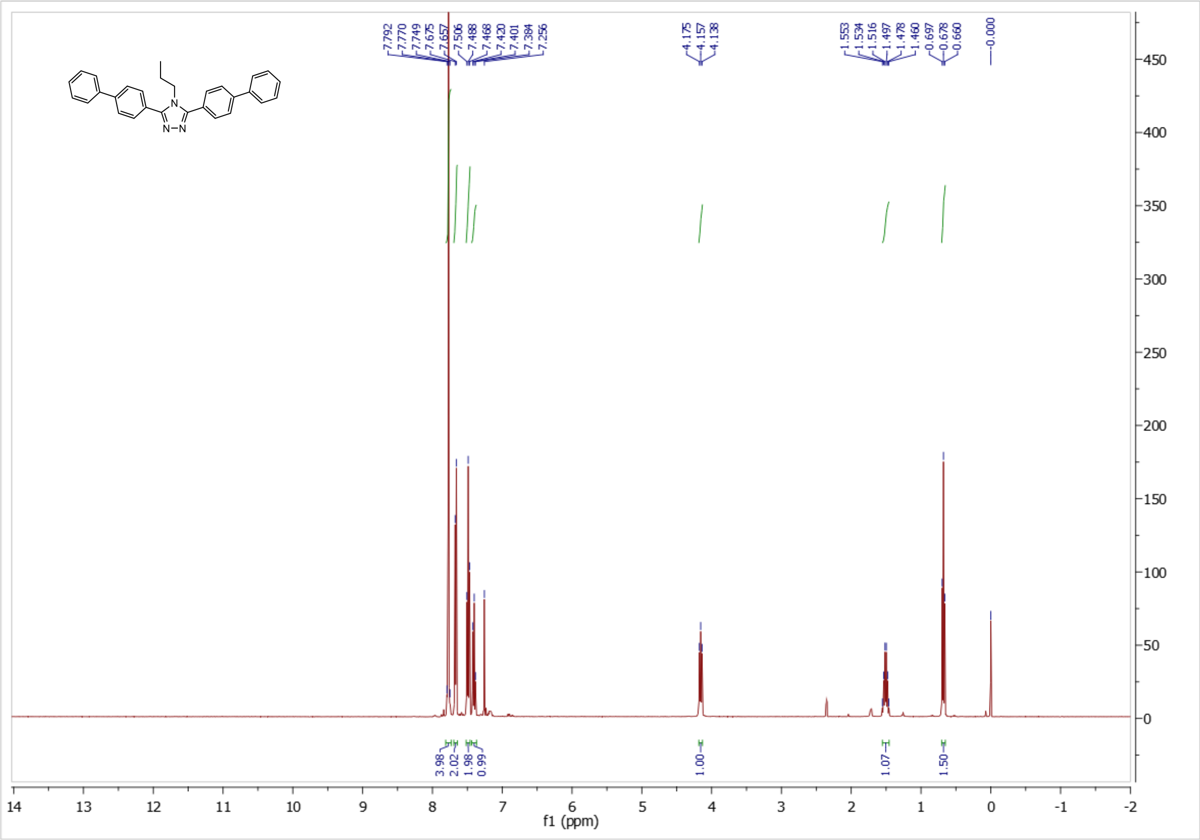

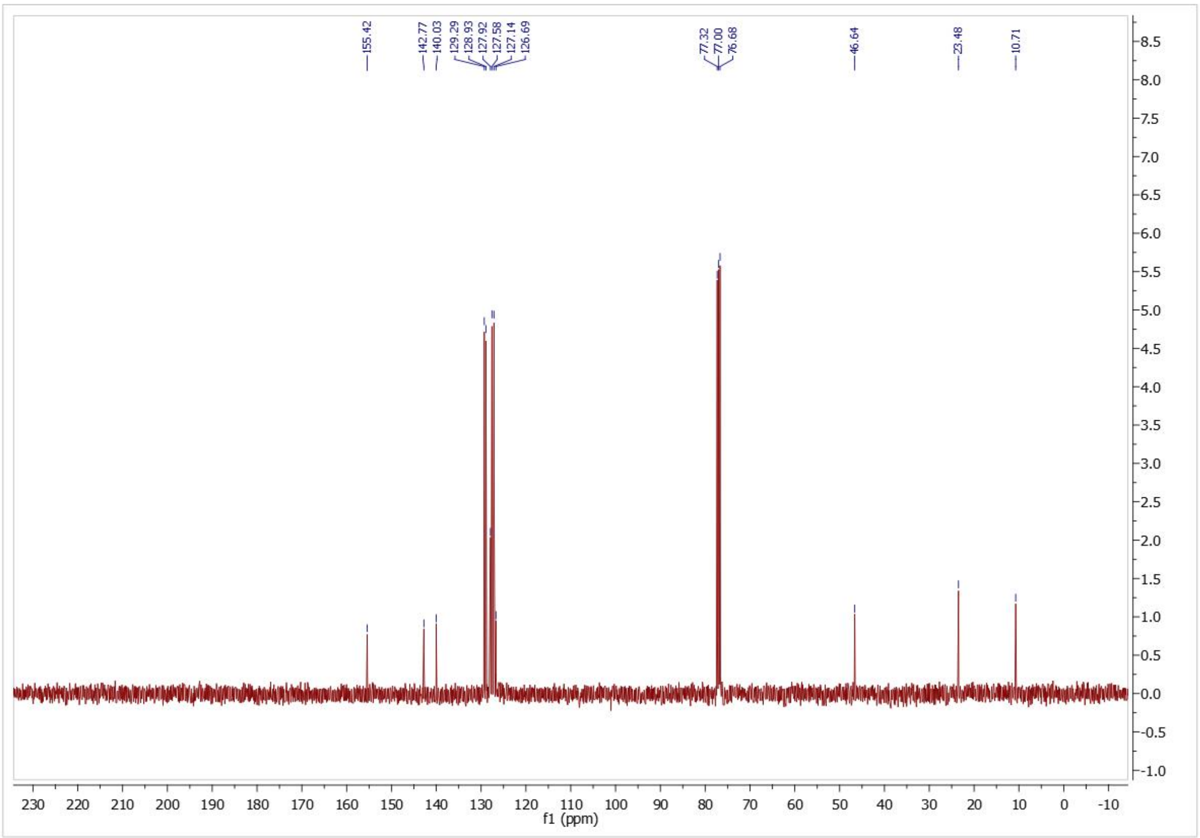

^{1}H and ^{13}C NMR of 3,5-bis(2’-methylbiphenyl-4-yl)-4-propyl-4*H*-1,2,4-triazole **(6b)**

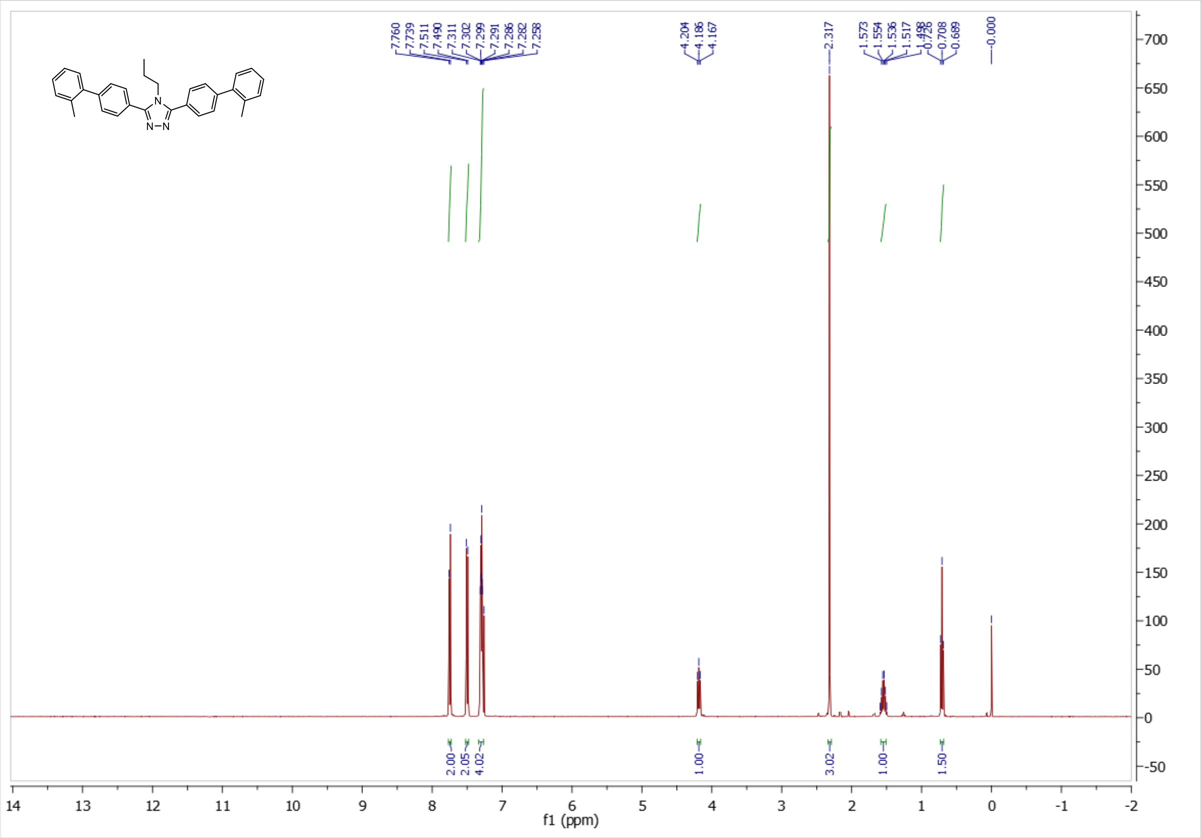

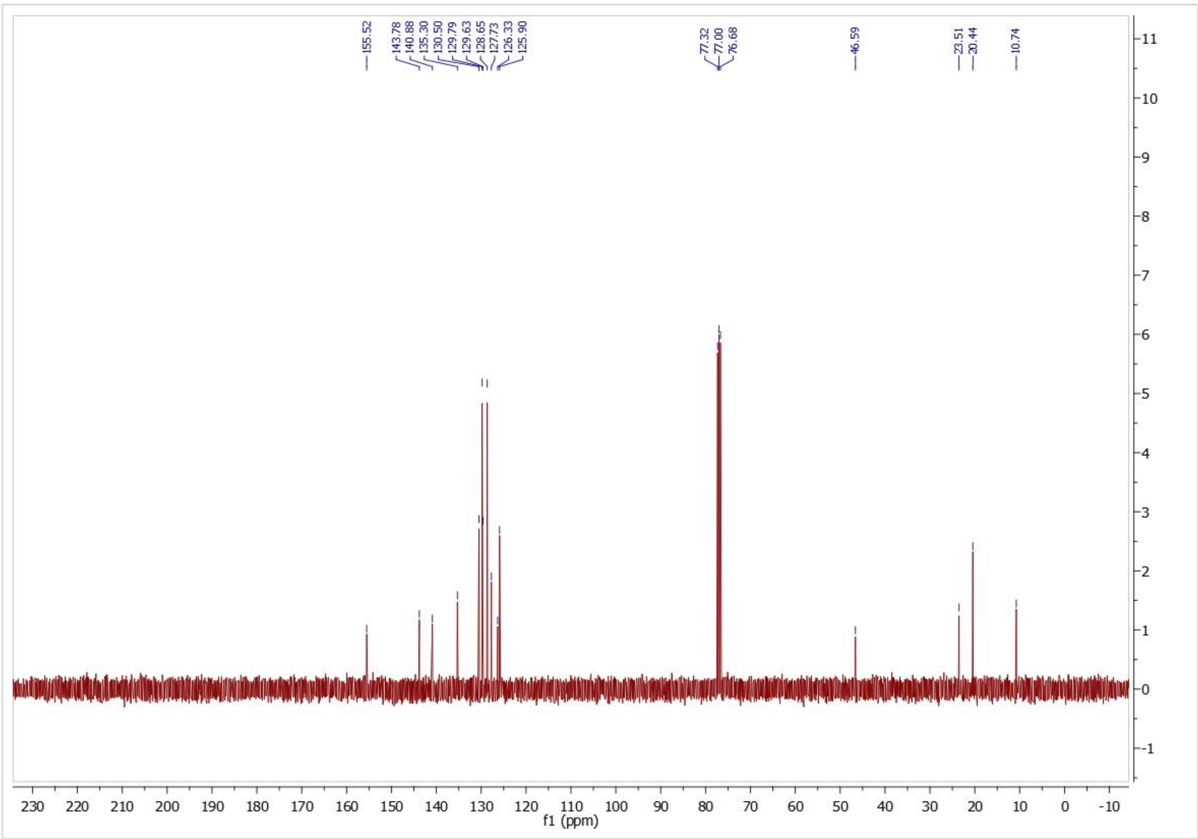

^{1}H and ^{13}C NMR of 3,5-bis(3'-methylbiphenyl-4-yl)-4-propyl-4*H*-1,2,4-triazole **(6c)**

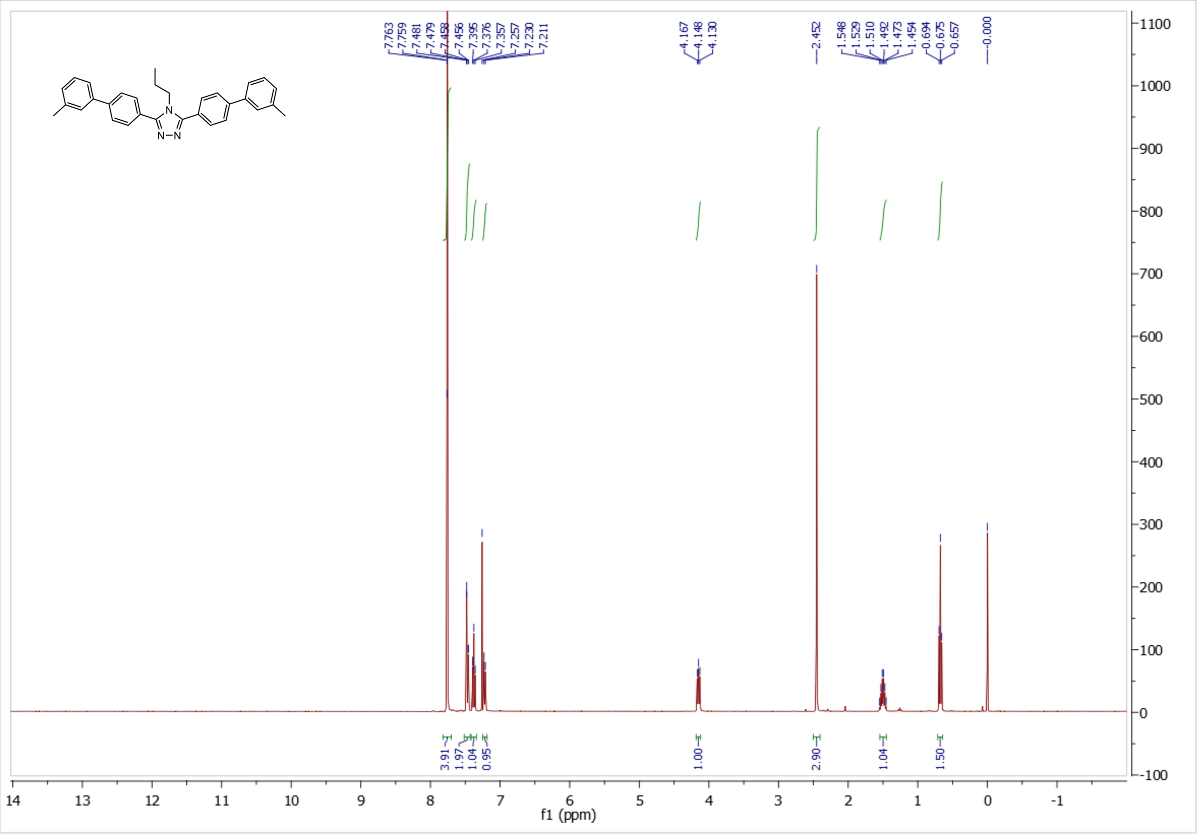

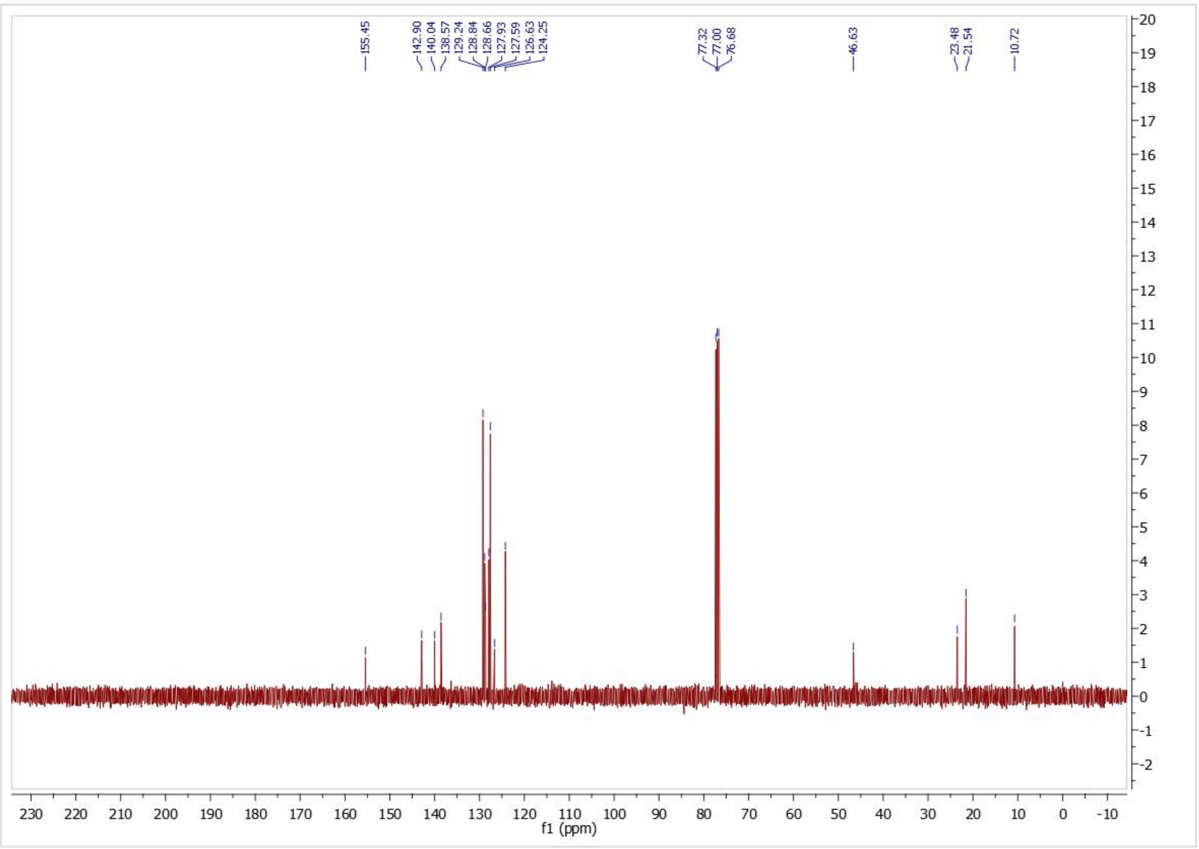

^{1}H and ^{13}C NMR of 3,5-bis(2',6'-dimethylbiphenyl-4-yl)-4-propyl-4*H*-1,2,4-triazole **(6d)**

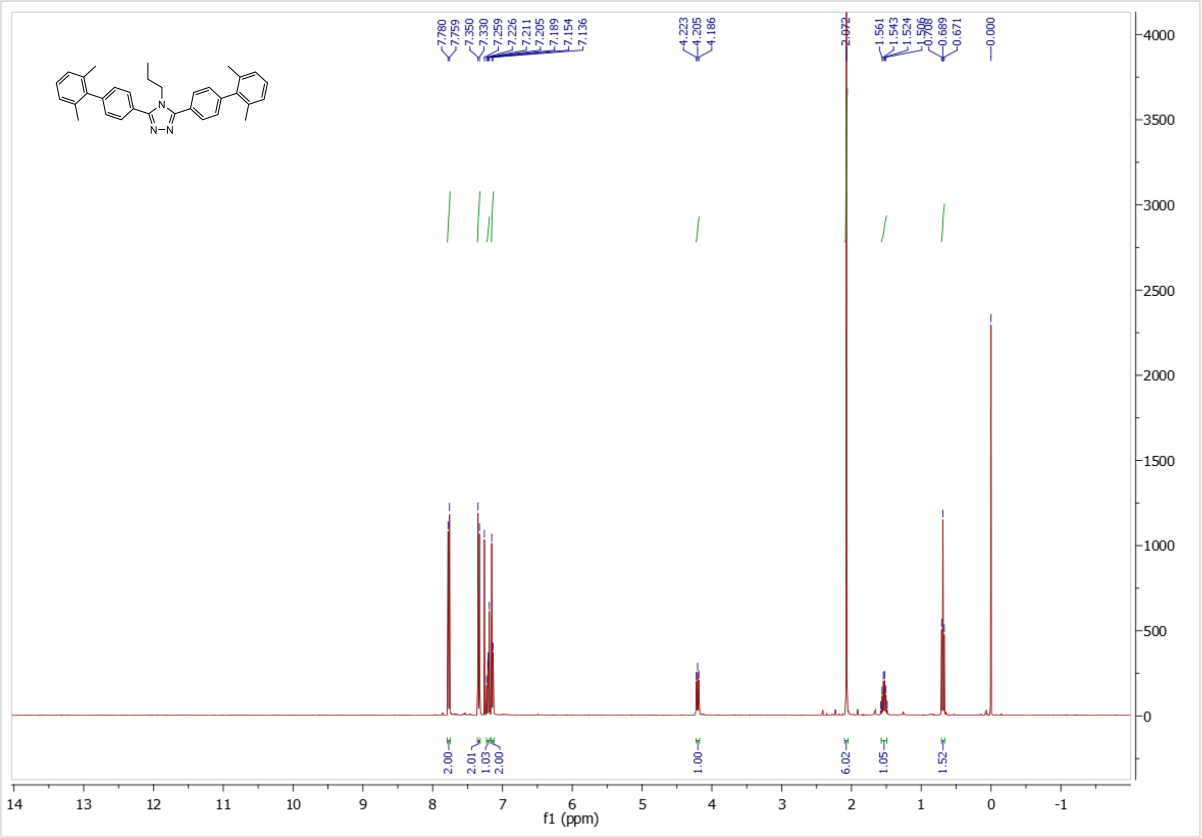

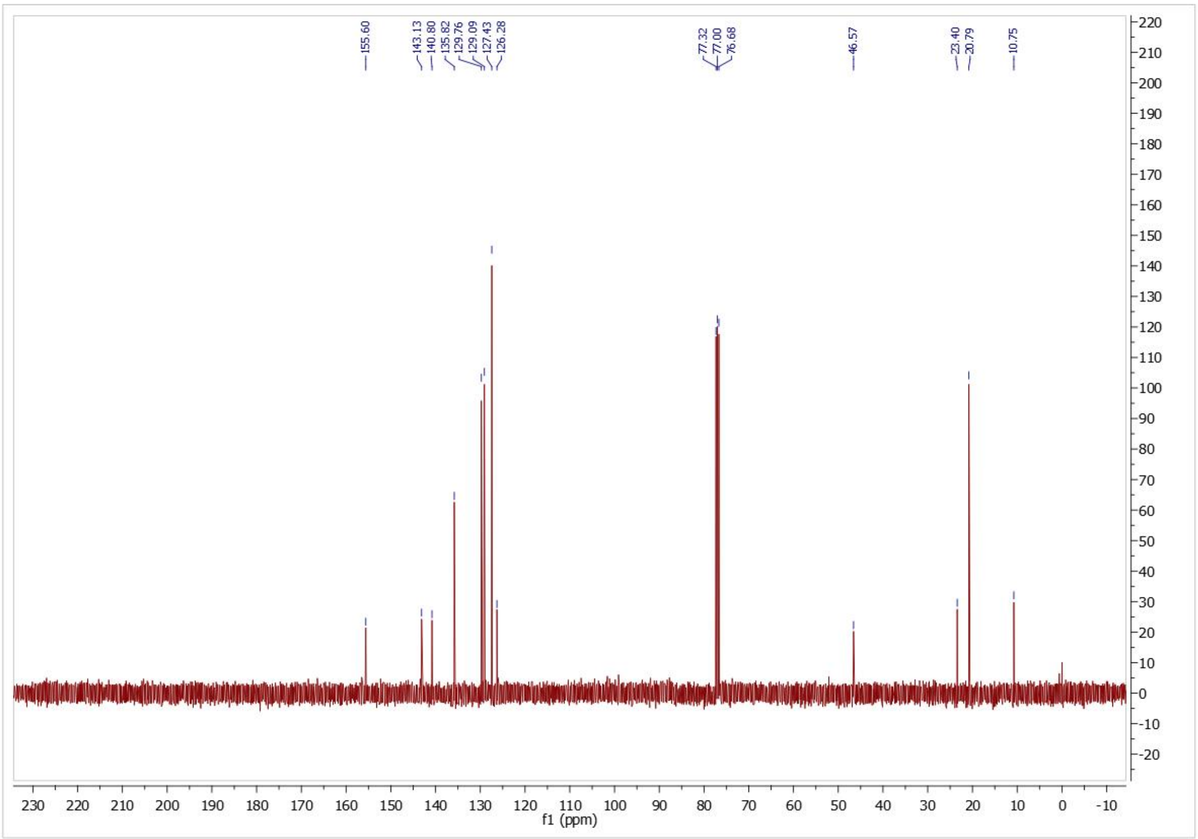

^{1}H and ^{13}C NMR of 3,5-bis(2’-methoxybiphenyl-4-yl)-4-propyl-4*H*-1,2,4-triazole **(6e)**

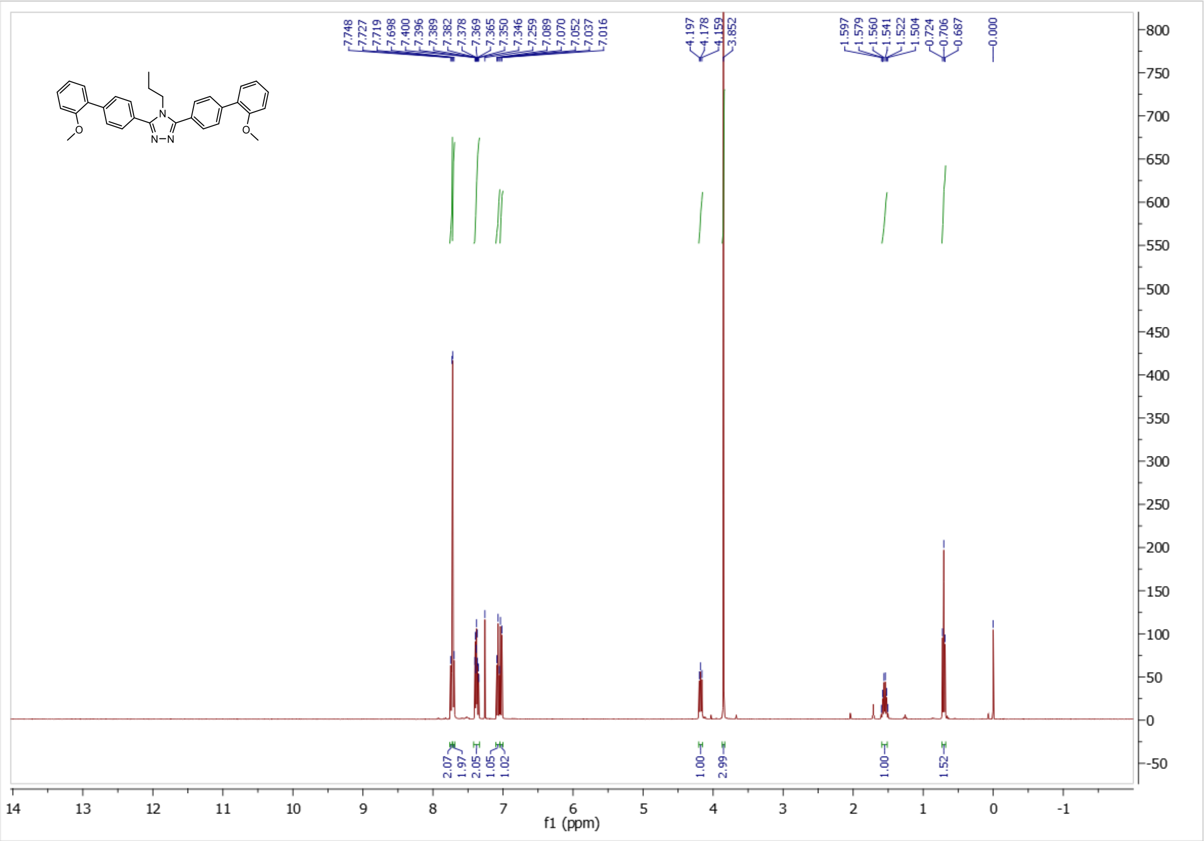

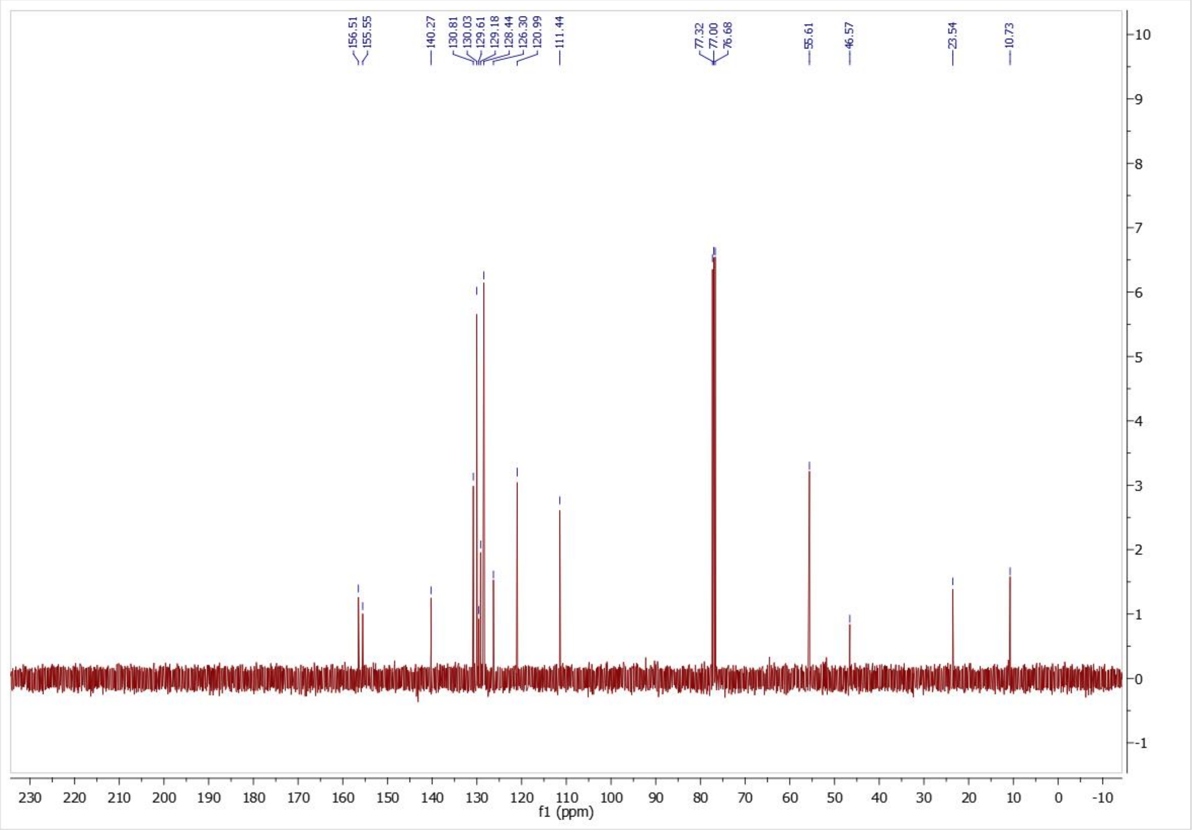

^{1}H and ^{13}C NMR of 3,5-bis(3'-methoxybiphenyl-4-yl)-4-propyl-4*H*-1,2,4-triazole **(6f)**

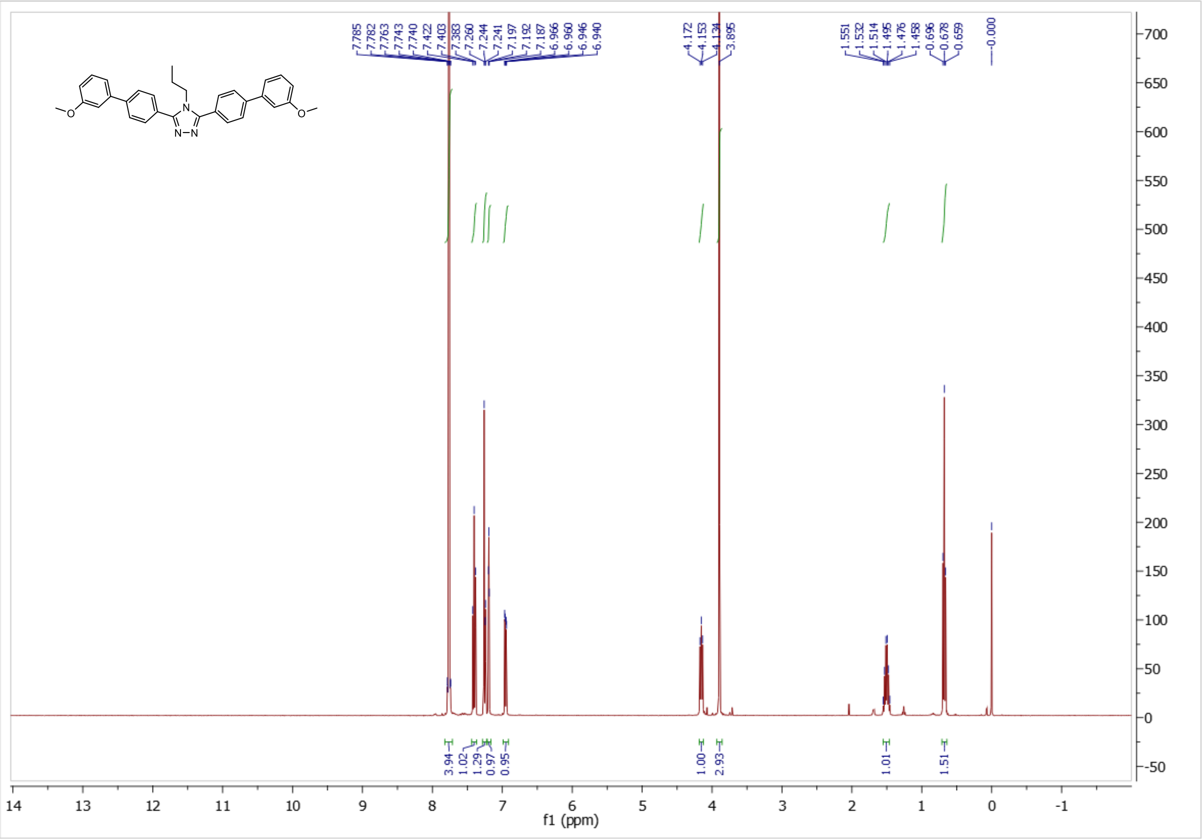

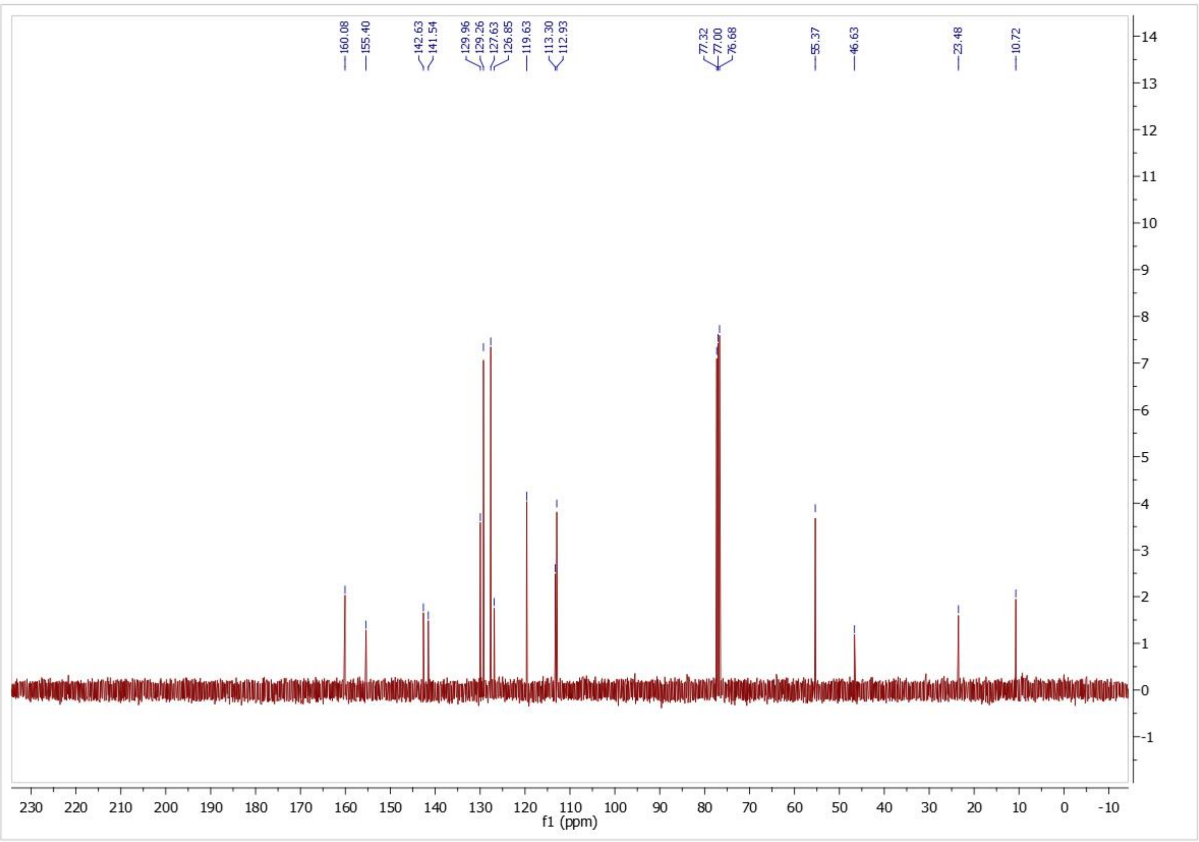

^{1}H and ^{13}C NMR of 3,5-bis(3'-nitrobiphenyl-4-yl)-4-propyl-4*H*-1,2,4-triazole **(6g)**

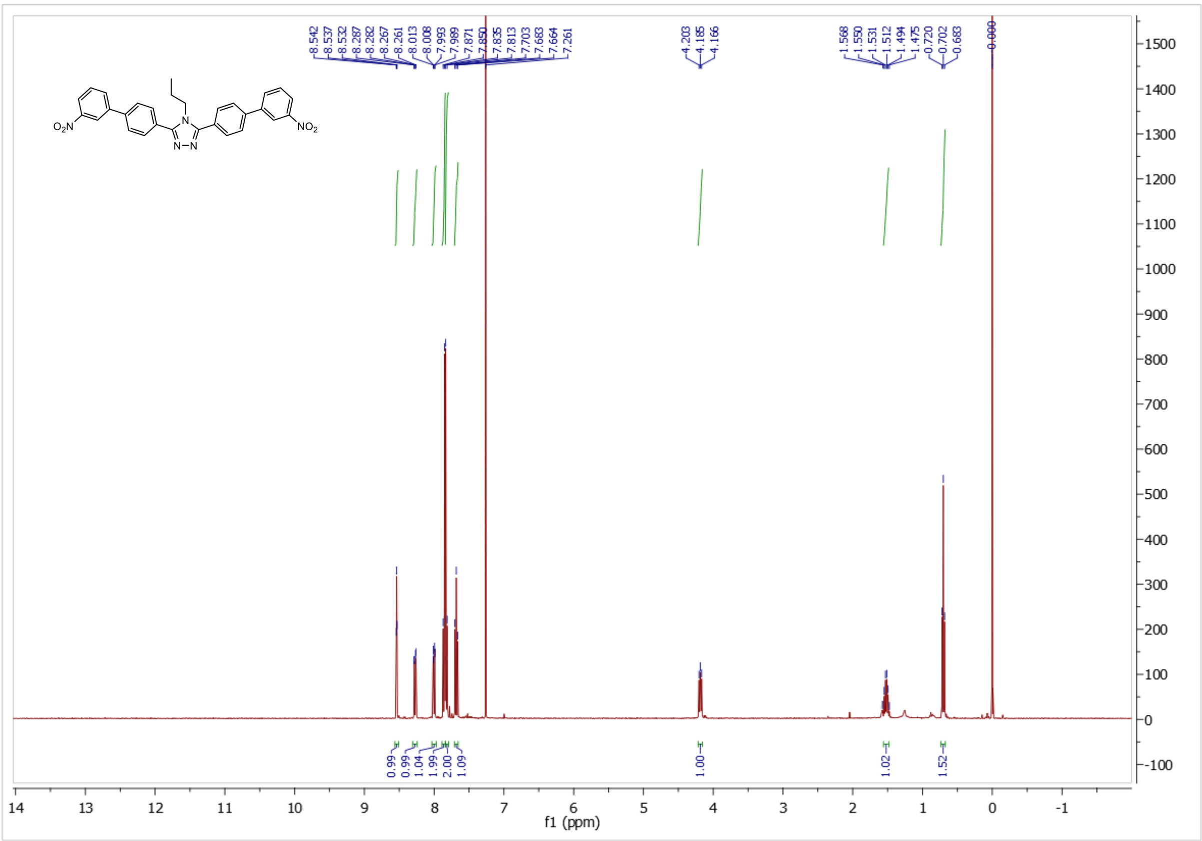

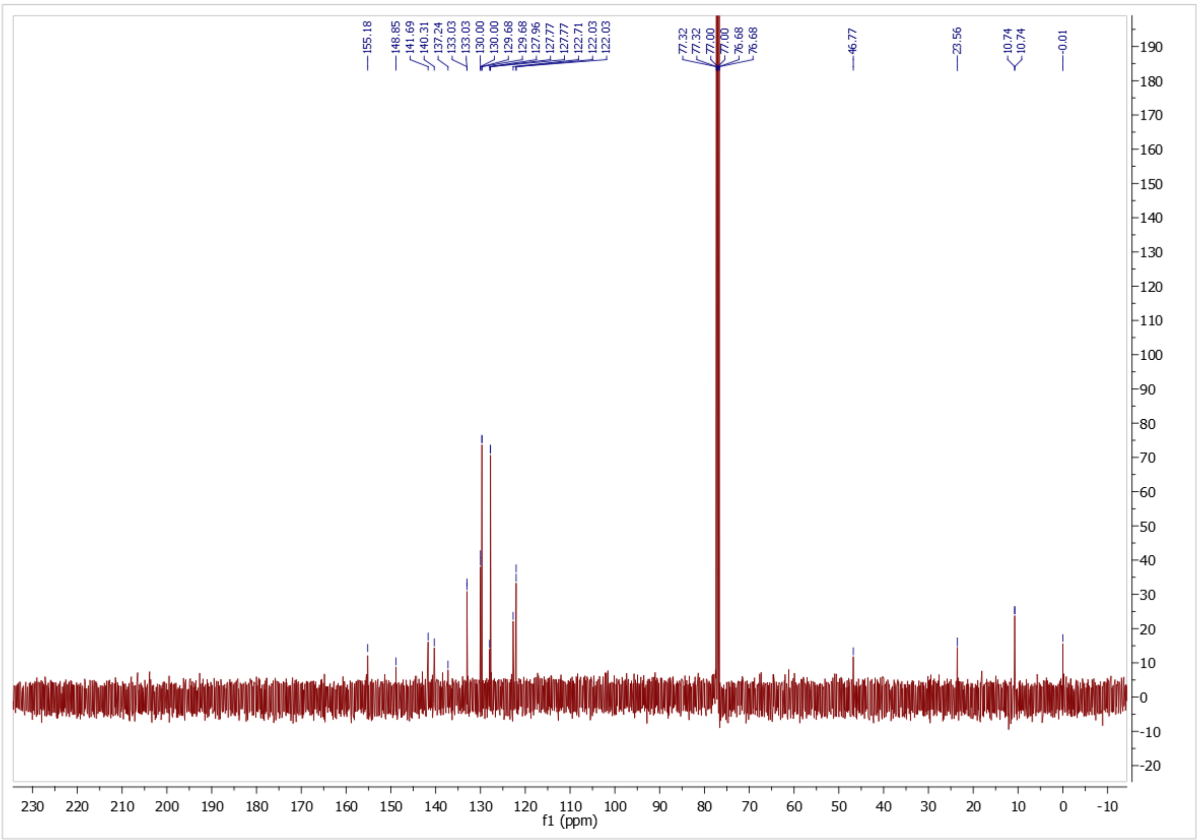

^{1}H and ^{13}C NMR of 3,5-bis(3’-aminobiphenyl-4-yl)-4-propyl-4*H*-1,2,4-triazole **(6h)**

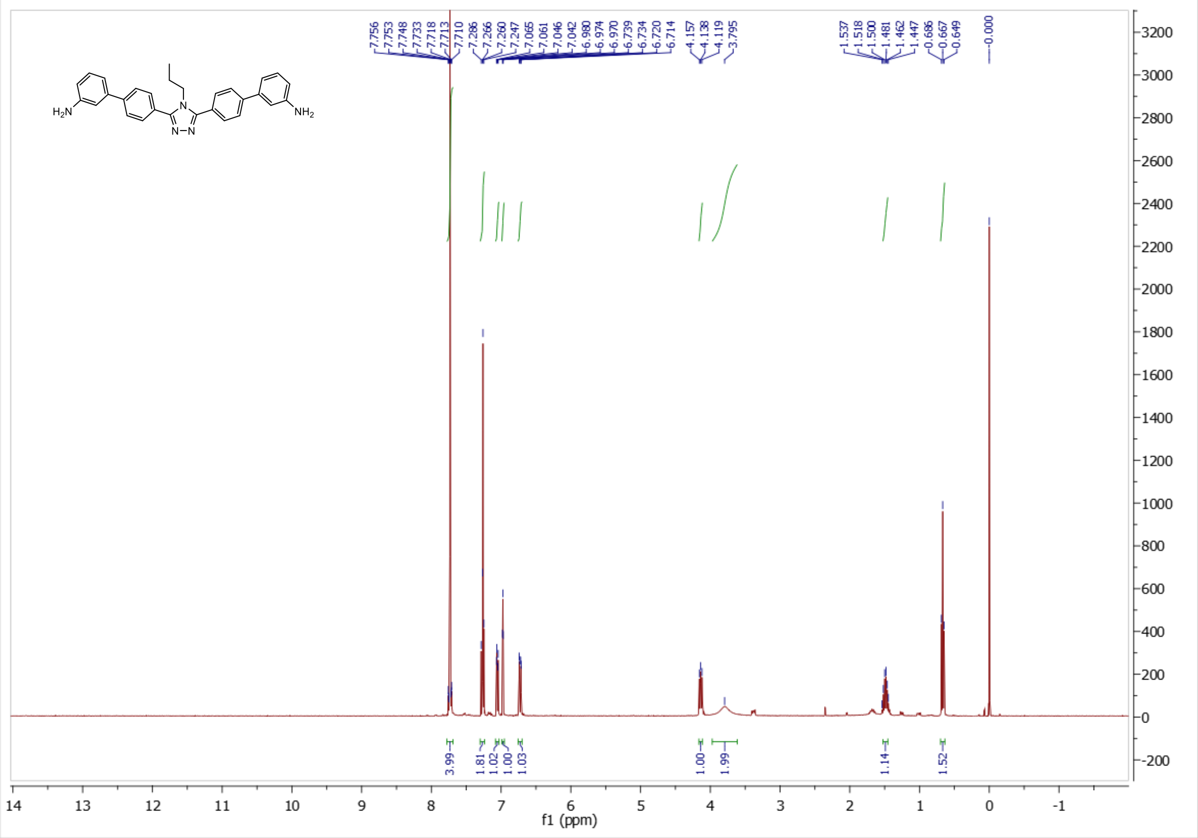

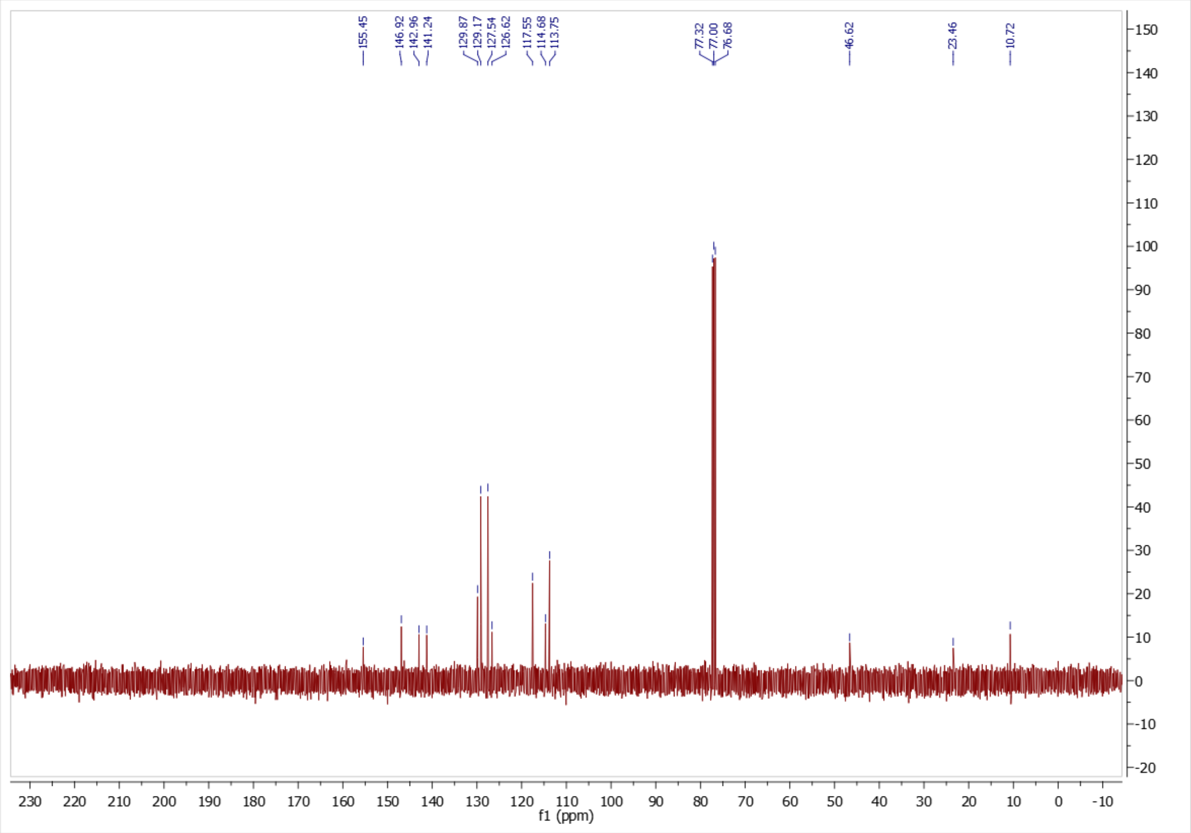

^{1}H and ^{13}C NMR of 4-propyl-3,5-bis[4-(pyridin-4-yl)phenyl]-4*H*-1,2,4-triazole **(6i)**

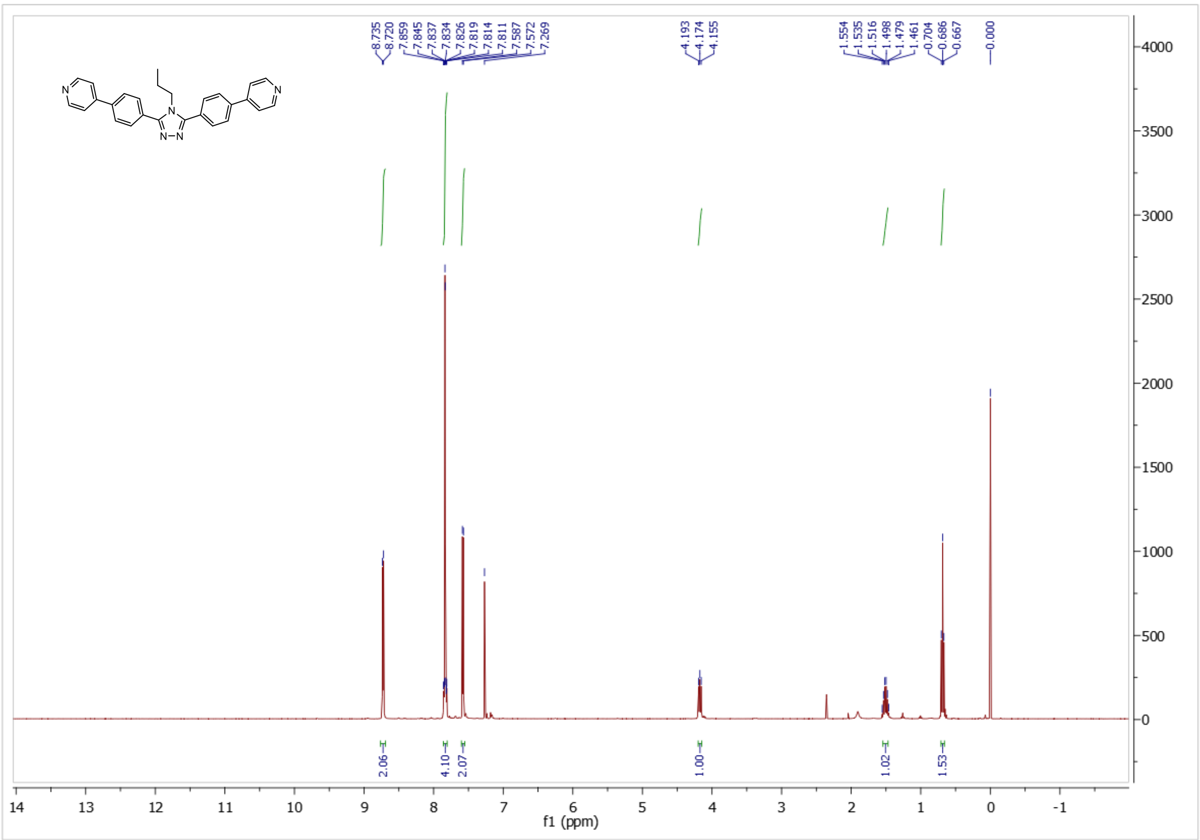

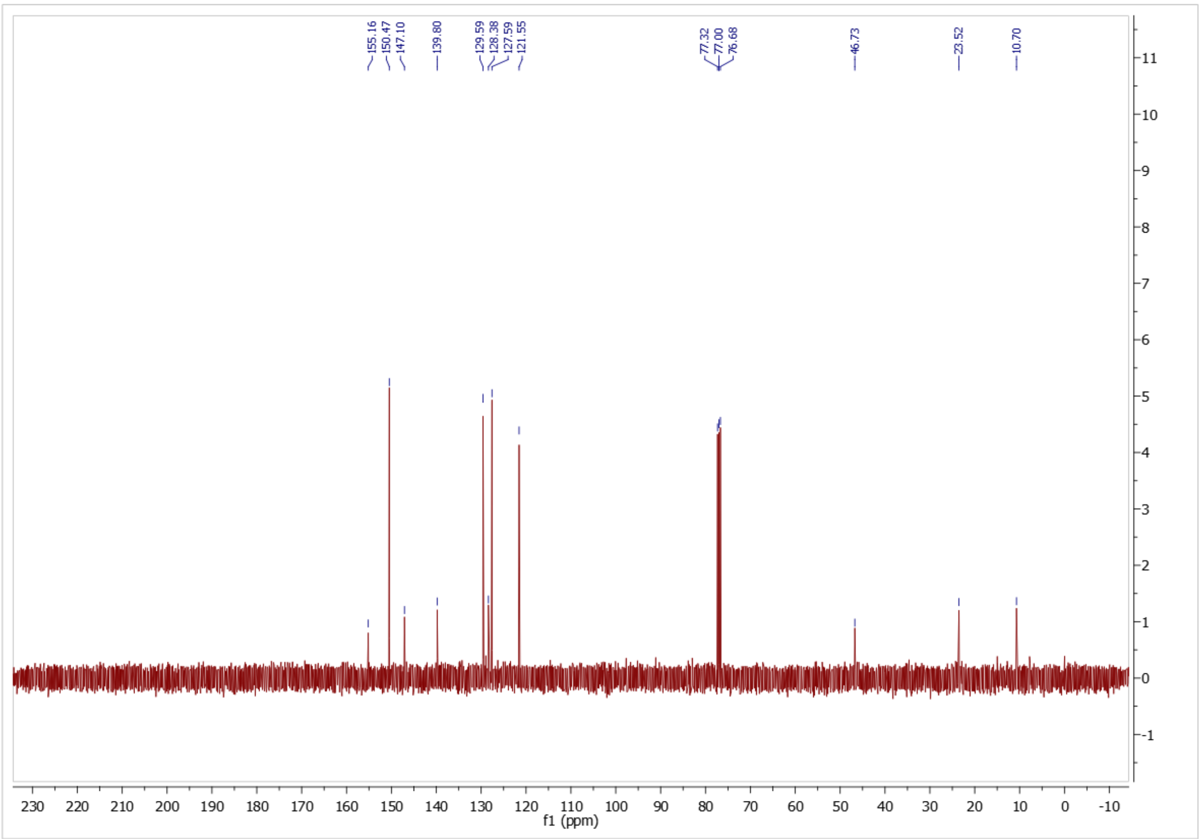

^{1}H and ^{13}C NMR of 4-propyl-3,5-bis[4-(pyridin-3-yl)phenyl]-4*H*-1,2,4-triazole **(6j)**

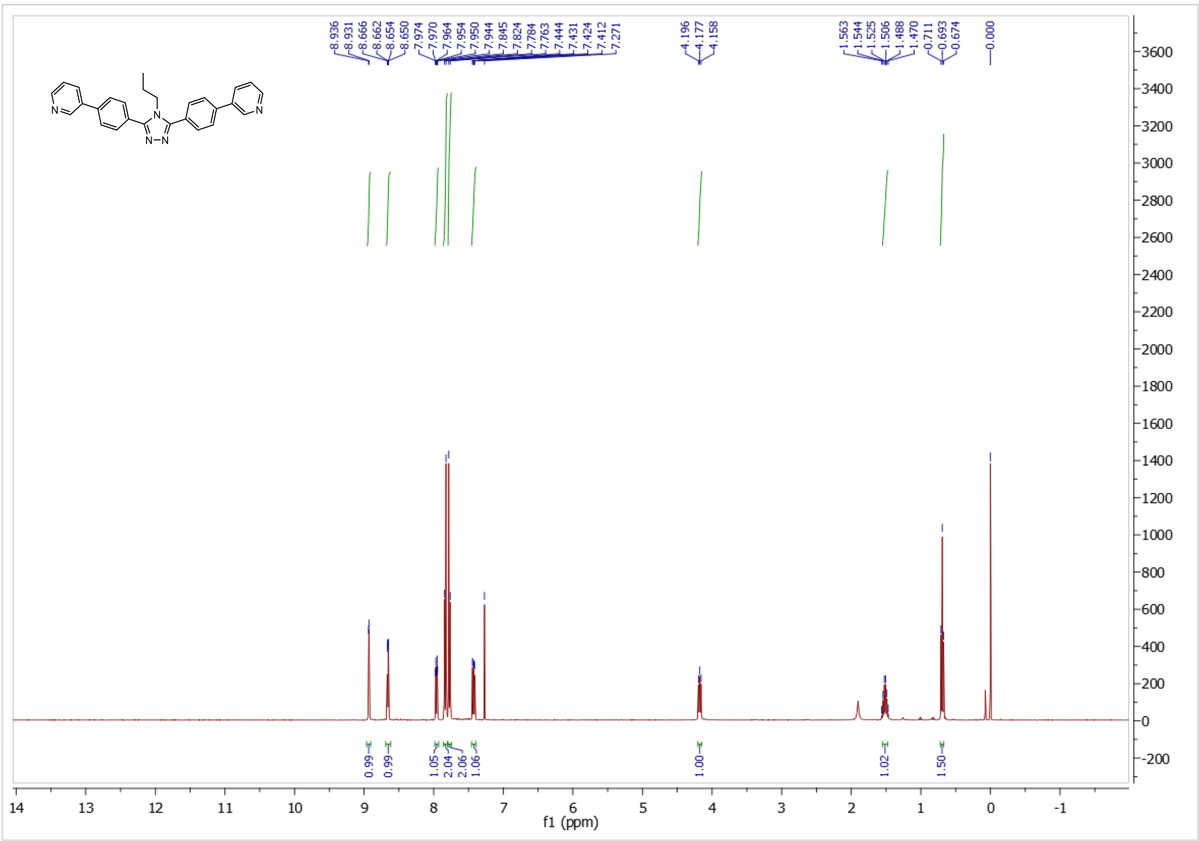

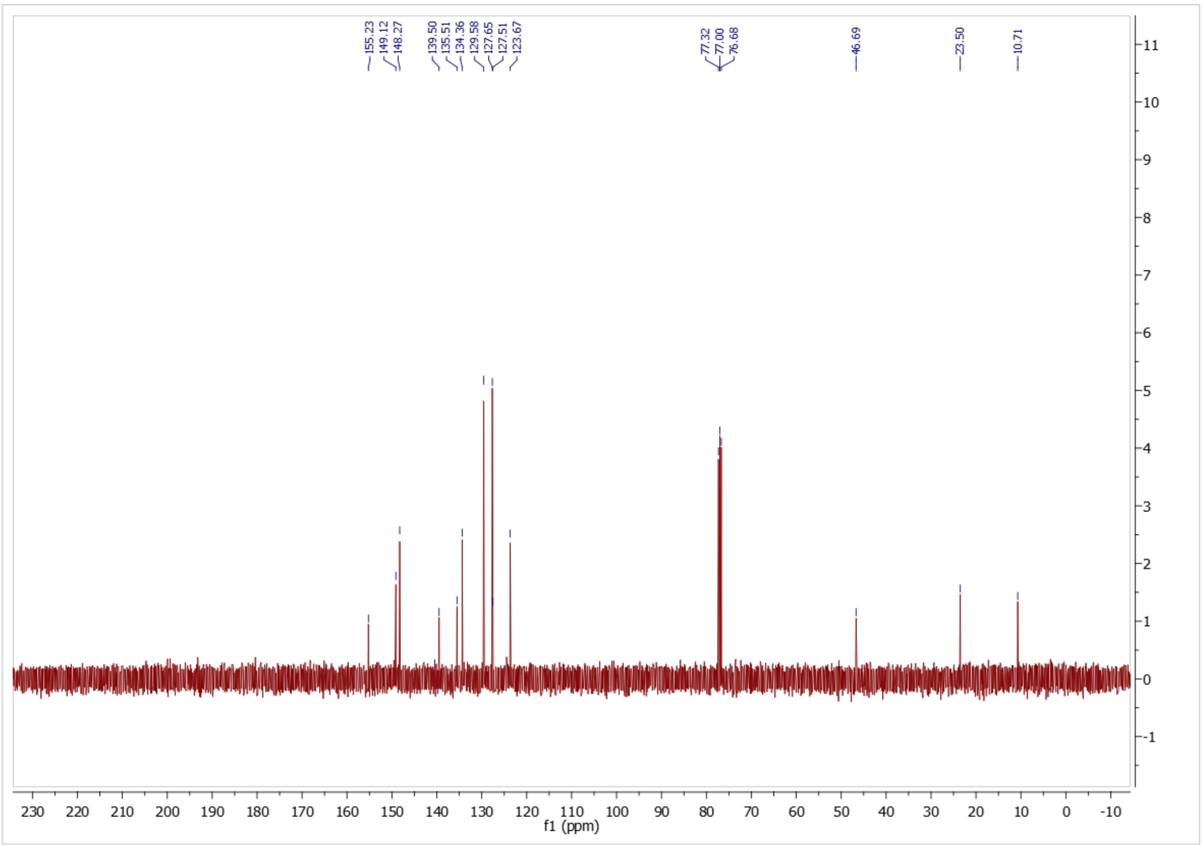

^{1}H and ^{13}C NMR of 3,5-bis[4-(furan-2-yl)phenyl]-4-propyl-4*H*-1,2,4-triazole **(6k)**

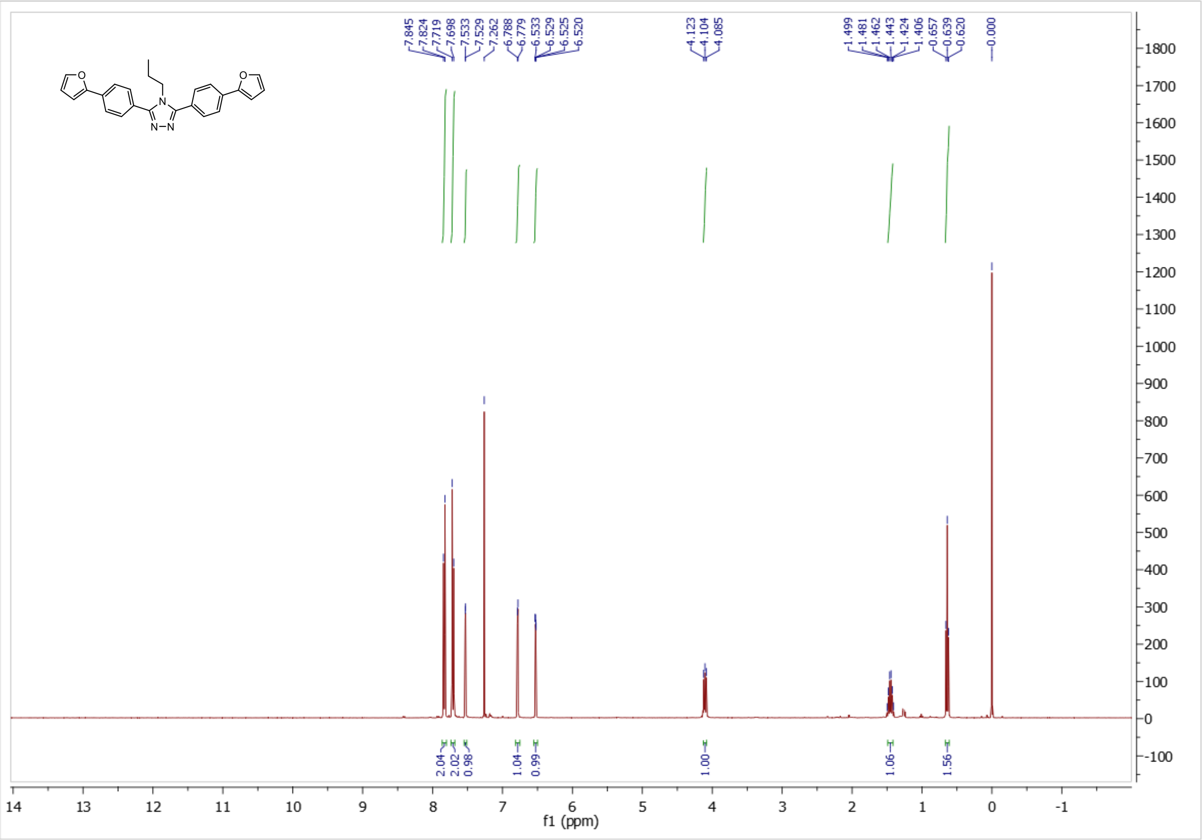

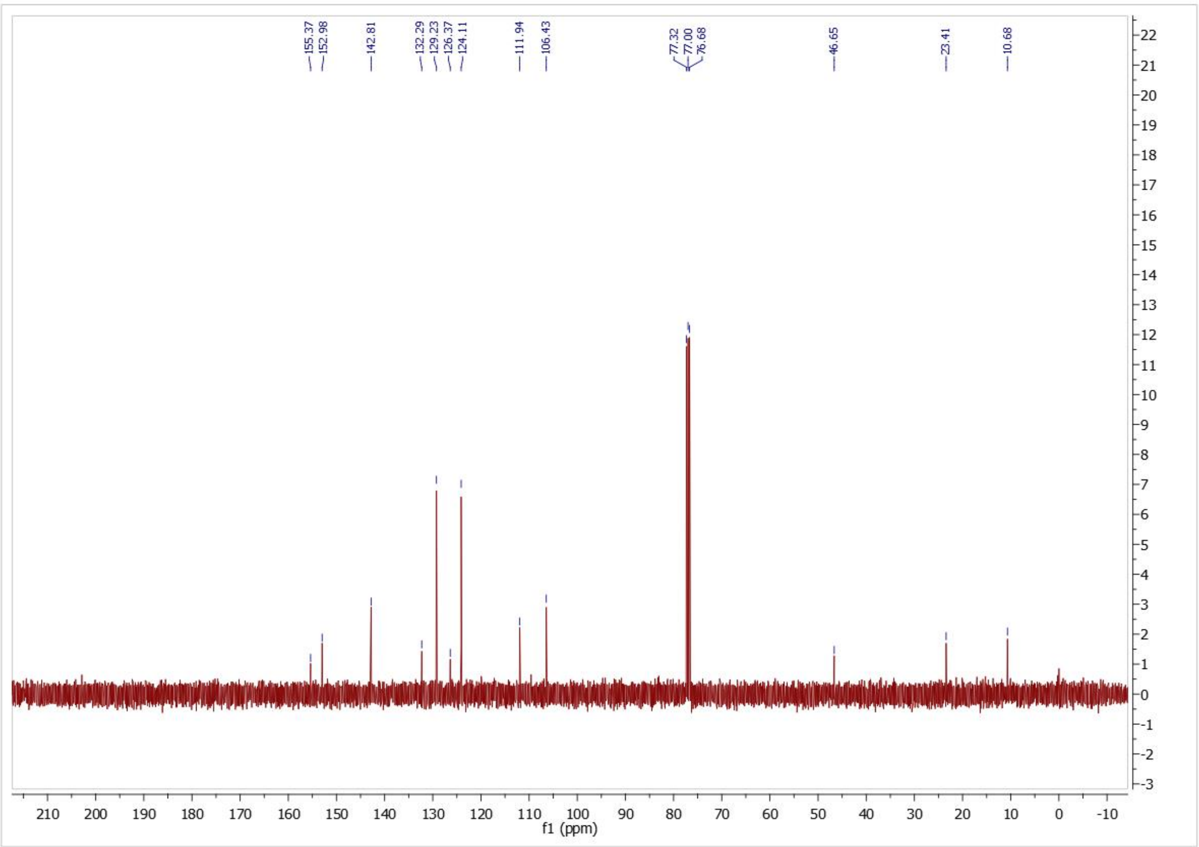

^{1}H and ^{13}C NMR of 3,5-bis[4-(furan-3-yl)phenyl]-4-propyl-4*H*-1,2,4-triazole **(6l)**

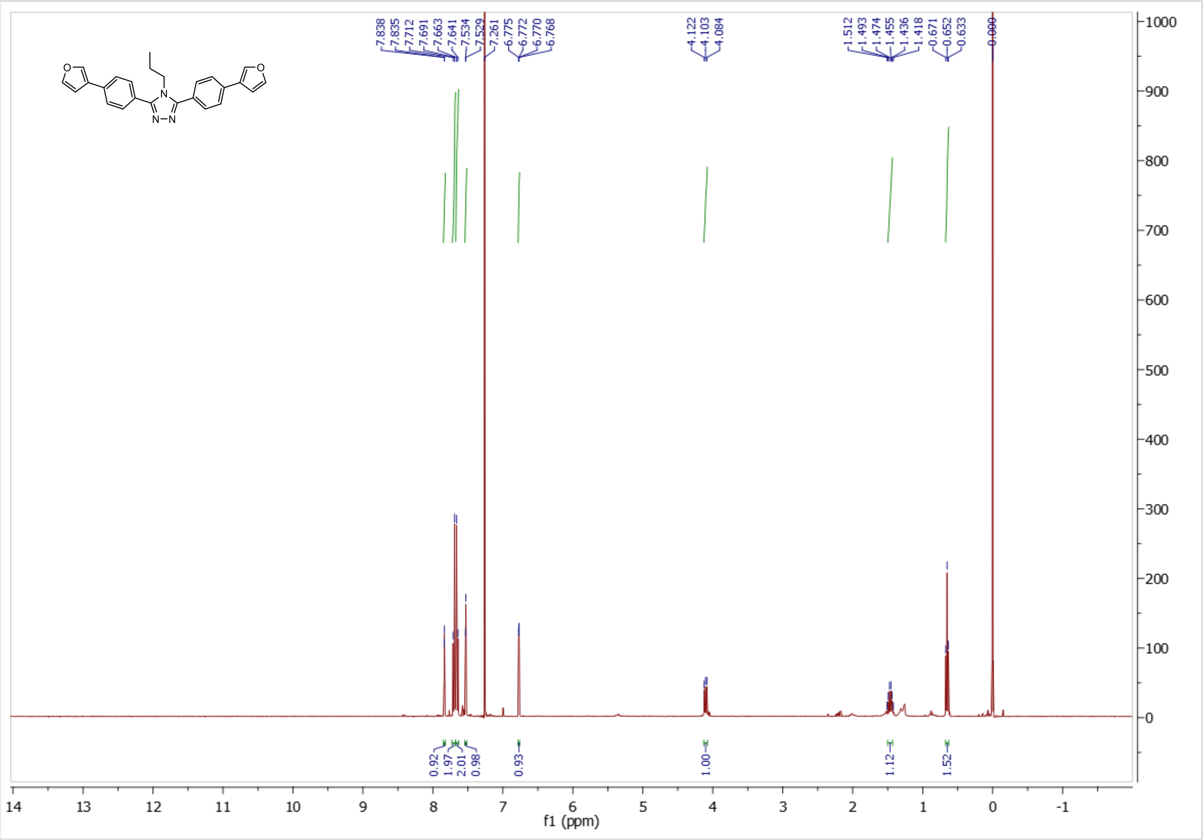

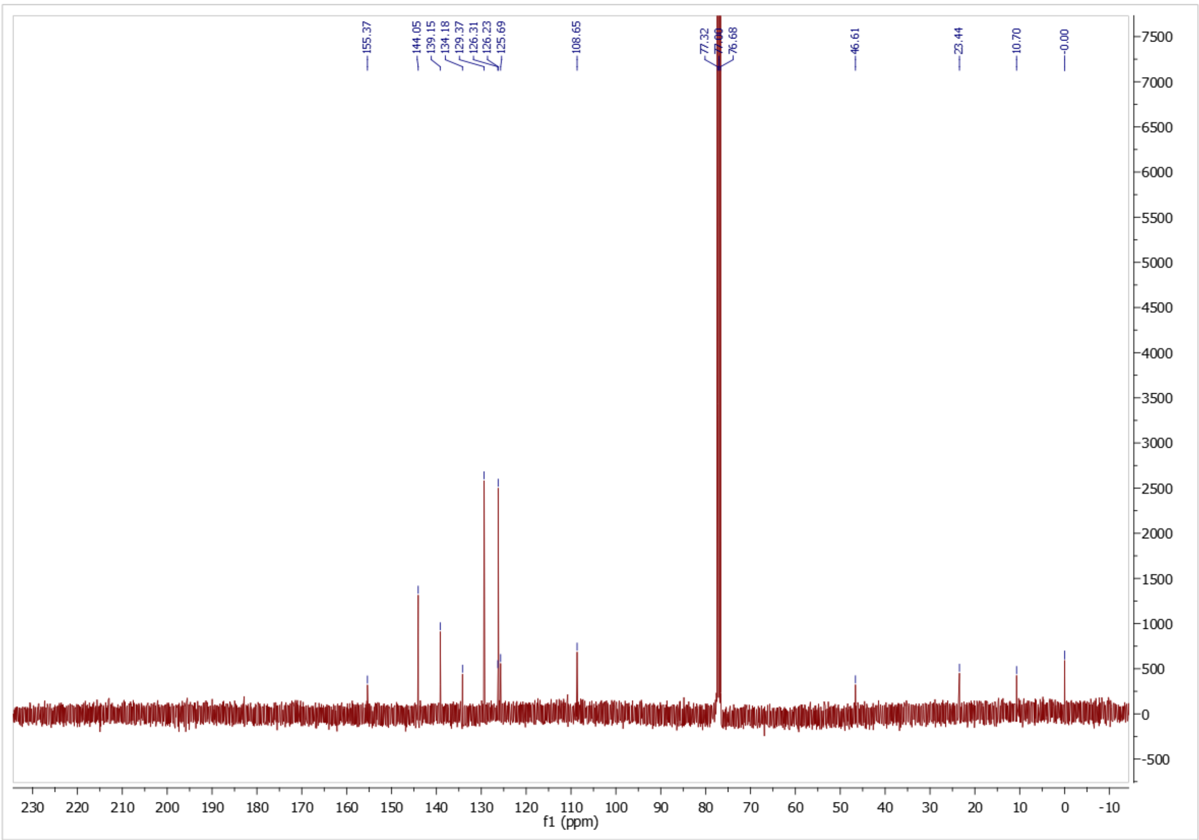

^{1}H and ^{13}C NMR of 4-propyl-3,5-bis[4-(thiophen-2-yl)phenyl]-4*H*-1,2,4-triazole **(6m)**

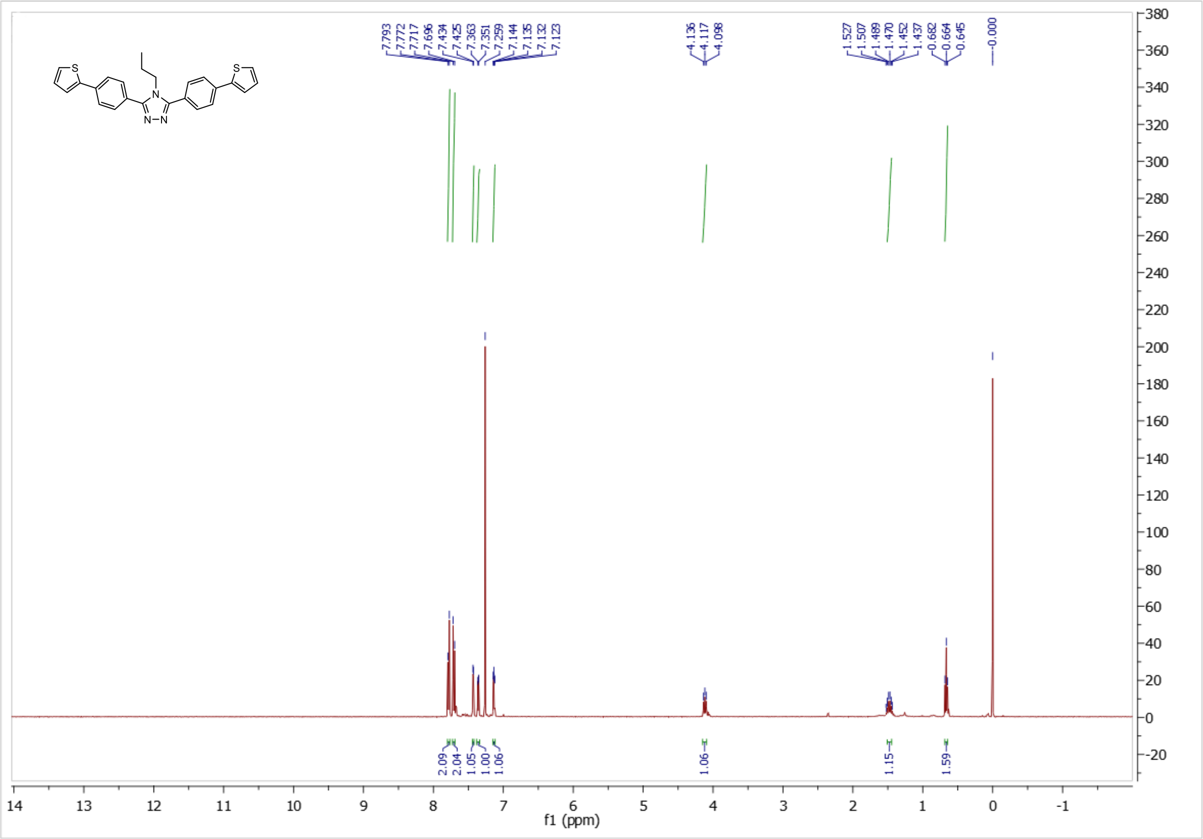

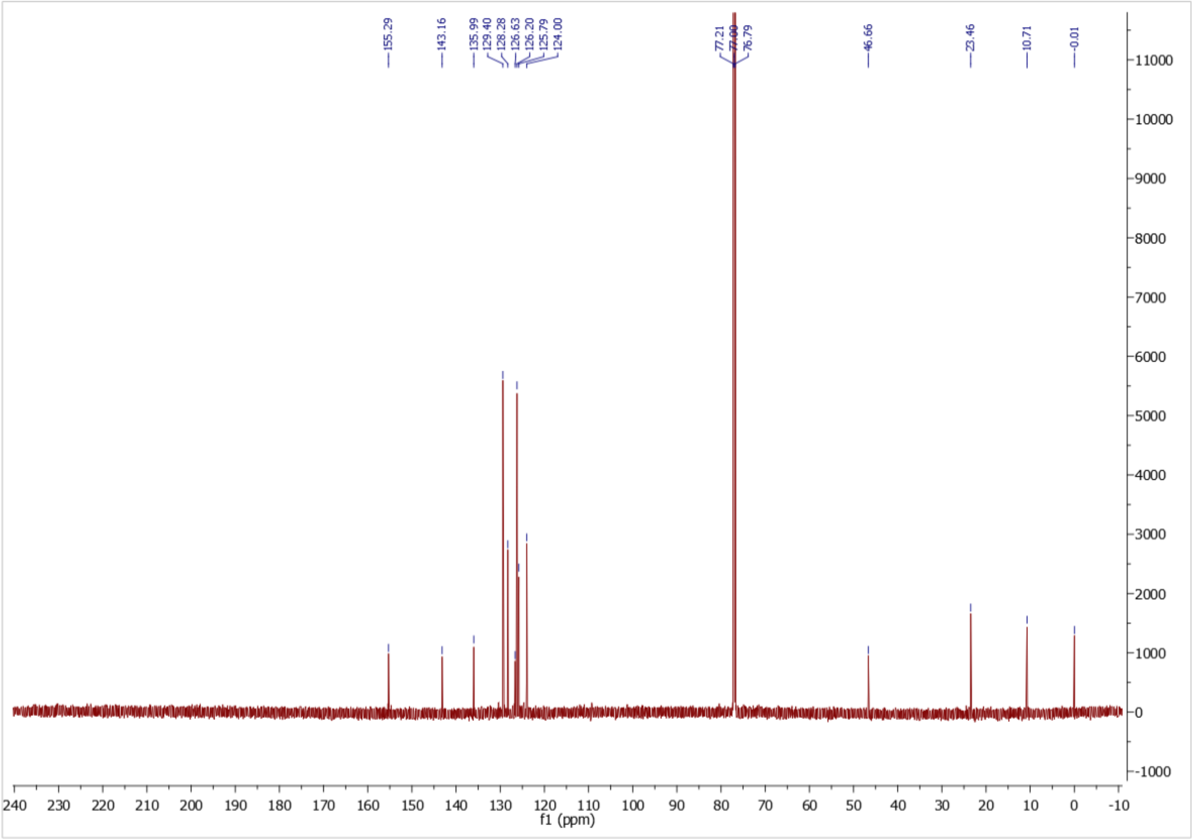

^{1}H and ^{13}C NMR of 4-propyl-3,5-bis[4-(thiophen-3-yl)phenyl]-4*H*-1,2,4-triazole **(6n)**

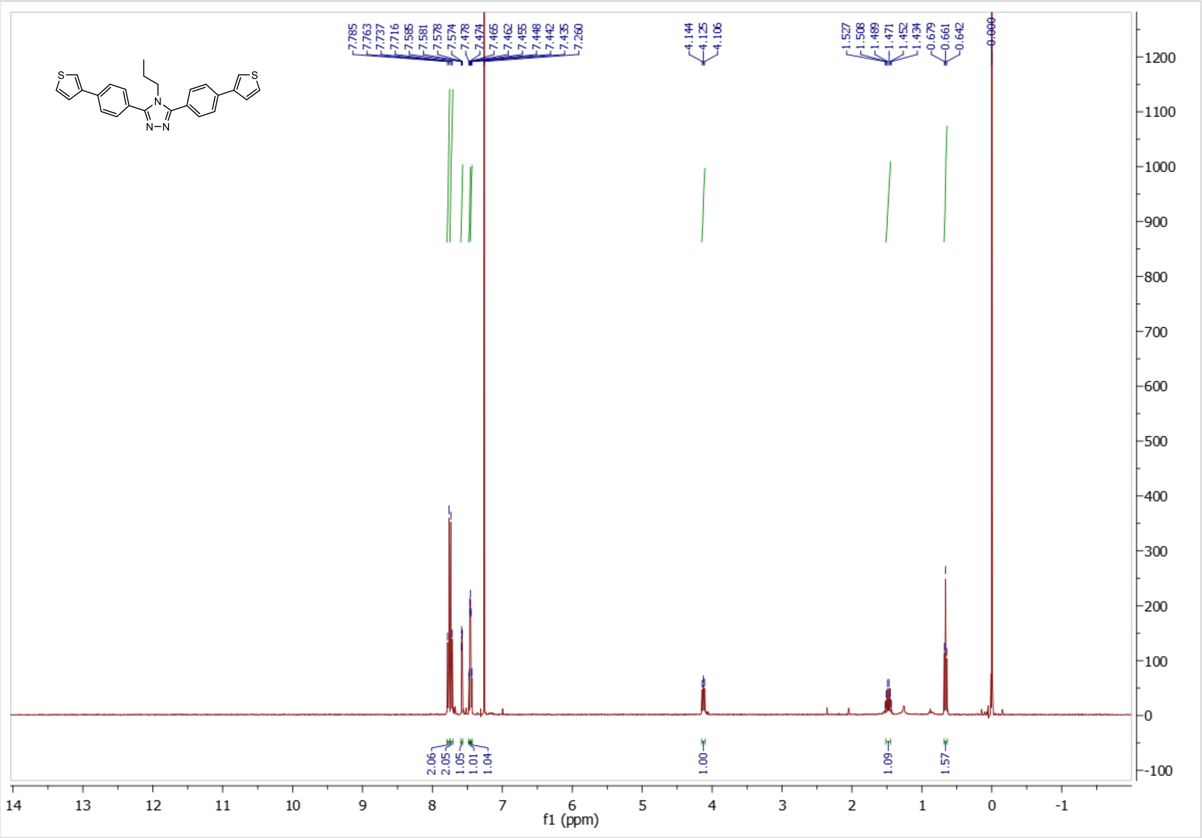

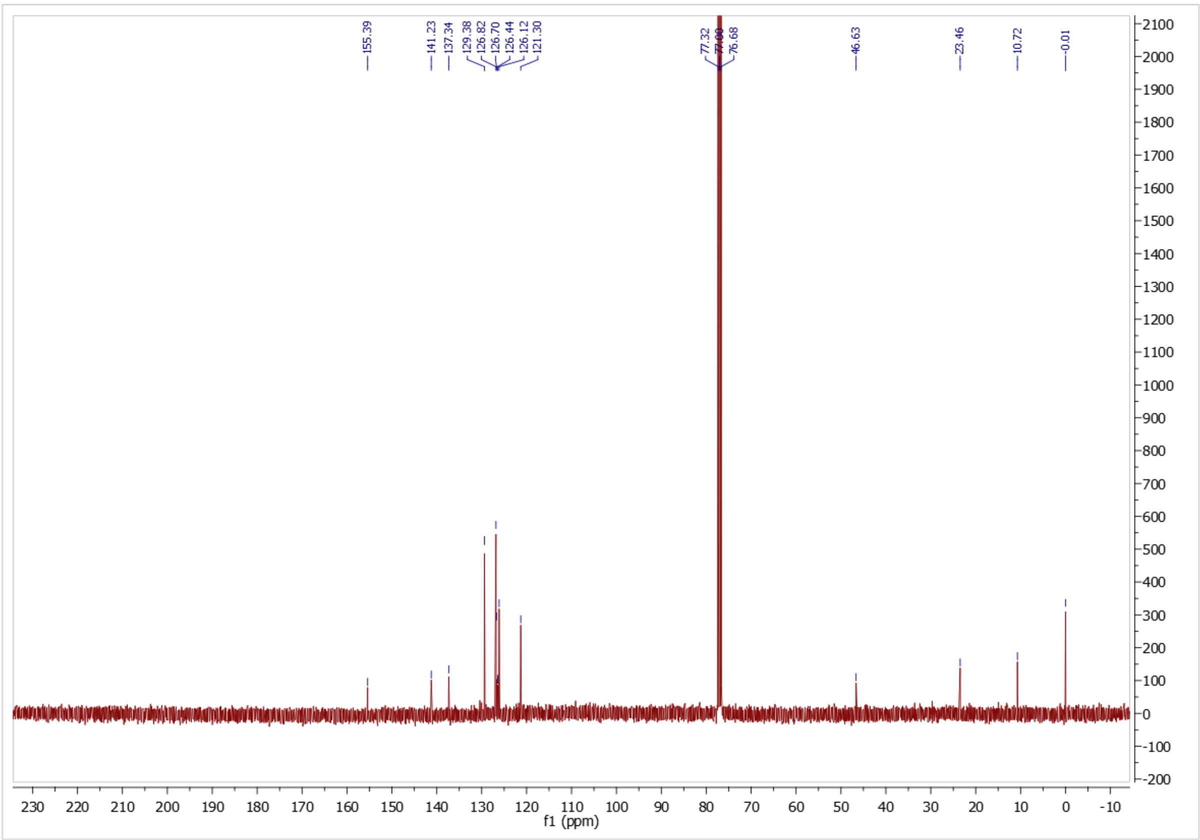

^{1}H and ^{13}C NMR of 3,5-bis(biphenyl-4-yl)-4-butyl-4*H*-1,2,4-triazole **(7a)**

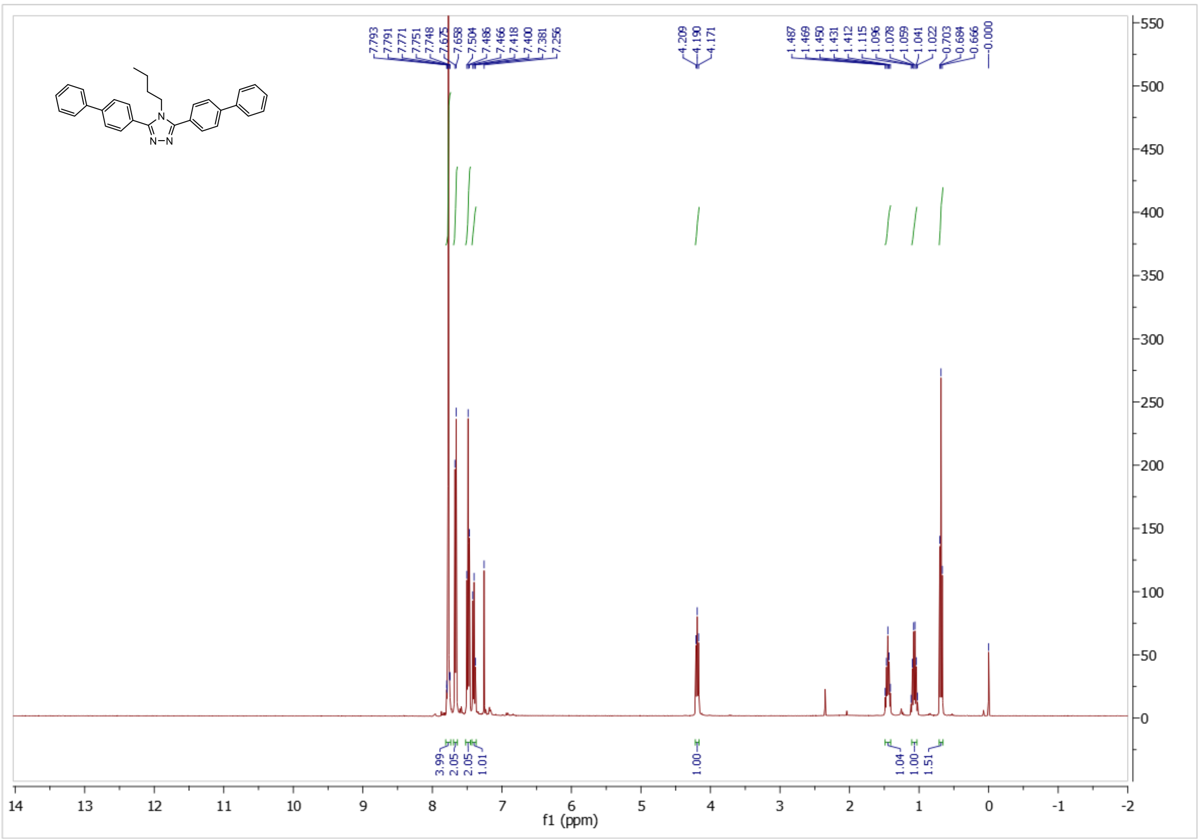

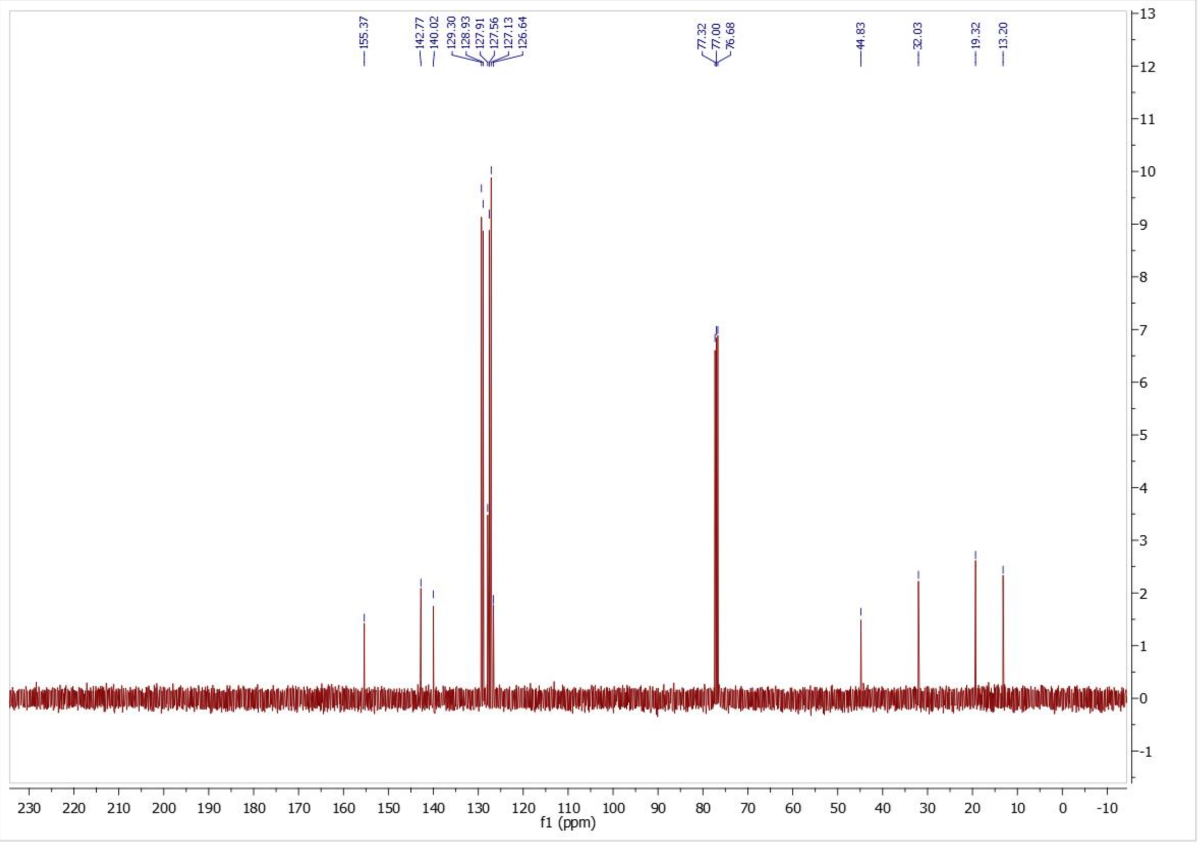

^{1}H and ^{13}C NMR of 4-butyl-3,5-bis(2’-methylbiphenyl-4-yl)-4*H*-1,2,4-triazole **(7b)**

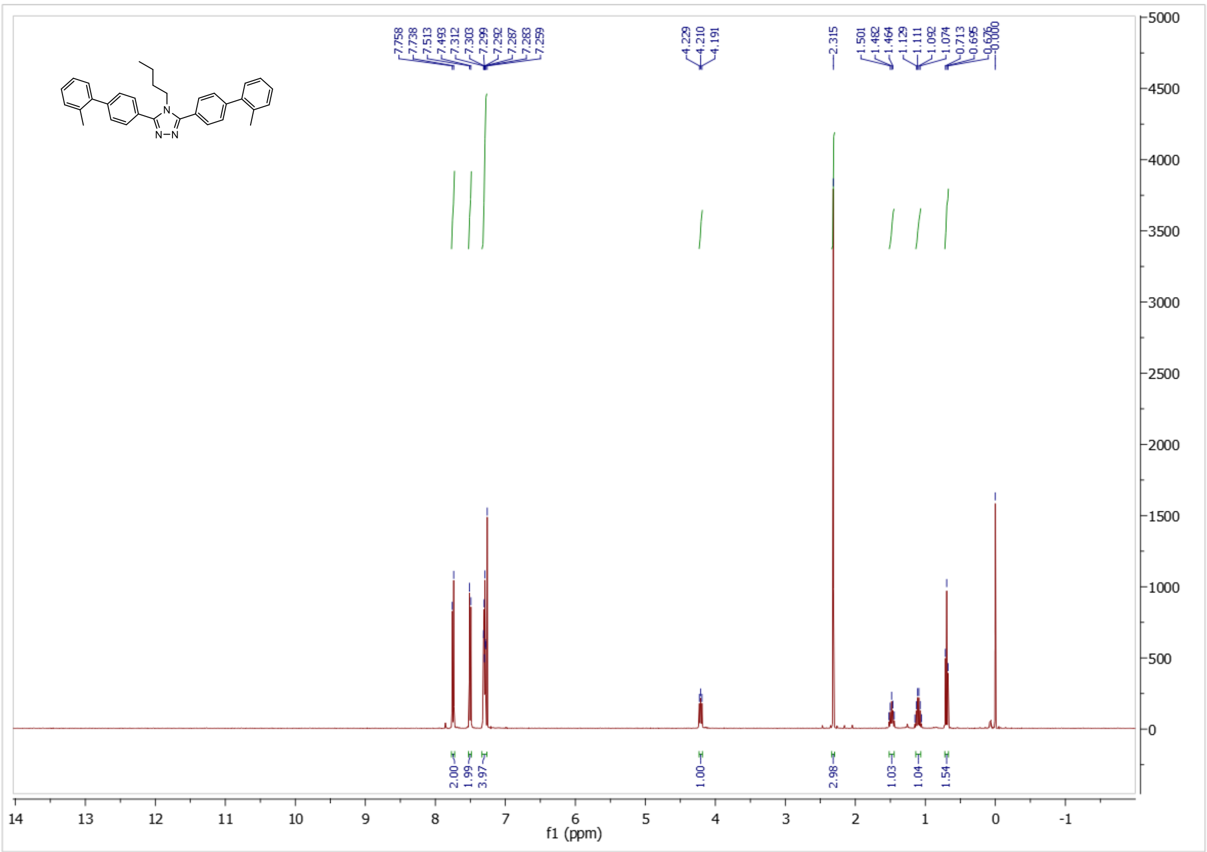

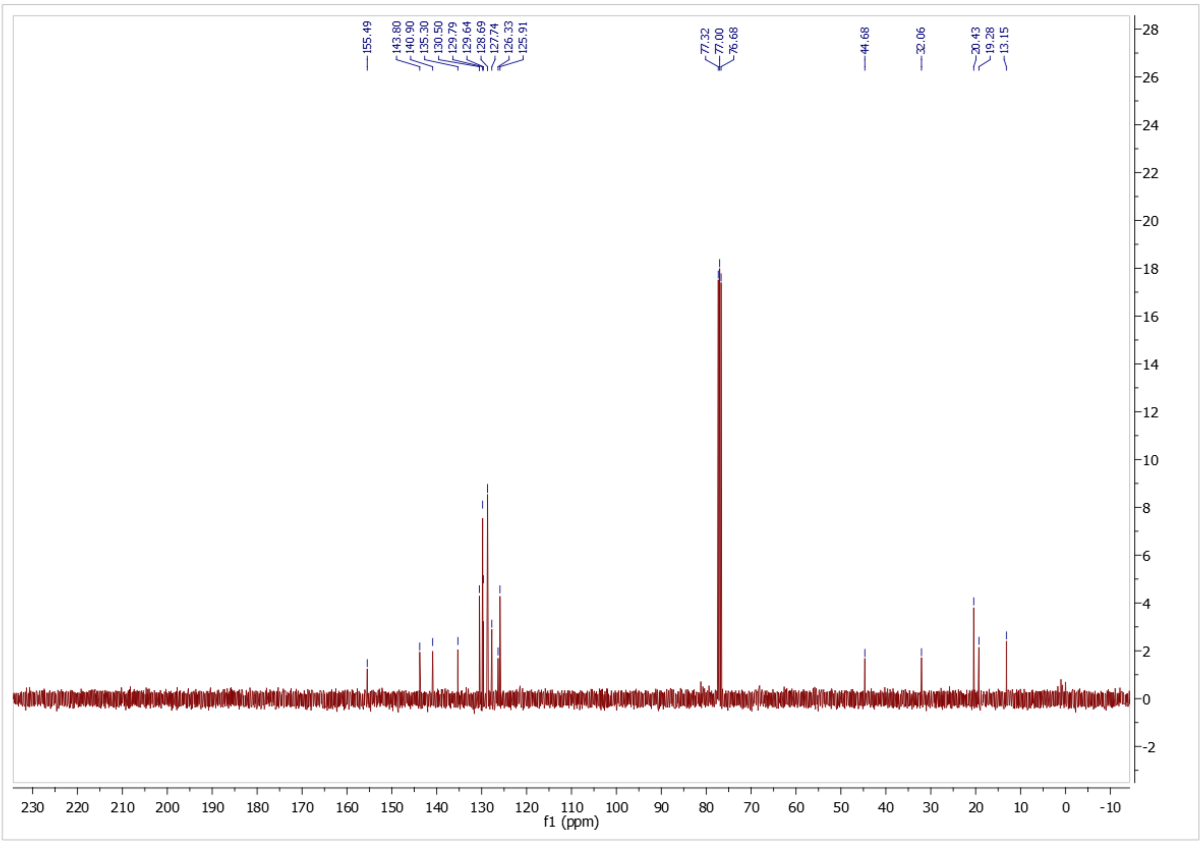

^{1}H and ^{13}C NMR of 4-butyl-3,5-bis(3'-methylbiphenyl-4-yl)-4*H*-1,2,4-triazole **(7c)**

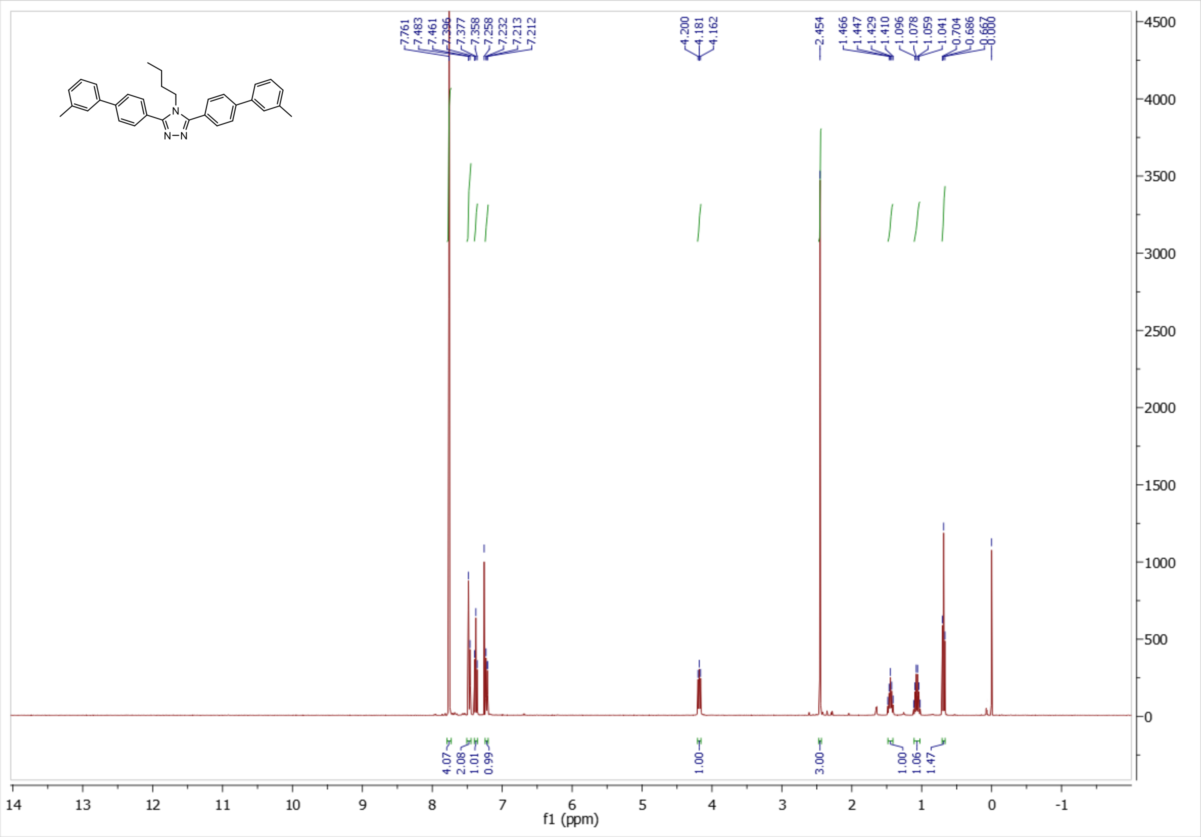

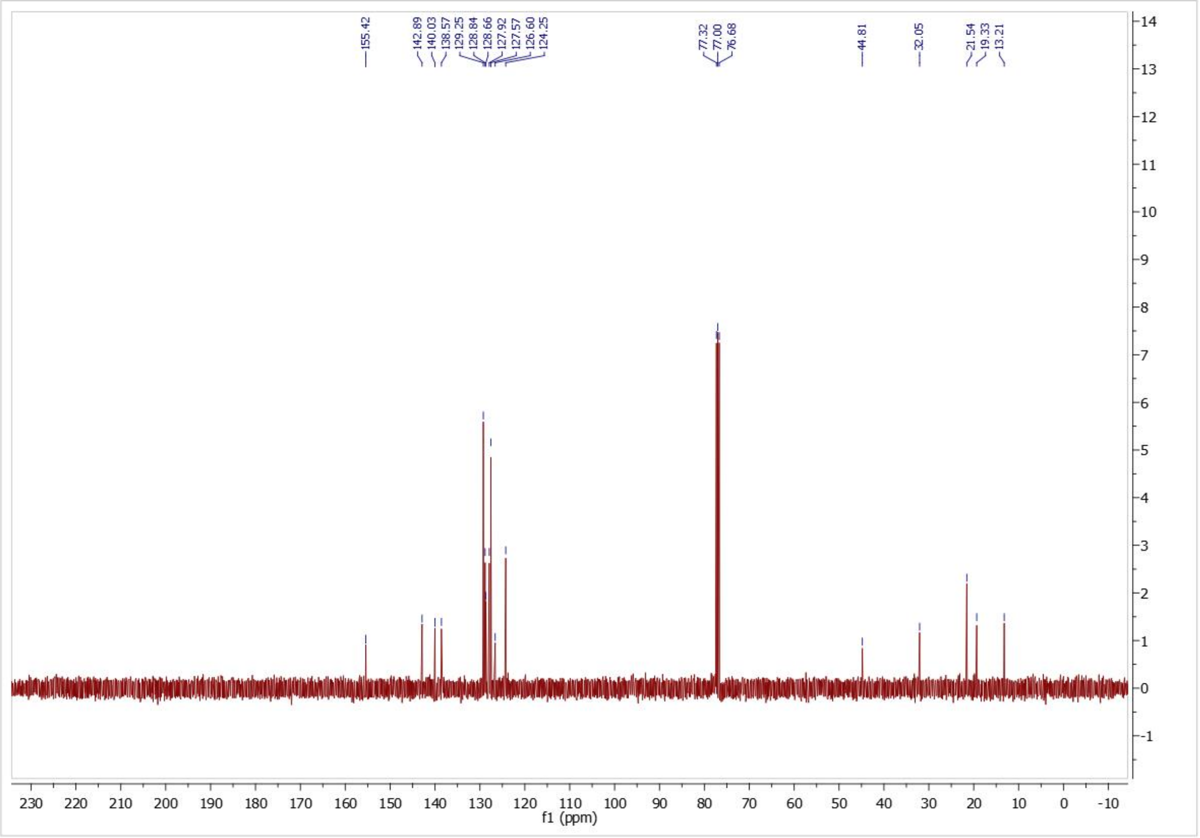

^{1}H and ^{13}C NMR of 4-butyl-3,5-bis(2’,6’-dimethylbiphenyl-4-yl)-4*H*-1,2,4-triazole **(7d)**

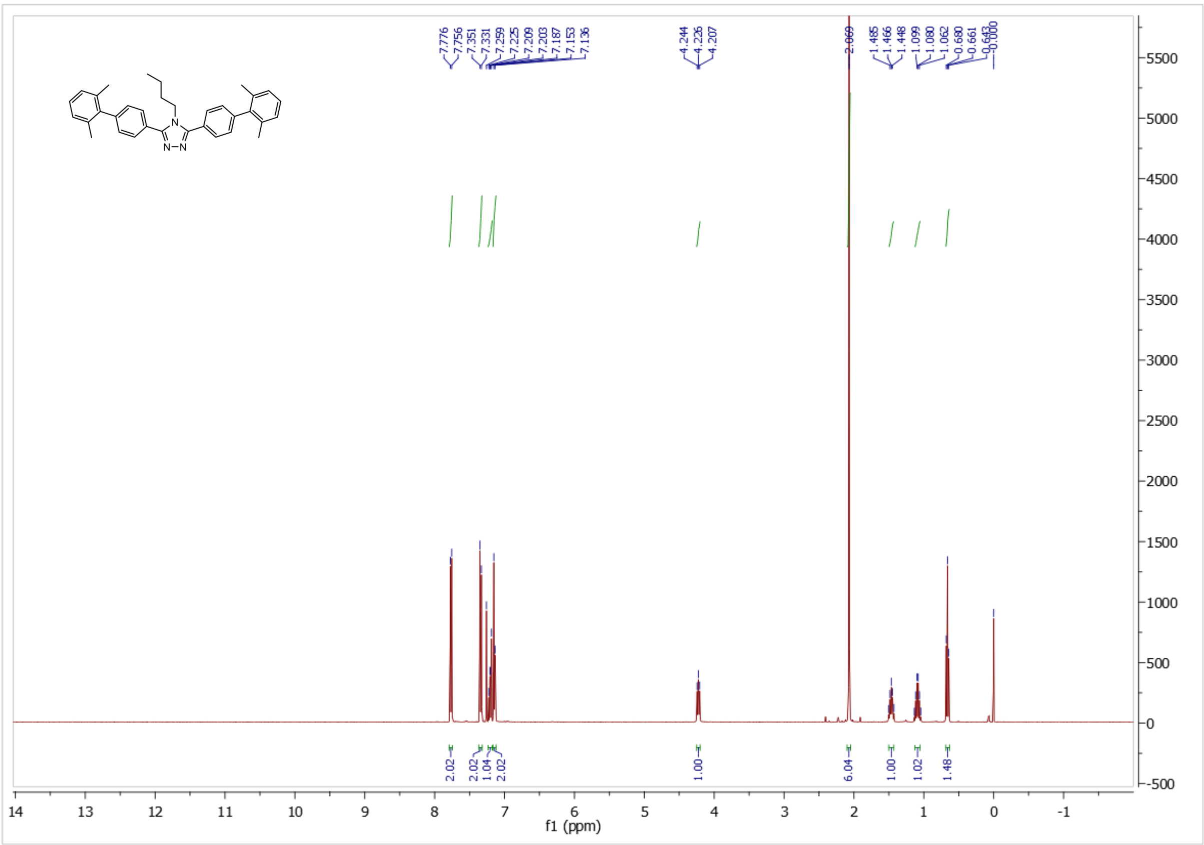

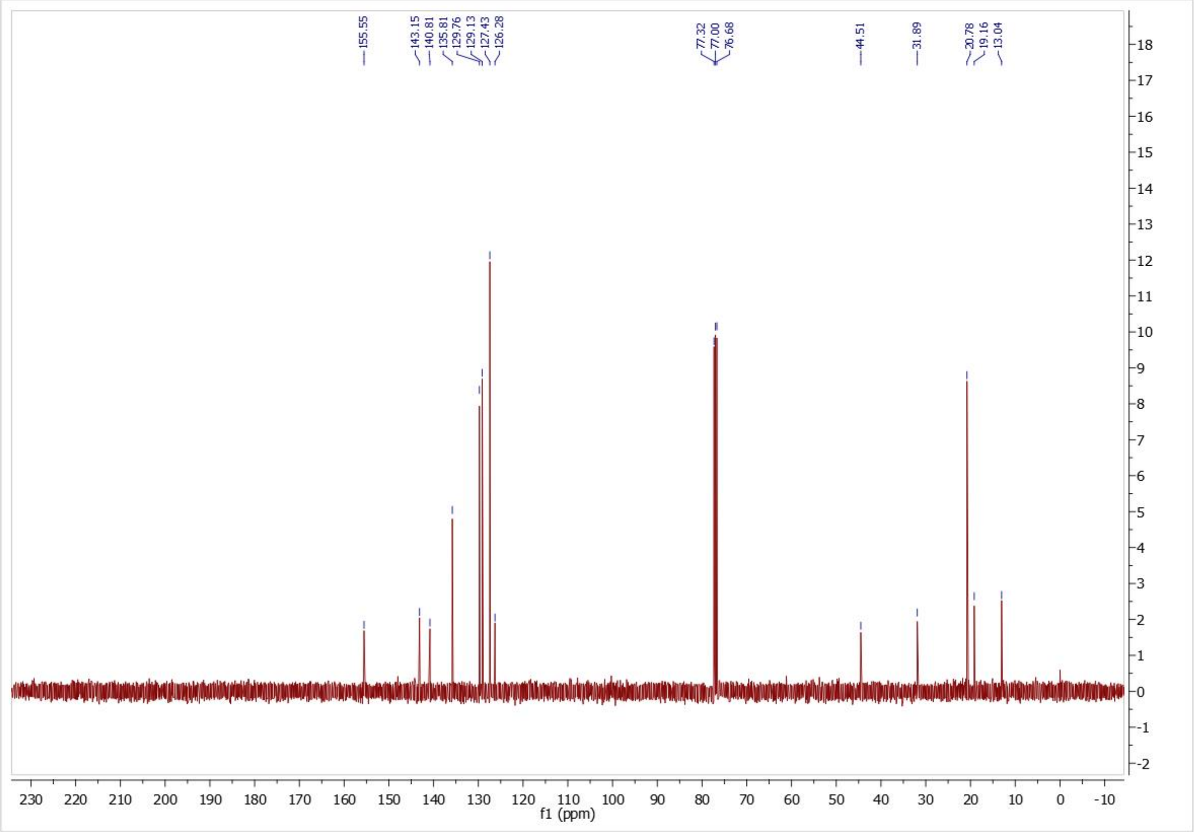

^{1}H and ^{13}C NMR of 4-butyl-3,5-bis(2'-methoxybiphenyl-4-yl)-4*H*-1,2,4-triazole **(7e)**

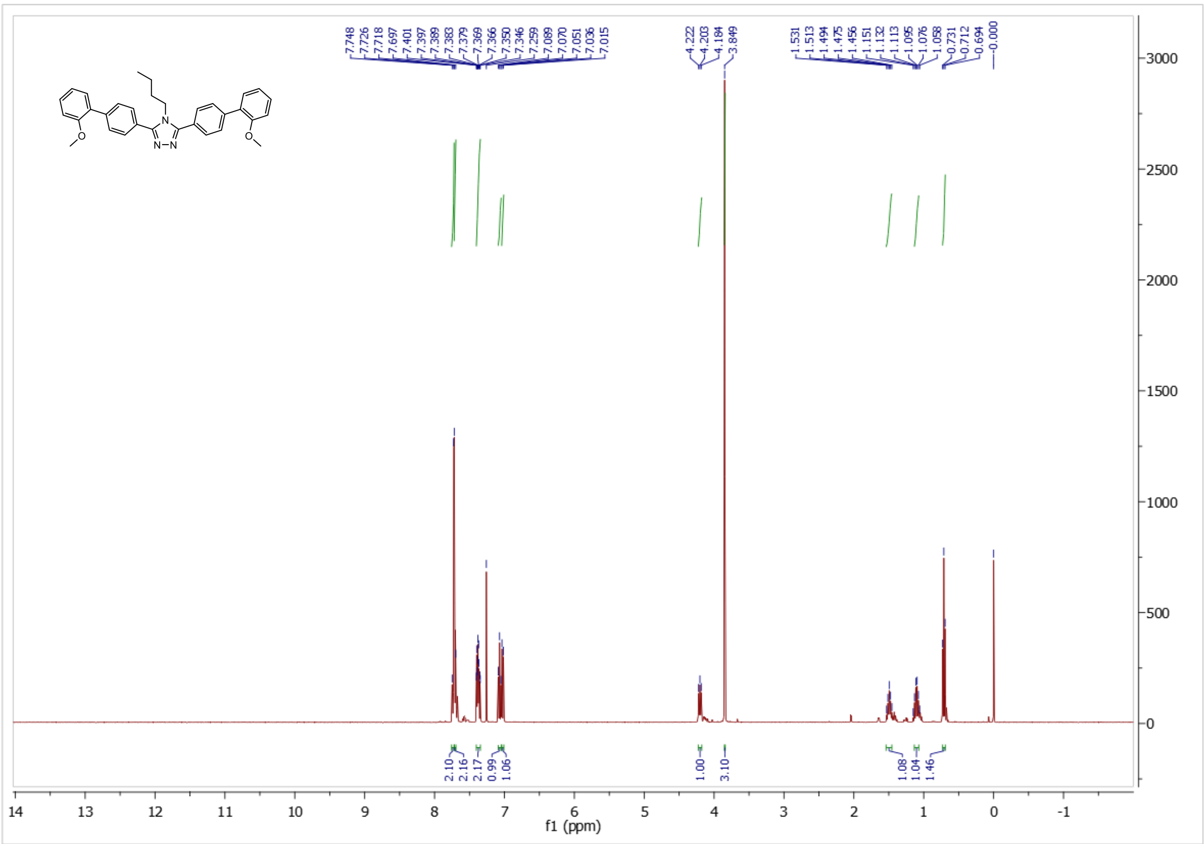

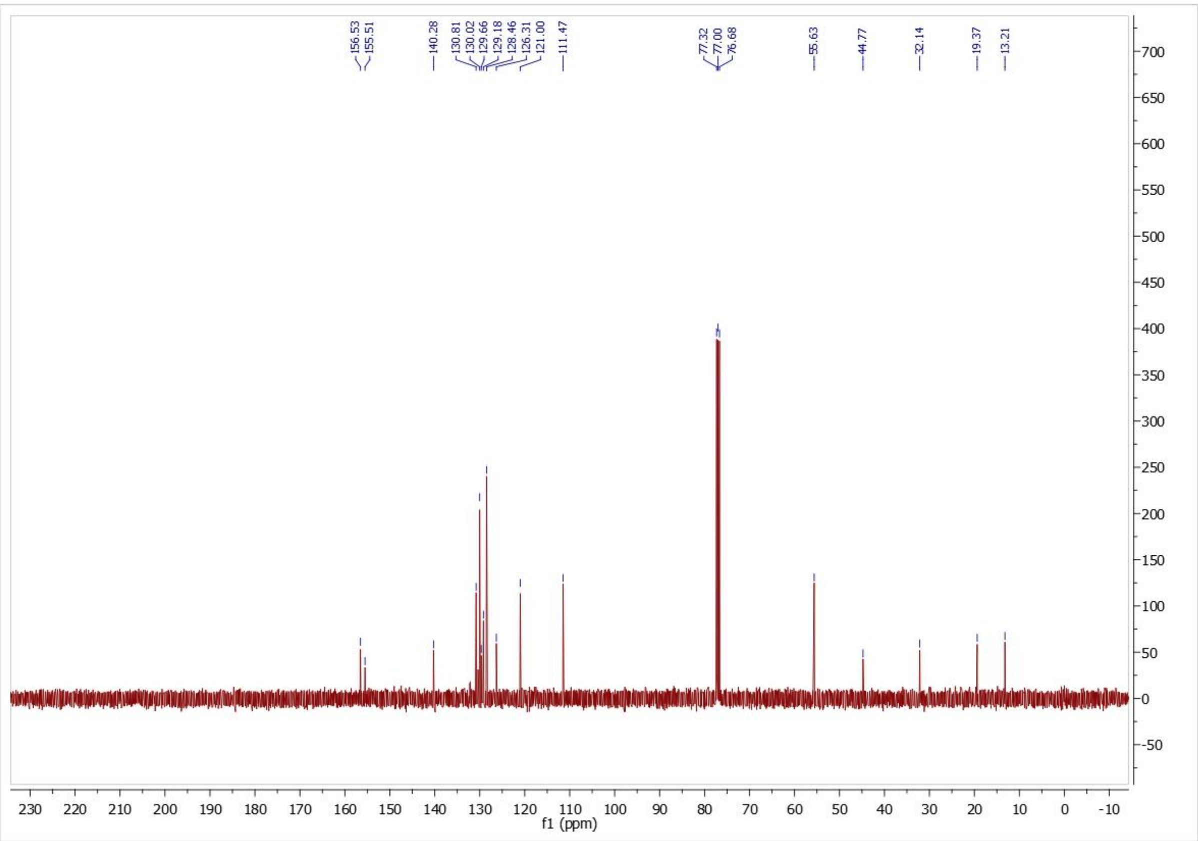

^{1}H and ^{13}C NMR of 4-butyl-3,5-bis(3'-methoxybiphenyl-4-yl)-4*H*-1,2,4-triazole **(7f)**

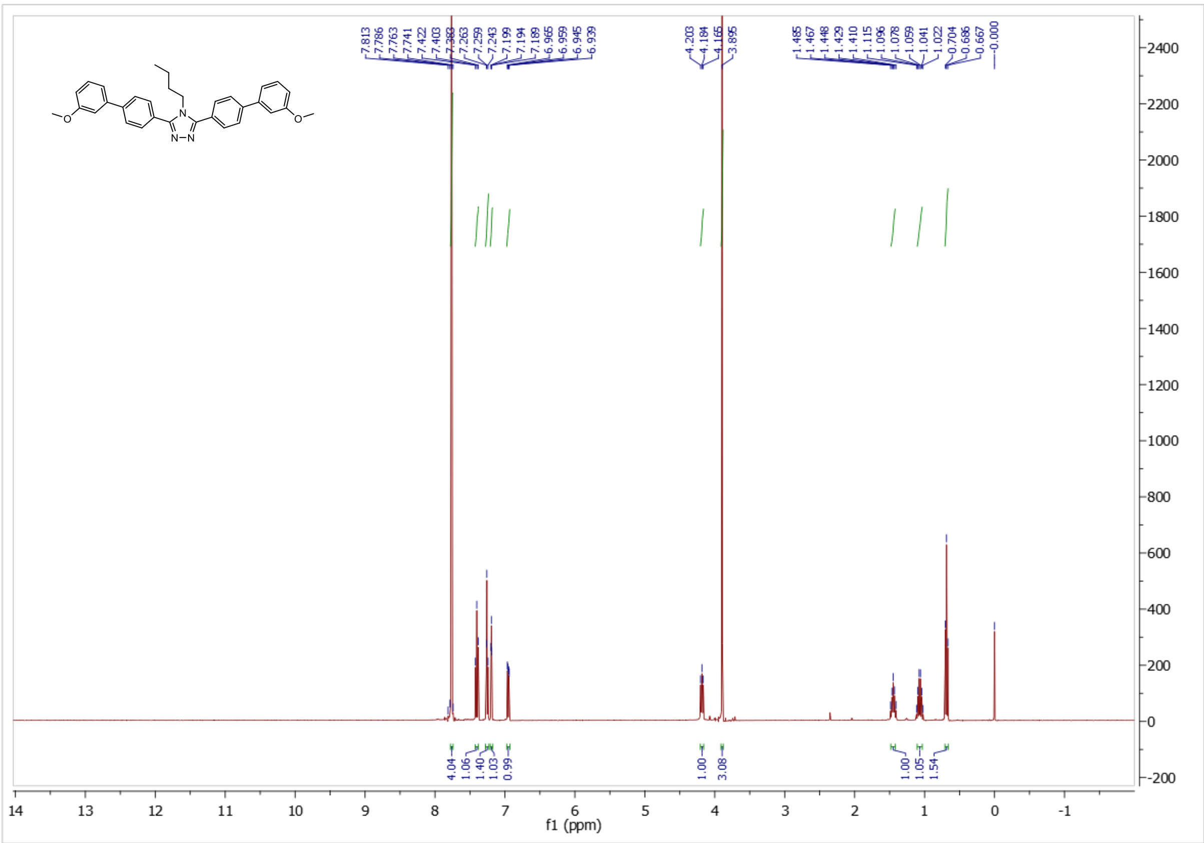

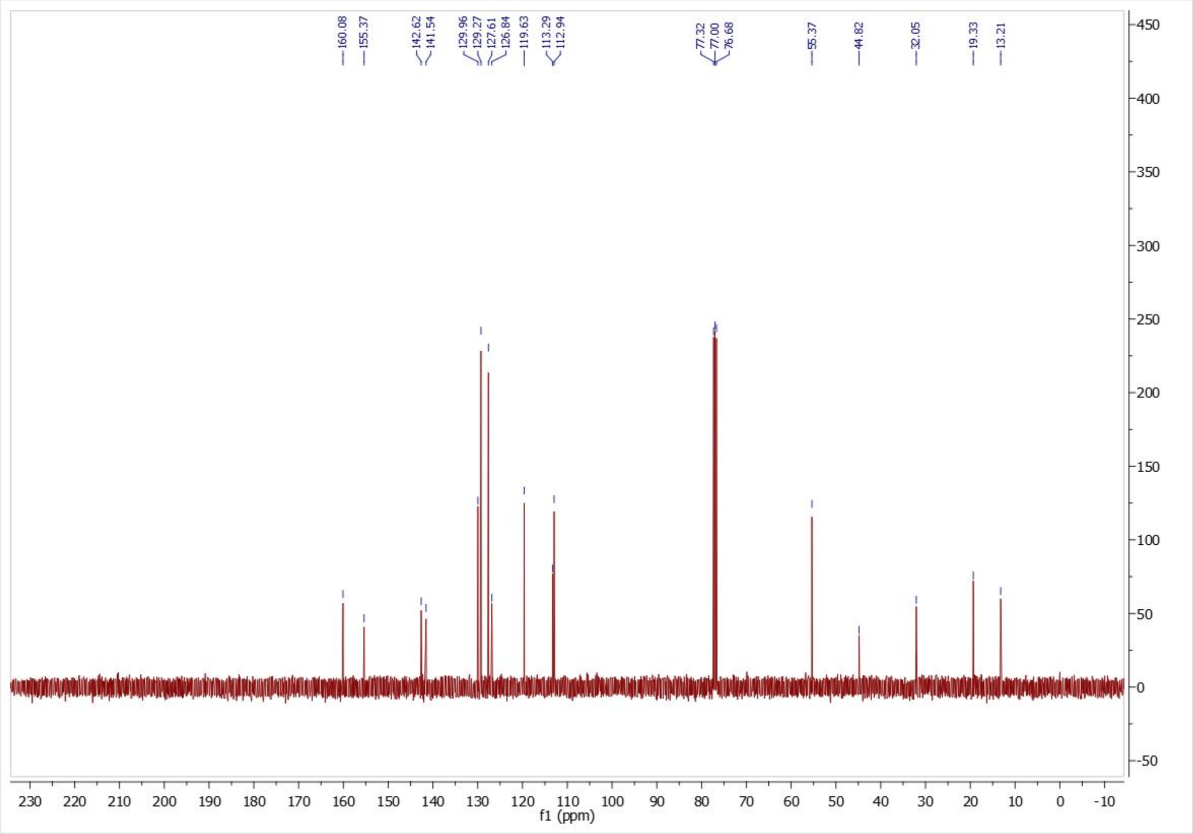

^{1}H and ^{13}C NMR of 4-butyl-3,5-bis(3'-nitrobiphenyl-4-yl)-4*H*-1,2,4-triazole **(7g)**

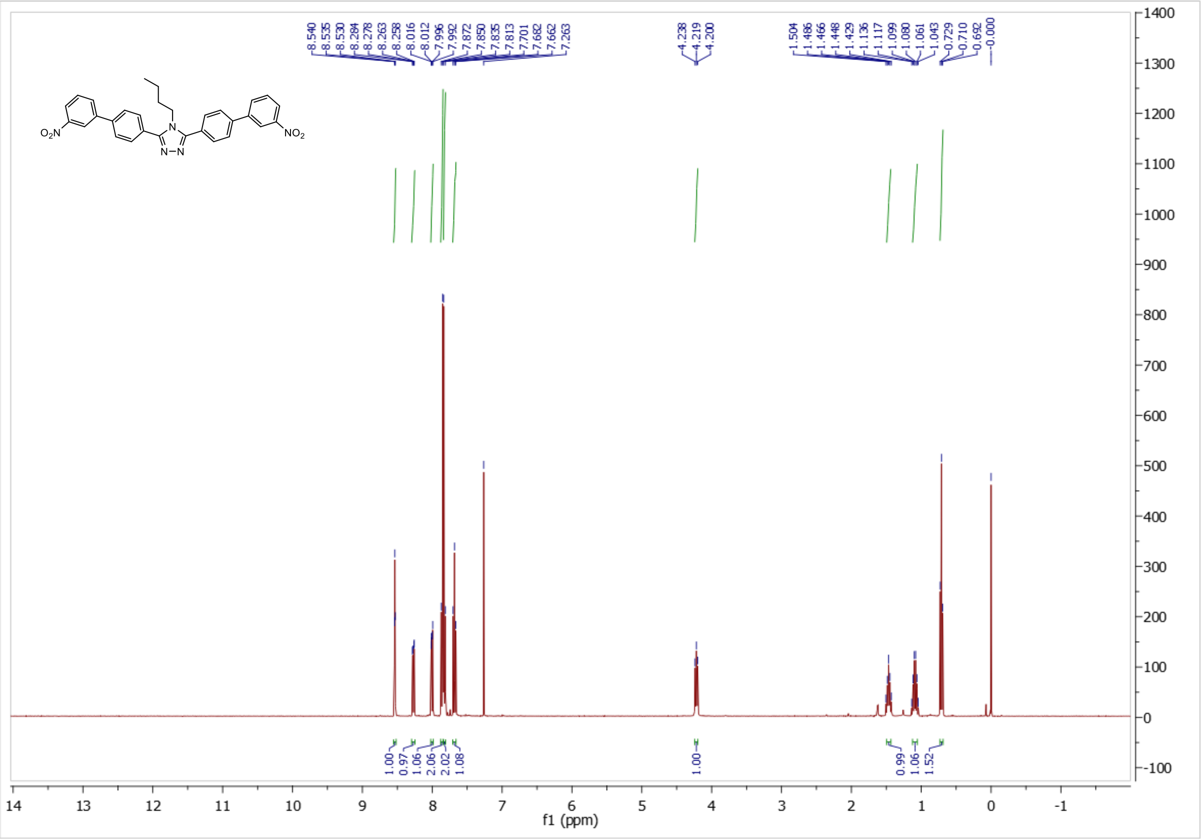

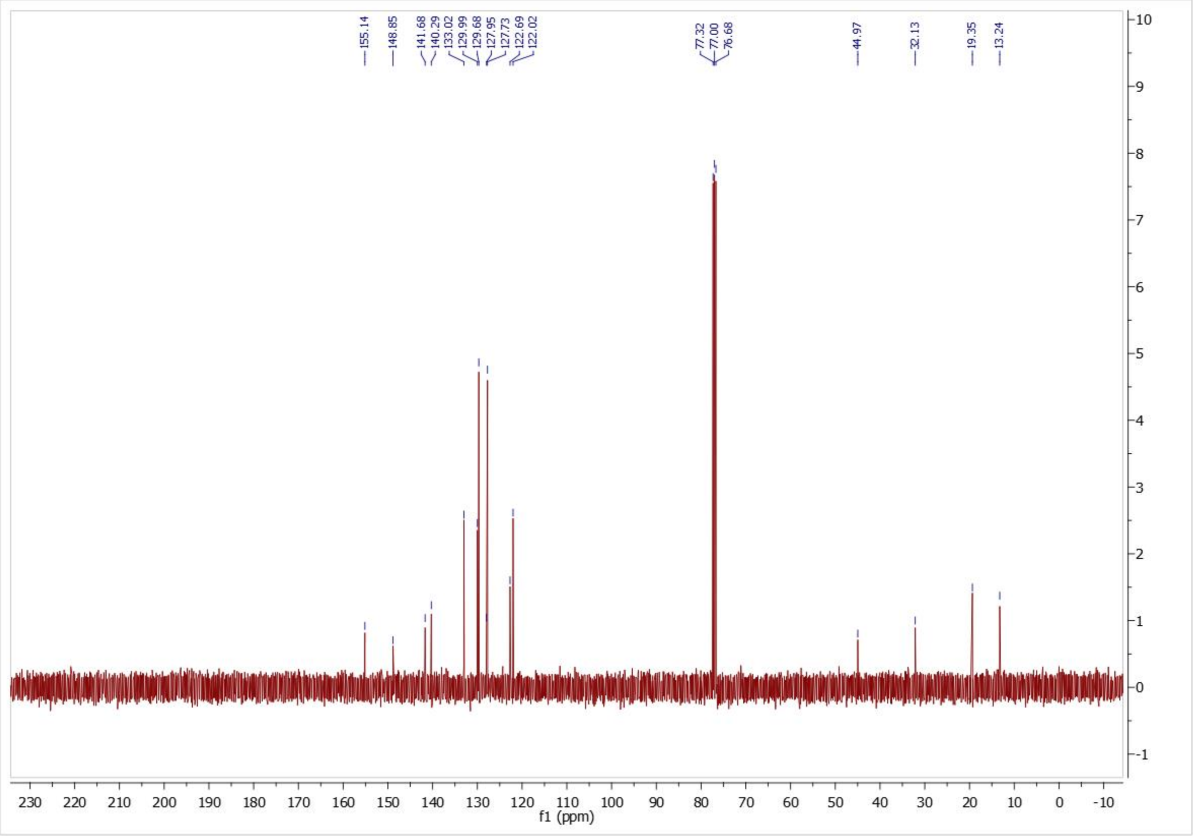

^{1}H and ^{13}C NMR of 3,5-bis(3’-aminobiphenyl-4-yl)-4-butyl-4*H*-1,2,4-triazole **(7h)**

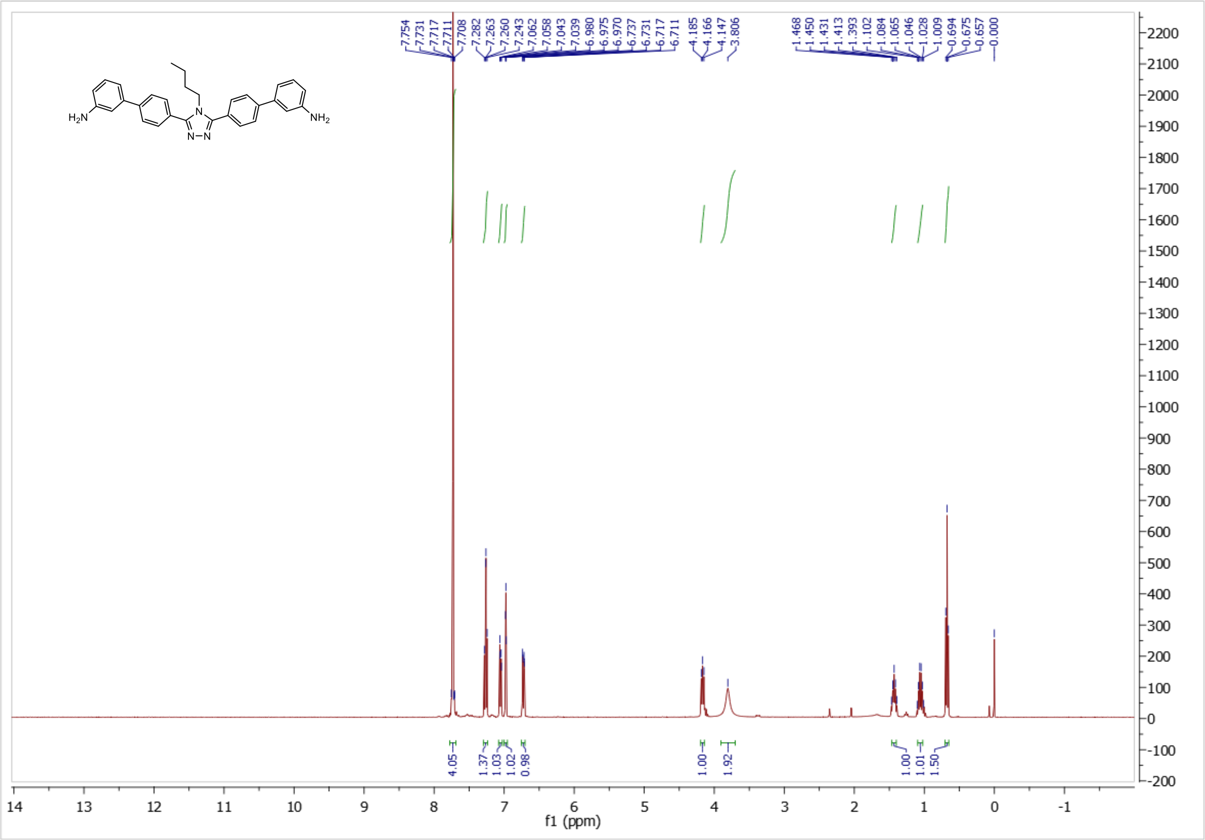

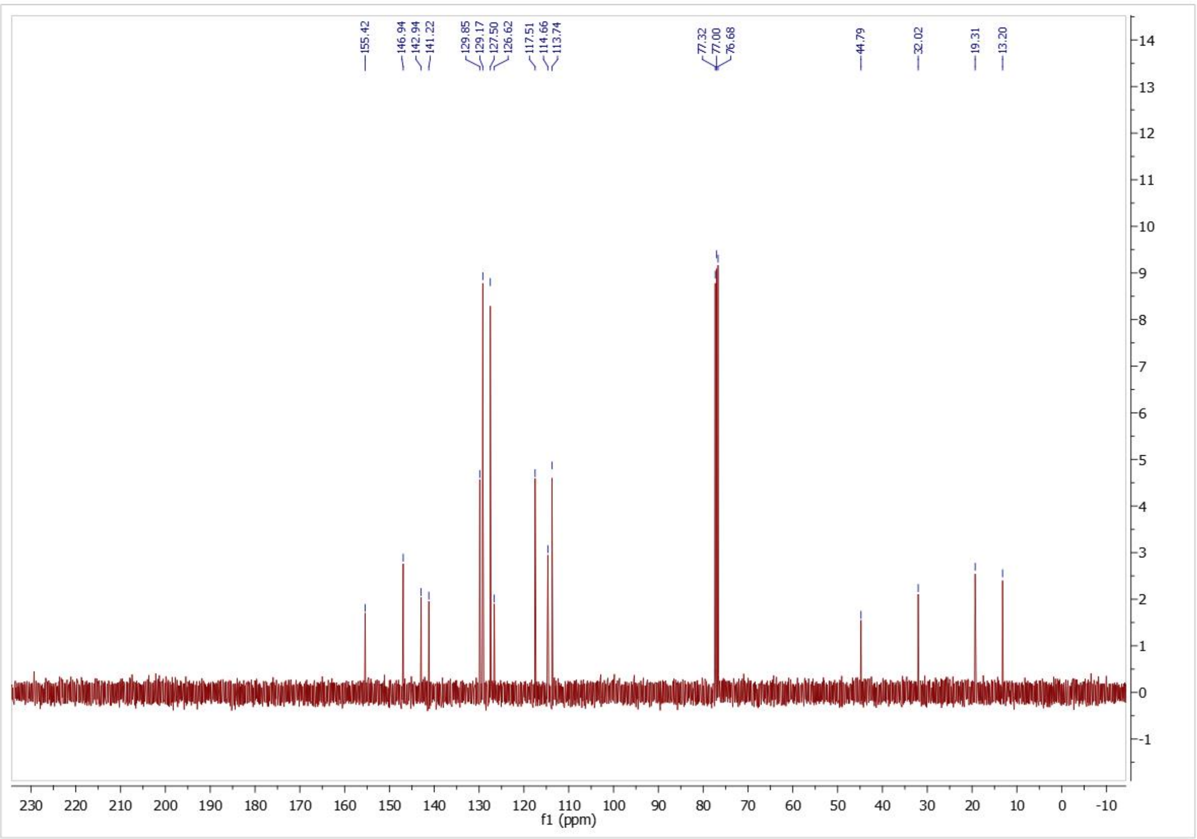

^{1}H and ^{13}C NMR of 4-butyl-3,5-bis[4-(pyridin-4-yl)phenyl]-4*H*-1,2,4-triazole **(7i)**

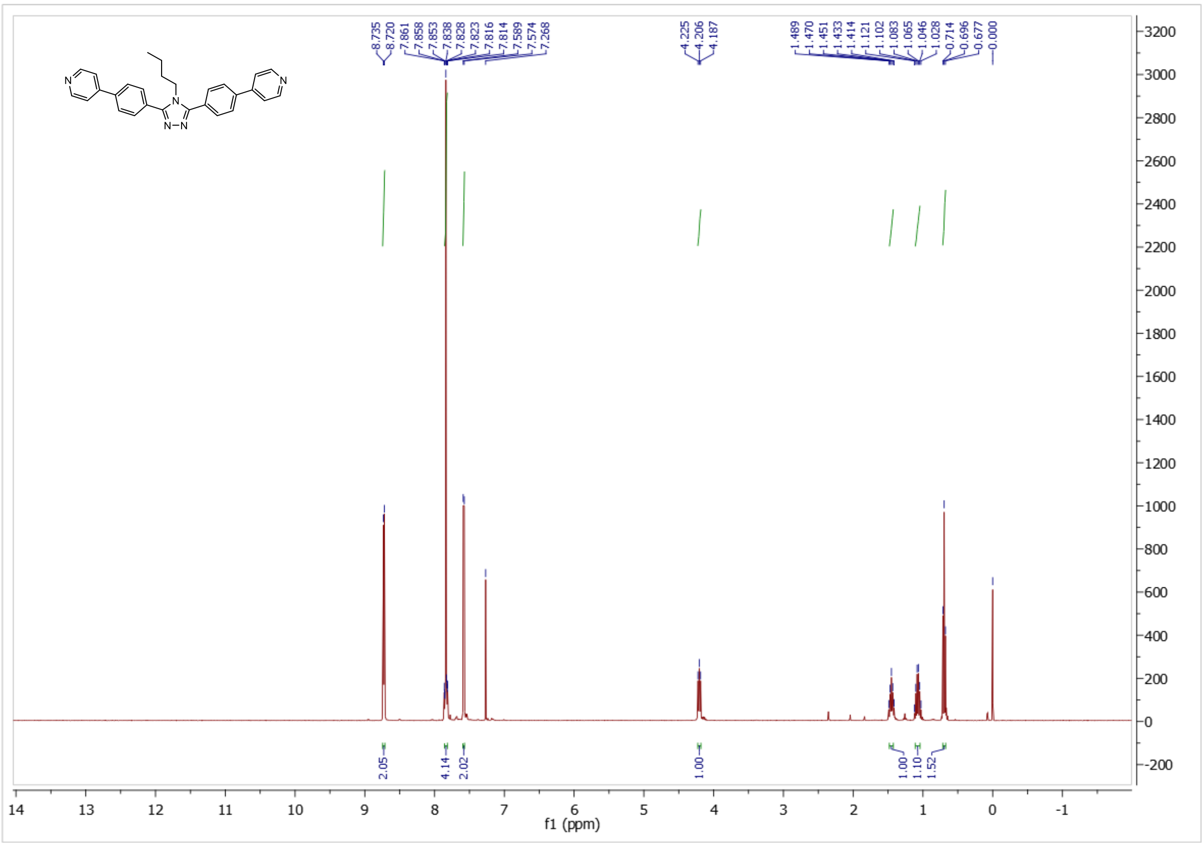

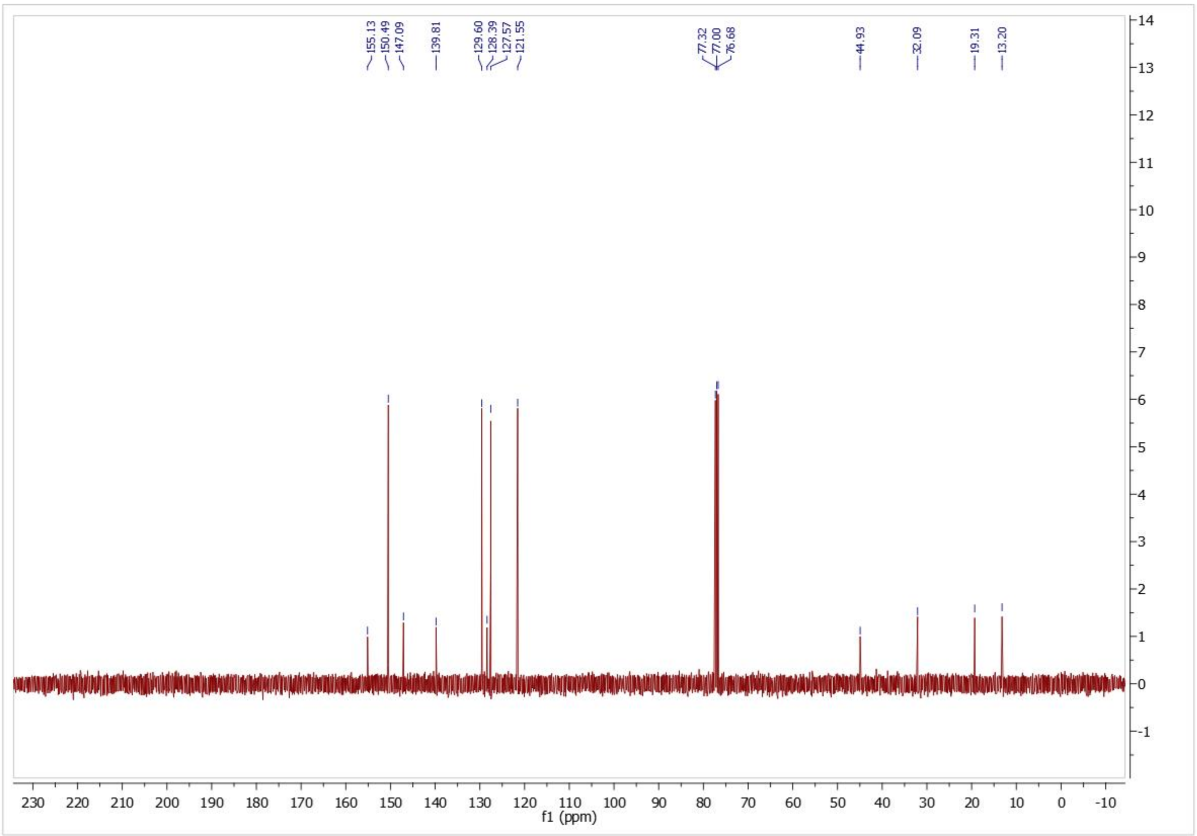

^{1}H and ^{13}C NMR of 4-butyl-3,5-bis[4-(pyridin-3-yl)phenyl]-4*H*-1,2,4-triazole **(7j)**

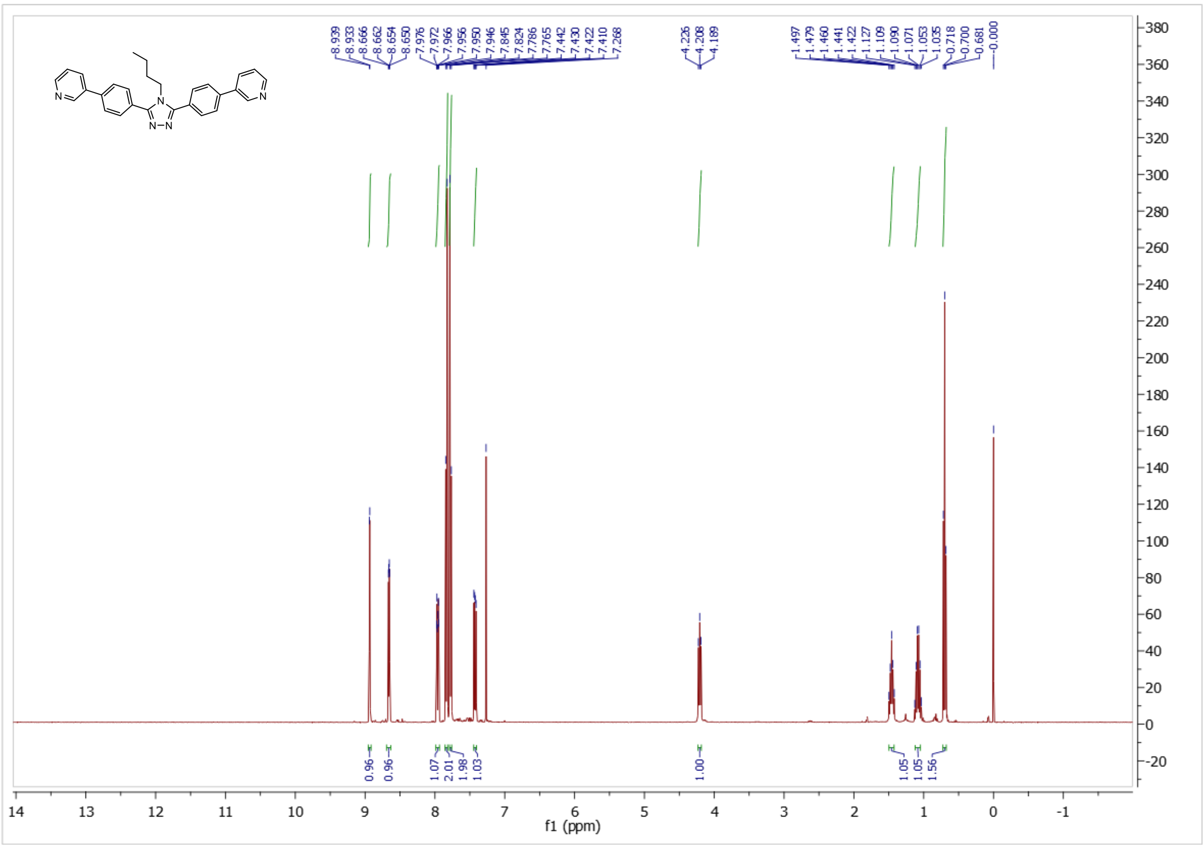

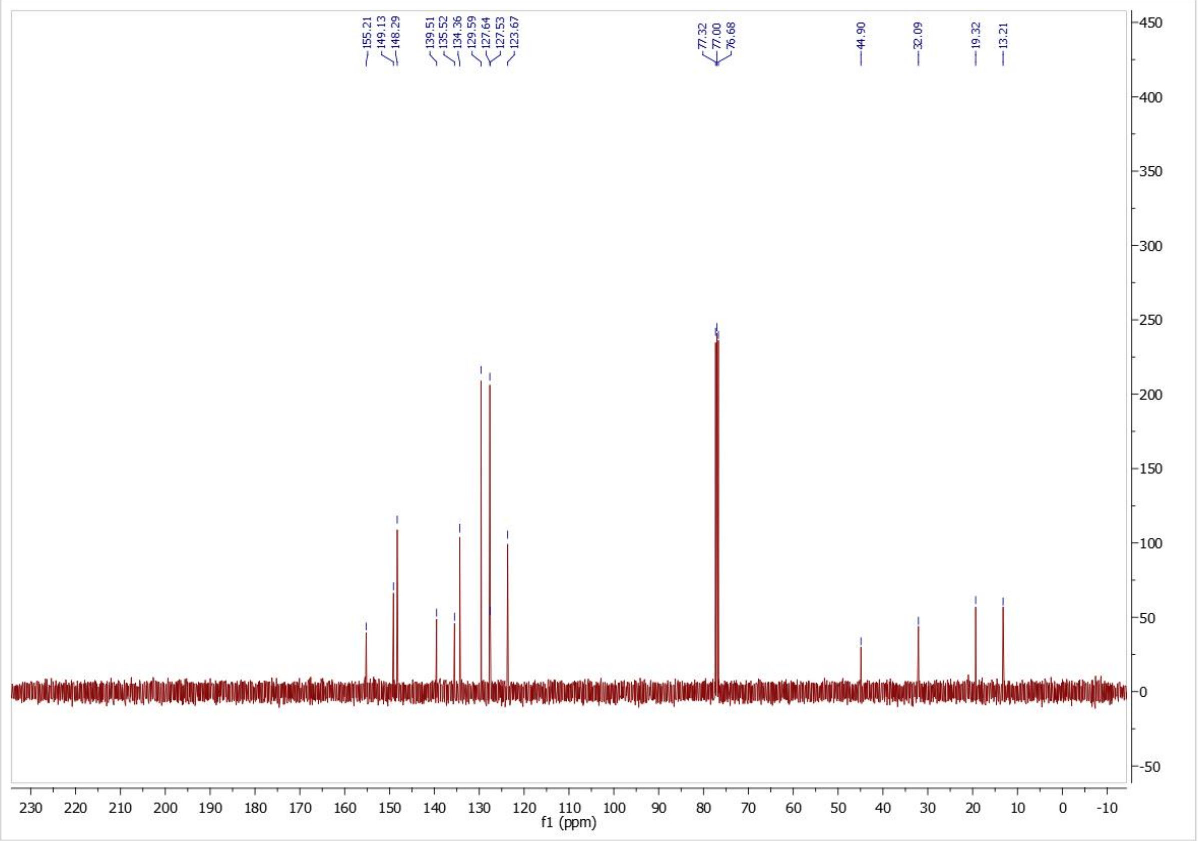

^{1}H and ^{13}C NMR of 4-butyl-3,5-bis[4-(furan-2-yl)phenyl]-4*H*-1,2,4-triazole **(7k)**

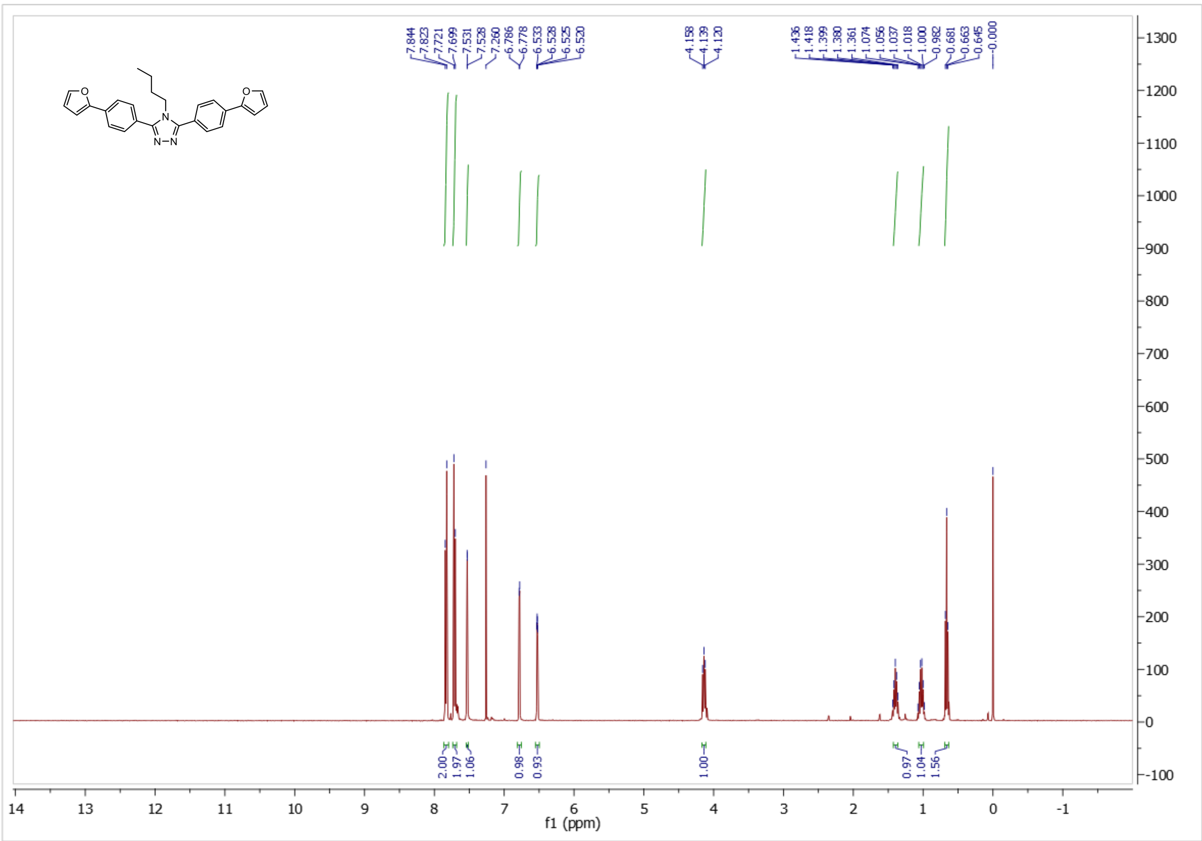

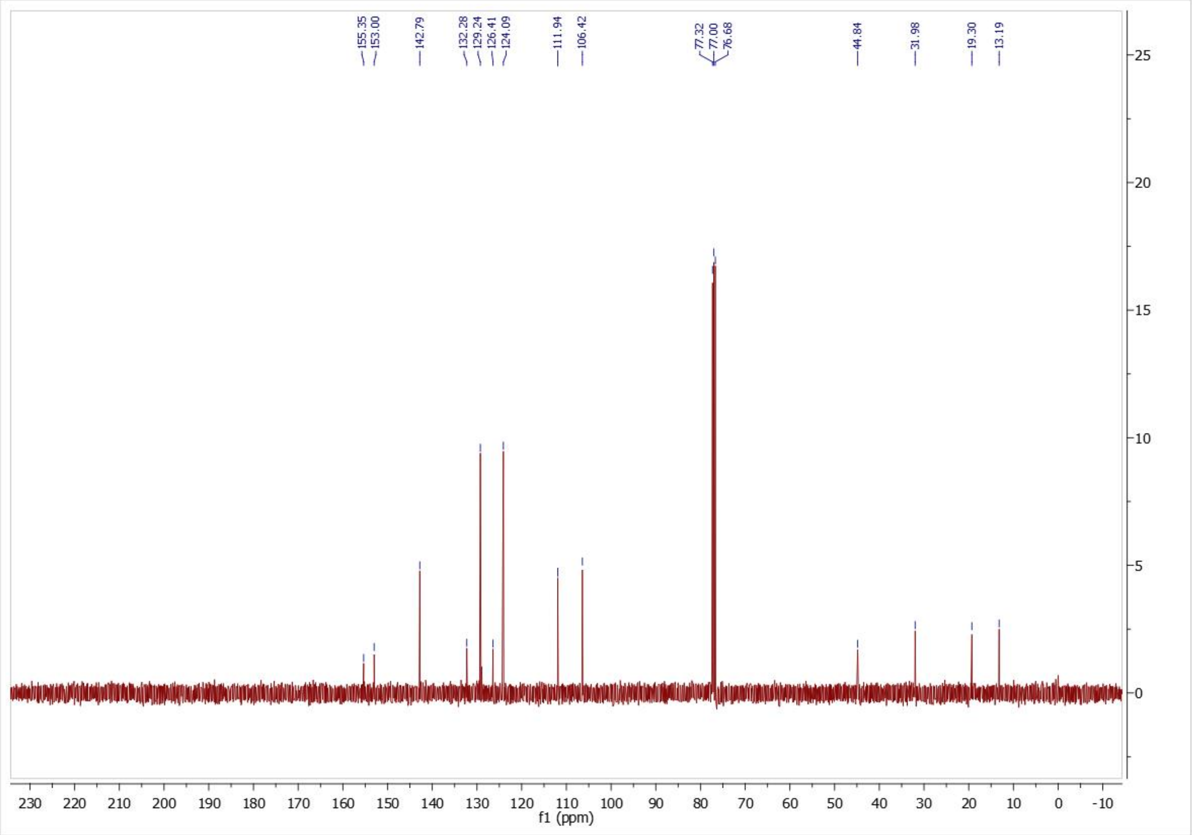

^{1}H and ^{13}C NMR of 4-butyl-3,5-bis[4-(furan-3-yl)phenyl]-4*H*-1,2,4-triazole **(7l)**

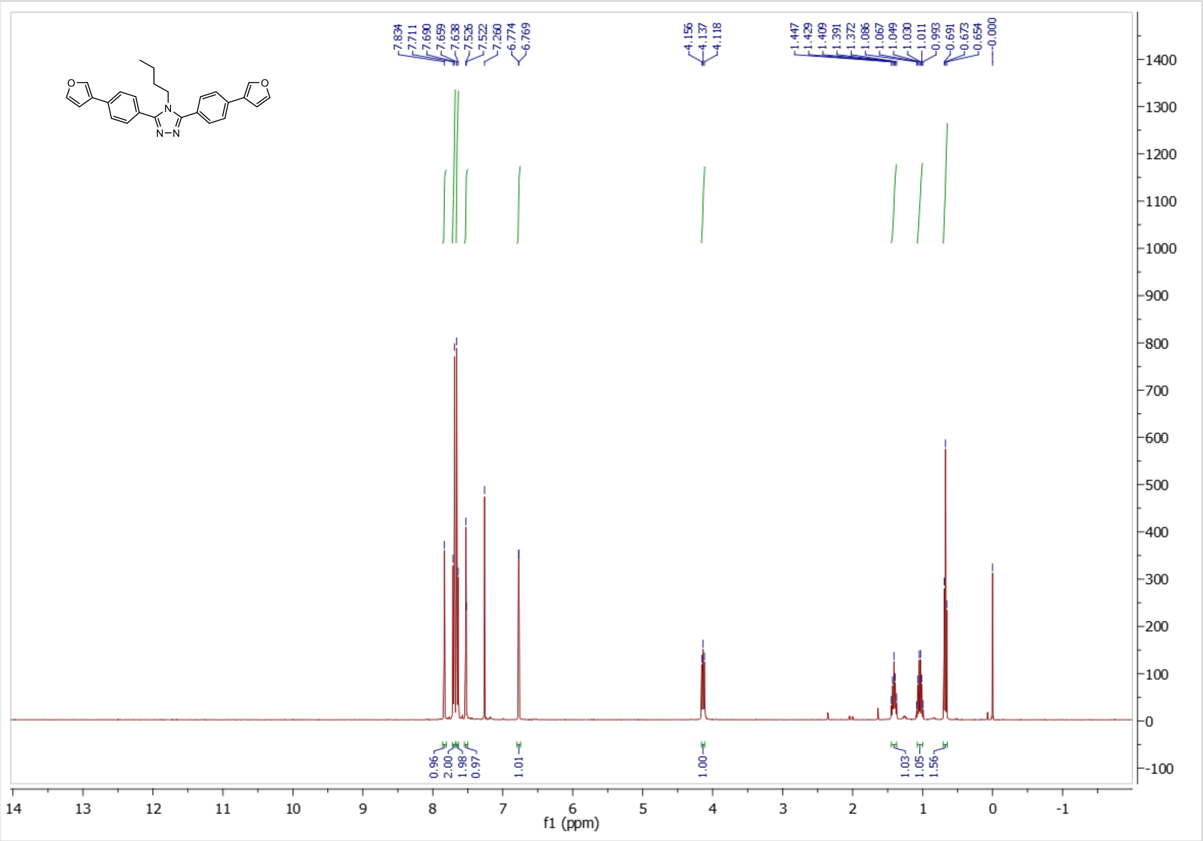

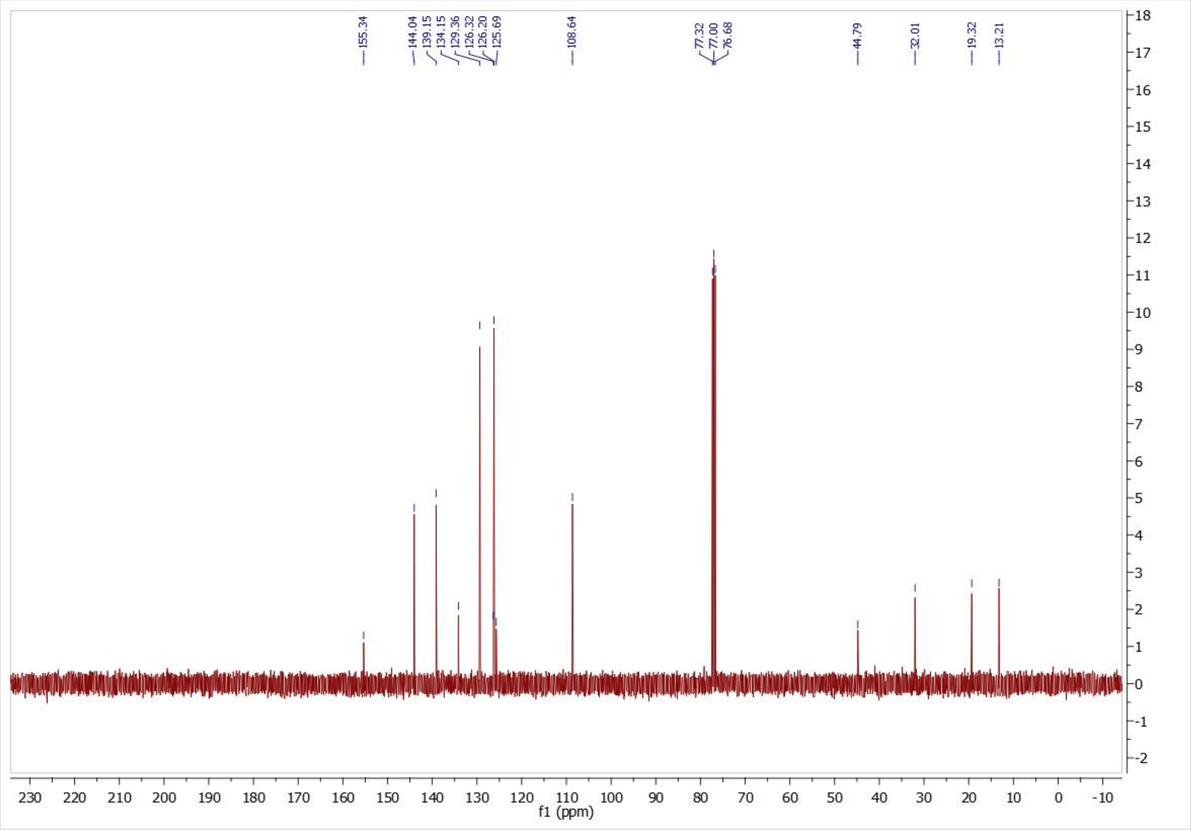

^1H and ^{13}C NMR of 4-butyl-3,5-bis[4-(thiophen-2-yl)phenyl]-4*H*-1,2,4-triazole **(7m)**

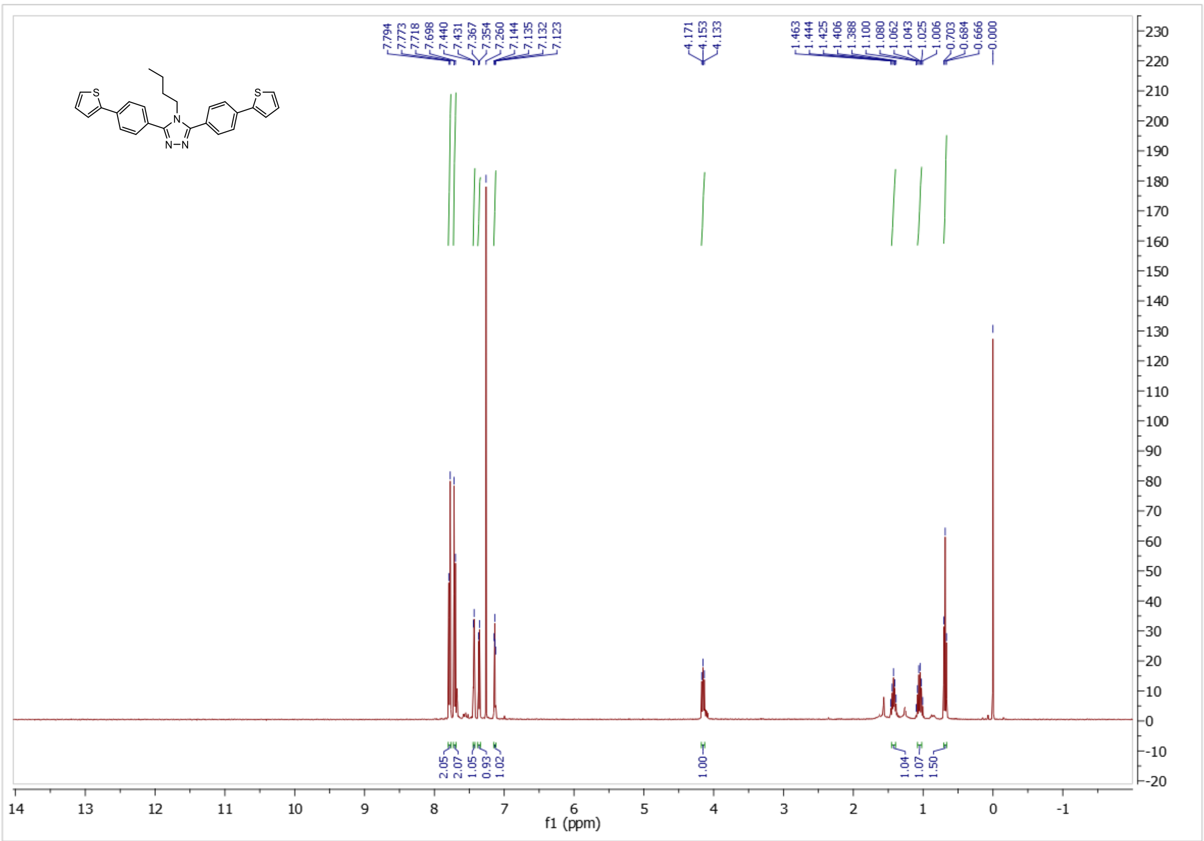

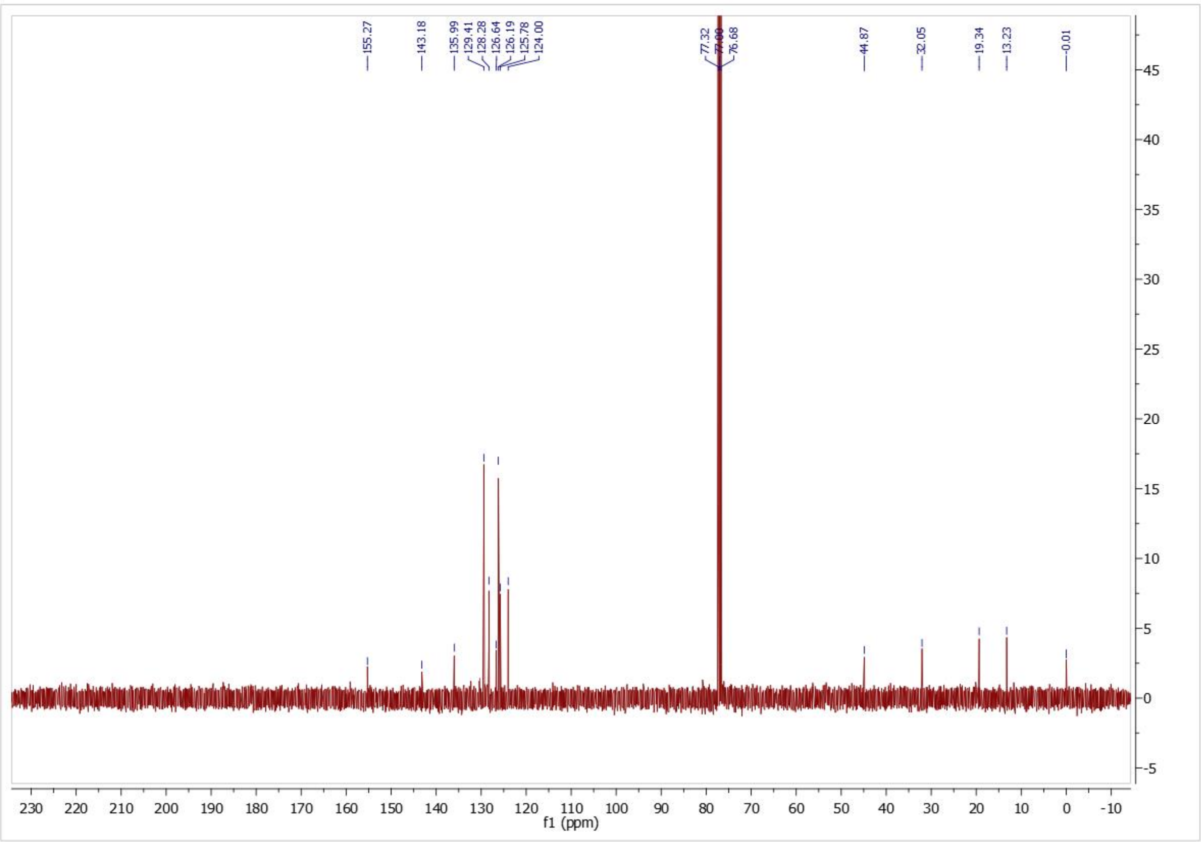

^{1}H and ^{13}C NMR of 4-butyl-3,5-bis[4-(thiophen-3-yl)phenyl]-4*H*-1,2,4-triazole **(7n)**

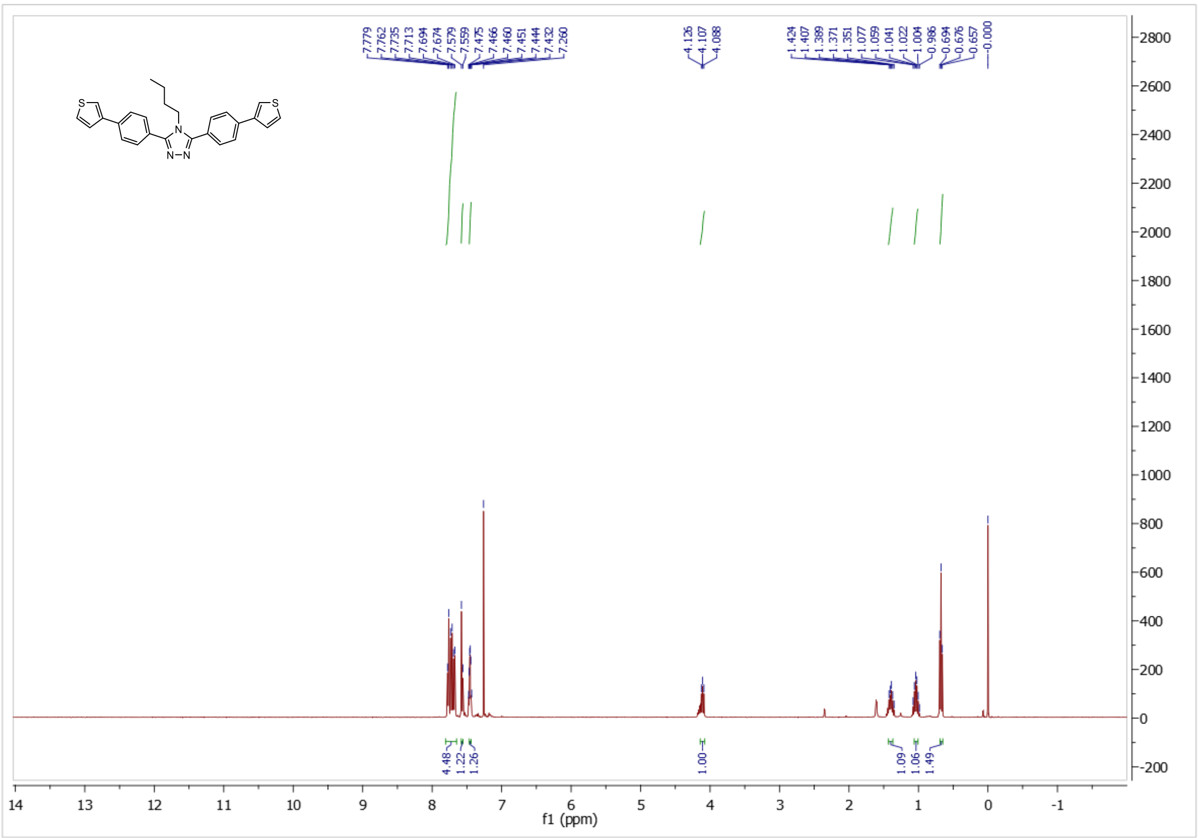

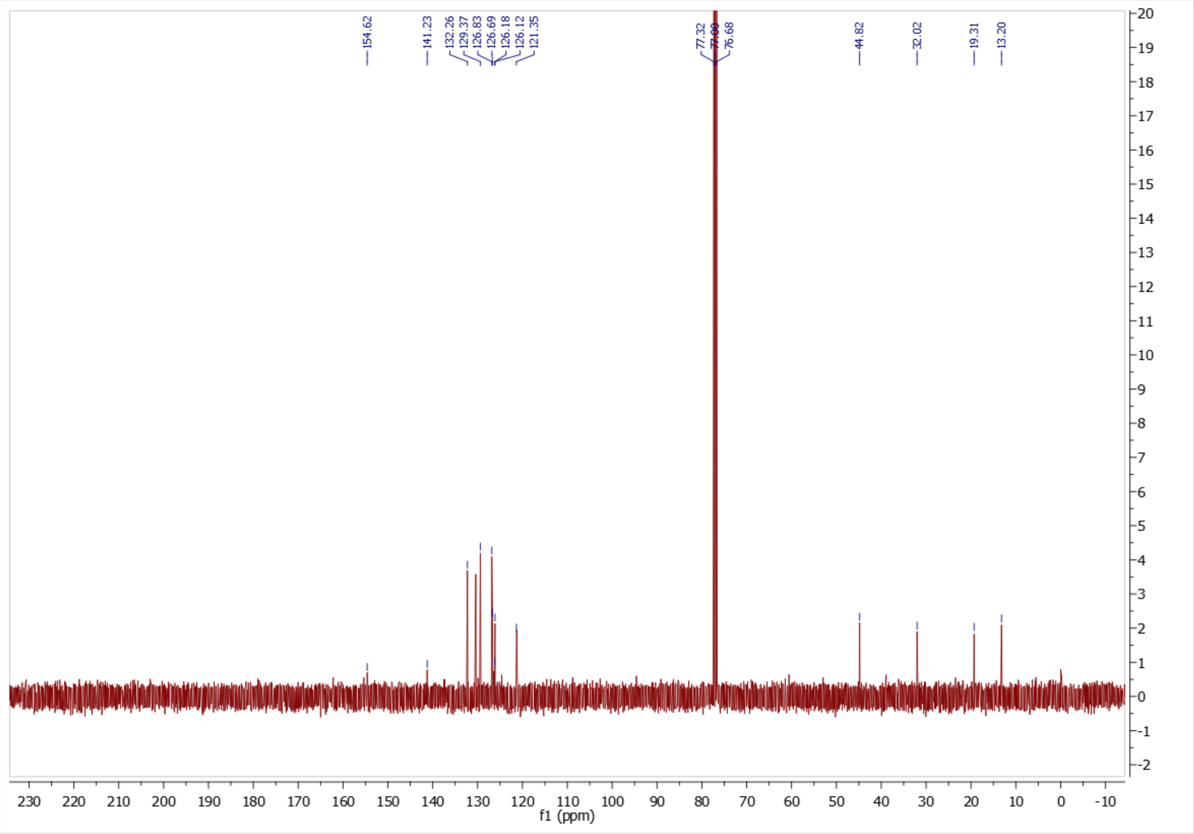

^{1}H and ^{13}C NMR of 3,5-bis(biphenyl-4-yl)-4-hexyl-4*H*-1,2,4-triazole **(8a)**

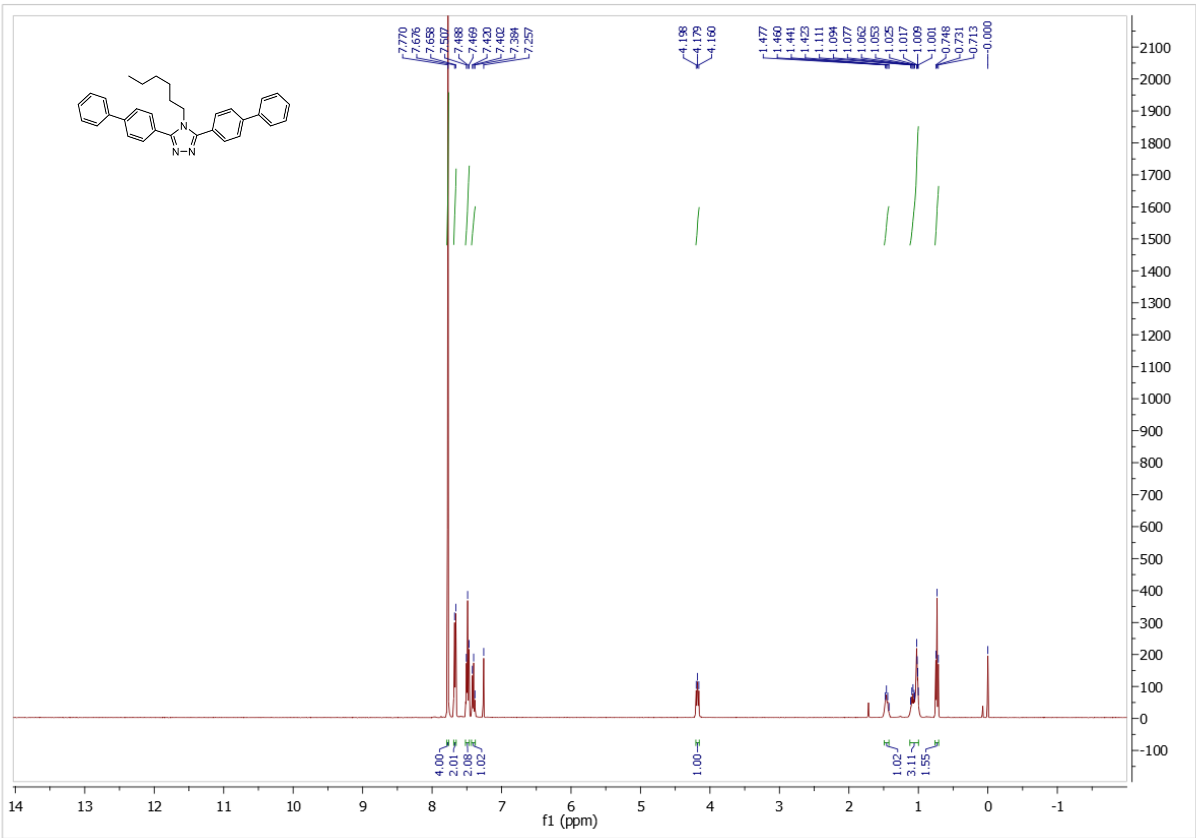

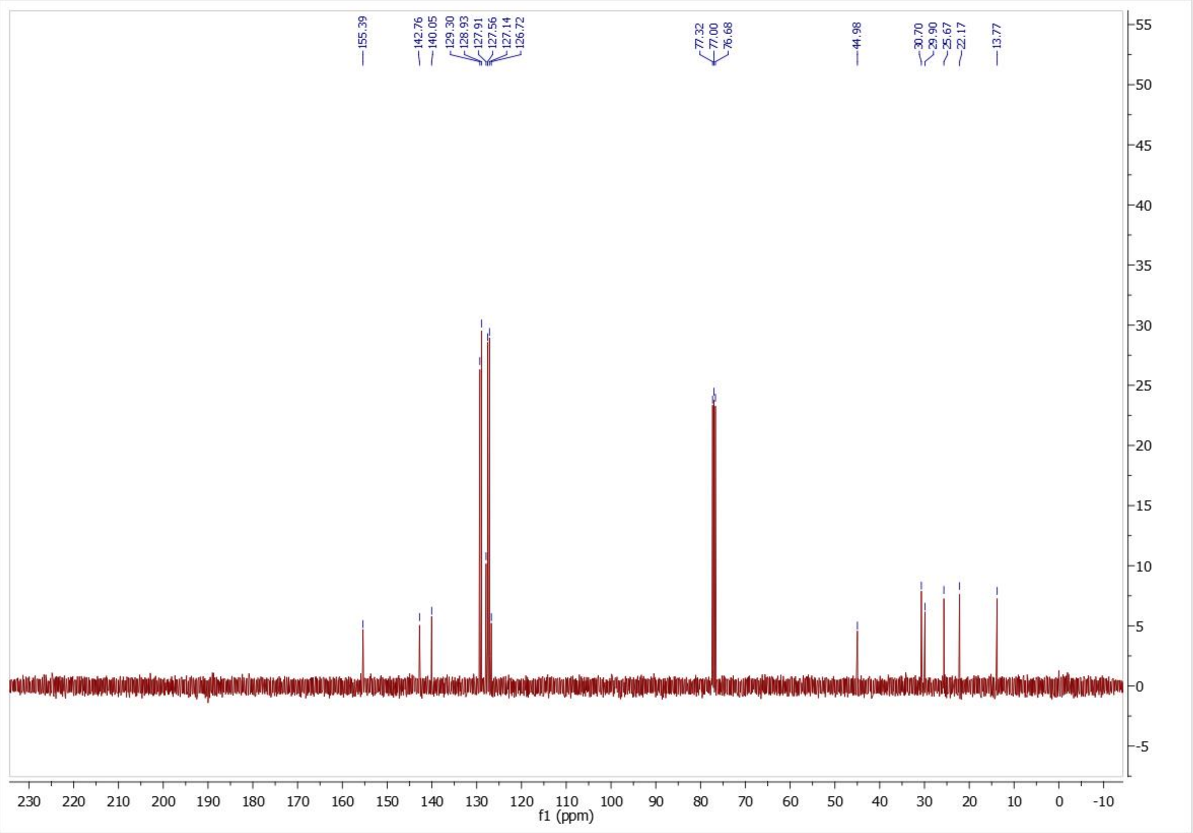

^{1}H and ^{13}C NMR of 4-hexyl-3,5-bis(2'-methylbiphenyl-4-yl)-4*H*-1,2,4-triazole **(8b)**

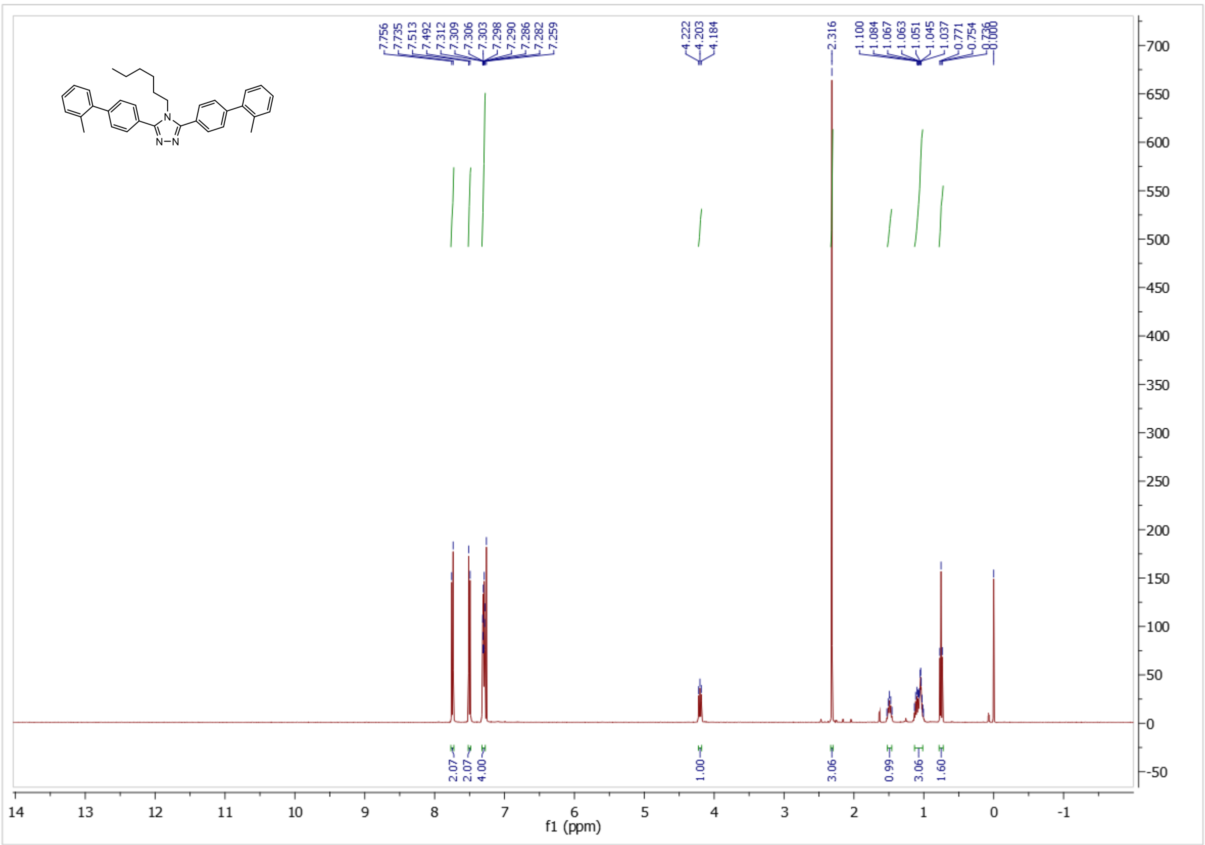

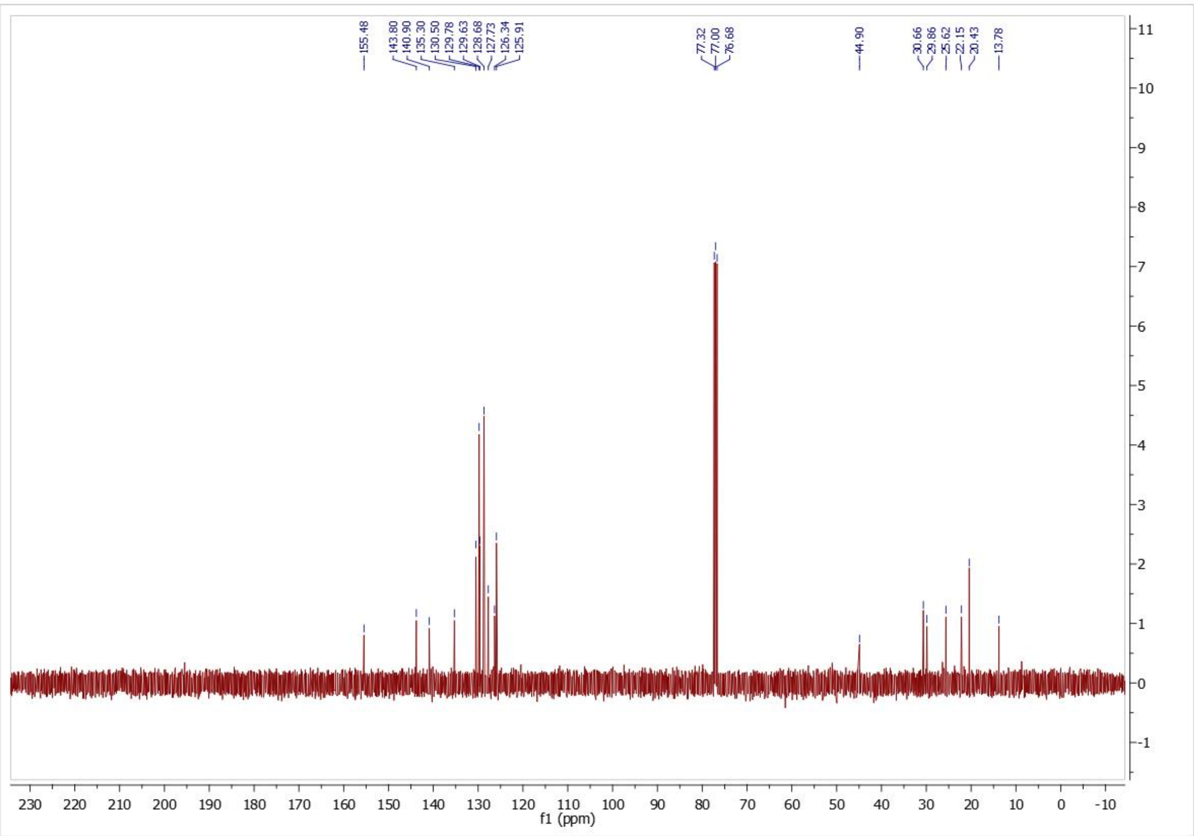

^{1}H and ^{13}C NMR of 4-hexyl-3,5-bis(3’-methylbiphenyl-4-yl)-4*H*-1,2,4-triazole **(8c)**

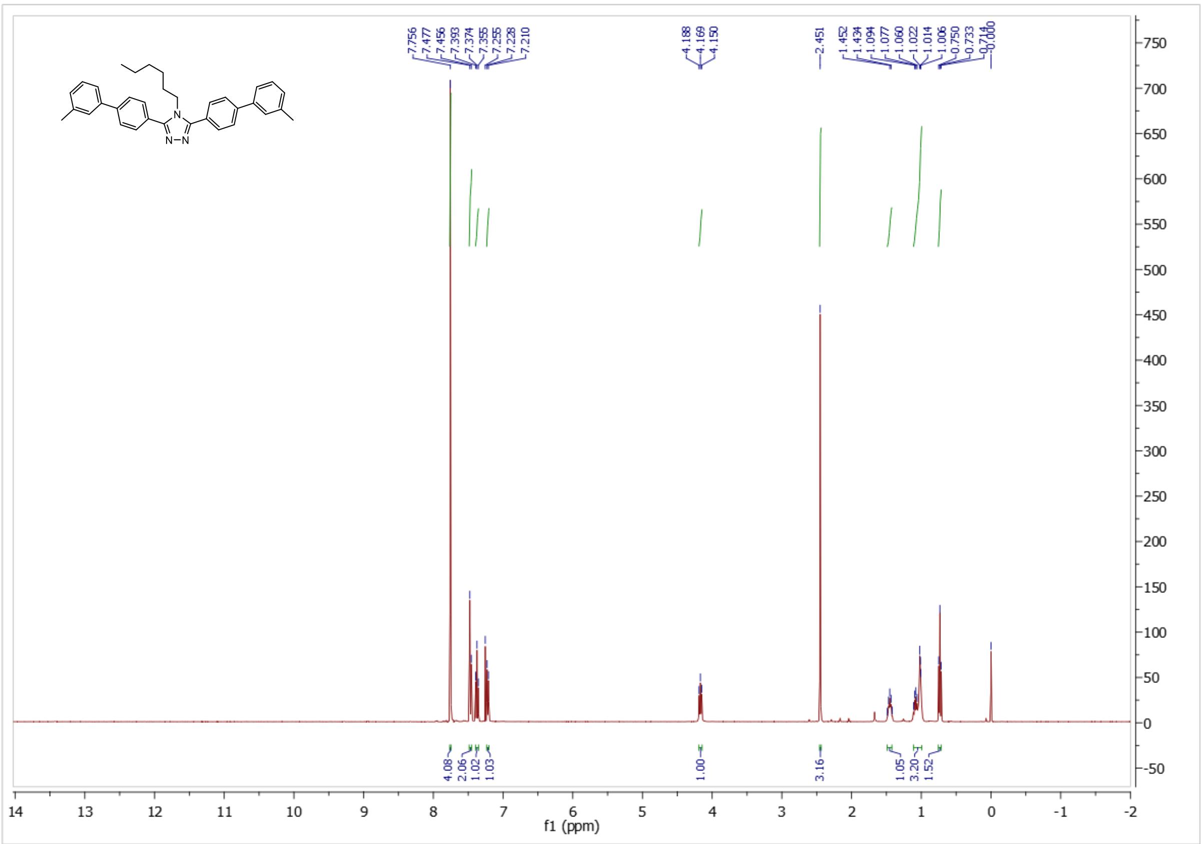

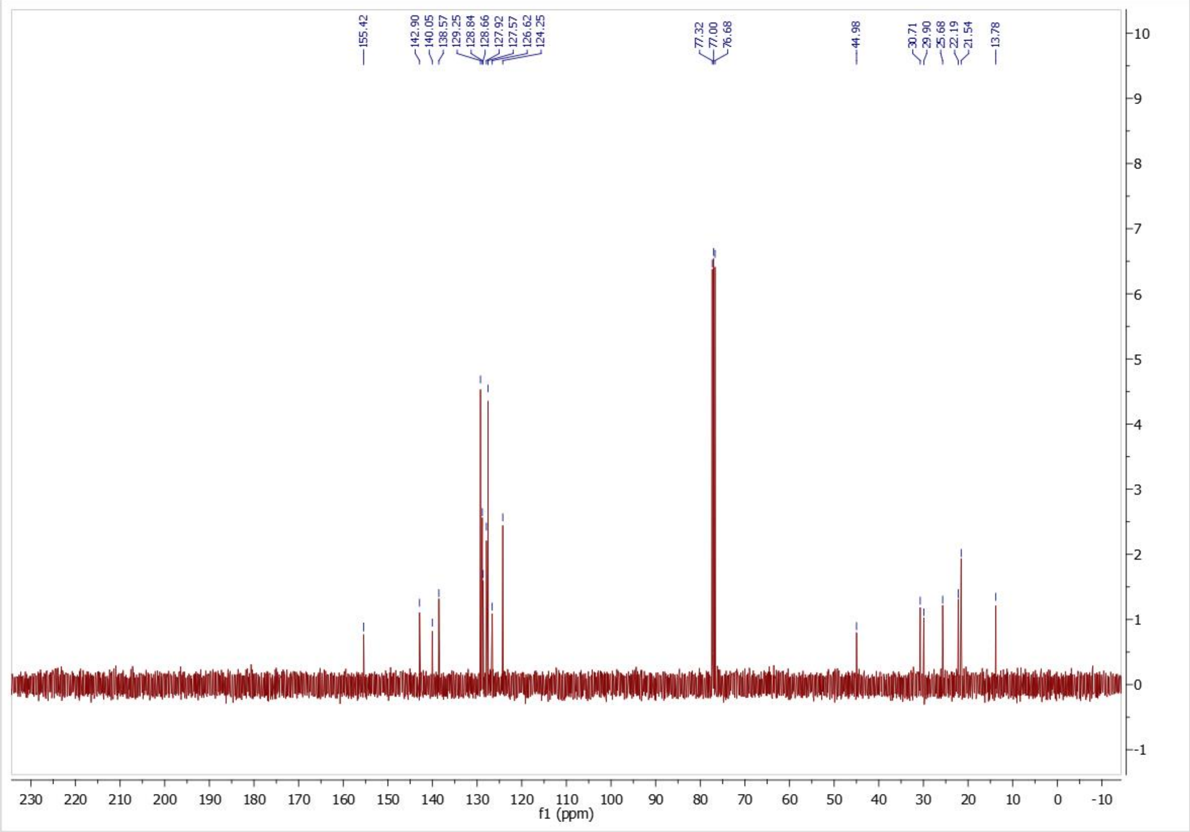

^{1}H and ^{13}C NMR of 4-hexyl-3,5-bis(2',6'-dimethylbiphenyl-4-yl)-4*H*-1,2,4-triazole **(8d)**

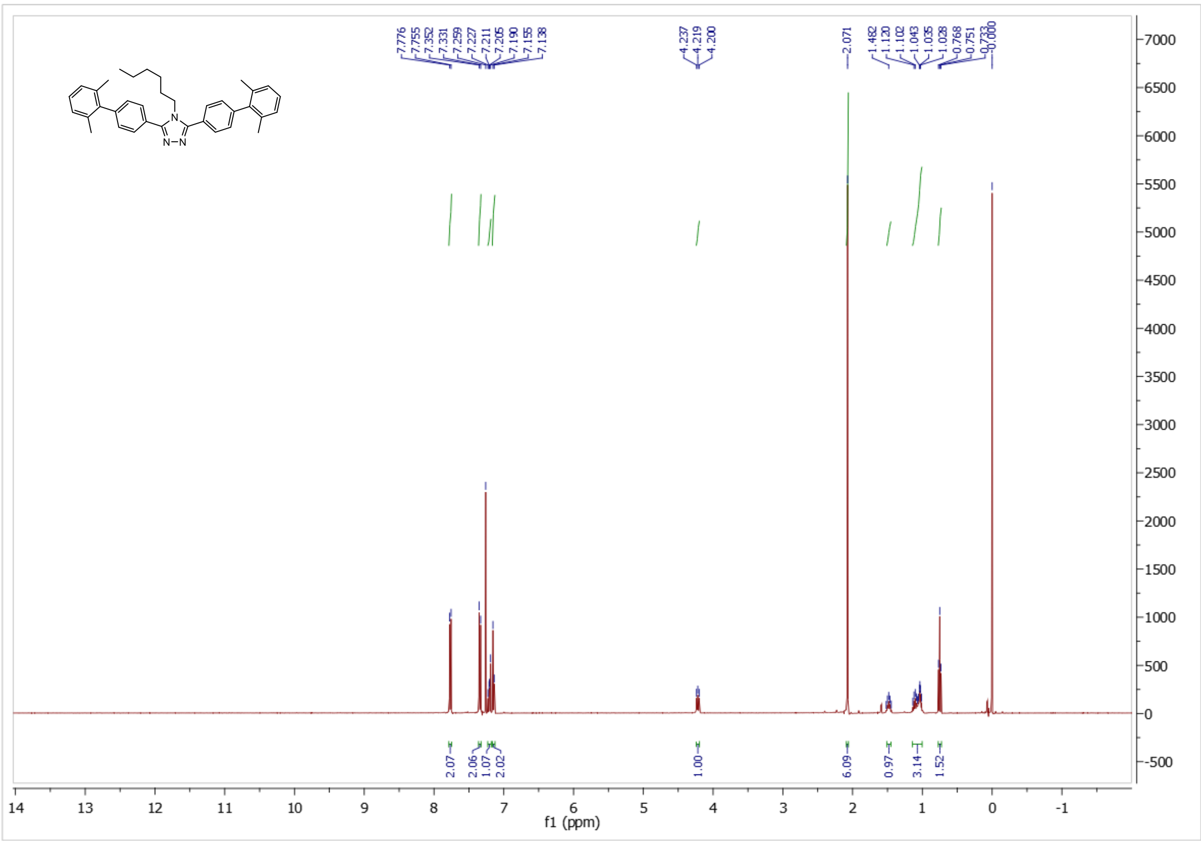

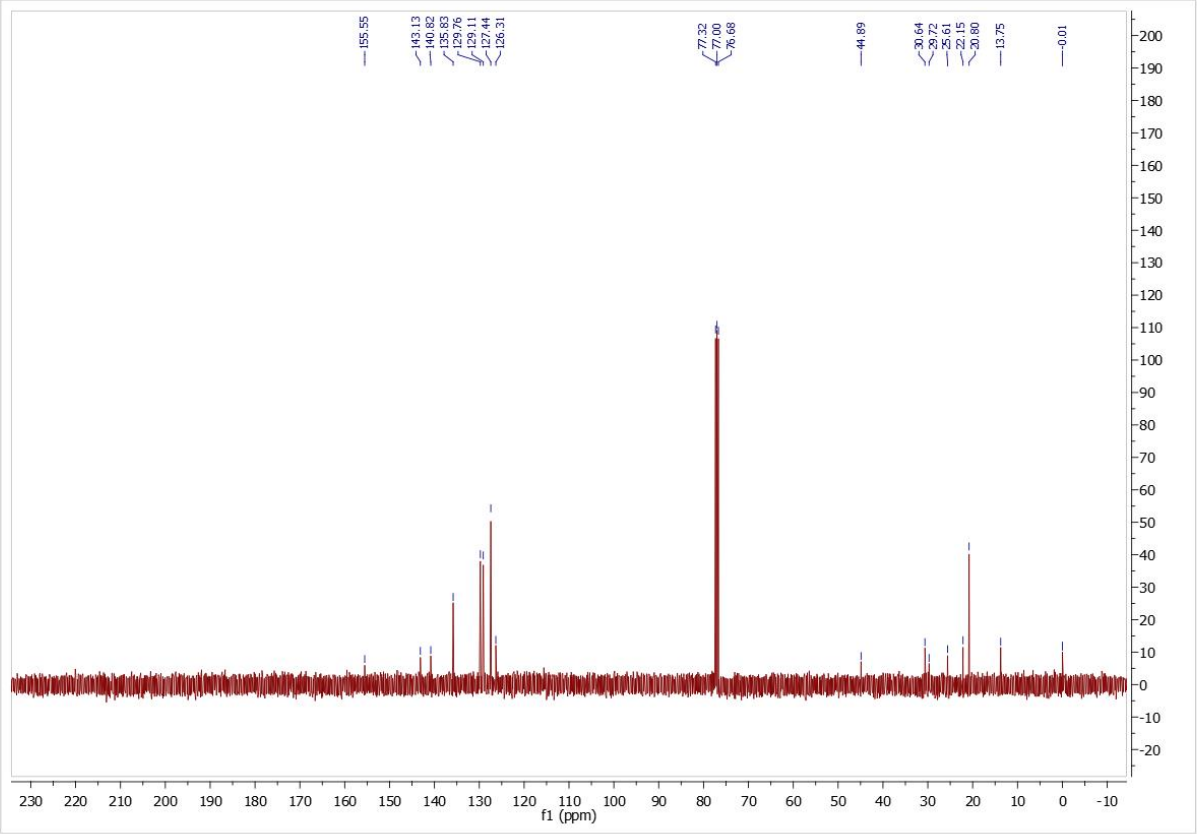

^{1}H and ^{13}C NMR of 4-hexyl-3,5-bis(2'-methoxybiphenyl-4-yl)-4*H*-1,2,4-triazole **(8e)**

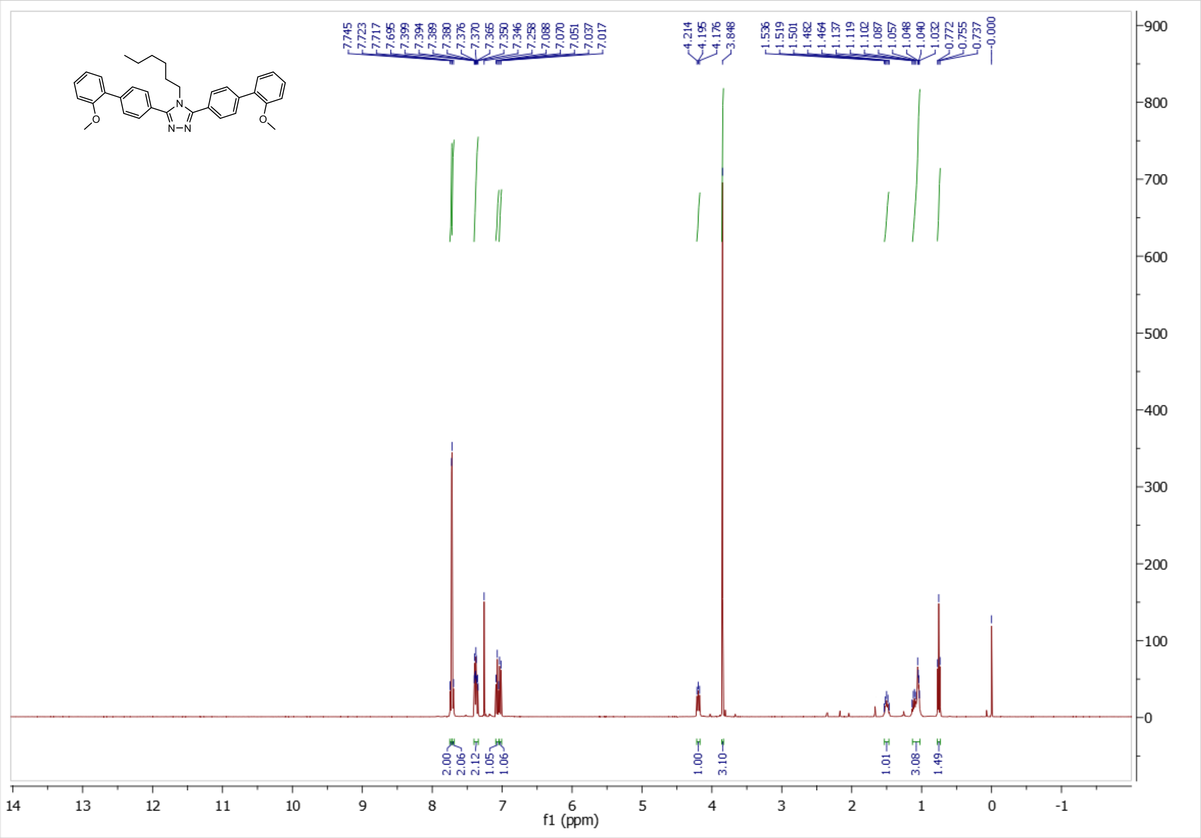

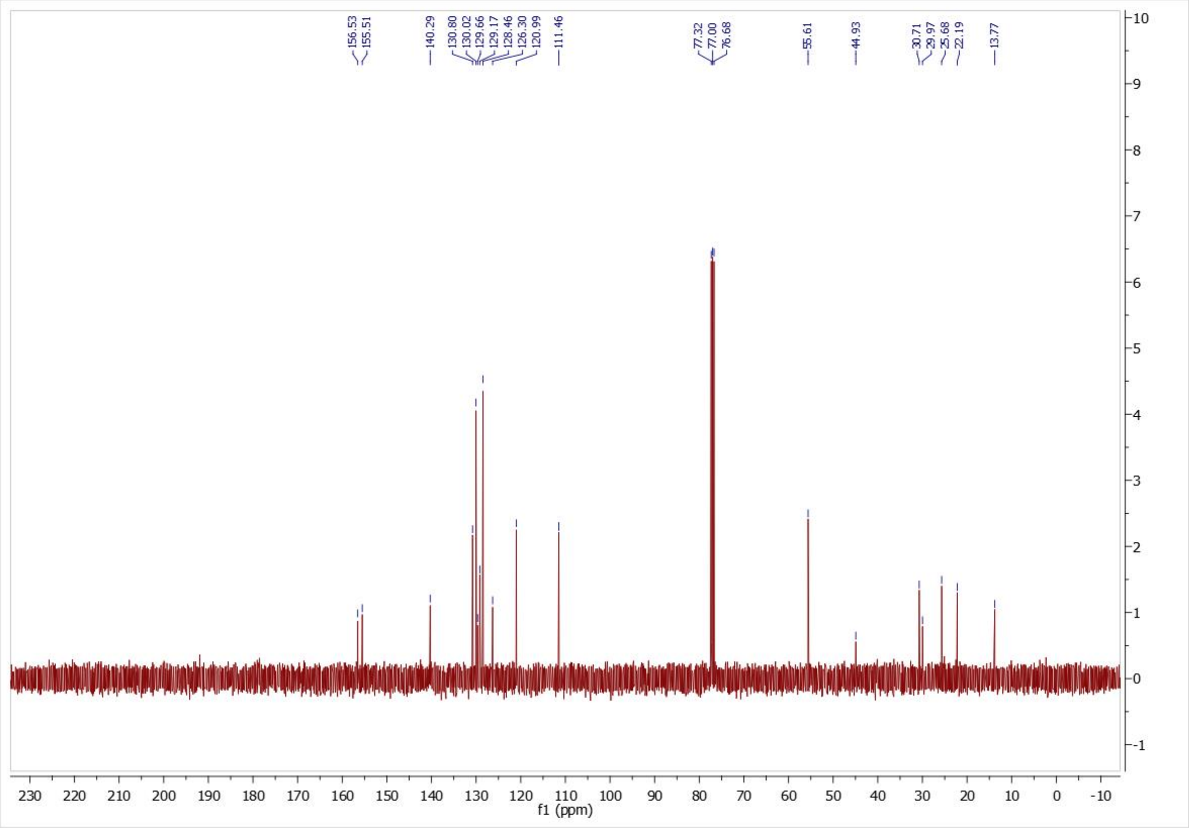

^{1}H and ^{13}C NMR of 4-hexyl-3,5-bis(3’-methoxybiphenyl-4-yl)-4*H*-1,2,4-triazole **(8f)**

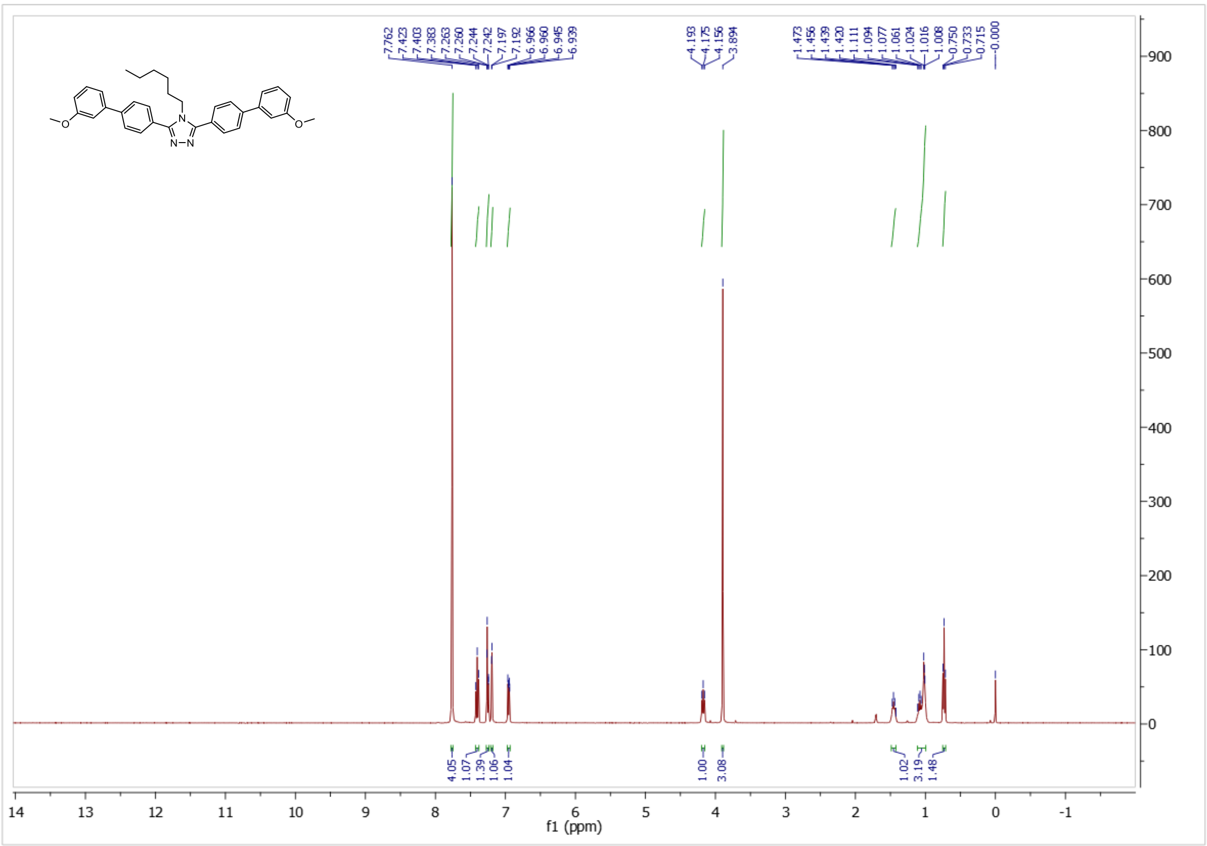

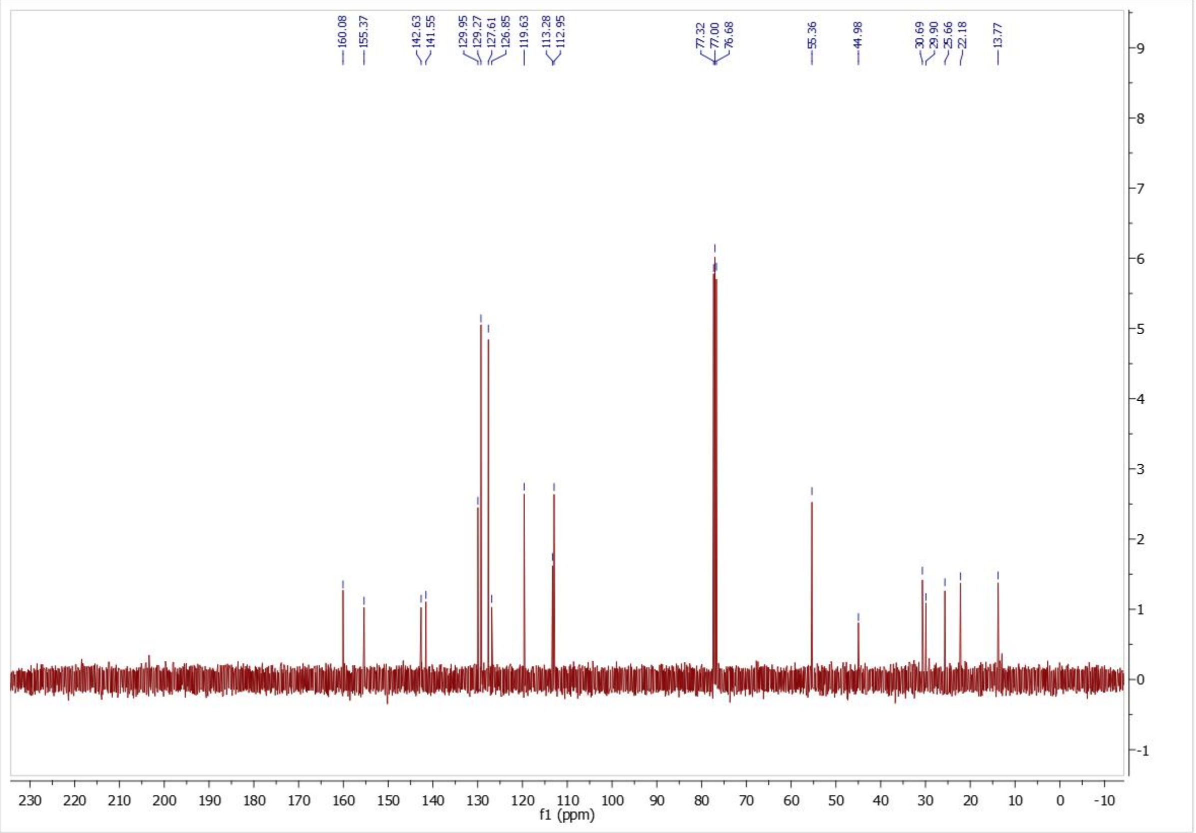

^{1}H and ^{13}C NMR of 4-hexyl-3,5-bis(3'-nitrobiphenyl-4-yl)-4*H*-1,2,4-triazole **(8g)**

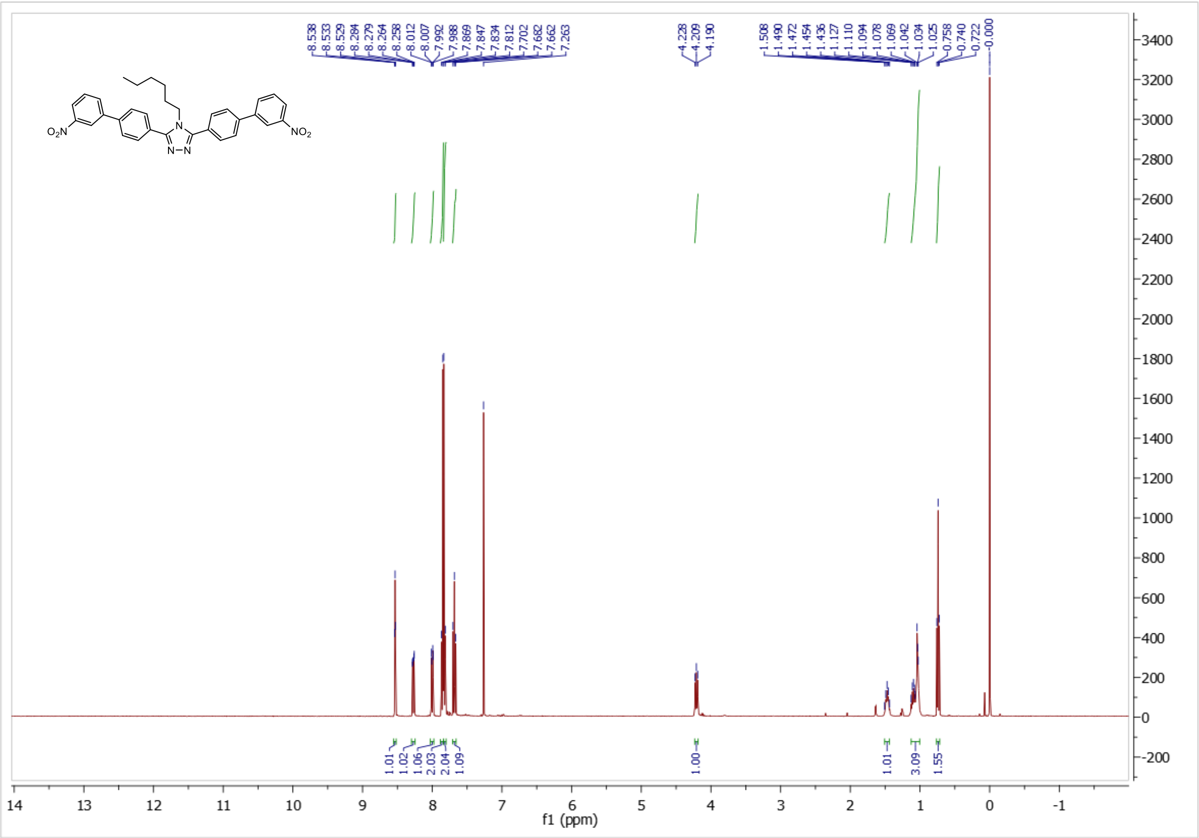

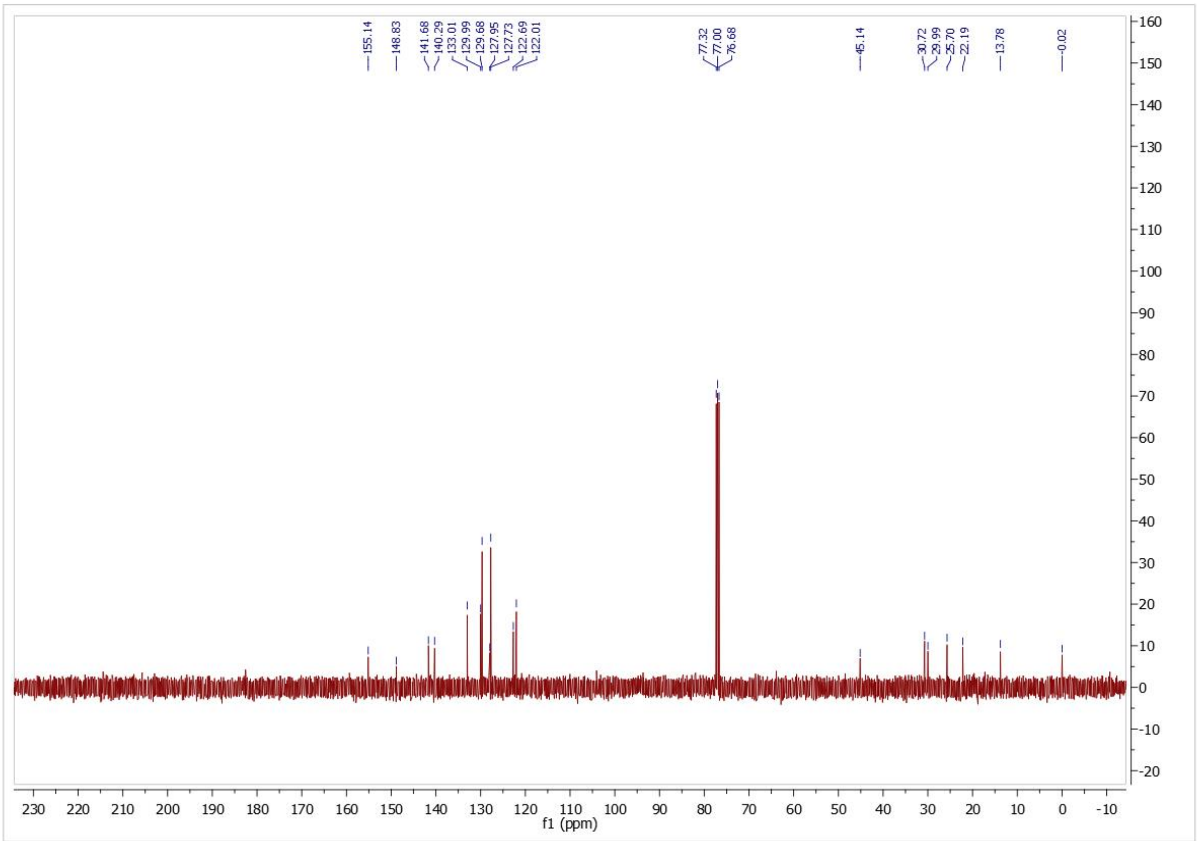

^{1}H and ^{13}C NMR of 3,5-bis(3'-aminobiphenyl-4-yl)-4*H*-1,2,4-triazole **(8h)**

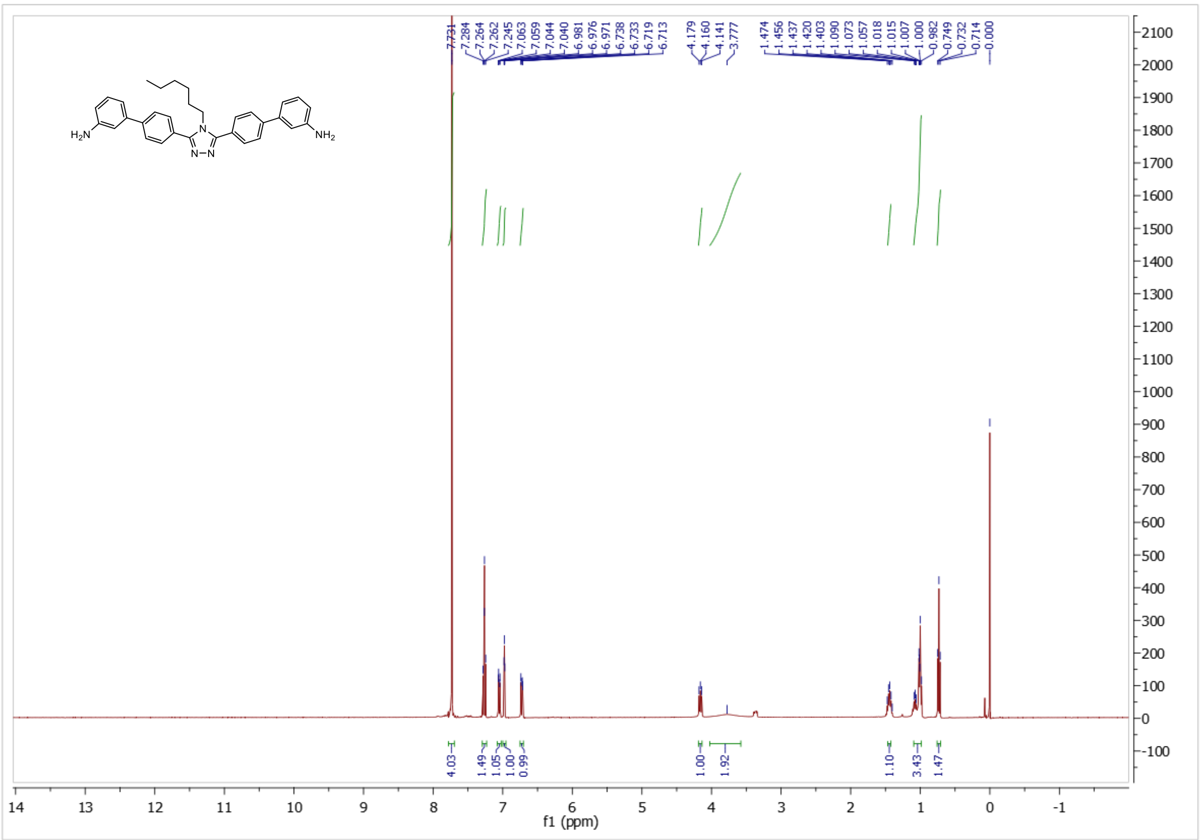

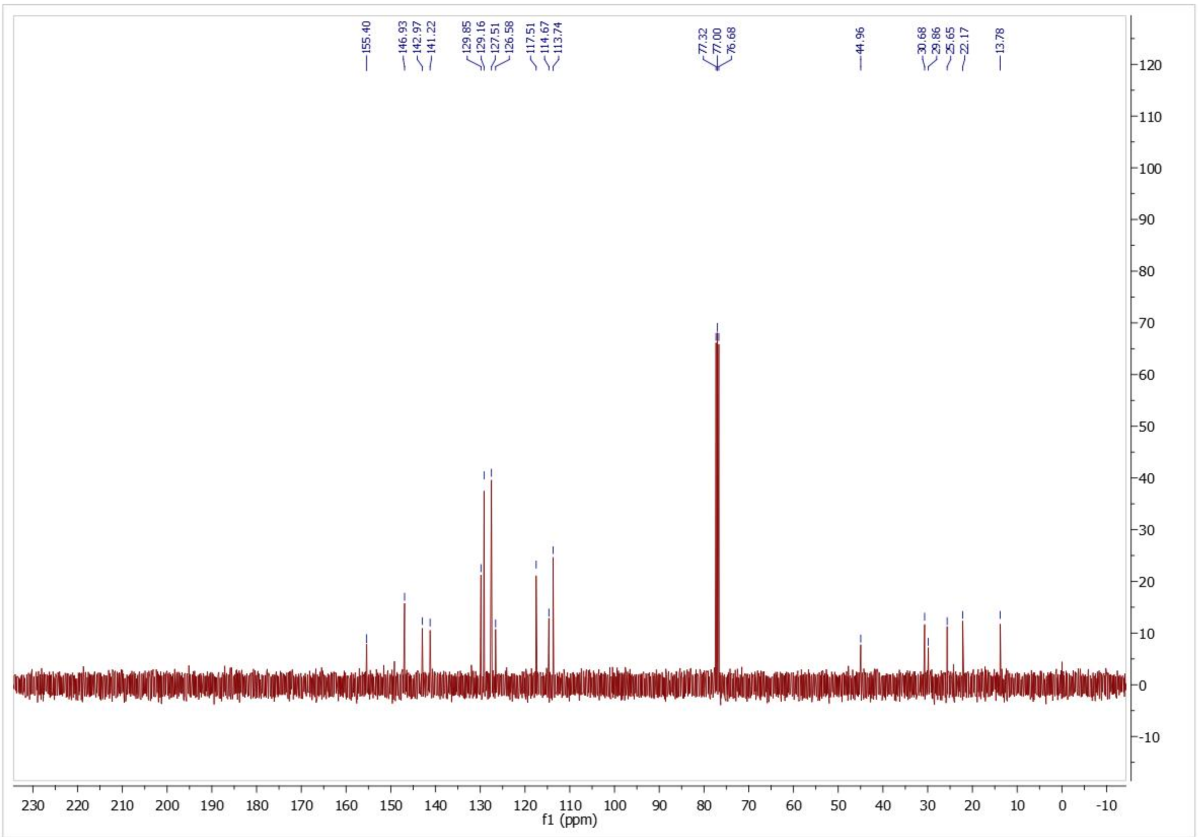

^{1}H and ^{13}C NMR of 4-hexyl-3,5-bis[4-(pyridin-4-yl)phenyl]-4*H*-1,2,4-triazole **(8i)**

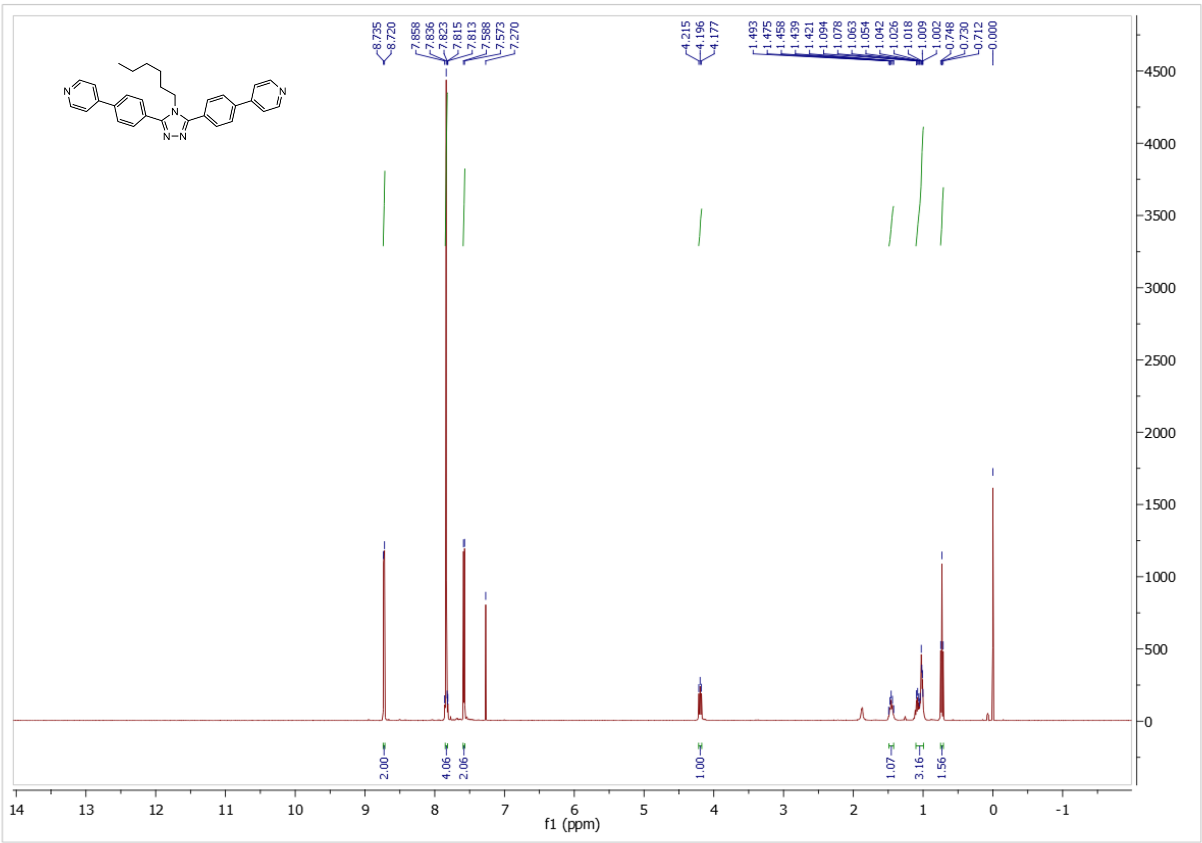

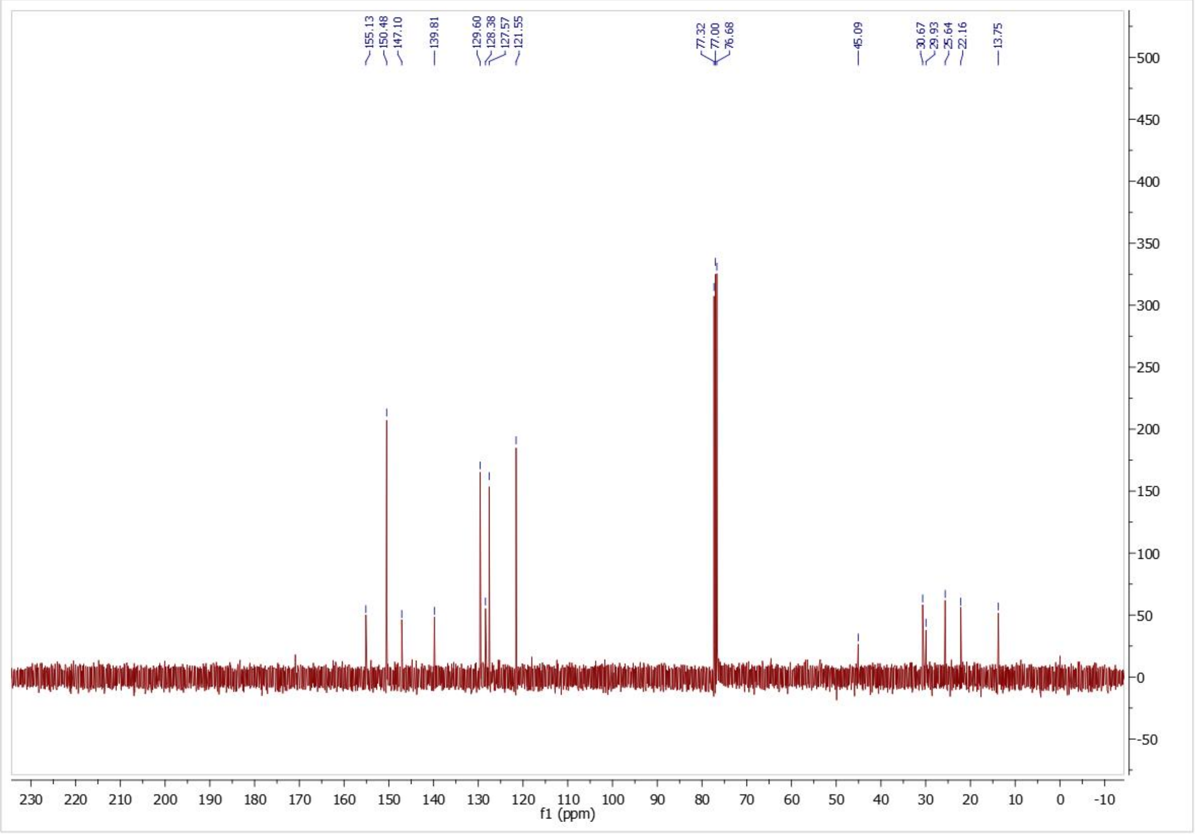

^{1}H and ^{13}C NMR of 4-hexyl-3,5-bis[4-(pyridin-3-yl)phenyl]-4*H*-1,2,4-triazole **(8j)**

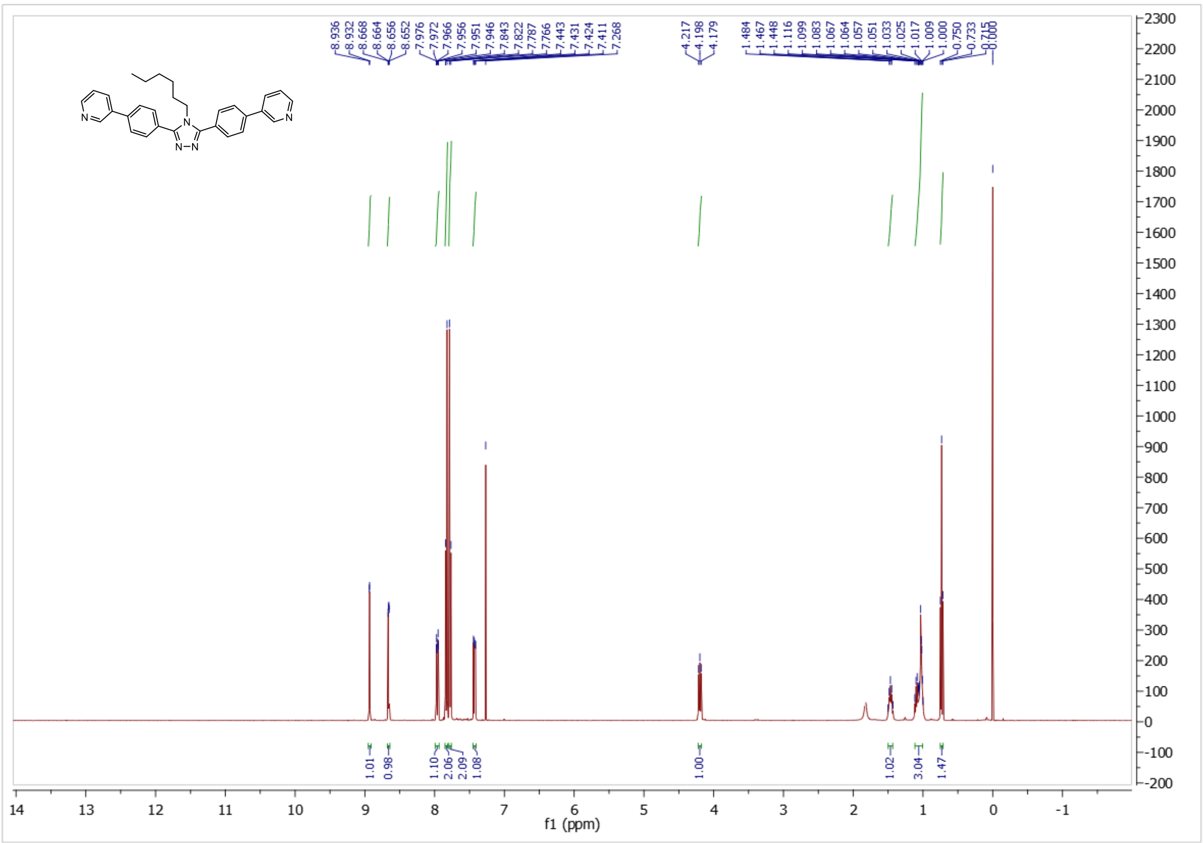

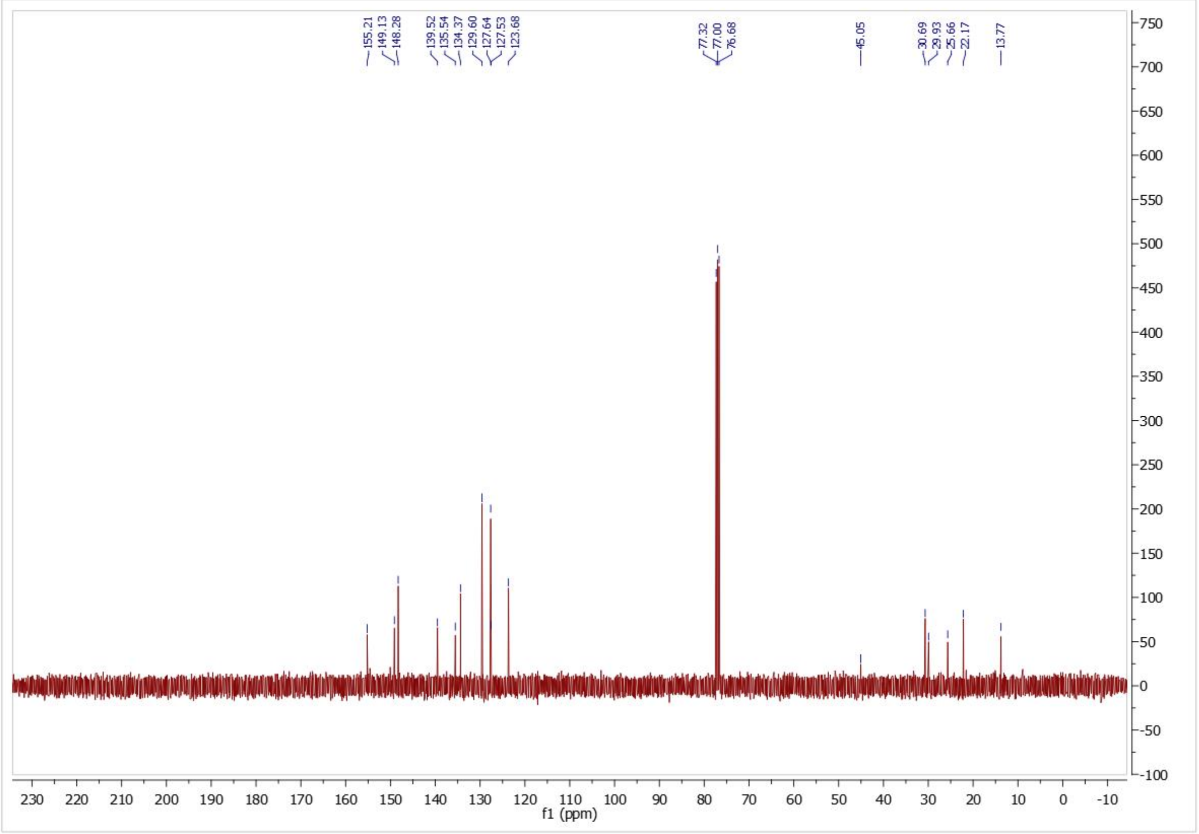

^{1}H and ^{13}C NMR of 3,5-bis[4-(furan-2-yl)phenyl]-4-hexyl-4*H*-1,2,4-triazole **(8k)**

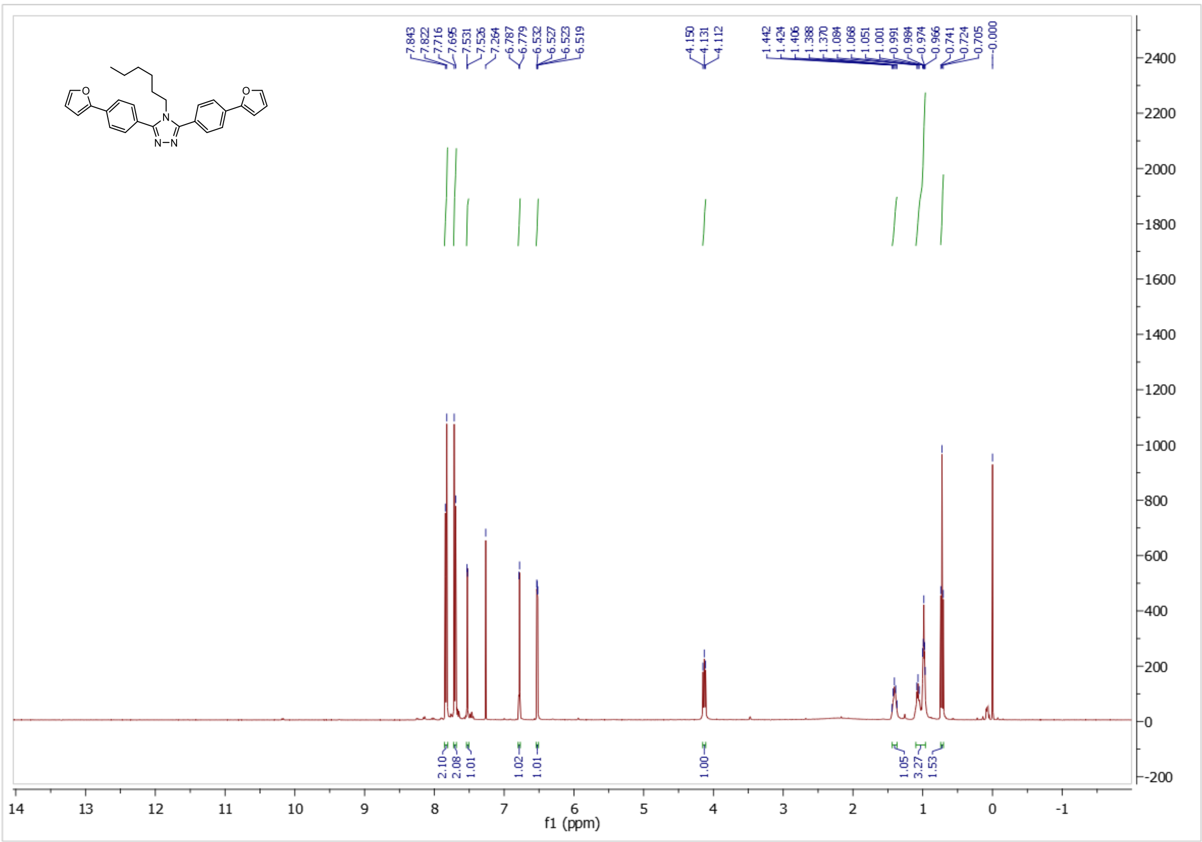

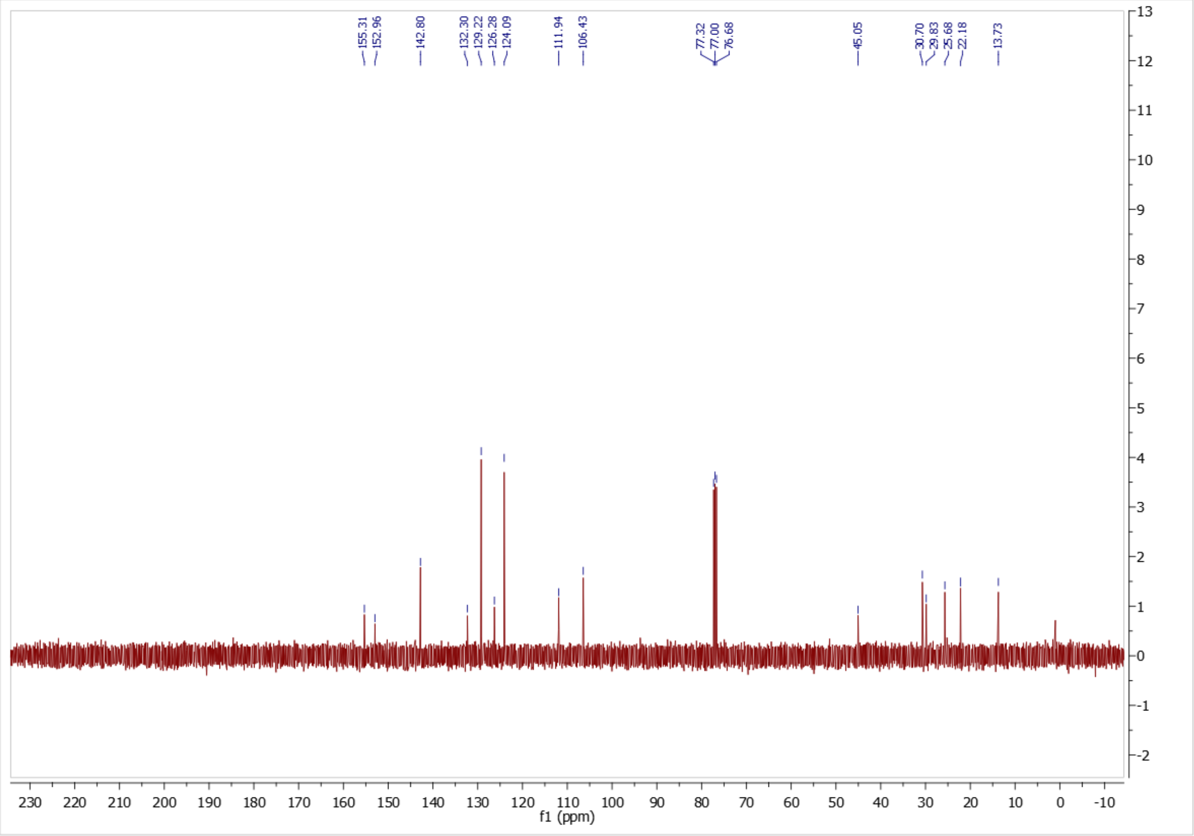

^{1}H and ^{13}C NMR of 3,5-bis[4-(furan-3-yl)phenyl]-4-hexyl-4*H*-1,2,4-triazole **(8l)**

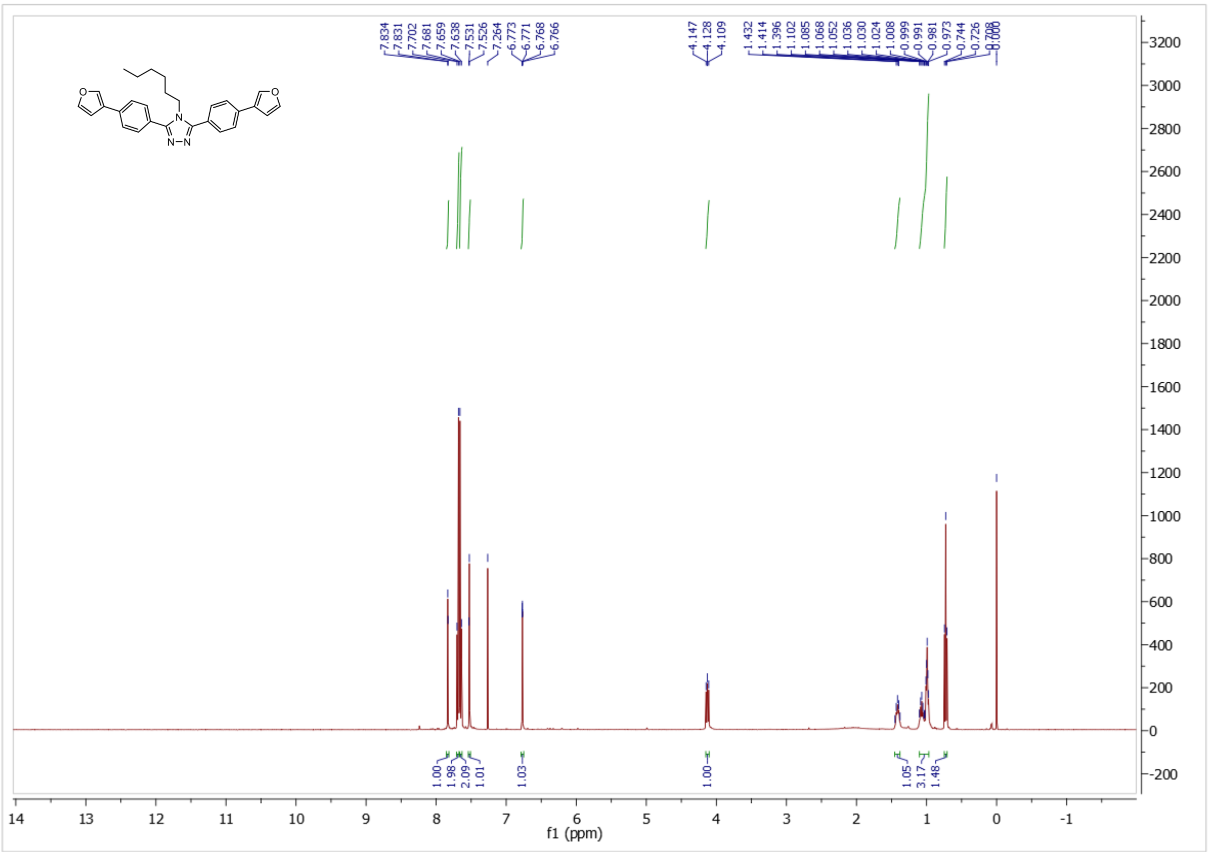

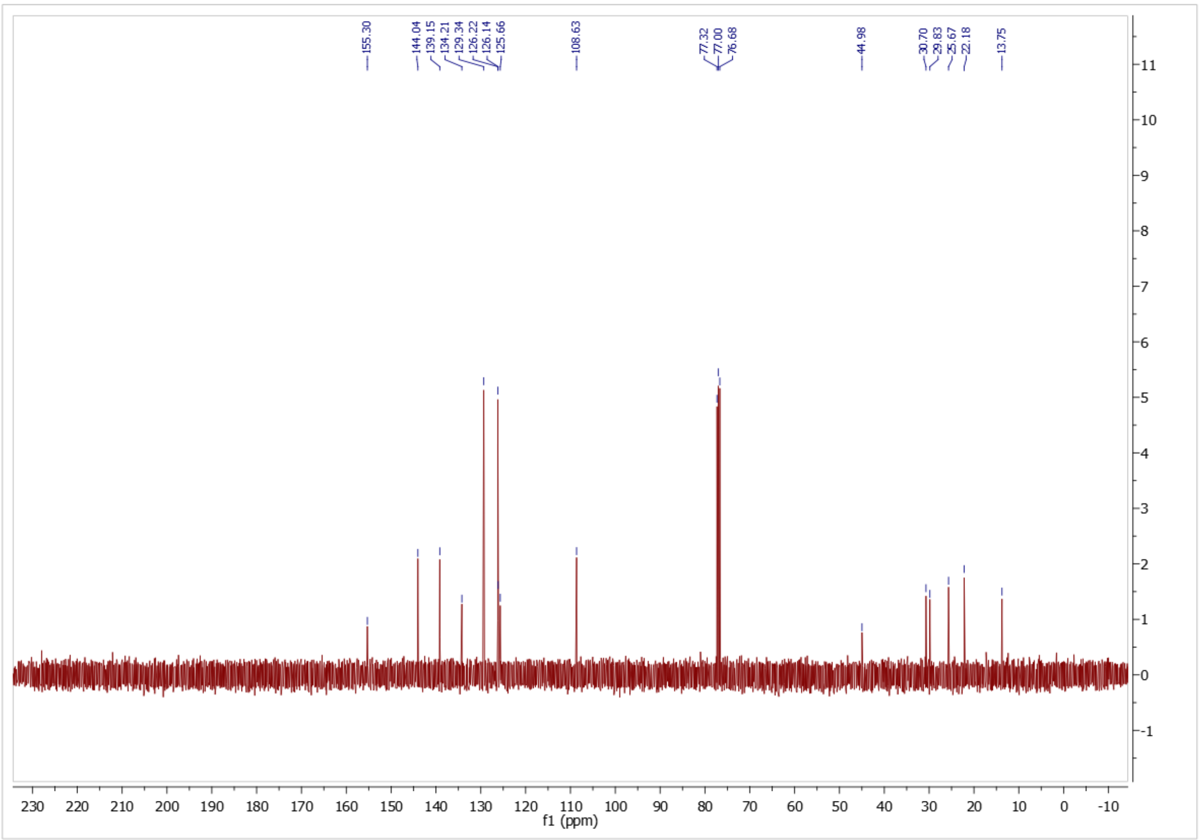

^{1}H and ^{13}C NMR of 4-hexyl-3,5-bis[4-(thiophen-2-yl)phenyl]-4*H*-1,2,4-triazole **(8m)**

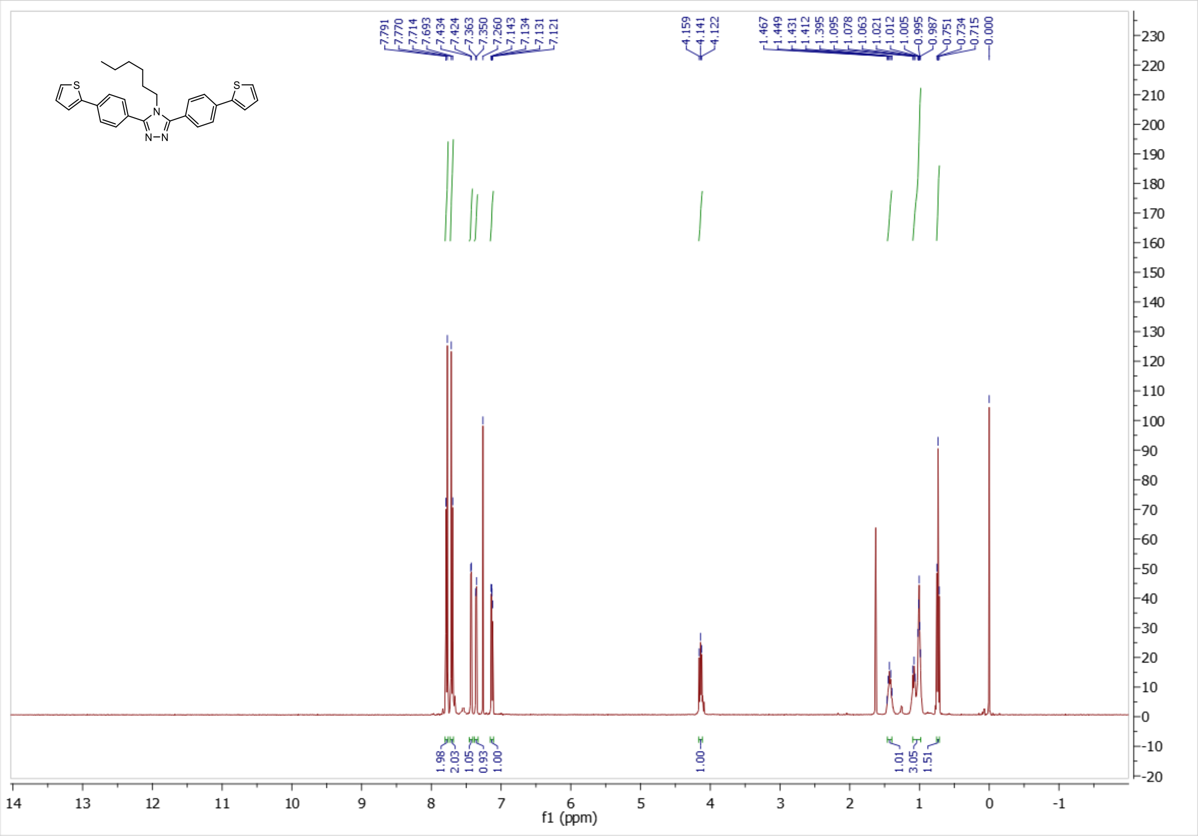

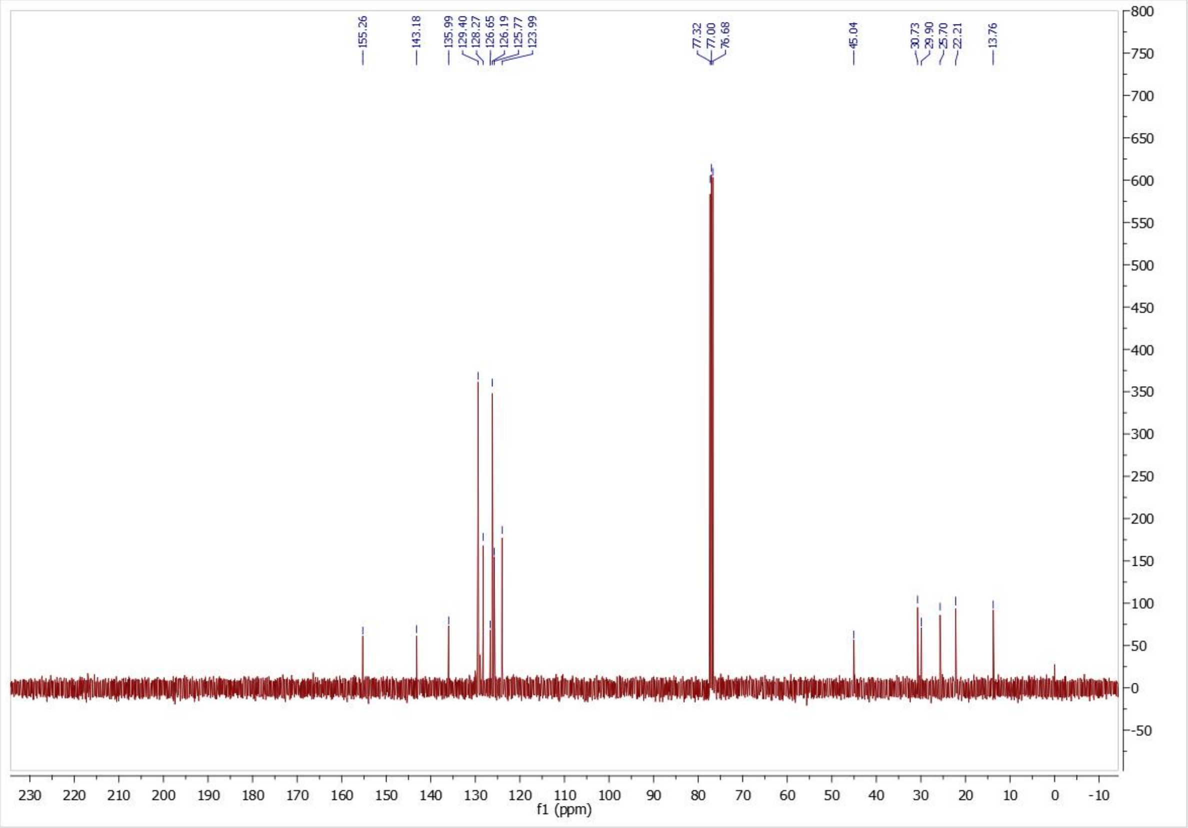

^{1}H and ^{13}C NMR of 4-hexyl-3,5-bis[4-(thiophen-3-yl)phenyl]-4*H*-1,2,4-triazole **(8n)**

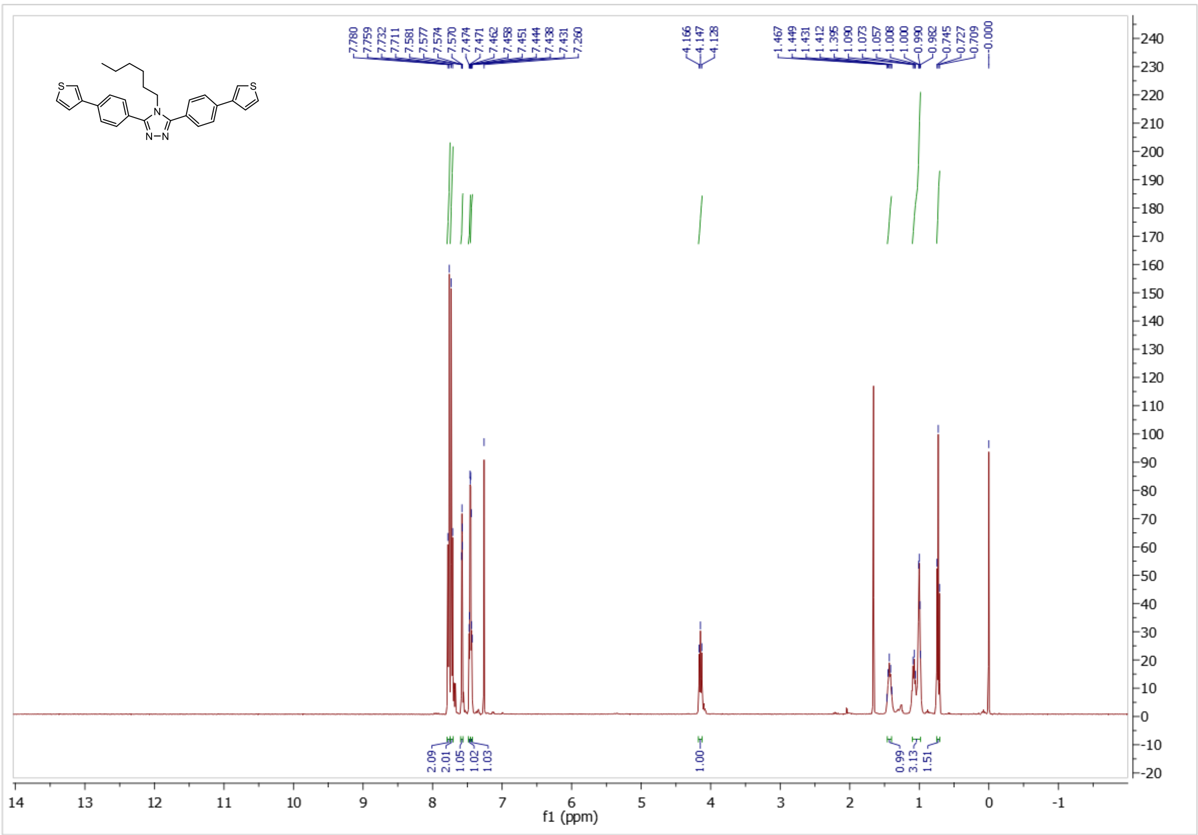

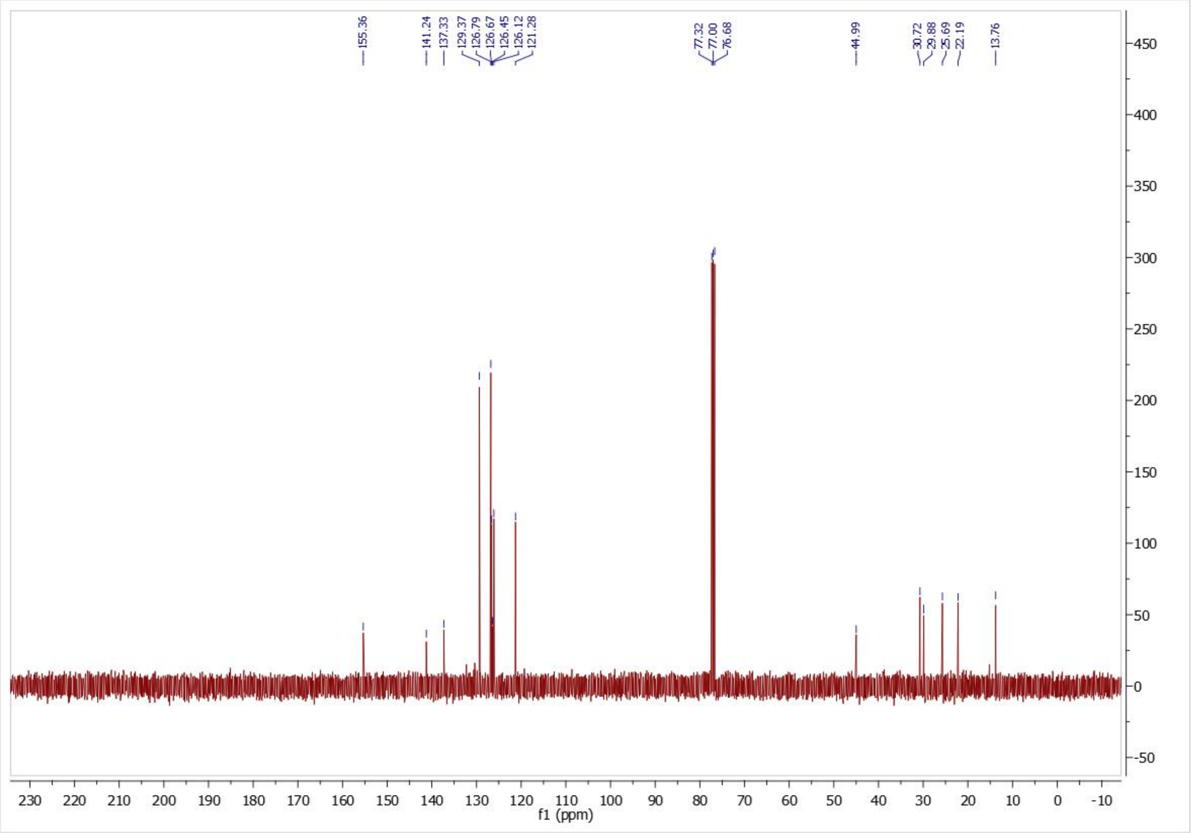

2. 3D emission spectra of compounds

Emission spectrum of 3,5-bis(biphenyl-4-yl)-4-ethyl-4*H*-1,2,4-triazole **(5a)**

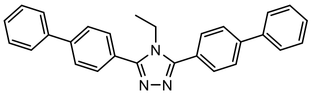

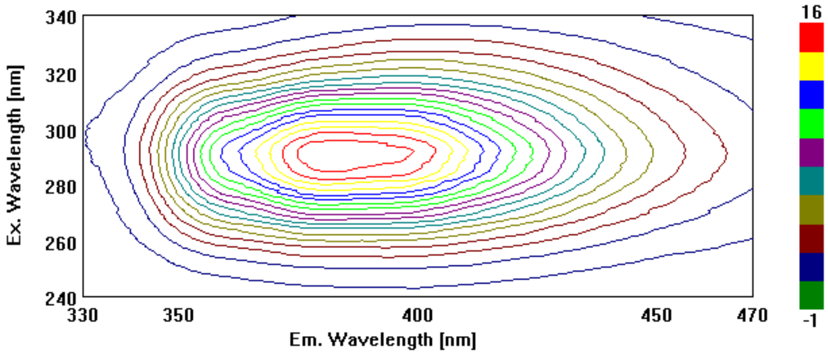

Emission spectrum of 4-ethyl-3,5-bis(2'-methylbiphenyl-4-yl)-4*H*-1,2,4-triazole **(5b)**

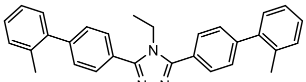

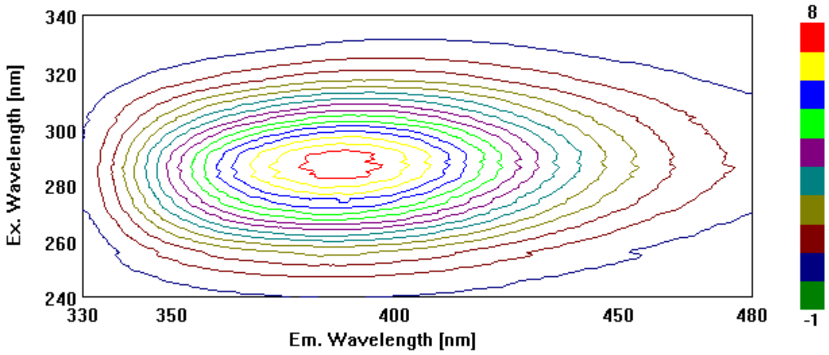

Emission spectrum of 4-ethyl-3,5-bis(3'-methylbiphenyl-4-yl)-4*H*-1,2,4-triazole **(5c)**

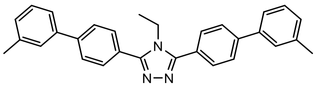

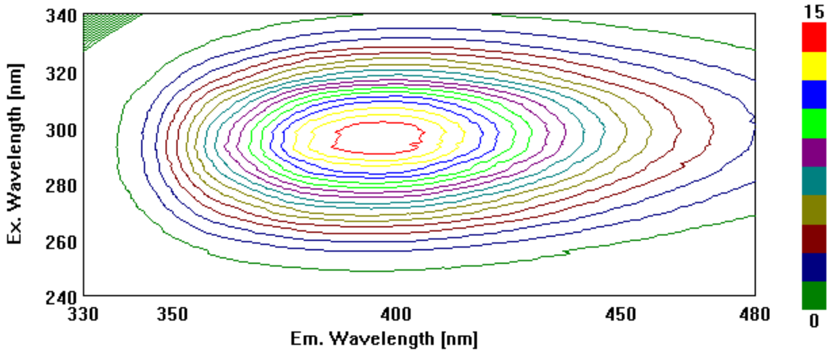

Emission spectrum of 4-ethyl-3,5-bis(2',6'-dimethylbiphenyl-4-yl)-4*H*-1,2,4-triazole **(5d)**

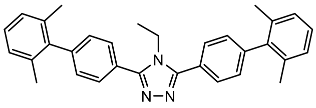

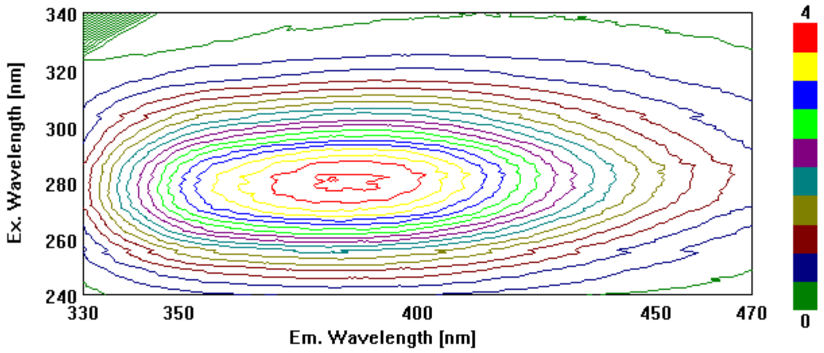

Emission spectrum of 4-ethyl-3,5-bis(2’-methoxybiphenyl-4-yl)-4*H*-1,2,4-triazole **(5e)**

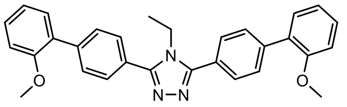

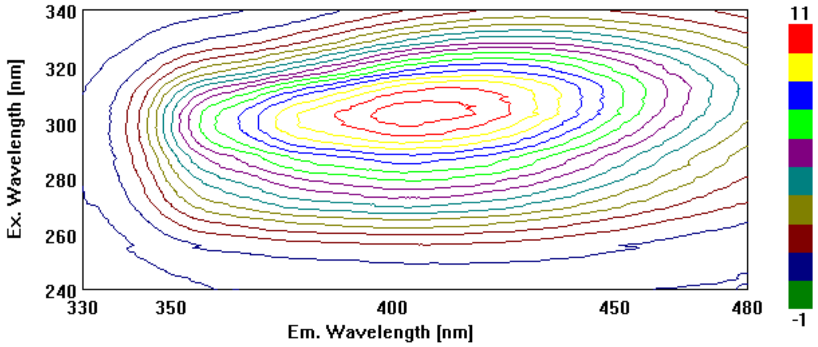

Emission spectrum of 4-ethyl-3,5-bis(3’-methoxybiphenyl-4-yl)-4*H*-1,2,4-triazole **(5f)**

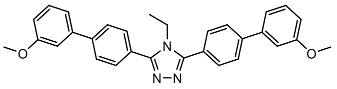

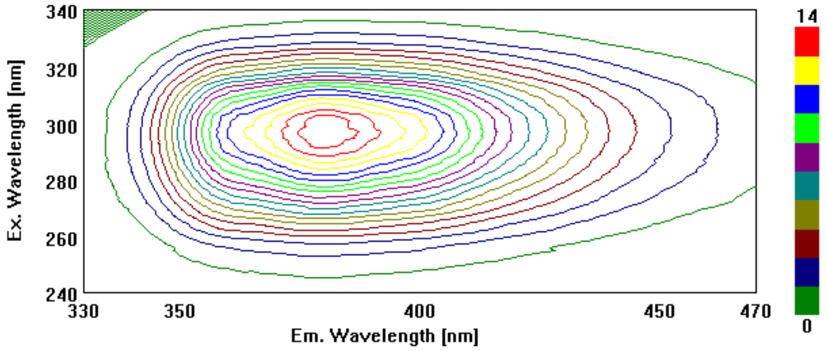

Emission spectrum of 4-ethyl-3,5-bis(3’-nitrobiphenyl-4-yl)-4*H*-1,2,4-triazole **(5g)**

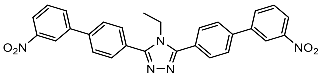

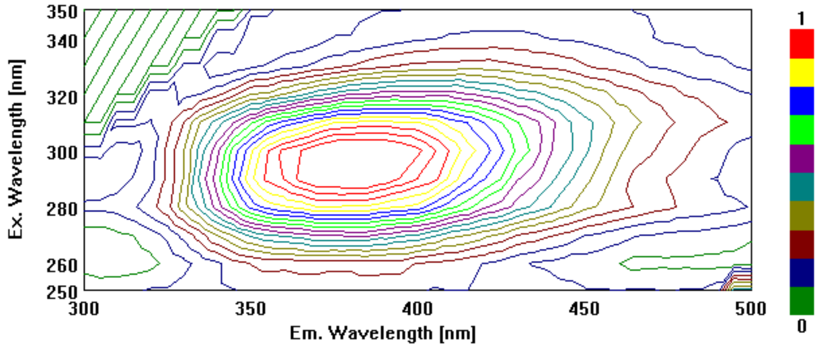

Emission spectrum of 3,5-bis(3’-aminobiphenyl-4-yl)-4-ethyl-4*H*-1,2,4-triazole **(5h)**

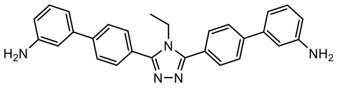

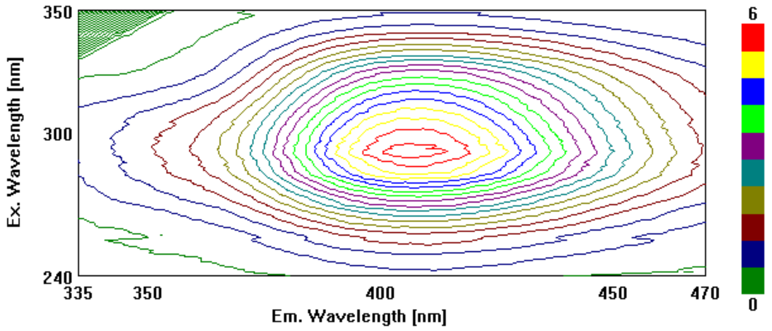

Emission spectrum of 4-ethyl-3,5-bis[4-(pyridin-4-yl)phenyl]-4*H*-1,2,4-triazole **(5i)**

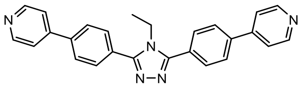

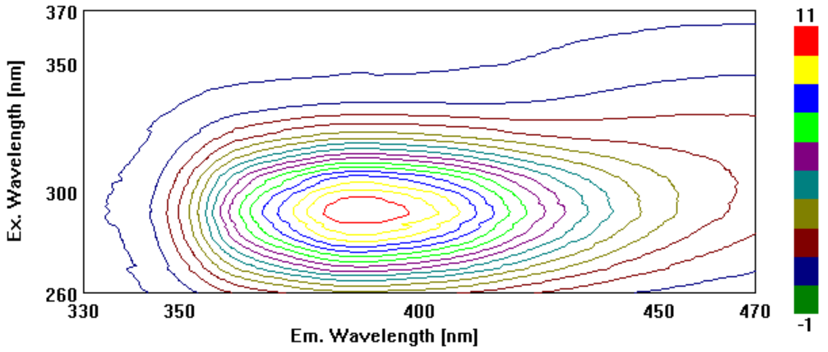

Emission spectrum of 4-ethyl-3,5-bis[4-(pyridin-3-yl)phenyl]-4*H*-1,2,4-triazole **(5j)**

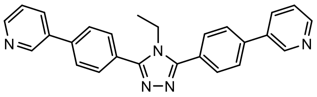

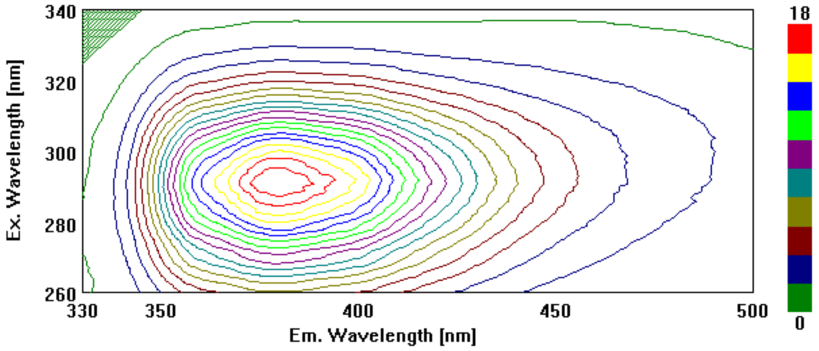

Emission spectrum of 4-ethyl-3,5-bis[4-(furan-2-yl)phenyl]-4*H*-1,2,4-triazole **(5k)**

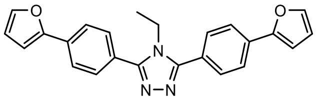

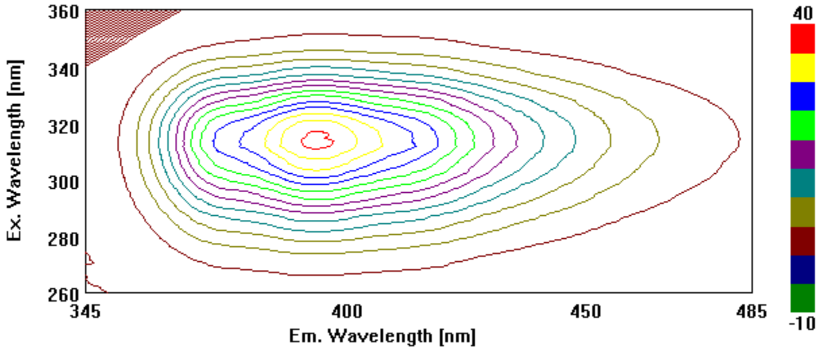

Emission spectrum of 4-ethyl-3,5-bis[4-(furan-3-yl)phenyl]-4*H*-1,2,4-triazole **(5l)**

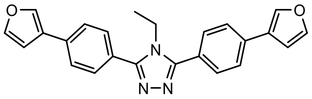

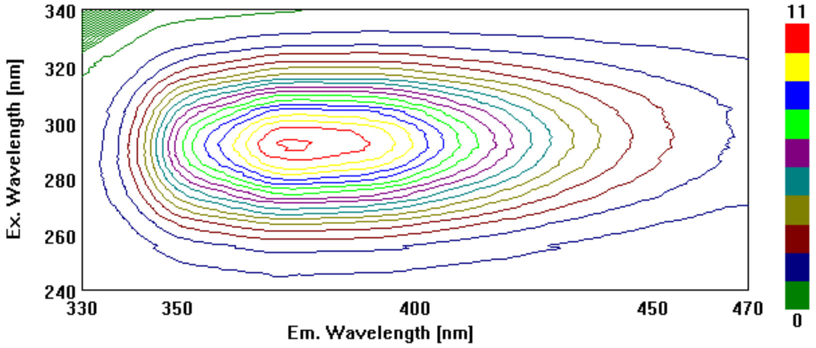

Emission spectrum of 4-ethyl-3,5-bis[4-(thiophen-2-yl)phenyl]-4*H*-1,2,4-triazole **(5m)**

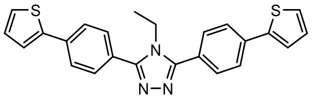

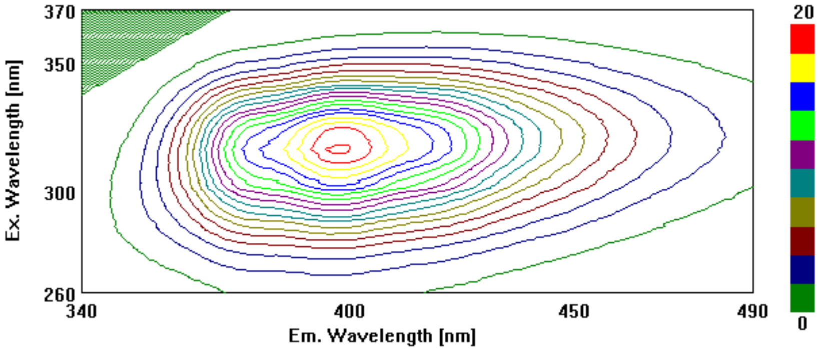

Emission spectrum of 4-ethyl-3,5-bis[4-(thiophen-3-yl)phenyl]-4*H*-1,2,4-triazole **(5n)**

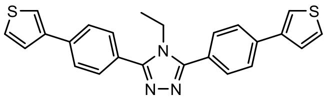

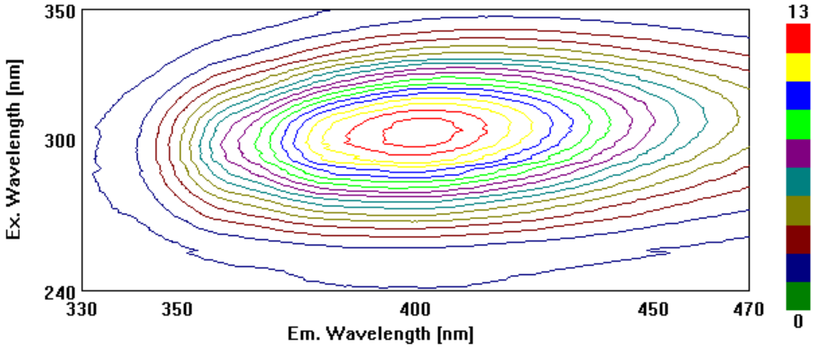

Emission spectrum of 3,5-bis(biphenyl-4-yl)-4-propyl-4*H*-1,2,4-triazole **(6a)**

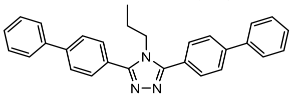

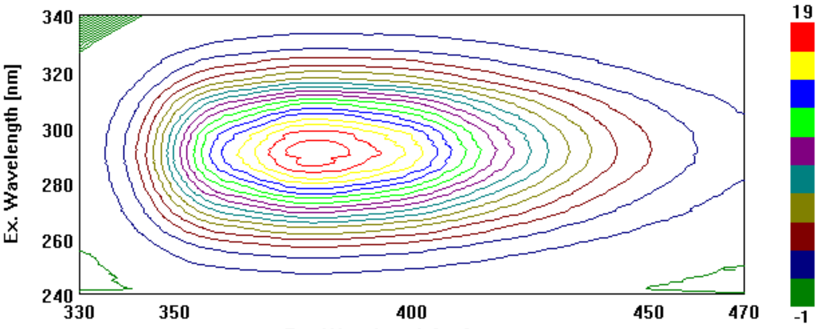

Emission spectrum of 3,5-bis(2'-methylbiphenyl-4-yl)-4-propyl-4*H*-1,2,4-triazole **(6b)**

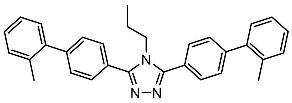

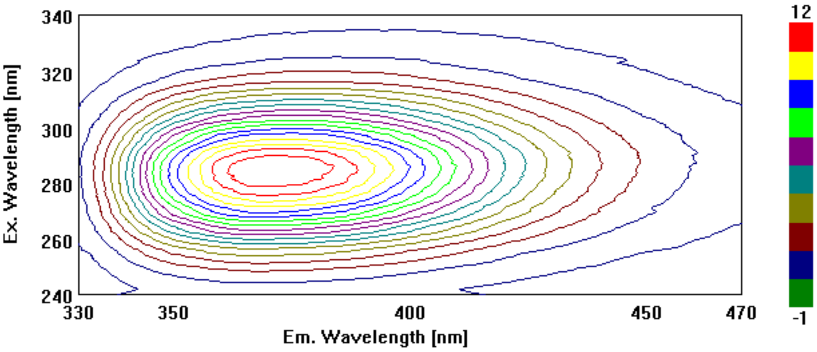

Emission spectrum of 3,5-bis(3’-methylbiphenyl-4-yl)-4-propyl-4*H*-1,2,4-triazole **(6c)**

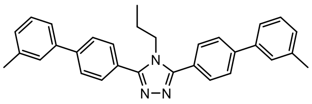

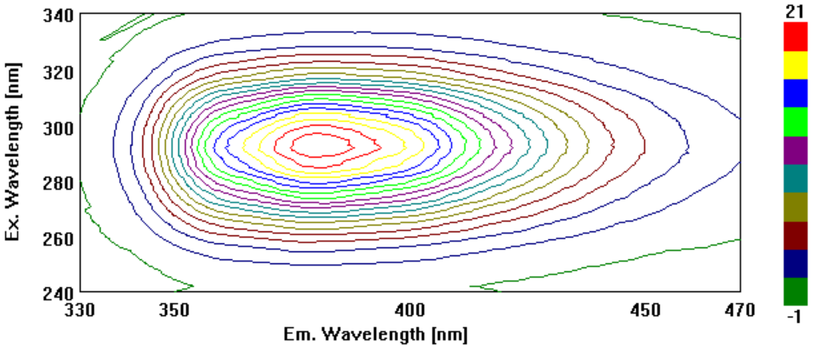

Emission spectrum of 3,5-bis(2’,6’-dimethylbiphenyl-4-yl)-4-propyl-4*H*-1,2,4-triazole **(6d)**

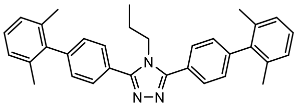

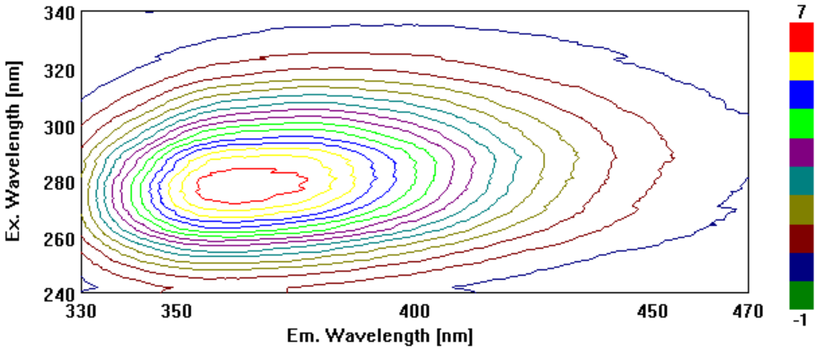

Emission spectrum of 3,5-bis(2’-methoxybiphenyl-4-yl)-4-propyl-4*H*-1,2,4-triazole **(6e)**

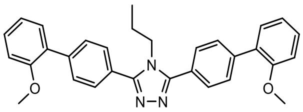

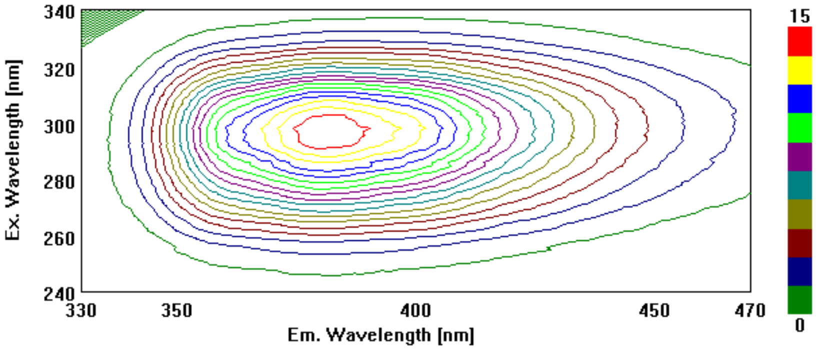

Emission spectrum of 3,5-bis(3’-methoxybiphenyl-4-yl)-4-propyl-4*H*-1,2,4-triazole **(6f)**

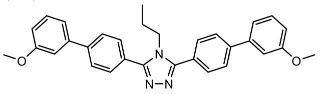

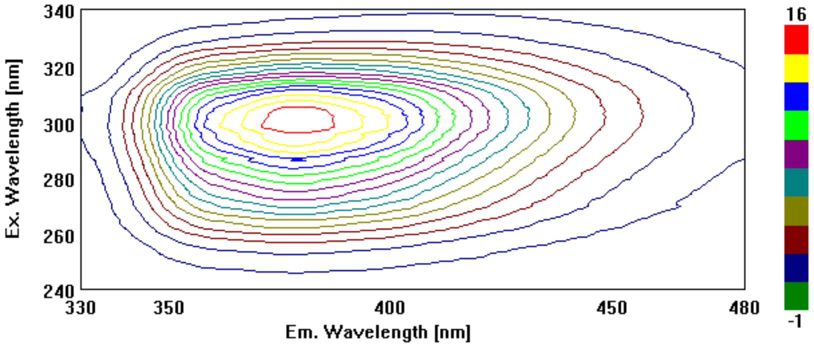

Emission spectrum of 3,5-bis(3’-nitrobiphenyl-4-yl)-4-propyl-4*H*-1,2,4-triazole **(6g)**

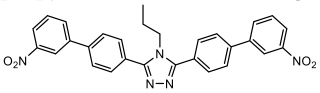

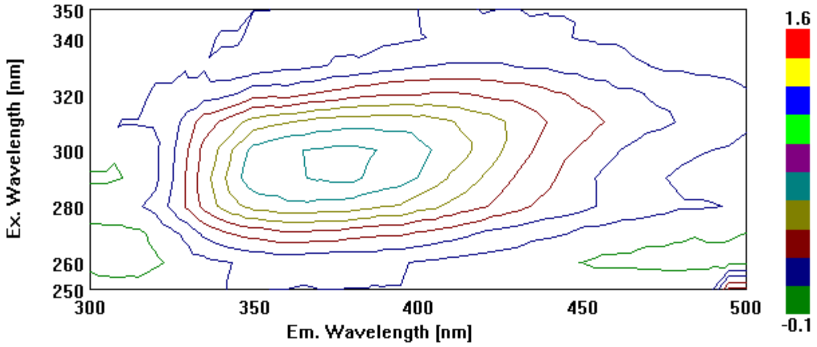

Emission spectrum of 3,5-bis(3’-aminobiphenyl-4-yl)-4-propyl-4*H*-1,2,4-triazole **(6h)**

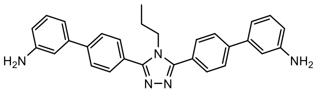

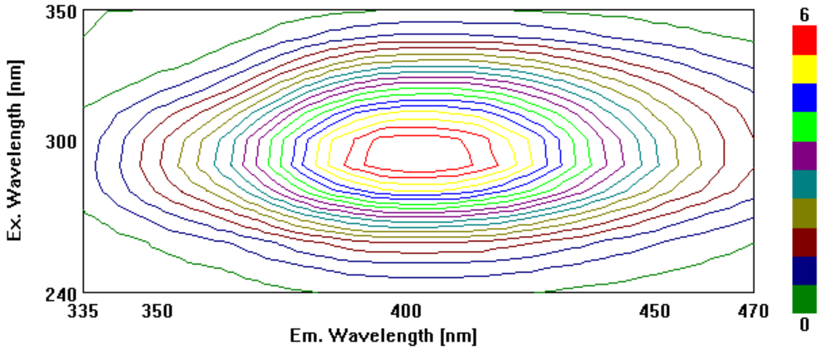

Emission spectrum of 4-propyl-3,5-bis[4-(pyridin-4-yl)phenyl]-4*H*-1,2,4-triazole **(6i)**

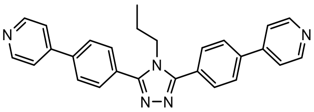

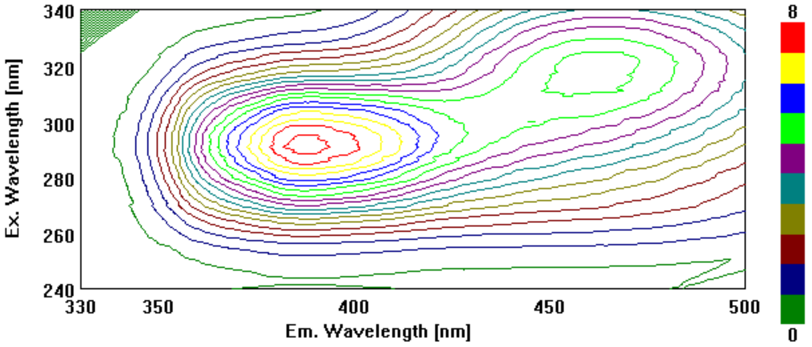

Emission spectrum of 4-propyl-3,5-bis[4-(pyridin-3-yl)phenyl]-4*H*-1,2,4-triazole **(6j)**

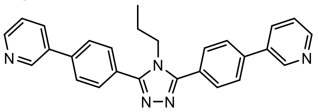

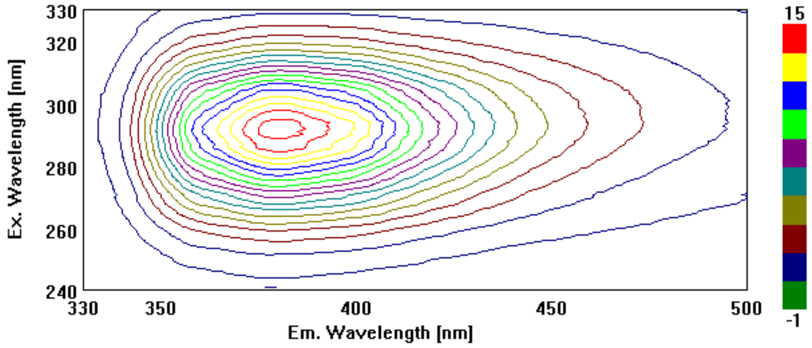

Emission spectrum of 3,5-bis[4-(furan-2-yl)phenyl]-4-propyl-4*H*-1,2,4-triazole **(6k)**

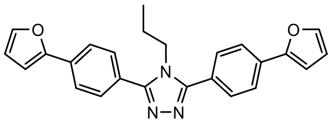

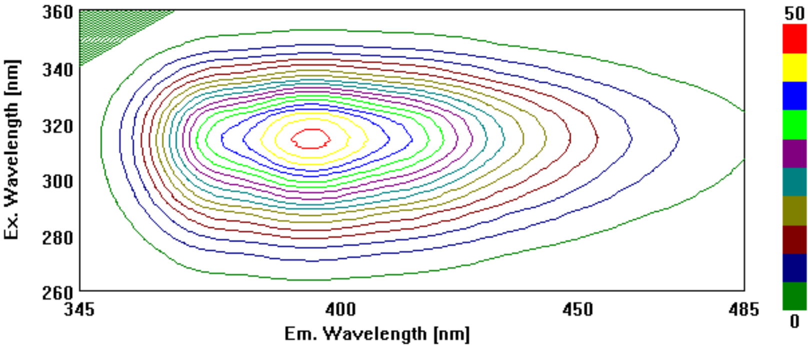

Emission spectrum of 3,5-bis[4-(furan-3-yl)phenyl]-4-propyl-4*H*-1,2,4-triazole **(6l)**

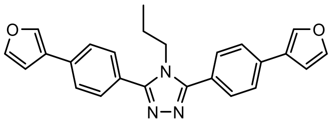

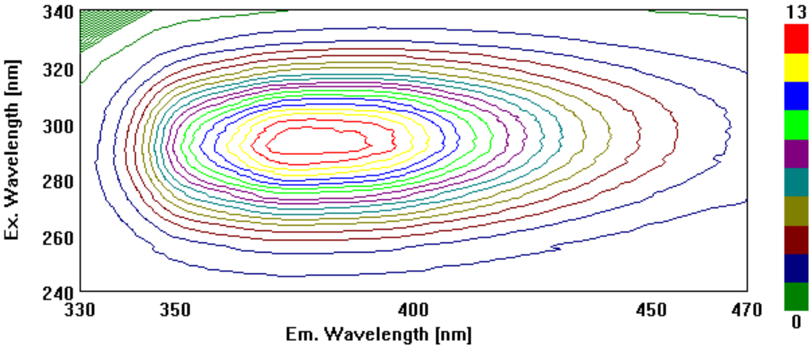

Emission spectrum of 4-propyl-3,5-bis[4-(thiophen-2-yl)phenyl]-4*H*-1,2,4-triazole **(6m)**

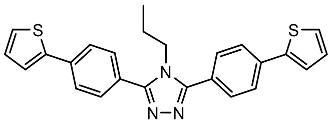

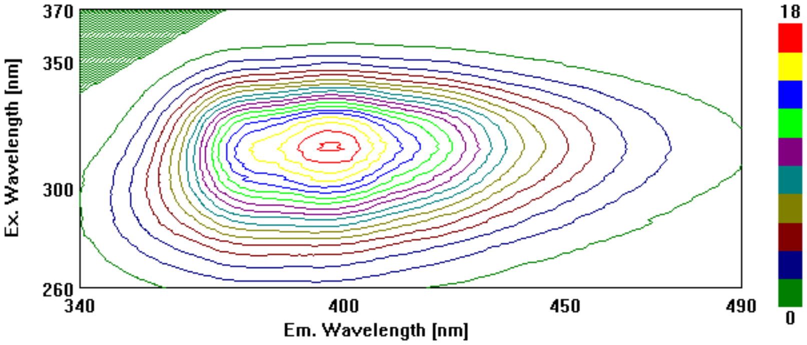

Emission spectrum of 4-propyl-3,5-bis[4-(thiophen-3-yl)phenyl]-4*H*-1,2,4-triazole **(6n)**

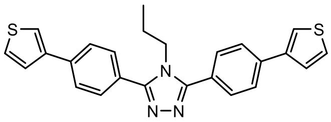

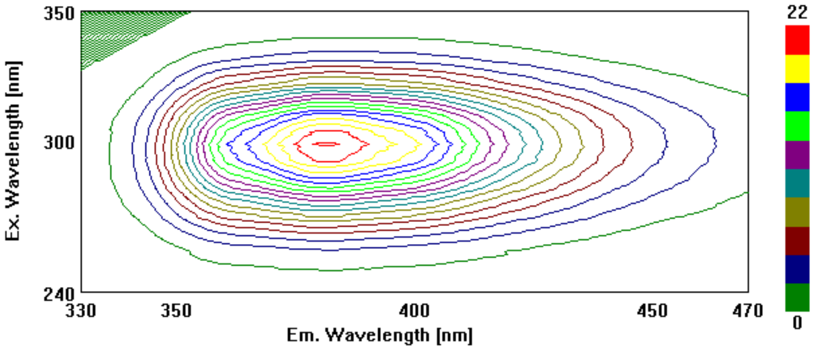

Emission spectrum of 3,5-bis(biphenyl-4-yl)-4-butyl-4*H*-1,2,4-triazole **(7a)**

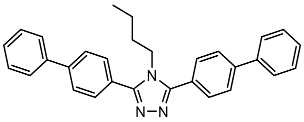

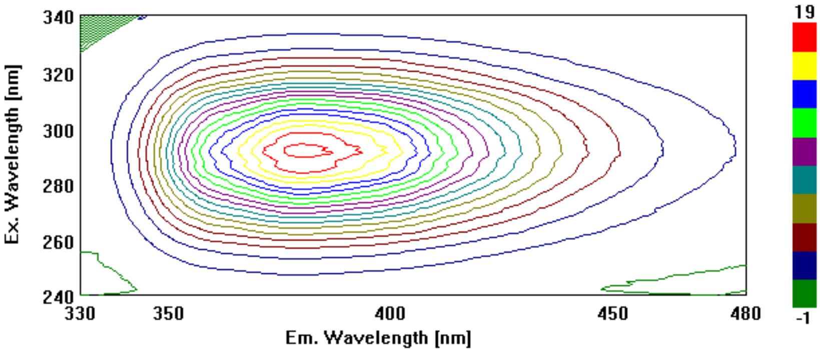

Emission spectrum of 4-butyl-3,5-bis(2'-methylbiphenyl-4-yl)-4*H*-1,2,4-triazole **(7b)**

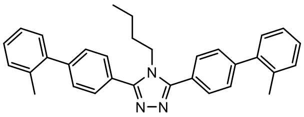

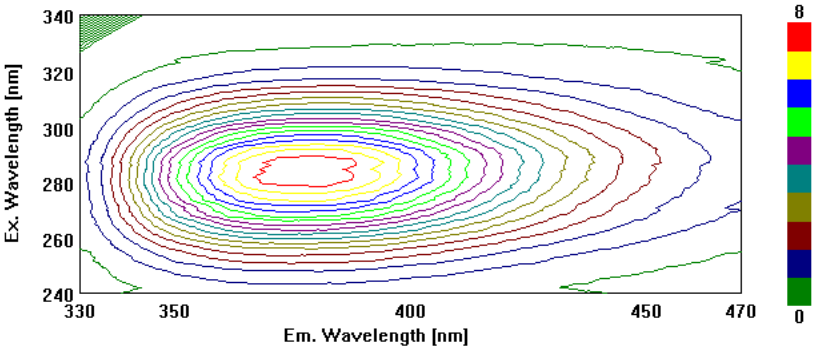

Emission spectrum of 4-butyl-3,5-bis(3'-methylbiphenyl-4-yl)-4*H*-1,2,4-triazole **(7c)**

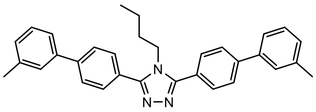

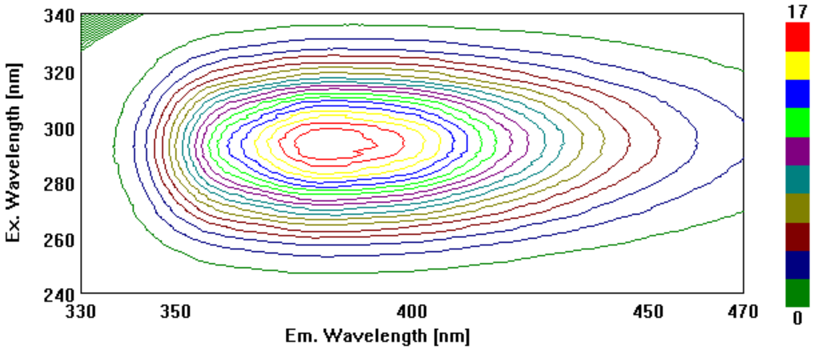

Emission spectrum of 4-butyl-3,5-bis(2',6'-dimethylbiphenyl-4-yl)-4*H*-1,2,4-triazole **(7d)**

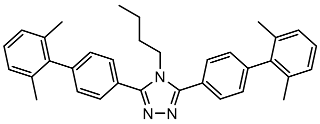

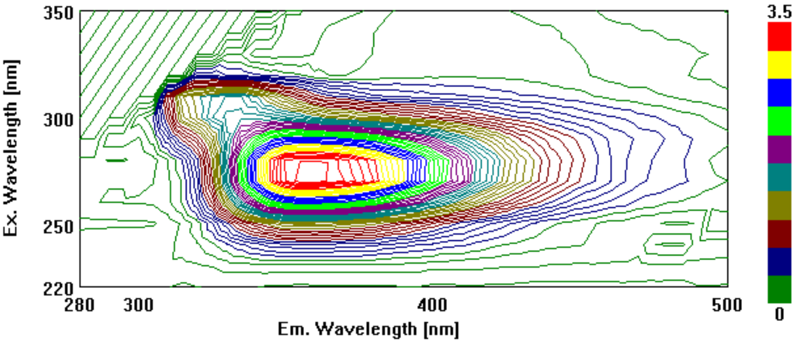

Emission spectrum of 4-butyl-3,5-bis(2'-methoxybiphenyl-4-yl)-4*H*-1,2,4-triazole **(7e)**

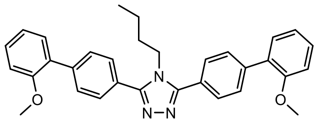

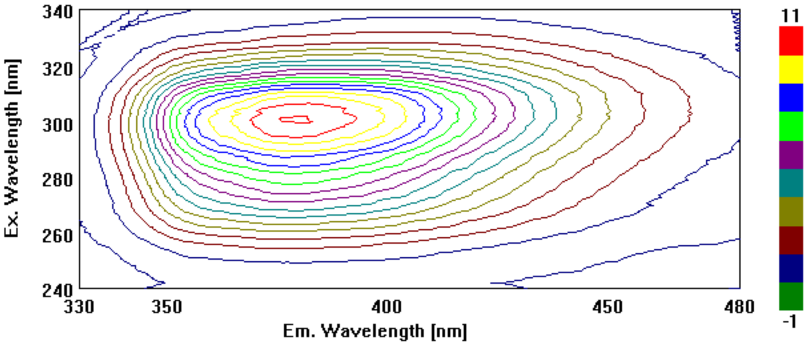

Emission spectrum of 4-butyl-3,5-bis(3'-methoxybiphenyl-4-yl)-4*H*-1,2,4-triazole **(7f)**

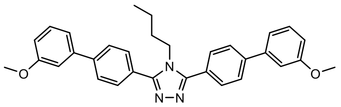

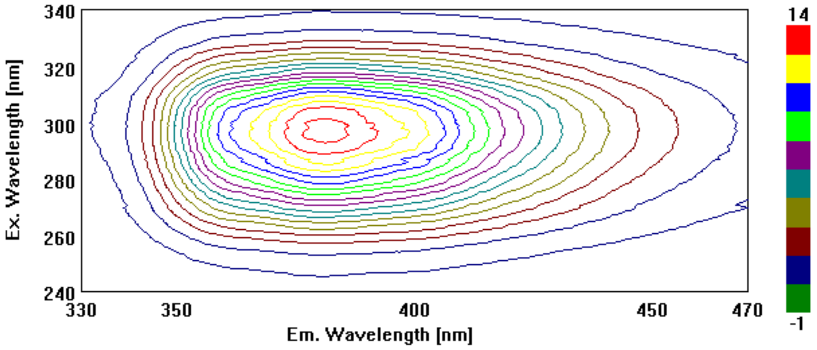

Emission spectrum of 4-butyl-3,5-bis[(3'-nitrobiphenyl-4-yl)-4*H*-1,2,4-triazole **(7g)**

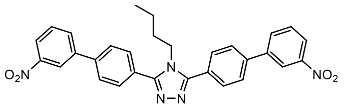

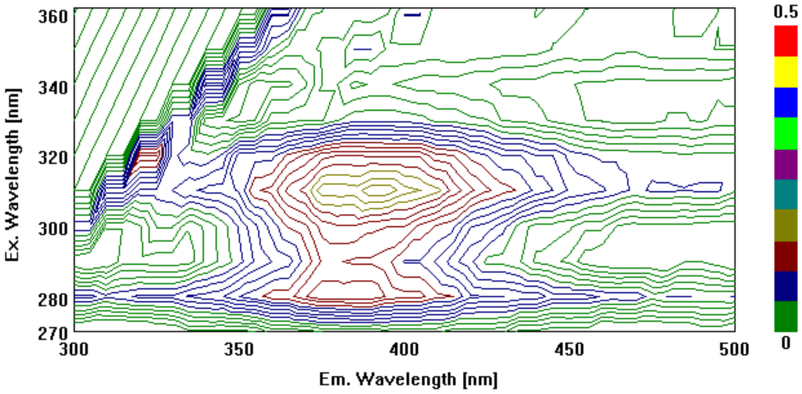

Emission spectrum of 3,5-bis(3'-aminobiphenyl-4-yl)-4-butyl-4*H*-1,2,4-triazole **(7h)**

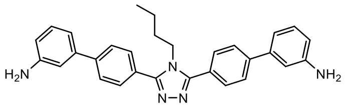

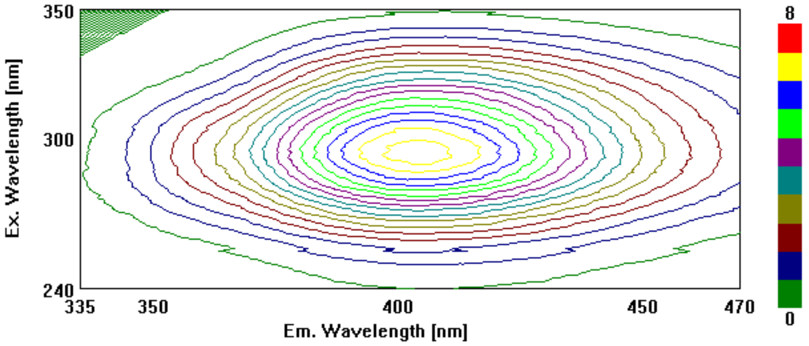

Emission spectrum of 4-butyl-3,5-bis[4-(pyridin-4-yl)phenyl]-4*H*-1,2,4-triazole **(7i)**

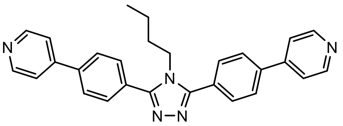

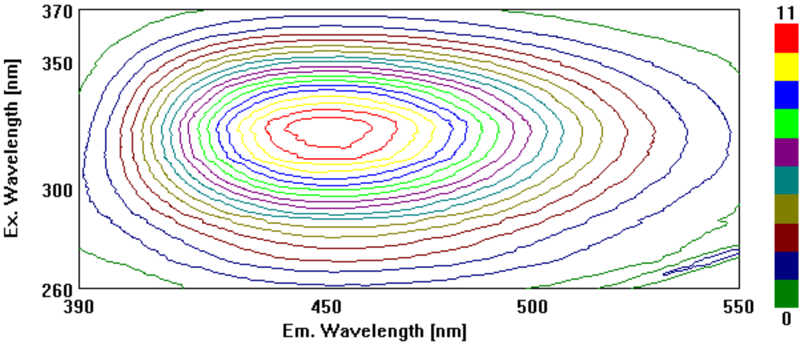

Emission spectrum of 4-butyl-3,5-bis[4-(pyridin-3-yl)phenyl]-4*H*-1,2,4-triazole **(7j)**

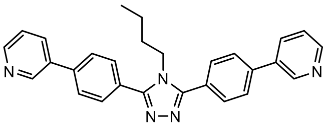

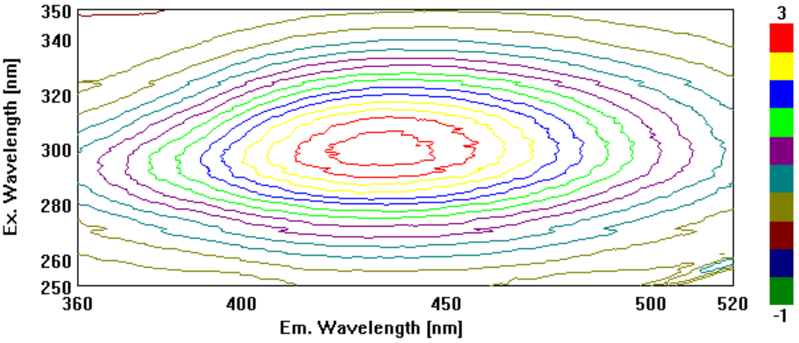

Emission spectrum of 4-butyl-3,5-bis[4-(furan-2-yl)phenyl]-4*H*-1,2,4-triazole **(7k)**

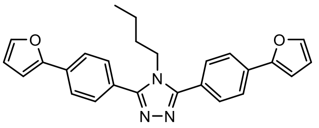

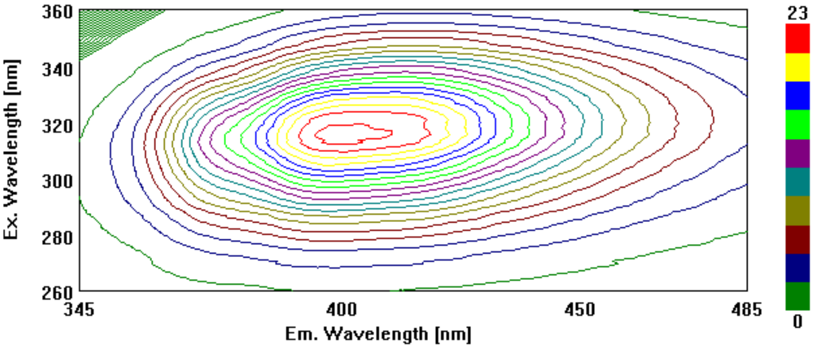

Emission spectrum of 4-butyl-3,5-bis[4-(furan-3-yl)phenyl]-4*H*-1,2,4-triazole **(7l)**

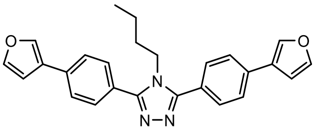

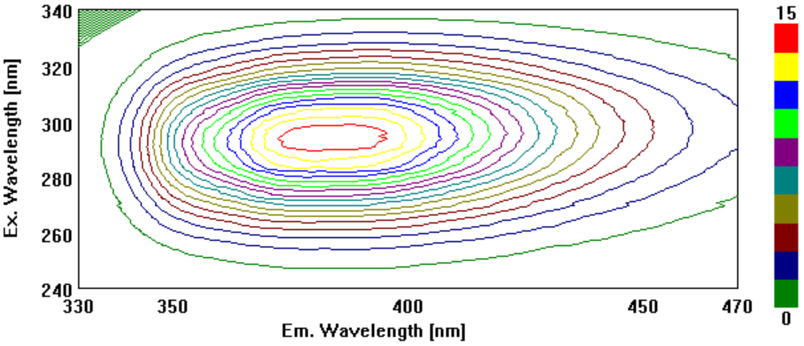

Emission spectrum of 4-butyl-3,5-bis[4-(thiophen-2-yl)phenyl]-4*H*-1,2,4-triazole **(7m)**

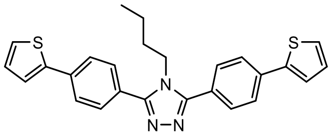

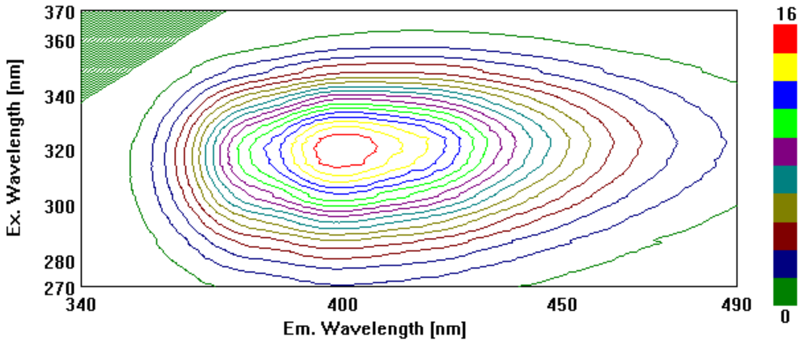

Emission spectrum of 4-butyl-3,5-bis[4-(thiophen-3-yl)phenyl]-4*H*-1,2,4-triazole **(7n)**

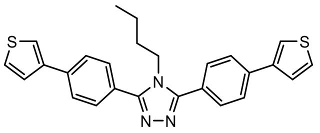

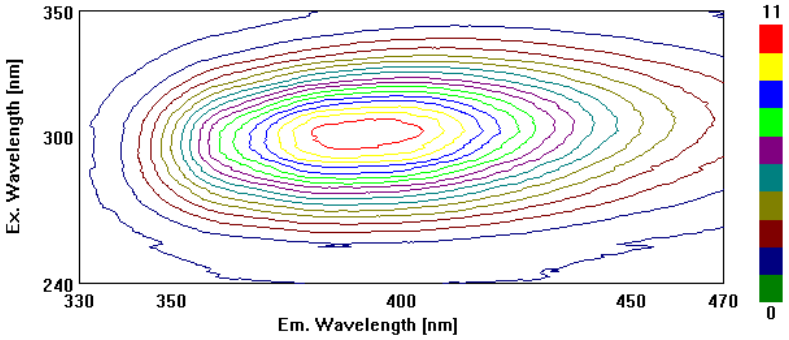

Emission spectrum of 3,5-bis(biphenyl-4-yl)-4-hexyl-4*H*-1,2,4-triazole **(8a)**

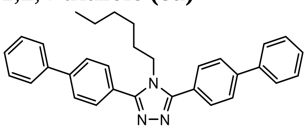

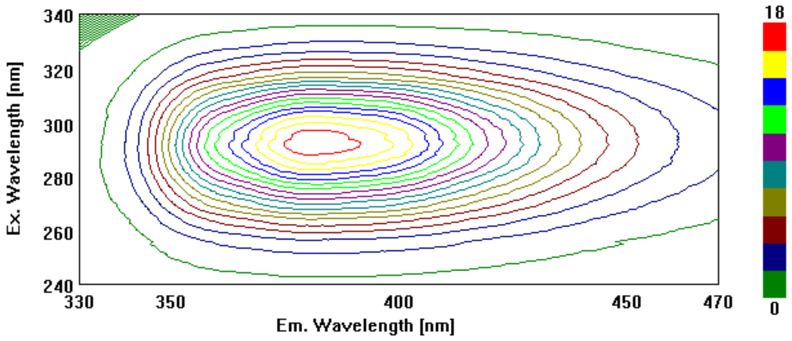

Emission spectrum of 4-hexyl-3,5-bis(2'-methylbiphenyl-4-yl)-4*H*-1,2,4-triazole **(8b)**

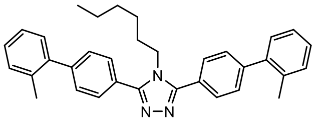

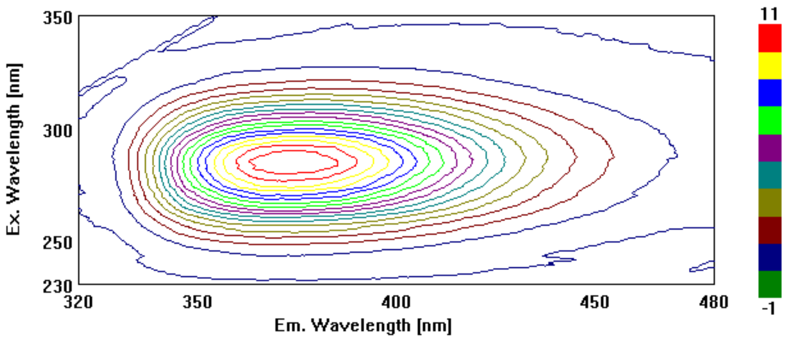

Emission spectrum of 4-hexyl-3,5-bis(3’-methylbiphenyl-4-yl)-4*H*-1,2,4-triazole **(8c)**

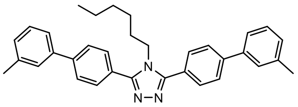

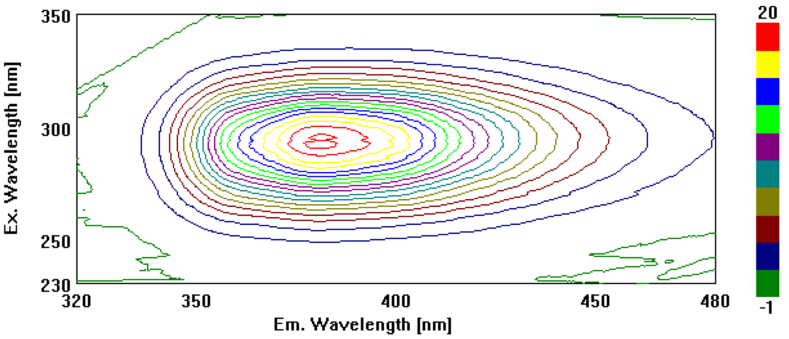

Emission spectrum of 4-hexyl-3,5-bis(2’,6’-dimethylbiphenyl-4-yl)-4*H*-1,2,4-triazole **(8d)**

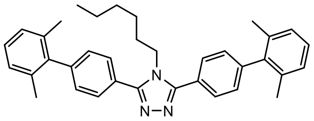

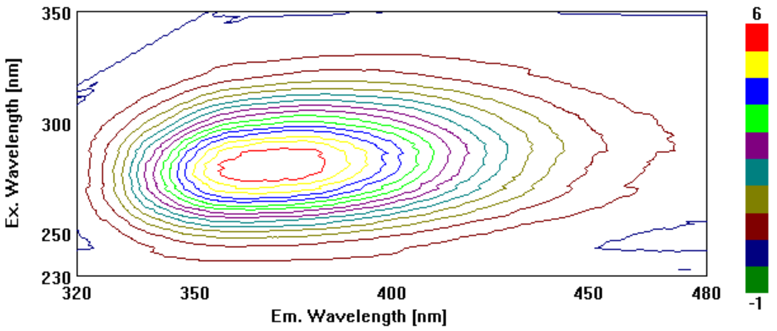

Emission spectrum of 4-hexyl-3,5-bis(2’-methoxybiphenyl-4-yl)-4*H*-1,2,4-triazole **(8e)**

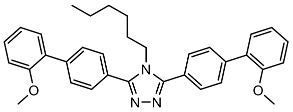

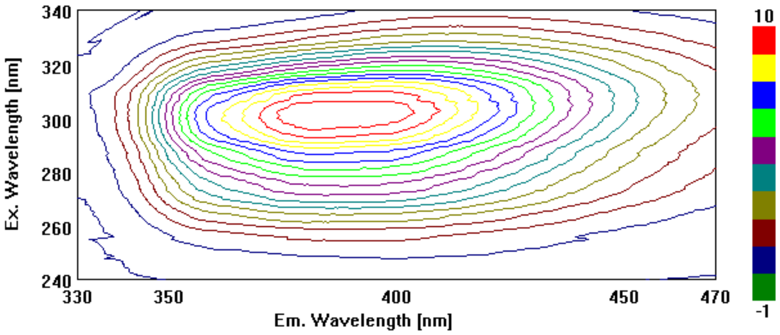

Emission spectrum of 4-hexyl-3,5-bis(3’-methoxybiphenyl-4-yl)-4*H*-1,2,4-triazole **(8f)**

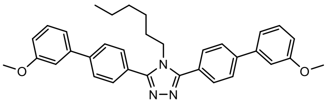

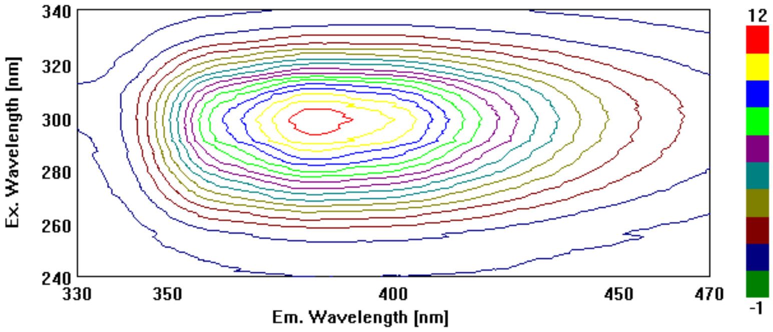

Emission spectrum of 4-hexyl-3,5-bis(3'-nitrobiphenyl-4-yl)-4*H*-1,2,4-triazole **(8g)**

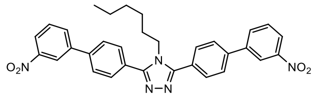

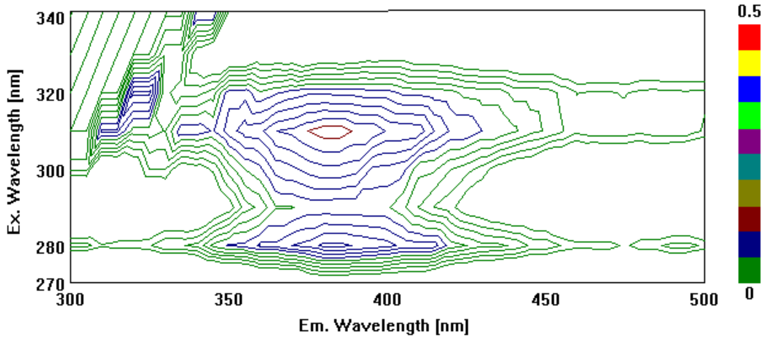

Emission spectrum of 3,5-bis(3'-aminobiphenyl-4-yl)-4-hexyl-4*H*-1,2,4-triazole **(8h)**

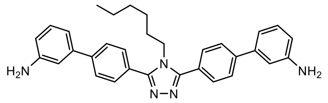

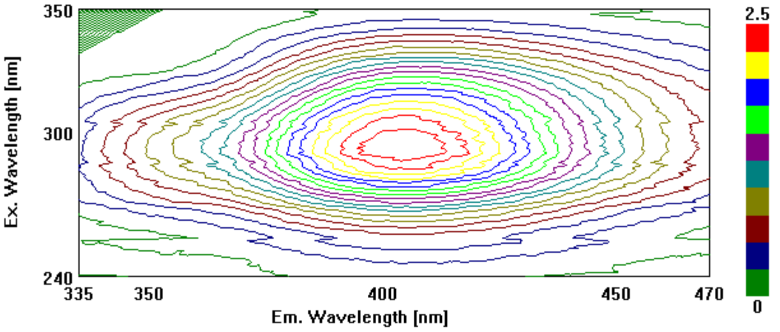

Emission spectrum of 4-hexyl-3,5-bis[4-(pyridin-4-yl)phenyl]-4*H*-1,2,4-triazole **(8i)**

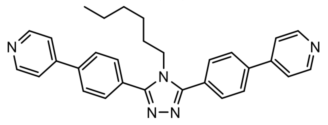

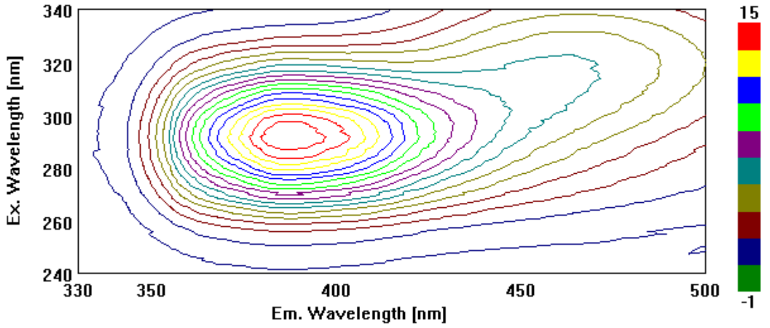

Emission spectrum of 4-hexyl-3,5-bis[4-(pyridin-3-yl)phenyl]-4*H*-1,2,4-triazole **(8j)**

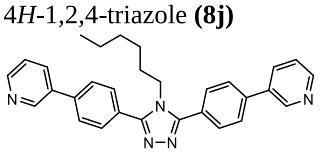

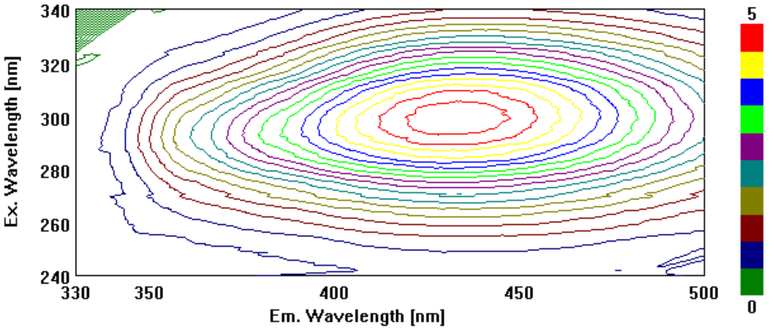

Emission spectrum of 3,5-bis[4-(furan-2-yl)phenyl]-4-hexyl-4*H*-1,2,4-triazole **(8k)**

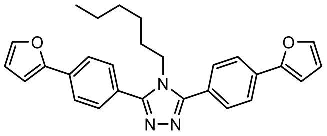

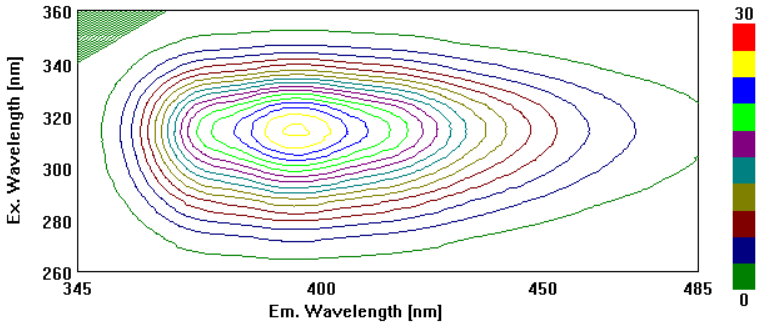

Emission spectrum of 3,5-bis[4-(furan-3-yl)phenyl]-4-hexyl-4*H*-1,2,4-triazole **(8l)**

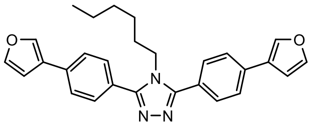

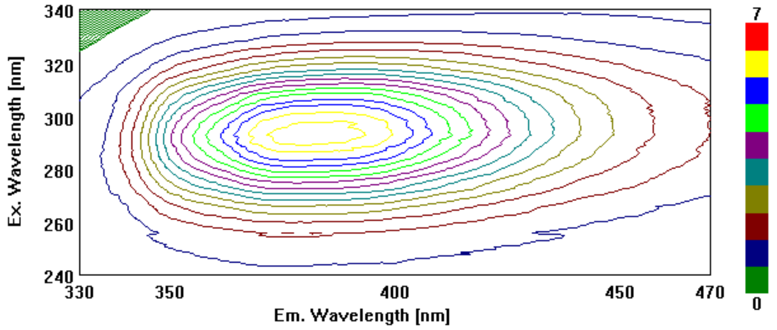

Emission spectrum of 4-hexyl-3,5-bis[4-(thiophen-2-yl)phenyl]-4*H*-1,2,4-triazole **(8m)**

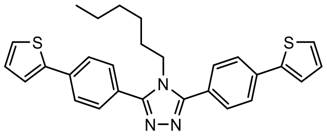

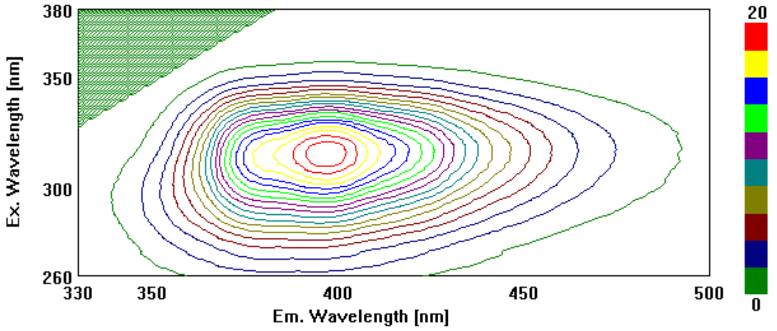

Emission spectrum of 4-hexyl-3,5-bis[4-(thiophen-3-yl)phenyl]-4*H*-1,2,4-triazole **(8n)**

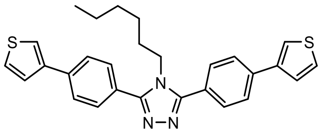

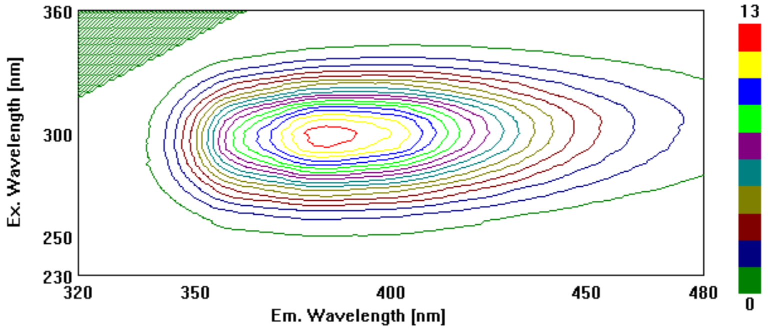

Supplement: Supplementary file 1 [file molecules-24-00652-s001.pdf]
